# Supplementary material for: Simultaneous construction of axial and planar chirality by gold/TY-Phos-catalyzed asymmetric hydroarylation
Source: Nat Commun. 2021 Jul 29;12:4609. doi: 10.1038/s41467-021-24678-5 (PMC8322429; doi:10.1038/s41467-021-24678-5)
Supplement: Supplementary file 1 — Supplementary Information [file 41467_2021_24678_MOESM1_ESM.pdf]

Supplementary Information

Simultaneous Construction of Axial and Planar Chirality by Gold/TY-Phos-Catalyzed Asymmetric Hydroarylation

Pei-Chao Zhang, Yin-Lin Li, Jiafeng He, Hai-Hong Wu, Zhiming Li*, and Junliang Zhang**

Contents

1. General Information: 2
2. X-ray of (*Sa,Sp*)-**2bb**, (*Sc,Rs*)-Au(**TY6**)Cl 3
3. The optimized transition states model. 3
4. Supplementary Proof-of-principle study. 3
5. Optimization of chiral catalyst. 4
6. Synthesis of Au(**TY-Phos**)Cl. 4
7. Preparation and Characterization of ortho-alkynylaryl ferrocenes derivatives **1**, **3** and **4**. 7
8. General procedure for Simultaneous Construction of Axial and Planar Chirality in Ferrocene Derivatives by Gold/TY-Phos-Catalyzed Asymmetric Hydroarylation. 18
9. Gram-scale synthesis. 43
10. Synthetic applications of the products 43
11. ^{1}H and ^{13}C NMR Spectra for new compouds 45
12. Supplementary References 142

1. General Information:

Unless otherwise noted, all reactions were carried out in standard Schlenk techniques with magnetic stirring bar under air. Materials obtained from commercial suppliers were used directly without further purification. ^{1}H NMR spectra were recorded on a BRUKER 400 (400 MHz) and 500 (125 MHz) spectrometer in $CDCl_3$ or THF-d_8. Chemical shifts are reported in ppm with tetramethylsilane (TMS: 0 ppm) with the solvent resonance as the internal standard. Data are reported as follows: chemical shift, multiplicity (s = singlet, d = doublet, t = triplet, q = quartet, m = multiplet), coupling constants (Hz), and integration. ^{13}C NMR spectra were recorded on a BRUKER 400 (100 MHz) and 500 (125 MHz) spectrometer in $CDCl_3$ or THF-d_8 with complete proton decoupling. Chemical shifts are reported in ppm with the deuterium solvent as the internal standard (e.g. $CDCl_3$: 77.0 ppm; THF-d_8: 25.1, 67.4); The $[\alpha]^D$ was recorded using PolAAr 3005 High Accuracy Polarimeter. The *ee* was recorded using LC-2030C 3D from Shimadzu Company. Flash column chromatography was performed over silica gel (10-40 mesh); The substrates **1**[1], **2**[2] and **4**[3] were synthesized according to published procedures. The spectral data of the substrates were consisted with that reported in the literature. The enantionmeric excesses of the products were determined by chiral stationary phase HPLC using a Chiralpak ADH, ODH, IC.

2. X-ray of (*Sa*,*Sp*)-2bb, (*Sc*,*Rs*)-Au(TY6)Cl

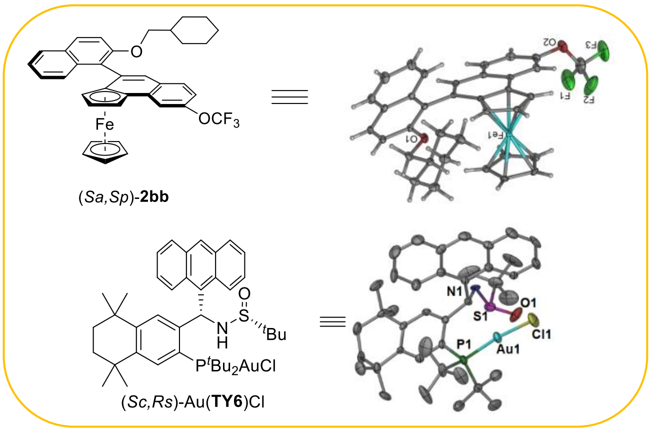

3. The optimized transition states model.

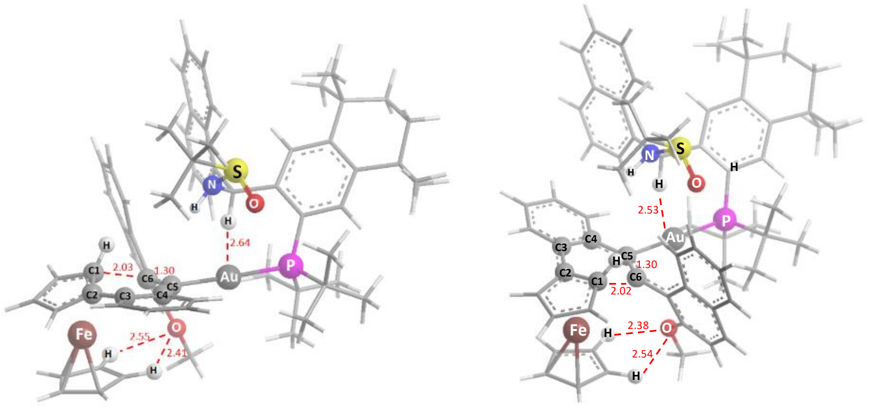

TS-A **TS-*en*-A**

Supplementary Fig. S1. The optimized transition states TS-A(left) and TS-en-A(right) for the enantioselectivity-determining step, calculated with SMD Model (Dichloromethane) with M062X method at 273 K.

4. Supplementary Proof-of-principle study.

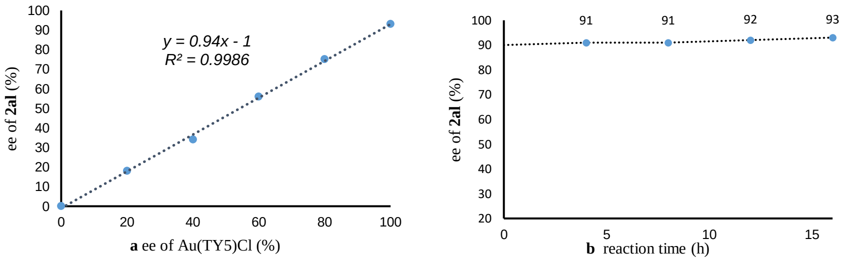

Supplementary Fig. S2 Proof-of-principle study. a Non-linear effect. **b** reaction pathway studies.

The linear relationship between the ees of the Au(TY5)Cl and those of product **2al** and the e.e. of the **2al** did not significantly change during the reaction, which reveal that the enantioselectivity determining step might involve a single chiral sulfinamide phosphine ligand and one gold species.

5. Optimization of chiral catalyst.

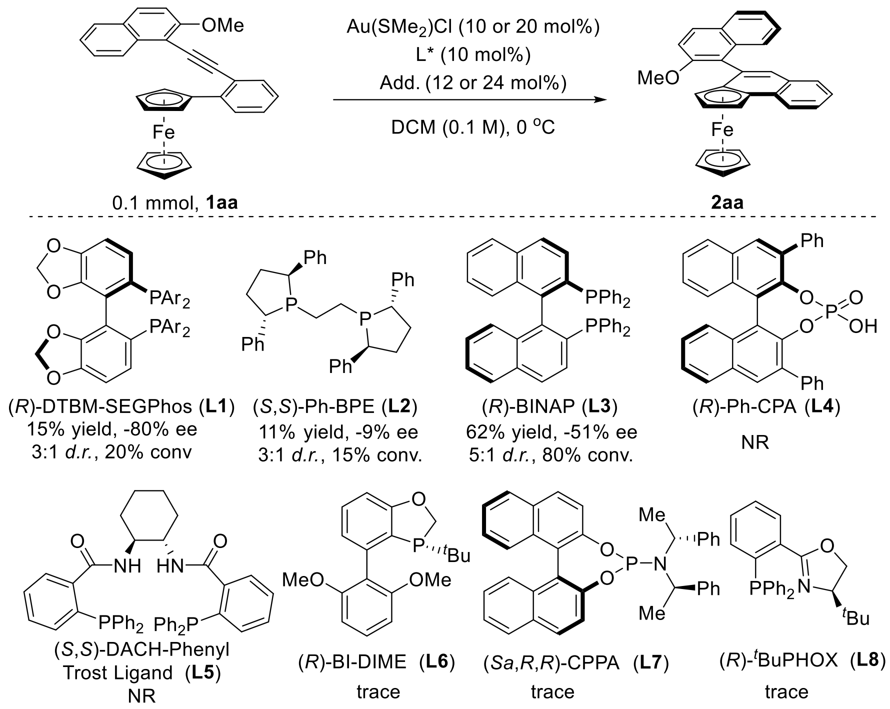

Supplementary Fig. S3. Unless otherwise noted, all reactions were carried out with 0.1 mmol of **1aa** and 10 mol% of catalyst (Au : P : $NaBAr^F$ (additive) = 1:1:1.2) in 1.0 mL DCM at 0 ºC for 24 h; yield and diastereoselectivity were determined by NMR, e.e. were determined by HPLC analysis.

6. Synthesis of Au(TY-Phos)Cl.

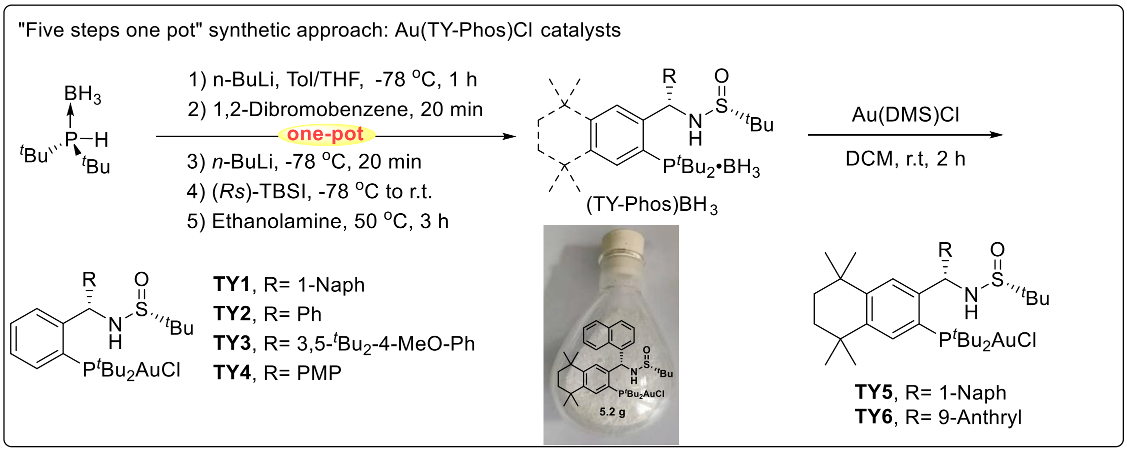

Typical procedure:

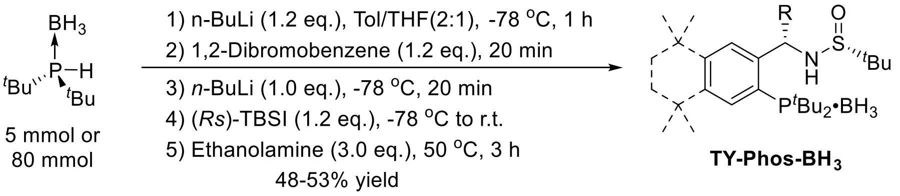

To a solution of di-tert-butylphosphine borane (5 or 80 mmol, cas: 128363-76-8) in dry Tol/THF (v:v= 2:1, 0.33 M) at -78 °C for 10 min under N_2, was added *n*-BuLi (1.2 equiv, 2.4 M in hexane) dropwise, the mixture was stirred at same temperature for 1 h, then (substituent)1,2-dibromobenzene (1.2 equiv, cas: 583-53-9 or 184885-74-3) was added dropwise, the mixture was stirred at same temperature for another 20 min, then was added *n*-BuLi (1.2 equiv, 2.4 M in hexanes,); After 20 minutes at -78 °C, (*Rs*)-tert-butyl sulfonyl imide (1.2 equiv, 1 M in Tol/THF (2:1), cas: 451503-13-2, 186249-76-3, 2241598-36-5, 244092-54-4, 336105-27-2 or 2003212-05-1) was added and the reaction mixture was warmed to room temperature overnight. Quenched by saturated aqueous solution of NH_4Cl, and then extracted with EA. The extracts were washed by saturated salt water and dried over anhydrous Na_2SO_4, then the solvent was removed under reduced pressure. The crude product **BH_3-TY-Phos** in dry THF (0.5 M) was added ethanolamine (3.0 equiv, cas: 141-43-5) and the resulting solution was stirred for 1 h under argon at 50 °C. After the reaction was complete (monitored by TLC), solvent was removed under reduced pressure. The crude product was purified by column chromatography (Petroleum ether: Acetone= 30:1 to 20:1) to give **TY-Phos** (48-53% yield) as a white solid.

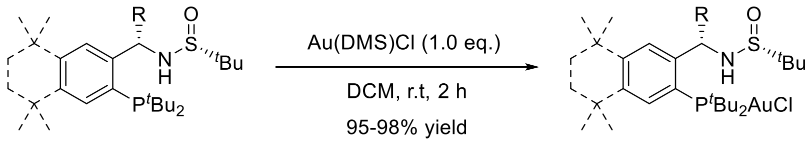

TY-Phos **Au(TY-Phos)Cl**

To a solution of **TY-Phos** (2.0 mmol) in 20 mL dry DCM at 0 °C for 10 min under N_2, was added Au(DMS)Cl (1.0 equiv, 590 mg, cas: 29892-37-3); The mixture was stirred at 0 °C for 10 min and then was warmed to room temperature with stirring 2 h. The organic layers were concentrated, and purified by flash chromatography (Hexane: EA= 3:1) to isolated **Au(TY-Phos)Cl** as white solid (95-98% yield);

6.1 (di-*tert*-butyl(2-((*S*)-(((*R*)-*tert*-butylsulfinyl)amino)(naphthalen-1-yl)methyl)phenyl)chloro-λ^5-phosphaneyl)gold ((*Sc*,*Rs*)-Au(TY1)Cl)

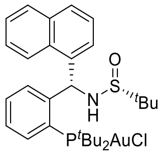

Isolated (*Sc*,*Rs*)-**Au(TY1)Cl** in 48% three steps total yield as white solid.

^{1}H NMR (500 MHz, $CDCl_3$) δ 8.72 (d, *J* = 8.5 Hz, 1H), 8.35 (d, *J* =3.0 Hz, 1H), 8.15 – 8.13 (m, 1H), 792 – 7.87 (m, 2H), 7.81 (t, *J* = 8.5 Hz, 2H), 7.71 (t, *J* = 7.5 Hz, 1H), 7.57 (t, *J* = 7.5 Hz, 1H), 7.52 (t, *J* = 7.5 Hz, 1H), 7.20 (t, *J* = 7.5 Hz, 1H), 6.67 (d, *J* = 7.5 Hz, 1H), 4.34 (s, 1H), 1.53 (d, *J* =16.0 Hz, 9H), 1.21 (d, *J* = 16.0 Hz, 9H), 1.17 (s, 9H); **^{13}C NMR** (125 MHz, $CDCl_3$) δ 146.5 (d, *J* = 7.9 Hz), 135.8, 134.6 (d, *J* = 2.4 Hz), 134.3, 133.6 (d, *J* = 7.6 Hz), 131.1 (d, *J* = 2.2 Hz), 130.6, 129.7, 128.6, 128.3, 127.2, 127.0, 126.7 (d, *J* = 7.2 Hz), 126.6, 125.2, 124.1, 56.0, 55.4 (d, *J* = 13.7 Hz), 38.6 (d, *J* = 24.3 Hz), 38.4 (d, *J* = 23.7 Hz), 31.2 (d, *J* = 6.6 Hz), 30.7 (d, *J* = 6.4 Hz), 22.6; **^{31}P NMR** (200 MHz, $CDCl_3$) δ 54.33; **HRMS** (ESI) calculated for [$C_{29}H_{40}$AuClNNaOPS] $[M+Na]^+$: 736.1815, found: 736.1800; **$[\alpha]_D^{20}$** = 81.1 (c = 0.2, $CHCl_3$).

6.2 (di-*tert*-butyl(2-((*S*)-(((*R*)-*tert*-butylsulfinyl)amino)(phenyl)methyl)phenyl)chloro-λ^5-phosphaneyl)gold. ((*Sc*,*Rs*)-Au(TY2)Cl)

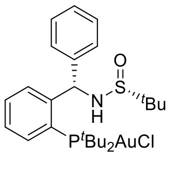

Isolated (*Sc*,*Rs*)-**Au(TY2)Cl** in 49% three steps total yield as white solid.

^{1}H NMR (500 MHz, $CDCl_3$) δ 8.04 – 7.99 (m, 2H), 7.85 (t, *J* = 7.5 Hz, 1H), 7.69 (t, *J* = 7.5 Hz, 1H), 7.49 (t, *J* = 7.5 Hz, 1H), 7.41 – 7.33 (m, 4H), 7.31 – 7.28 (m, 1H), 3.98 (s, 1H), 1.53 (d, *J* = 16.0 Hz, 9H), 1.30 (d, *J* = 16.0 Hz, 9H), 1.25 (s, 9H); **^{13}C NMR** (125 MHz, $CDCl_3$) δ 146.61 (d, *J* = 8.0 Hz), 140.64, 134.38 (d, *J* = 2.6 Hz), 132.0 (d,

J = 7.6 Hz), 131.26 (d, J = 2.1 Hz), 129.38, 128.74, 128.13, 126.60 (d, J = 7.4 Hz), 125.8 (d, J = 41 Hz), 58.6 (d, J = 13.5 Hz), 56.10, 38.6 (d, J = 50 Hz), 38.56, 31.2 (d, J = 6.5 Hz), 30.7 (d, J = 6.5 Hz), 22.65; **^{31}P NMR** (200 MHz, $CDCl_3$) δ 54.51; **HRMS** (ESI) calculated for [$C_{25}H_{38}AuClNNaOPS$] $[M+Na]^+$: 686.1658, found: 686.1665; **$[\alpha]_D^{20}$** = -33.5 (c = 0.2, $CHCl_3$).

6.3 (di-*tert*-butyl(2-((*S*)-(((*R*)-*tert*-butylsulfinyl)amino)(3,5-di-tert-butyl-4-methoxyphenyl)methyl)phenyl)chloro-λ^5-phosphaneyl)gold ((*Sc*,*Rs*)-Au(TY3)Cl)

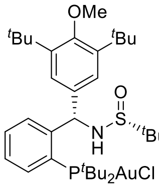

Isolated (*Sc*,*Rs*)-**Au(TY3)Cl** in 43% three steps total yield as white solid.

^{1}H NMR (500 MHz, $CDCl_3$) δ 7.95 – 7.89 (m, 2H), 7.80 (t, J = 8.0 Hz, 1H), 7.63 (t, J = 7.5 Hz, 1H), 7.43 (t, J = 7.5 Hz, 1H), 7.16 (s, 2H), 3.99 (s, 1H), 3.64 (s, 3H), 1.48 (d, J = 15.5 Hz, 9H), 1.34 (s, 18H), 1.28 (d, J = 15.5 Hz, 9H), 1.21 (s, 9H); **^{13}C NMR** (125 MHz, $CDCl_3$) δ 159.2, 147.7 (d, J = 8.3 Hz), 143.8, 135.1, 134.2 (d, J = 2.6 Hz), 132.0 (d, J = 7.6 Hz), 131.1 (d, J = 2.1 Hz), 127.6, 126.5 (d, J = 7.4 Hz), 125.8 (d, J = 41.5 Hz), 64.3, 59.0 (d, J = 13.7 Hz), 56.1, 38.6 (d, J = 21.4 Hz), 38.4 (d, J = 21.0 Hz), 36.0, 32.0, 31.4 (d, J = 6.6 Hz), 30.9 (d, J = 6.5 Hz), 22.7; **^{31}P NMR** (200 MHz, $CDCl_3$) δ 54.43; **HRMS** (ESI) calculated for [$C_{34}H_{56}AuClNNaO_2PS$] $[M+Na]^+$: 828.3016, found: 828.3009; **$[\alpha]_D^{20}$** = -43.1 (c = 0.2, $CHCl_3$).

6.4 (di-*tert*-butyl(2-((*S*)-(((*R*)-*tert*-butylsulfinyl)amino)(4-methoxyphenyl)methyl)phenyl)chloro-λ^5-phosphaneyl)gold ((*Sc*,*Rs*)-Au(TY4)Cl)

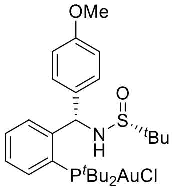

Isolated (*Sc*,*Rs*)-**Au(TY4)Cl** in 49% three steps total yield as white solid.

^{1}H NMR (500 MHz, $CDCl_3$) δ 8.00- 7.97 (m, 1H), 7.91 (s, 1H), 7.80 (t, J = 8.0 Hz, 1H), 7.64 (t, J = 7.5 Hz, 1H), 7.43 (t, J = 7.5 Hz, 1H), 7.27 (s, 1H), 7.25 (s, 1H), 6.82 (d, J = 8.5 Hz, 2H), 3.76 (s, 3H), 3.74 (s, 1H), 1.47 (d, J = 16.0 Hz, 9H), 1.25 (d, J = 16.0 Hz, 9H), 1.19 (s, 9H); **^{13}C NMR** (125 MHz, $CDCl_3$) δ 159.2, 146.9 (d, J = 8.0 Hz), 134.4 (d, J = 2.6 Hz), 132.7,131.8 (d, J = 7.5 Hz), 131.2 (d, J = 2.2 Hz), 130.7, 126.5 (d, J = 7.4 Hz), 125.7 (d, J = 41.3 Hz), 114.0, 58.1 (d, J = 13.7 Hz), 55.9, 55.2, 38.6 (d, J = 25.6 Hz), 38.4 (d, J = 25.3 Hz), 31.3 (d, J = 6.7 Hz), 30.7 (d, J = 6.5 Hz), 22.6; **^{31}P NMR** (200 MHz, $CDCl_3$) δ 54.45; **HRMS** (ESI) calculated for [$C_{26}H_{40}AuClNNaO_2PS$] $[M+Na]^+$: 716.1764, found: 716.1766; **$[\alpha]_D^{20}$** = -32.8 (c = 0.2, $CHCl_3$).

6.5 (di-*tert*-butyl(3-((*S*)-(((*R*)-*tert*-butylsulfinyl)amino)(naphthalen-1-yl)methyl)-5,5,8,8-tetra-methyl-5,6,7,8-tetrahydronaphthalen-2-yl)chloro-λ^5-phosphaneyl)gold((*Sc*,*Rs*)-Au(TY5)Cl)

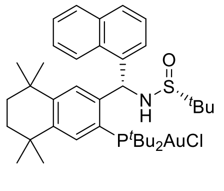

Isolated (*Sc*,*Rs*)-**Au(TY5)Cl** in 47% three steps total yield as white solid.

^{1}H NMR (500 MHz, $CDCl_3$) δ 8.74 (d, J = 9.0 Hz, 1H), 8.27 (d, J = 3.0 Hz, 1H), 8.04 (d, J = 5.0 Hz, 1H), 7.88 – 7.75 (m, 4H), 7.56 (t, J = 7.5 Hz, 1H), 7.21 (t, J = 7.5 Hz, 1H), 6.62 (d, J = 6.0 Hz, 1H), 4.34 (s, 1H), 1.83 – 1.76 (m, 4H), 1.52 (d, J = 16.0 Hz, 9H), 1.39 (s, 3H), 1.36 (d, J = 3.0 Hz, 6H), 1.32 (s, 3H), 1.20 – 1.17 (m, 18H); **^{13}C NMR** (125 MHz, $CDCl_3$) δ 148.5 (d, J = 2.3 Hz), 143.5 (d, J = 6.9 Hz), 142.3 (d, J = 8.4 Hz), 136.4, 134.3, 133.6 (d, J = 2.7 Hz), 131.7 (d, J = 8.0 Hz), 130.7, 129.5, 128.6, 128.3, 127.1, 126.6, 125.4, 124.2, 123.3 (d, J = 42.5 Hz), 55.9, 55.1 (d, J = 13.0 Hz), 38.6 (d, J = 12.0 Hz), 38.3 (d, J = 11.5 Hz), 34.8, 34.6, 34.3, 31.9, 31.8 (d, J = 6.0 Hz), 31.3 (d, J = 6.5 Hz), 31.2, 30.9 (d, J = 6.3 Hz), 22.8; **^{31}P NMR** (200 MHz, $CDCl_3$) δ 53.58; **HRMS** (ESI) calculated for [$C_{37}H_{54}AuClNNaOPS$] $[M+Na]^+$: 846.2910, found: 846.2897; **$[\alpha]_D^{20}$** = 91.7 (c = 0.2, $CHCl_3$).

6.6 ((3-((*S*)-anthracen-9-yl(((*R*)-*tert*-butylsulfinyl)amino)methyl)-5,5,8,8-tetramethyl-5,6,7,8-tetrahydronaphthalen-2-yl)di-*tert*-butylchloro-λ^5-phosphaneyl)gold ((*Sc*,*Rs*)-Au(TY6)Cl)

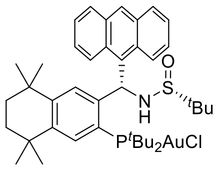

Isolated (*Sc*,*Rs*)-**PC6** in 45% three steps total yield as white solid.
^{1}H NMR (500 MHz, $CDCl_3$) δ 8.55 (s, 1H), 8.47 (d, *J* = 5.0 Hz, 1H), 8.24 – 7.88 (m, 5H), 7.82 (d, *J* = 9.5 Hz, 1H), 7.47 – 7.29 (m, 4H), 4.54 (s, 1H), 1.86 – 1.80 (m, 4H), 1.48 (d, *J* = 15.5 Hz, 9H), 1.40 (d, *J* = 28.5 Hz, 6H), 1.33 (d, *J* = 26.5 Hz, 6H), 1.21 (s, 9H), 1.15 (d, *J* = 15.5 Hz, 9H); **^{13}C NMR** (125 MHz, $CDCl_3$) **^{13}C NMR** (125 MHz, $CDCl_3$) δ 148.5 (d, *J* = 2.4 Hz), 143. 8 (d, *J* = 6.6 Hz), 143.2 (d, *J* = 8.5 Hz), 134.5 (d, *J* = 2.5 Hz), 132.3, 132.2 (d, *J* = 1.6 Hz), 130.8, 130.1, 129.9, 126.5, 125.1, 124.6, 124.4, 124.0, 56.2, 55.7 (d, *J* = 10.4 Hz), 39.0 (d, *J* = 26.3 Hz), 38.2 (d, *J* = 25.9 Hz), 34.8 (d, *J* = 5.3 Hz), 34.6, 34.3, 32.1, 31.9 (d, *J* = 6.5 Hz), 31.7 (d, *J* = 10.4 Hz), 31.0, 30.2 (d, *J* = 6.1 Hz), 22.8; **^{31}P NMR** (200 MHz, $CDCl_3$) δ 54.54; **HRMS** (ESI) calculated for [$C_{29}H_{40}AuClNNaOPS$] $[M+Na]^+$: 896.3066, found: 896.3065; **$[\alpha]_D^{20}$** = 133.6 (c = 0.2, $CHCl_3$).

7. Preparation and Characterization of ortho-alkynylaryl ferrocenes derivatives 1, 3[1] and 4[2].

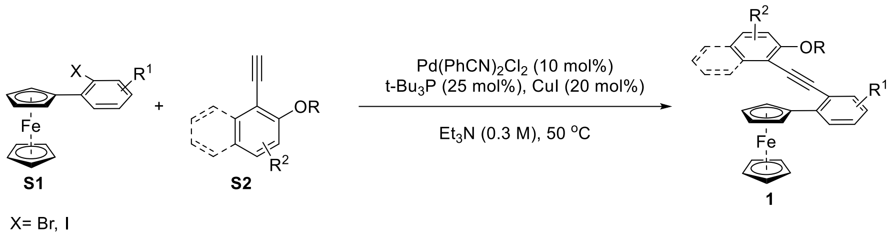

$Pd(PhCN)_2Cl_2$ (10 mol%, 38.4 mg, cas: 14220-64-5, *Laajoo*), CuI (10 mol%, 38 mg, *Energy Chemical*) the substituted 2-ferrocenyl-1-halobenzene **S1** (1 mmol, cas: 97494-36-5 or 97737-44-5) were transferred to a Schlenk tube under a N_2 atmosphere. In a separate flask, Et_3N (0.3 M, *Adamas-beta*) was sparged with N_2 for 30 min, after which it was transferred into the Schlenk tube. *t*-Bu_3P (25 mol%, 0.25 mL, 1 M in toluene, *Macklin*) was then added to the solution, stirred at room temperature for 10 min, then the substituted arylalkyne **S2**[3] (1.5 equiv) were added, the reaction mixture was stirred at 50 °C for 12 h, during which the reaction progress was monitored by TLC. After **S1** was completely, quenched by saturated aqueous solution of NH_4Cl, and then extracted with EA. The extracts were washed by saturated salt water and dried over anhydrous Na_2SO_4, then the solvent was removed under reduced pressure, the crude product was purified by flash column chromatography on silica gel (Hexane: DCM= 10:1 to 5:1) to give product **1** as orange-yellow solid.

7.1 1-((2-ferrocenephenyl)ethynyl)-2-methoxynaphthalene (1aa)

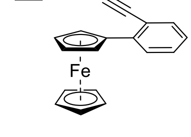

Isolated **1aa** in 83% yield as orange-yellow solid.
^{1}H NMR (500 MHz, $CDCl_3$) δ 8.33 (d, *J* = 8.5 Hz, 1H), 7.84-7.78 (m, 2H), 7.71 – 7.63 (m, 2H), 7.54 – 7.51 (m, 1H), 7.38 (t, *J* = 7.4 Hz, 1H), 7.31 – 7.25 (m, 2H), 7.25 – 7.20 (m, 1H), 5.23 – 5.18 (m, 2H), 4.36 – 4.31 (m, 2H), 4.09 (s, 5H), 4.07 (s, 3H); **^{13}C NMR** (125 MHz, $CDCl_3$) δ 159.0, 140.4, 134.5, 134.1, 130.0, 128.8, 128.6, 128.0, 127.9, 127.2, 125.6, 125.5, 124.2, 121.2, 112.7, 107.0, 99.8, 88.1, 84.8, 69.7, 69.2, 68.6, 56.4; **HRMS** (ESI) calculated for [$C_{29}H_{22}FeNaO$] $[M+Na]^+$: 465.0913, found: 465.0922; **IR** (KBr, cm^{-1}) 3082, 1603, 1508, 1251, 1084, 810, 760, 495.

7.2 1-((2-ferrocenephenyl)ethynyl)-2-ethoxynaphthalene (1ab)

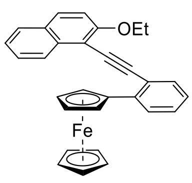

Isolated **1ab** in 79% yield as white solid.

^{1}H NMR (500 MHz, $CDCl_3$) δ 8.34 (d, *J* = 8.5 Hz, 1H), 7.79 (m, 2H), 7.71 – 7.63 (m, 2H), 7.53 – 7.50 (m, 1H), 7.39 – 7.36 (m, 1H), 7.30 – 7.25 (m, 2H), 7.23 – 7.20 (m, 1H), 5.23 – 5.18 (m, 2H), 4.35 – 4.29 (m, 4H), 4.09 (s, 5H), 1.57 (t, *J* = 7.0 Hz, 3H); **^{13}C NMR** (125 MHz, $CDCl_3$) δ 158.4, 140.3, 134.6, 134.2, 129.9, 128.8, 128.6, 128.0, 127.9, 127.2, 125.6, 125.5, 124.1, 121.4, 114.0, 107.4, 99.6, 88.4, 84.8, 69.7, 69.2, 68.7, 65.1, 15.2; **HRMS** (ESI) calculated for [$C_{30}H_{24}FeNaO$] $[M+Na]^+$: 479.1069, found: 479.1080; **IR** (KBr, cm^{-1}) 2926, 1620, 1589, 1261, 1070, 816, 744, 501.

7.3 1-((2-ferrocenephenyl)ethynyl)-2-propoxynaphthalene (1ac)

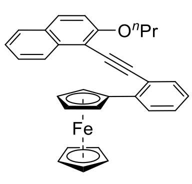

Isolated **1ac** in 81% yield as orange-yellow solid.

^{1}H NMR (500 MHz, $CDCl_3$) δ 8.32 (d, *J* = 8.5 Hz, 1H), 7.78 (t, *J* = 9.5 Hz, 2H), 7.66 (d, *J* = 8.0 Hz, 2H), 7.50 (t, *J* = 7.5 Hz, 1H), 7.37 (t, *J* = 7.5 Hz, 1H), 7.29 – 7.26 (m, 2H), 7.24 – 7.20 (m, 1H), 5.18 (s, 2H), 4.33 (s, 2H), 4.20 (t, *J* = 6.5 Hz, 2H), 4.09 (s, 5H), 2.00 – 1.90 (m, 2H), 1.13 (t, *J* = 7.5 Hz, 3H); **^{13}C NMR** (125 MHz, $CDCl_3$) δ 158.5, 140.3, 134.6, 134.1, 129.9, 128.9, 128.6, 128.0, 127.8, 127.1, 125.6, 125.6, 124.1, 121.4, 114.1, 107.3, 99.5, 88.3, 84.9, 71.1, 69.7, 69.3, 68.7, 22.9, 10.6; **HRMS** (ESI) calculated for [$C_{31}H_{26}FeNaO$] $[M+Na]^+$: 493.1226, found: 493.1236; **IR** (KBr, cm^{-1}) 2964, 1589, 1508, 1273, 1074, 810, 763, 496.

7.4 1-((2-ferrocenephenyl)ethynyl)-2-butoxynaphthalene (1ad)

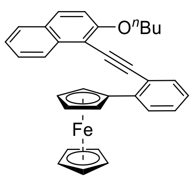

Isolated **1ad** in 76% yield as orange-yellow solid.

^{1}H NMR (500 MHz, $CDCl_3$) δ 8.39 (d, *J* = 8.5 Hz, 1H), 7.85 (t, *J* = 9.5 Hz, 2H), 7.76 – 7.70 (m, 2H), 7.57 (t, *J* = 7.0 Hz, 1H), 7.43 (t, *J* = 7.0 Hz, 1H), 7.37 – 7.32 (m, 2H), 7.28 (dd, *J* = 11.0, 4.0 Hz, 1H), 5.24 (d, *J* = 1.5 Hz, 2H), 4.40 (d, *J* = 1.5 Hz, 2H), 4.31 (t, *J* = 6.5 Hz, 2H), 4.15 (s, 5H), 2.03 – 1.95 (m, 2H), 1.67 (dt, *J* = 15.0, 7.5 Hz, 2H), 1.08 (t, *J* = 7.5 Hz, 3H); **^{13}C NMR** (125 MHz, $CDCl_3$) δ 158.6, 140.3, 134.6, 134.1, 129.8, 128.9, 128.5, 128.0, 127.8, 127.1, 125.6, 125.5, 124.1, 121.5, 114.1, 107.3, 99.5, 88.3, 84.9, 69.7, 69.29, 69.27, 68.7, 31.6, 19.3, 14.0; **HRMS** (ESI) calculated for [$C_{32}H_{28}FeNaO$] $[M+Na]^+$: 507.1382, found: 507.1384; **IR** (KBr, cm^{-1}) 2965, 1586, 1510, 1275, 1079, 812, 750, 501.

7.5 1-((2-ferrocenephenyl)ethynyl)-2-isopropoxynaphthalene (1ae)

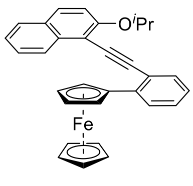

Isolated **1ae** in 71% yield as orange-yellow solid.

^{1}H NMR (500 MHz, $CDCl_3$) δ 8.33 (d, *J* = 8.4 Hz, 1H), 7.78 – 7.77 (m, 2H), 7.67 – 7.65 (m, 2H), 7.50 (t, *J* = 7.7 Hz, 1H), 7.39 – 7.36 (m, 1H), 7.29 – 7.20 (m, 3H), 5.21 (s, 2H), 4.86 – 4.81 (m, 1H), 4.35 (s, 2H), 4.10 (s, 5H), 1.47 (d, *J* = 6.0 Hz, 6H); **^{13}C NMR** (125 MHz, $CDCl_3$) δ 157.6, 140.3, 134.8, 134.1, 129.7, 128.9, 128.7, 127.9, 127.8, 127.1, 125.7, 125.6, 124.3, 121. 5, 116.3, 108.9, 99.4, 88.6, 85.0, 72.2, 69.7, 69.3, 68.8, 22.5; **HRMS** (ESI) calculated for [$C_{31}H_{26}FeNaO$] $[M+Na]^+$: 493.1226, found: 493.1222; **IR** (KBr, cm^{-1}) 2976, 1576, 1500, 1271, 1105, 1001, 810, 760, 495.

7.6 1-((2-ferrocenephenyl)ethynyl)-2-(naphthalen-2-ylmethoxy)naphthalene (1af)

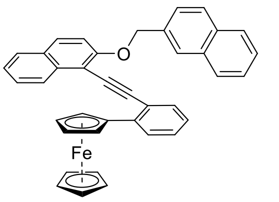

Isolated **1af** in 92% yield as orange-yellow solid.

^{1}H NMR (400 MHz, $CDCl_3$) δ 8.35 (d, *J* = 8.4 Hz, 1H), 7.99 (s, 1H), 7.86 – 782 (m, 2H), 7.80 – 7.70 (m, 3H), 7.67 – 7.62 (m, 3H), 7.53 – 7.49 (m, 1H), 7.47 – 7.45 (m, 2H), 7.37 (t, *J* = 7.2 Hz, 1H), 7.33 – 7.25 (m, 2H), 7.17 (t, *J* = 7.2 Hz, 1H), 5.50 (s, 2H), 5.12 (s, 2H), 4.17 (s, 2H), 4.01 (s, 5H); **^{13}C NMR** (100 MHz, $CDCl_3$) δ 158.3, 140.6, 134.7, 134.6, 134.1, 133.4, 133.1, 129.9, 129.1, 128.9, 128.5, 128.1, 128.1, 128.0, 127.8, 127.3, 126.3, 126.1, 125.8, 125.6, 125.1, 124.5, 121.5, 114.8, 108.2, 100.0, 88.2, 85.0, 71.6, 69.7, 69.5, 68.7; **HRMS** (ESI) calculated for [$C_{38}H_{39}NO_2PS$] $[M+H]^+$: 604.2434, found: 604.2434; **IR** (KBr, cm^{-1}) 3057, 1589, 1508, 1265, 1080, 815, 748, 498.

7.7 2-(benzyloxy)-1-((2-ferrocenephenyl)ethynyl)naphthalene (1ag)

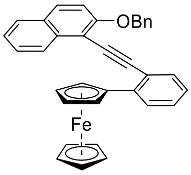

Isolated **1ag** in 89% yield as orange-yellow solid.

^{1}H NMR (500 MHz, $CDCl_3$) δ 8.45 – 8.32 (m, 1H), 7.94 – 7.74 (m, 3H), 7.68– 7.63 (m, 2H), 7.55– 7.50 (m, 3H), 7.45 – 7.35 (m, 3H), 7.34 – 7.20 (m, 3H), 5.39 (s, 2H), 5.14 (s, 2H), 4.23 (s, 2H), 4.07 (s, 5H); **^{13}C NMR** (125 MHz, $CDCl_3$) δ 158.1, 140.5, 137.1, 134.6, 134.1, 129.8, 129.0, 128.8, 128.6, 128.0, 127.9, 127.9, 127.2, 125.7, 125.6, 124.4, 121.4, 114.6, 108.0, 99.9, 88.1, 84.9, 71.3, 69.7, 69.4, 68.6; **HRMS** (ESI) calculated for [$C_{35}H_{26}FeNaO$] $[M+Na]^+$: 541.1226, found: 541.1232; **IR** (KBr, cm^{-1}) 3061, 1589, 1508, 1267, 1068, 808, 749, 495.

7.8 1-((2-ferrocenephenyl)ethynyl)-2-(cyclopropylmethoxy)naphthalene (1ah)

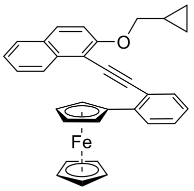

Isolated **1ah** in 82% yield as orange-yellow solid.

^{1}H NMR (400 MHz, $CDCl_3$) δ 8.33 (d, *J* = 8.4 Hz, 1H), 7.80 – 7.76 (m, 2H), 7.71 – 7.65 (m, 2H), 7.54 – 7.47 (m, 1H), 7.41 – 7.35 (m, 1H), 7.33 – 7.24 (m, 2H), 7.24 – 7.19 (m, 1H), 5.21 – 5.16 (m, 2H), 4.34 – 4.29 (m, 2H), 4.10 (d, *J* = (7.0 Hz, 2H), 4.09 (s, 5H), 1.45 – 1.36 (m, 1H), 0.71 – 0.63 (m, 2H), 0.41 – 0.46 (m, 2H); **^{13}C NMR** (100 MHz, $CDCl_3$) δ 158.6, 140.4, 134.6, 134.1, 129.8, 128.9, 128.7, 128.0, 127.9, 127.1, 125.7, 125.6, 124.2, 121.5 115.0, 107.9, 99.6, 88.4, 85.0, 74.5, 69.7, 69.3, 68.7, 10.7, 3.4; **HRMS** (ESI) calculated for [$C_{32}H_{26}FeNaO$] $[M+Na]^+$: 505.1226, found: 505.1230; **IR** (KBr, cm^{-1}) 3057, 1589, 1500, 1265, 1069, 806, 749, 490.

7.9 1-((2-ferrocenephenyl)ethynyl)-2-(cyclobutylmethoxy)naphthalene (1ai)

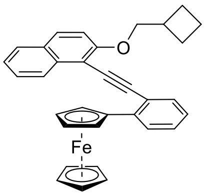

Isolated **1ai** in 71% yield as orange-yellow solid.

^{1}H NMR (400 MHz, $CDCl_3$) δ 8.31 (d, *J* = 8.4 Hz, 1H), 7.77 (t, *J* = 7.6 Hz, 2H), 7.66 (t, *J* = 8.0 Hz, 2H), 7.49 (t, *J* = 7.6 Hz, 1H), 7.36 (t, *J* = 7.4 Hz, 1H), 7.29 (d, *J* = 7.4 Hz, 1H), 7.25 – 7.19 (m, 2H), 5.15 – 5.14 (m, 2H), 4.32 – 4.31 (m, 2H), 4.10 (d, *J* = 7.0 Hz, 2H), 4.08 (s, 5H), 2.60 – 2.44 (m, 1H), 1.97– 1.90 (m, 1H), 2H), 1.67 – 1.60 (m, 2H), 1.49 – 1.45 (m, 2H); **^{13}C NMR** (100 MHz, $CDCl_3$) δ 158.8, 140.4, 134.7, 134.1, 129.9, 129.0, 128.6, 128.0, 127.8, 127.1, 125.7, 125.6, 124.1, 121.6, 114.6, 114.4, 107.5, 99.5, 88.4, 85.1, 73.9, 69.7, 69.4, 68.7, 39.4, 29.6, 25.6; **HRMS** (ESI) calculated for [$C_{33}H_{28}FeNaO$] $[M+Na]^+$: 519.1382, found: 519.1391; **IR** (KBr, cm^{-1}) 2951, 1589, 1456, 1271, 1070, 808, 746, 509.

7.10 1-((2-ferrocenephenyl)ethynyl)-2-(cyclopentylmethoxy)naphthalene (1aj)

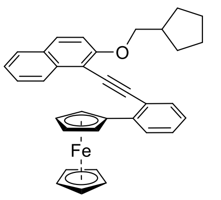

Isolated **1aj** in 73% yield as orange-yellow solid.

^{1}H NMR (400 MHz, $CDCl_3$) δ 8.33 (d, *J* = 8.4 Hz, 1H), 7.80 (t, *J* = 7.8 Hz, 2H), 7.72 – 7.65 (m, 2H), 7.53 – 7.49 (m, 1H), 7.41 – 7.36 (m, 1H), 7.33 – 7.28 (m, 2H), 7.26 – 7.22 (m, 1H), 5.19 – 5.16 (m, 2H), 4.36 – 4.33 (m, 2H), 4.13 (d, *J* = 7.0 Hz, 2H), 4.11 (s, 5H), 2.58 – 2.50 (m, 1H), 1.98 – 1.89 (m, 2H), 1.69 – 1.61 (m, 4H), 1.53 – 1.48 (m, 2H); **^{13}C NMR** (100 MHz, $CDCl_3$) δ 158.8, 140.4, 134.7, 134.1, 129.8, 129.0, 128.6, 128.0, 127.8, 127.1, 125.7, 125.6, 124.1, 121.6, 114.4, 107.5, 99.5, 88.4, 85.1, 73.9, 69.7, 69.4, 68.7, 39.4, 29.6, 25.5; **HRMS** (ESI) calculated for [$C_{34}H_{30}FeNaO$] [M+Na]$^+$: 533.1539, found: 533.1540; **IR** (KBr, cm^{-1}) 2951, 1589, 1508, 1261, 1070, 808, 747, 509.

7.11 1-((2-ferrocenephenyl)ethynyl)-2-(2-ethylbutoxy)naphthalene (1ak)

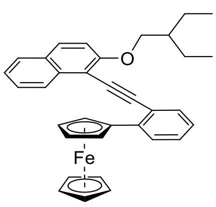

Isolated **1ak** in 73% yield as orange-yellow solid.

^{1}H NMR (400 MHz, $CDCl_3$) δ 8.31 (d, *J* = 8.4 Hz, 1H), 7.80 (t, *J* = 9.0 Hz, 2H), 7.71 (dd, *J* = 8.0, 1.2Hz, 1H), 7.65 (dd, *J* = 7.6, 1.2 Hz, 1H), 7.52 – 7.46 (m, 1H), 7.41 – 7.35 (m, 1H), 7.34 – 7.27 (m, 2H), 7.25 – 7.21 (m, 1H), 5.15 – 5.12 (m, 2H), 4.36 – 4.33 (m, 2H), 4.14 (d, *J* = 6.0 Hz, 2H), 4.10 (s, 5H), 1.88 – 1.81 (m, 1H), 1.67 – 1.54 (m, 4H), 0.99 (t, *J* = 7.2 Hz, 6H); **^{13}C NMR** (100 MHz, $CDCl_3$) δ 158.9, 140.4, 134.6, 133.9, 129.8, 129.1, 128.5, 128.0, 127.8, 127.1, 125.7, 125.6, 124.1, 121.7, 114.2, 107.4, 99.4, 88.2, 85.2, 71.8, 69.7, 69. 5, 68.6, 41.1, 23.3, 11.2; **HRMS** (ESI) calculated for [$C_{34}H_{32}FeNaO$] [M+Na]$^+$: 535.1695, found: 535.1699; **IR** (KBr, cm^{-1}) 2960, 1589, 1508, 1273, 1070, 808, 750, 500.

7.12 1-((2-ferrocenephenyl)ethynyl)-2-(cyclohexylmethoxy)naphthalene (1al)

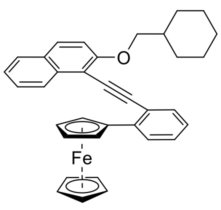

Isolated **1al** in 85% yield as orange-yellow solid.

^{1}H NMR (400 MHz, $CDCl_3$) δ 8.31 (d, *J* = 8.4 Hz, 1H), 7.78 – 7.75 (m, 2H), 7.71 – 7.62 (m, 2H), 7.50– 7.46 (m, 1H), 7.40 – 7.32 (m, 1H), 7.31 – 7.25 (m, 1H), 7.25 – 7.14 (m, 2H), 5.17 – 5.09 (m, 2H), 4.36 – 4.28 (m, 2H), 4.08 (s, 5H), 4.02 (d, *J* = 6.0 Hz, 2H), 2.02 – 1.68 (m, 6H), 1.34 – 1.11 (m, 5H); **^{13}C NMR** (100 MHz, $CDCl_3$) δ 158.8, 140.4, 134.6, 134.0, 129.8, 129.0, 128.5, 128.0, 127.8, 127.1, 125.7, 125.6, 124.1, 121.6, 114.2, 107.3, 99.5, 88.3, 85.1, 75.0, 69.7, 69.4, 68.6, 37.9, 29.9, 26.5, 25.8; **HRMS** (ESI) calculated for [$C_{35}H_{30}FeNaO$] [M+Na]$^+$: 547.1695, found: 547.1701; **IR** (KBr, cm^{-1}) 2963, 1590, 1508, 1270, 1065, 808, 754, 500.

7.13 1-((2-ferrocene-5-fluorophenyl)ethynyl)-2-(cyclohexylmethoxy)naphthalene (1am)

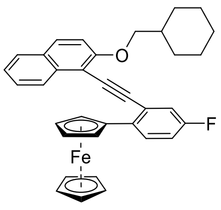

Isolated **1am** in 76% yield as orange-yellow solid.

^{1}H NMR (400 MHz, $CDCl_3$) δ 8.25 (d, *J* = 8.4 Hz, 1H), 7.80 – 7.75 (m , 2H), 7.63 – 7.61 (m, 1H), 7.49 – 7.47 (m, 1H), 7.37 – 7.30 (m, 2H), 7.26 – 7.23 (m, 1H), 7.03 – 6.97 (m, 1H), 5.06 (t, *J* = 1.6 Hz, 2H), 4.32 (t, *J* = 1.6 Hz, 2H), 4.08 (s, 5H), 4.02 (d, *J* = 6.2 Hz, 2H), 1.97– 1.94 (m, 3H), 1.81 – 1.70 (m, 3H), 1.39 – 1.22 (m, 5H); **^{13}C NMR** (100 MHz, $CDCl_3$) δ 160.4 (d, *J* = 245.5 Hz), 159.1, 136.5 (d, *J* = 3.1 Hz), 134.5, 130.6 (d, *J* = 8.4 Hz), 130.2, 128.5, 128.0, 127.2, 125.5, 124.1, 123.1 (d, *J* = 9.3 Hz), 119.9 (d, *J* = 2.2 Hz), 115.3 (d, *J* = 21.4 Hz), 114.1, 106.8, 98.2 (d, *J* = 2.9 Hz), 89.3, 84.5, 75.0, 69.8, 69.6, 69.3, 68.6, 38.0, 29.9, 26.5, 25.8; **HRMS** (ESI) calculated for [$C_{35}H_{31}FFeNaO$] [M+Na]$^+$: 565.1601, found: 565.1600; **IR** (KBr, cm^{-1}) 2965, 2450, 1510, 1270, 1065, 810, 750, 500.

7.14 1-((2-ferrocene-5-chlorophenyl)ethynyl)-2-(cyclohexylmethoxy)naphthalene (1an)

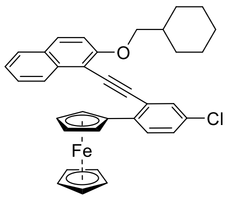

Isolated **1an** in 75% yield as orange-yellow solid.

^{1}H NMR (400 MHz, $CDCl_3$) δ 8.27 (d, *J* = 8.4 Hz, 1H), 7.81 – 7.76 (m, 2H), 7.61 – 7.58 (m , 2H), 7.53 – 7.47 (m, 1H), 7.37 (t, *J* = 6.8 Hz, 1H), 7.25 – 7.21 (m, 2H), 5.10 (t, *J* = 2.0 Hz, 2H), 4.35(t, *J* = 2.0 Hz,, 2H), 4.08 (s, 5H), 4.03 (d, *J* = 6.1 Hz, 2H), 2.00– 1.95 (m, 3H), 1.81 – 1.70 (m, 3H), 1.28 – 1.07 (m, 5H); **^{13}C NMR** (100 MHz, $CDCl_3$) δ 159.1, 139.1, 134.5, 133.3, 130.9, 130.2, 130.2, 128.5, 128.1, 127.9, 127.3, 125.5, 124.1, 123.0, 114.0, 106.7, 98.1, 89.6, 84.0, 75.0, 69.8, 69.7, 69.3, 68.9, 38.0, 30.0, 26.5, 25.8; **HRMS** (ESI) calculated for [$C_{35}H_{31}ClFeNaO$] $[M+Na]^+$: 581.1306, found: 581.1300; **IR** (KBr, cm^{-1}) 2924, 1589, 1499, 1266, 1082, 809, 750, 500.

7.15 1-((5-bromo-2-ferrocenephenyl)ethynyl)-2-(cyclohexylmethoxy)naphthalene (1ao)

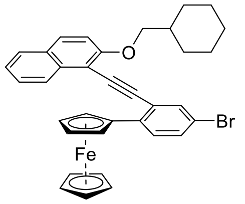

Isolated **1ao** in 78% yield as orange-yellow solid.

^{1}H NMR (400 MHz, $CDCl_3$) δ 8.27 (d, *J* = 8.4 Hz, 1H), 7.83 – 7.78 (m, 2H), 7.76 (d, *J* = 2.4 Hz, 1H), 7.55 – 7.49 (m, 2H), 7.40 – 7.34 (m, 2H), 7.24 (d, *J* = 2.8 Hz, 1H), 5.12 – 5.09 (m, 2H), 4.37 – 4.34 (m, 2H), 4.08 (s, 5H), 4.03 (d, *J* = 6.0 Hz, 2H), 2.04 – 1.94 (m, 3H), 1.83 – 1.70 (m, 3H), 1.27 (ddd, *J* = 20.1, 15.6, 3.1 Hz, 5H); **^{13}C NMR** (100 MHz, $CDCl_3$) δ 159.1, 139.6, 136.2, 134.5, 130.8, 130.4, 130.3, 128.5, 128.1, 127.3, 125.5, 124.1, 123.4, 118.6, 114.0, 106.7, 98.0, 89.7, 83.9, 75.0, 69.8, 69.8, 69.3, 68.9, 38.0, 30.0, 26.6, 25.9; **HRMS** (ESI) calculated for [$C_{35}H_{31}BrFeNaO$] $[M+Na]^+$: 625.0802, found: 625.0804; **IR** (KBr, cm^{-1}) 2925, 1560, 1510, 1050, 1260, 812, 750, 500.

7.16 1-((2-ferrocene-4-fluorophenyl)ethynyl)-2-(cyclohexylmethoxy)naphthalene (1ap)

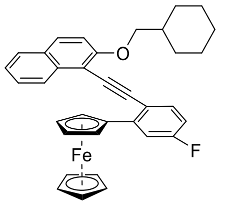

Isolated **1ap** in 82% yield as orange-yellow solid.

^{1}H NMR (400 MHz, $CDCl_3$) δ 8.29 (d, *J* = 8.4 Hz, 1H), 7.78 (t, *J* = 7.8 Hz, 2H), 7.62 – 7.59 (m, 1H), 7.52 – 7.46 (m, 1H), 7.39 – 7.32 (m, 2H), 7.24 (s, 1H), 6.94 – 6.90 (m, 1H), 5.15 (t, *J* = 2.0 Hz, 2H), 4.35 (t, *J* = 2.0 Hz, 2H), 4.10 (s, 5H), 4.03 (d, *J* = 6.2 Hz, 2H), 2.01 – 1.91 (m, 3H), 1.80 – 1.68 (m, 3H), 1.32 – 1.13 (m, 5H); **^{13}C NMR** (100 MHz, $CDCl_3$) δ 162.2 (d, *J* = 247.9 Hz), 158.8, 143.2 (d, *J* = 8.5 Hz), 135.8 (d, *J* = 8.7 Hz), 134.6, 129.9, 128.6, 128.1, 127.2, 125.6, 124.1, 117.7 (d, *J* = 3.0 Hz), 115.3 (d, *J* = 22.7 Hz), 114.2, 113.0 (d, *J* = 22.1 Hz), 107.2, 98.5, 88.0, 83.8, 83.8, 75.1, 69.8, 69.4, 69.1, 38.0, 30.0, 26.6, 25.9; **HRMS** (ESI) calculated for [$C_{35}H_{31}FFeNaO$] $[M+Na]^+$: 565.1601, found: 565.1614; **IR** (KBr, cm^{-1}) 2924, 1589, 1500, 1262, 1018, 810, 748, 500.

7.17 1-((2-ferrocene-4-chlorophenyl)ethynyl)-2-(cyclohexylmethoxy)naphthalene (1aq)

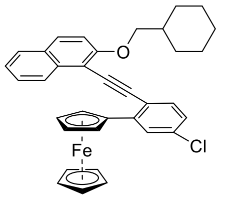

Isolated **1aq** in 80% yield as orange-yellow solid.

^{1}H NMR (400 MHz, $CDCl_3$) δ 8.28 (d, *J* = 8.4 Hz, 1H), 7.78 (t, *J* = 9.0 Hz, 2H), 7.63 (d, *J* = 2.0 Hz, 1H), 7.56 (d, *J* = 8.0 Hz, 1H), 7.52 – 7.47 (m, 1H), 7.39 – 7.33 (m, 1H), 7.24 (d, *J* = 1.1 Hz, 1H), 7.19 – 7.16 (m, 1H), 5.16 – 5.12 (m, 2H), 4.38 – 4.33 (m, 2H), 4.10 (s, 5H), 4.02 (d, *J* = 6.2 Hz, 2H), 2.00 – 1.90 (m, 3H), 1.80 – 1.68 (m, 3H), 1.34 – 1.15 (m, 5H); **^{13}C NMR** (100 MHz, $CDCl_3$) δ 159.0, 142.5, 135.2, 134.6, 133.6, 130.1, 128.7, 128.6, 128.1, 127.2, 125.7, 125.5, 124.2, 120.1, 114.2, 107.0, 98.5, 89.3, 83.7, 75.1, 69.8, 69.7, 69.1, 38.0, 30.0, 26.5, 25.9; **HRMS** (ESI) calculated for [$C_{35}H_{31}ClFeNaO$]

$[M+Na]^+$: 581.1306, found: 581.1306; **IR** (KBr, cm^{-1}) 2924, 1587, 1508, 1274, 1016, 795, 750, 480.

7.18 1-((4-bromo-2-ferrocenephenyl)ethynyl)-2-(cyclohexylmethoxy)naphthalene (1ar)

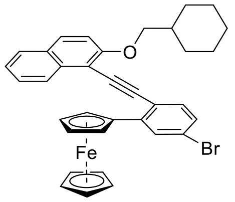

Isolated **1ar** in 86% yield as orange-yellow solid.
^{1}H NMR (400 MHz, $CDCl_3$) δ 8.27 (d, *J* = 8.4 Hz, 1H), 7.83 – 7.75 (m, 3H), 7.52 – 7.49 (m, 2H), 7.38 – 7.36 (m, 1H), 7.35– 7.33 (m, 1H), 7.24 (d, *J* = 2.0 Hz, 1H), 5.15 – 5.12 (m, 2H), 4.38 – 4.34 (m, 2H), 4.10 (s, 5H), 4.03 (d, *J* = 6.4 Hz, 2H), 2.01 – 1.90 (m, 3H), 1.82 – 1.68 (m, 3H), 1.32 – 1.13 (m, 5H); **^{13}C NMR** (100 MHz, $CDCl_3$) δ 159.0, 142.7, 135.3, 134.6, 131.7, 130.1, 128.6, 128.6, 128.1, 127.2, 125.5, 124.2, 121.9, 120.5, 114.2, 107.0, 98.5, 89.5, 83.6, 75.0, 69.8, 69.4, 69.1, 38.0, 30.0, 26.5, 25.9; **HRMS** (ESI) calculated for $[C_{35}H_{31}BrFeNaO]$ [M+Na]+: 625.0802, found: 625.0812; **IR** (KBr, cm^{-1}) 2924, 1587, 1508, 1274, 1016, 810, 763, 500.

7.19 1-((2-ferrocene-3-fluorophenyl)ethynyl)-2-(cyclohexylmethoxy)naphthalene (1as)

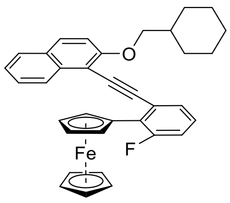

Isolated **1as** in 67% yield as orange-yellow solid.
^{1}H NMR (400 MHz, $CDCl_3$) δ 8.25 (d, *J* = 8.4 Hz, 1H), 7.79 (t, *J* = 9.6 Hz, 2H), 7.52 – 7.45 (m, 2H), 7.39 – 7.37 (m, 1H), 7.25 (d, *J* = 6.8 Hz, 1H), 7.22 – 7.17 (m, 1H), 7.10 – 7.05 (m, 1H), 5.22 – 5.19 (m, 2H), 4.41 – 4.36 (m, 2H), 4.13 (s, 5H), 4.03 (d, *J* = 6.2 Hz, 2H), 2.03 – 1.91 (m, 3H), 1.83 – 1.70 (m, 3H), 1.36 – 1.15 (m, 5H); **^{13}C NMR** (100 MHz, $CDCl_3$) δ 160.4 (d, *J* = 248.2 Hz), 159.1, 134.6, 130.1, 128.5,128.2 (d, *J* = 13.9 Hz), 128.0, 127.2, 126.4 (d, *J* = 9.9 Hz), 125.7, 124.4 (d, *J* = 5.7 Hz), 124.1, 115.6 (d, *J* = 24.2 Hz), 114.2, 107.0, 98.9 (d, *J* = 4.9 Hz), 89.1, 78.2, 75.1, 70.7 (d, *J* = 4.8 Hz), 69.7, 68.5, 38.0, 30.0, 26.6, 25.9; **HRMS** (ESI) calculated for $[C_{35}H_{31}FFeNaO]$ $[M+Na]^+$: 565.1601, found: 565.1605; **IR** (KBr, cm^{-1}) 2924, 1568, 1508, 1275, 1086, 808, 750, 500.

7.20 1-((2-ferrocene-3-chlorophenyl)ethynyl)-2-(cyclohexylmethoxy)naphthalene (1at)

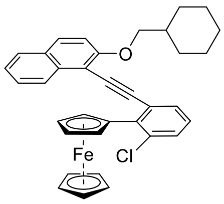

Isolated **1at** in 63% yield as orange-yellow solid.
^{1}H NMR (400 MHz, $CDCl_3$) δ 8.18 (d, *J* = 8.4 Hz, 1H), 7.78 (t, *J* = 9.2 Hz, 2H), 7.62 (dd, *J* = 8.0, 1.6 Hz, 1H), 7.49 – 7.45 (m, 1H), 7.42 – 7.34 (m, 2H), 7.24 (d, *J* = 9.2 Hz, 1H), 7.16 (t, *J* = 8.0 Hz, 1H), 5.24 – 5.19 (m, 2H), 4.44 – 4.40 (m, 2H), 4.18 (s, 5H), 4.02 (d, *J* = 6.2 Hz, 2H), 2.03 – 1.92 (m, 3H), 1.84 – 1.71 (m, 3H), 1.38 – 1.15 (m, 5H); **^{13}C NMR** (100 MHz, $CDCl_3$) δ 159.0, 137.7, 134.5, 133.9, 133.2, 130.7, 130.1, 128.5, 128.0, 127.1, 126.2, 126.1, 125.8, 124.1, 114.1, 107.1, 99.5, 89.8, 82.5, 75.0, 71.7, 69.8, 68.0, 38.1, 30.0, 26.6, 25.9; **HRMS** (ESI) calculated for $[C_{35}H_{31}ClFeNaO]$ $[M+Na]^+$: 581.1306, found: 581.1324; **IR** (KBr, cm^{-1}) 2924, 1589, 1508, 1275, 1085, 808, 750, 500.

7.21 1-((2-ferrocene-4,5-difluorophenyl)ethynyl)-2-(cyclohexylmethoxy)naphthalene (1au)

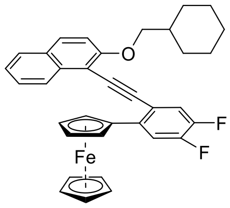

Isolated **1au** in 74% yield as orange-yellow solid.
^{1}H NMR (400 MHz, $CDCl_3$) δ 8.23 (d, *J* = 8.4 Hz, 1H), 7.84 – 7.76 (m, 2H), 7.52 – 7.47 (m, 1H), 7.47 – 7.34 (m, 3H), 7.25 (d, *J* = 8.8 Hz, 1H), 5.07 (t, *J* = 2.0 Hz, 2H), 4.38 – 4.32 (m, 2H), 4.10 (s, 5H), 4.03 (d, *J* = 6.0 Hz, 2H), 2.02 – 1.90 (m, 3H), 1.82 – 1.68 (dd, *J* = 18.4, 8.5 Hz, 3H), 1.35 – 1.12 (m, 5H); **^{13}C NMR** (100 MHz, $CDCl_3$) δ 159.1, 151.1 (d, *J* = 12.8 Hz), 149.2 (d, *J* = 13.5), 148.6 (d, *J* = 13.5), 146.7 (d, *J* = 13.5), 138.2, 134.5, 130.3, 128.5, 127.7 (d, *J* = 81.7), 124.8 (d, *J* =

123.6), 122.0 (d, *J* = 16.7), 117.9, 117.3 (d, *J* = 18.0), 114.0, 106.6, 97.3, 88.8, 83.4, 75.0, 69.8, 69.3, 69.0, 37.9, 29.9, 26.5, 25.8; **HRMS** (ESI) calculated for [$C_{35}H_{30}F_2FeNaO$] [M+Na]$^+$: 583.1507, found: 583.1515; **IR** (KBr, cm^{-1}) 2926, 1589, 1508, 1470, 1274, 808, 750, 500.

7.22 1-((2-ferrocene-3-chloro-5-fluorophenyl)ethynyl)-2-(cyclohexylmethoxy)naphthalene (1av)

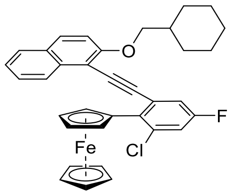

Isolated **1av** in 80% yield as orange-yellow solid.
^{1}H NMR (400 MHz, $CDCl_3$) δ 8.11 (d, *J* = 8.4 Hz, 1H), 7.79 (d, *J* = 9.1 Hz, 1H), 7.75 (d, *J* = 8.1 Hz, 1H), 7.49 – 7.43 (m, 1H), 7.38 – 7.33 (m, 1H), 7.32– 7.30 (m, 1H), 7.22 (d, *J* = 9.2 Hz, 1H), 7.16 (dd, *J* = 8.4, 2.8 Hz, 1H), 5.15 – 5.11 (m, 2H), 4.42 – 4.37 (m, 2H), 4.16 (s, 5H), 4.00 (d, *J* = 6.0 Hz, 2H), 2.01 – 1.90 (m, 3H), 1.83 – 1.71 (m, 3H), 1.26– 1.14 (m, 5H); **^{13}C NMR** (100 MHz, $CDCl_3$) δ 160.7, 159.3, 158.3, 134.6, 134.4,134.0 (d, *J* = 3.7 Hz), 130.5, 128.4, 128.0, 127.3, 127.1 (d, *J* = 10.1 Hz), 125.6, 124.2, 119.5 (d, *J* = 21.9 Hz), 118.2 (d, *J* = 24.3 Hz), 113.9, 106.5, 98.4, 98.4, 90.8, 82.0, 74.9, 71.5, 69.8, 68.0, 38.1, 30.0, 26.5, 25.8; **HRMS** (ESI) calculated for [$C_{35}H_{30}ClFFeNaO$] [M+Na]$^+$: 599.1212, found: 599.1214; **IR** (KBr, cm^{-1}) 2926, 2372, 1589, 1508, 1277, 808, 750, 500.

7.23 1-((2-ferrocene-5-(trifluoromethyl)phenyl)ethynyl)-2-(cyclohexylmethoxy)naphthalene (1aw)

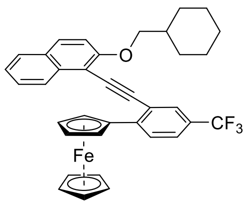

Isolated **1aw** in 85% yield as orange-yellow solid.
^{1}H NMR (400 MHz, $CDCl_3$) δ 8.28 (d, *J* = 8.4 Hz, 1H), 7.87 (d, *J* = 0.8 Hz, 1H), 7.82 (d, *J* = 8.8 Hz, 1H), 7.77 (t, *J* = 8.6 Hz, 2H), 7.54 – 7.46 (m, 2H), 7.40 – 7.35 (m, 1H), 7.35 – 7.35 (m, 1H), 7.24 (d, *J* = 2.8 Hz, 1H), 5.19 – 5.16 (m, 2H), 4.42 – 4.38 (m, 2H), 4.09 (s, 5H), 4.03 (d, *J* = 6.2 Hz, 2H), 2.02 – 1.93 (m, 3H), 1.80 – 1.69 (m, 3H), 1.32 – 1.13 (m, 5H); **^{13}C NMR** (100 MHz, $CDCl_3$) δ 159.3, 144.5, 134.5, 130.9 (q, *J* = 3.6 Hz), 130.4, 129.3, 128.5, 128.1, 127.7 (q, *J* = 32.6 Hz), 127.3, 125.5, 124.1 (q, *J* = 3.6 Hz), 122.0, 114.1, 106.6, 98.1, 89.8, 83.3, 75.1, 69.9, 69.7, 69.4, 38.0, 30.0, 26.5, 25.8; **HRMS** (ESI) calculated for [$C_{36}H_{31}F_3FeNaO$] [M+Na]$^+$: 615.1569, found: 615.1560; **IR** (KBr, cm^{-1}) 2926, 1508, 1330, 1274, 1078, 808, 750, 500.

7.24 1-(4-ferrocene-3-((2-(cyclohexylmethoxy)naphthalen-1-yl)ethynyl)phenyl)ethan-1-one (1ax)

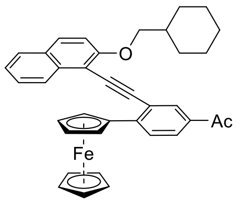

Isolated **1ax** in 89% yield as orange-yellow solid.
^{1}H NMR (400 MHz, $CDCl_3$) δ 8.32 (d, *J* = 8.4 Hz, 1H), 8.21 (d, *J* = 2.0 Hz, 1H), 7.87 – 7.76 (m, 3H), 7.72 (d, *J* = 8.4 Hz, 1H), 7.52 (ddd, *J* = 8.0, 6.8, 1.2 Hz, 1H), 7.38 (ddd, *J* = 8.0, 6.8, 1.2 Hz, 1H), 7.28 (d, *J* = 9.2 Hz, 1H), 5.27 – 5.21 (m, 2H), 4.44 – 4.38 (m, 2H), 4.08 (s, 5H), 4.05 (d, *J* = 6.0 Hz, 2H), 2.63 (s, 3H), 2.04 – 1.93 (m, 3H), 1.79 – 1.67 (m, 3H), 1.37 – 1.07 (m, 5H); **^{13}C NMR** (100 MHz, $CDCl_3$) δ 197.0, 159.0, 146.1, 134.5, 134.3, 130.2, 129.0, 128.5, 128.1, 127.3, 127.1, 125.5, 124.2, 121.5, 114.1, 106.8, 98.5, 89.1, 83.2, 75.1, 69.9, 69.7, 69.6, 37.9, 30.0, 26.5, 25.8; **HRMS** (ESI) calculated for [$C_{37}H_{34}FeNaO_2$] [M+Na]$^+$: 589.1801, found: 589.1799; **IR** (KBr, cm^{-1}) 2926, 1683, 1591, 1508, 1274, 808, 750, 500.

7.25 1-((2-ferrocene-4-(trifluoromethyl)phenyl)ethynyl)-2-(cyclohexylmethoxy)naphthalene (1ay)

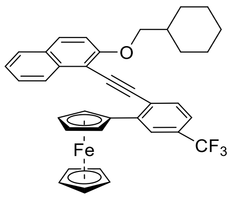

Isolated **1ay** in 81% yield as orange-yellow solid.

^{1}H NMR (400 MHz, $CDCl_3$) δ 8.26 (d, *J* = 8.4 Hz, 1H), 7.91 (s, 1H), 7.82 (d, *J* = 9.2 Hz, 1H), 7.78 (d, *J* = 8.0 Hz, 1H), 7.72 (d, *J* = 8.0 Hz, 1H), 7.54 – 7.47 (m, 1H), 7.45 – 7.41 (m, 1H), 7.40 – 7.35 (m, 1H), 7.24 (s, 1H), 5.19 – 5.14 (m, 2H), 4.41 – 4.36 (m, 2H), 4.10 (s, 5H), 4.04 (d, *J* = 6.1 Hz, 2H), 2.02 – 1.91 (m, 3H), 1.82 – 1.69 (m, 3H), 1.34 – 1.14 (m, 5H); **^{13}C NMR** (100 MHz, $CDCl_3$) δ 159.2, 141.5, 134.6, 134.3, 130.5, 129.4 (q, *J* = 32.4 Hz), 128.5, 128.1, 127.4, 125.8 (q, *J* = 3.9 Hz), 125.4, 125.0, 124.2, 122.8, 121.9 (q, *J* = 3.5 Hz), 114.0, 106.6, 98.2, 90.9, 83.8, 75.0, 69.8, 69.5, 69.2, 38.0, 30.0, 26.5, 25.8; **HRMS** (ESI) calculated for [$C_{36}H_{31}F_3FeNaO$] $[M+Na]^+$: 615.1569, found: 615.1569; **IR** (KBr, cm^{-1}) 2926, 1502, 1329, 1277, 1128, 808, 750, 500.

7.26 methyl 3-ferrocene-4-((2-(cyclohexylmethoxy)naphthalen-1-yl)ethynyl)benzoate (1az)

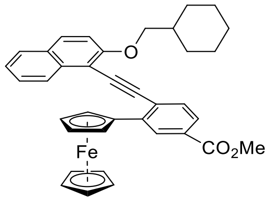

Isolated **1az** in 88% yield as orange-yellow solid.

^{1}H NMR (400 MHz, $CDCl_3$) δ 8.29 (d, *J* = 1.6 Hz, 1H), 8.21 (d, *J* = 8.0 Hz, 1H), 7.77 (dd, *J* = 8.0, 1.6 Hz, 1H), 7.76 – 7.69 (m, 2H), 7.61 (d, *J* = 8.0 Hz, 1H), 7.47 – 7.40 (m, 1H), 7.32 – 7.27 (m, 1H), 7.17 (d, *J* = 1.6 Hz, 1H), 5.14 – 5.11 (m, 2H), 4.32 – 4.28 (m, 2H), 4.03 (s, 5H), 3.96 (d, *J* = 6.0 Hz, 2H), 3.90 (s, 3H), 1.94 – 1.84 (m, 3H), 1.73 – 1.61 (m, 3H), 1.26 – 1.06 (m, 5H); **^{13}C NMR** (100 MHz, $CDCl_3$) δ 166.9, 159.2, 140.8, 134.6, 134.0, 130.5, 130.3, 129.0, 128.5, 128.1, 127.3, 126.2, 126.0, 125.5, 124.2, 114.0, 106.7, 98.9, 91.5, 84.2, 75.0, 69.8, 69.5, 69.0, 52.3, 37.96, 30.0, 26.5, 25.8; **HRMS** (ESI) calculated for [$C_{37}H_{34}FeNaO_3$] $[M+Na]^+$: 605.1750, found: 605.1743; **IR** (KBr, cm^{-1}) 2951, 1722, 1508, 1275, 1018, 810, 750, 500.

7.27 1-((2-ferrocene-4-methylphenyl)ethynyl)-2-(cyclohexylmethoxy)naphthalene (1ba)

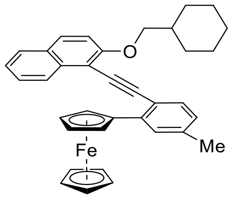

Isolated **1aa** in 83% yield as orange-yellow solid.

^{1}H NMR (400 MHz, $CDCl_3$) δ 8.25 (d, *J* = 8.4 Hz, 1H), 7.69 (dd, *J* = 8.8, 3.6 Hz, 2H), 7.47 (d, *J* = 7.8 Hz, 1H), 7.44 – 7.37 (m, 2H), 7.29 (d, *J* = 8.0 Hz, 1H), 7.17 (d, *J* = 8.8 Hz, 1H), 6.96 (d, *J* = 7.6 Hz, 1H), 5.08 – 5.03 (m, 2H), 4.27 – 4.22 (m, 2H), 4.01 (s, 5H), 3.95 (d, *J* = 6.2 Hz, 2H), 2.34 (s, 3H), 1.94 – 1.84 (m, 3H), 1.72 – 1.60 (m, 3H), 1.24 – 1.06 (m, 5H); **^{13}C NMR** (100 MHz, $CDCl_3$) δ 158.7, 140.1, 137.7, 134.7, 134.1, 129.6, 129.6, 128.6, 128.0, 127.0, 126.7, 125.8, 124.1, 118.8, 114.3, 107.6, 99.7, 87.7, 85.2, 75.1, 69.7, 69.3, 68.6, 38.0, 30.0, 26.6, 25.9, 21.6; **HRMS** (ESI) calculated for [$C_{36}H_{34}FeNaO$] $[M+Na]^+$: 561.1852, found: 561.1857; **IR** (KBr, cm^{-1}) 2924, 1589, 1508, 1273, 1018, 810, 750, 500.

7.28 1-((2-ferrocene-4-(trifluoromethoxy)phenyl)ethynyl)-2-(cyclohexylmethoxy)naphthalene (1bb)

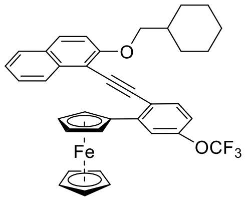

Isolated **1bb** in 84% yield as orange-yellow solid.

^{1}H NMR (400 MHz, $CDCl_3$) δ 8.25 (d, *J* = 8.4 Hz, 1H), 7.79 (t, *J* = 9.2 Hz, 2H), 7.64 (d, *J* = 8.4 Hz, 1H), 7.53 (s, 1H), 7.49 (t, *J* = 7.6 Hz, 1H), 7.36 (t, *J* = 7.4 Hz, 1H), 7.24 (d, *J* = 1.6 Hz, 1H), 7.06 (d, *J* = 8.8 Hz, 1H), 5.16 – 5.11 (m, 2H), 4.39 – 4.35 (m, 2H), 4.10 (s, 5H), 4.03 (d, *J* = 6.0 Hz, 2H), 2.00 – 1.88 (m, 3H), 1.81 – 1.69 (m, 3H), 1.33 – 1.13 (m, 5H); **^{13}C NMR** (100 MHz, $CDCl_3$) δ 159.0, 148.4, 143.0, 135.3, 134.6, 130.2, 128.5, 128.1, 127.2, 125.5, 124.2, 121.2, 120.6 (q, *J* = 256 Hz), 120.3, 118.0, 114.1, 106.9, 98.1, 89.1, 83.6, 75.0, 69.8, 69.5, 69.1, 38.0, 30.0, 26.5, 25.9; **HRMS** (ESI) calculated for

[$C_{36}H_{31}F_3FeNaO_2$] [M+Na]$^+$: 631.1518, found: 631.1515; **IR** (KBr, cm^{-1}) 2926, 1508, 1255, 1219, 1015, 810, 750, 500.

7.29 1-((2-ferrocene-5-methylphenyl)ethynyl)-2-(cyclohexylmethoxy)naphthalene (1bc)

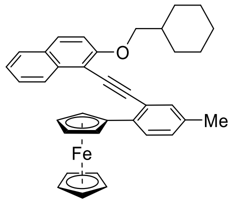

Isolated **1bc** in 80% yield as orange-yellow solid.

^{1}H NMR (400 MHz, $CDCl_3$) δ 8.32 (d, *J* = 8.4 Hz, 1H), 7.79 – 7.75 (m, 2H), 7.57 (d, *J* = 8.0 Hz, 1H), 7.52 – 7.45 (m, 2H), 7.39 – 7.32 (m, 1H), 7.24 (d, *J* = 2.4 Hz, 1H), 7.11– 7.09 (m, 1H), 5.10 (t, *J* = 2.0 Hz, 2H), 4.31 (t, *J* = 2.0 Hz, 2H), 4.08 (s, 5H), 4.02 (d, *J* = 6.2 Hz, 2H), 2.35 (s, 3H), 2.03 – 1.93 (m, 3H), 1.82 – 1.69 (m, 3H), 1.37 – 1.15 (m, 6H); **^{13}C NMR** (100 MHz, $CDCl_3$) δ 158.8, 137.4, 135.2, 134.6, 134.4, 129.7, 129.0, 128.9, 128.6, 128.0, 127.1, 125.8, 124.1, 121. 5, 114.2, 107.5, 99.7, 88.0, 85.3, 77.3, 77.0, 76.7, 75.1, 69.6, 69.3, 68.4, 38.0, 30.0, 26.6, 25.9, 20.9; **HRMS** (ESI) calculated for [$C_{36}H_{34}FeNaO$] [M+Na]$^+$: 561.1852, found: 561.1858; **IR** (KBr, cm^{-1}) 2924, 2372, 1508, 1275, 1210, 818, 750, 500.

7.30 1-((2-ferrocene-5-(tert-butyl)phenyl)ethynyl)-2-(cyclohexylmethoxy)naphthalene (1bd)

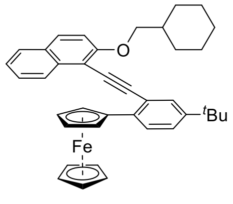

Isolated **1bd** in 71% yield as orange-yellow solid.

^{1}H NMR (400 MHz, $CDCl_3$) δ 8.30 (d, *J* = 8.4 Hz, 1H), 7.78 – 7.75 (m, 2H), 7.64 – 7.61 (m, 2H), 7.47 (t, *J* = 8.0 Hz, 1H), 7.38 – 7.30 (m, 2H), 7.24 (s, 1H), 5.06 (d, *J* = 1.5 Hz, 2H), 4.30 (d, *J* = 1.6 Hz, 2H), 4.09 (s, 5H), 4.02 (d, *J* = 6.3 Hz, 2H), 2.03 – 1.92 (m, 3H), 1.79 – 1.67 (m, 3H), 1.37 (s, 9H), 1.30 – 1.11 (m, 5H); **^{13}C NMR** (100 MHz, $CDCl_3$) δ 158.8, 148.5, 137.4, 134.6, 130.6, 129.7, 129.0, 128.6, 128.0, 127.0, 125.9, 125.3, 124.1, 121.2, 114.4, 107.6, 100.0, 87.4, 85.4, 75.2, 69.6, 69.4, 68.4, 38.0, 34.4, 31.2, 30.0, 26.6, 25.9; **HRMS** (ESI) calculated for [$C_{39}H_{40}FeNaO$] [M+Na]$^+$: 603.2321, found: 603.2321; **IR** (KBr, cm^{-1}) 2926, 2372, 1508, 1274, 1018, 808, 750, 500.

7.31 1-((2-ferrocene-5-methoxyphenyl)ethynyl)-2-(cyclohexylmethoxy)naphthalene (1be)

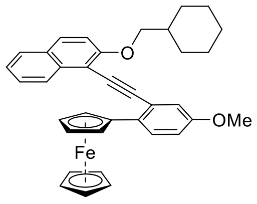

Isolated **1be** in 62% yield as orange-yellow solid.

^{1}H NMR (400 MHz, $CDCl_3$) δ 8.32 (d, *J* = 8.4 Hz, 1H), 7.77 (d, *J* = 8.8 Hz, 2H), 7.59 (d, *J* = 8.8 Hz, 1H), 7.51 – 7.47 (m, 1H), 7.38 – 7.34 (m, 1H), 7.24 – 7.22 (m, 2H), 6.81 – 6.78 (m, 1H), 5.18 – 5.11 (m, 2H), 4.37 – 4.31 (m, 2H), 4.10 (s, 5H), 4.03 (d, *J* = 6.4 Hz, 2H), 3.89 (s, 3H), 2.03 – 1.92 (m, 3H), 1.81 – 1.69 (m, 3H), 1.34 – 1.14 (m, 5H); **^{13}C NMR** (100 MHz, $CDCl_3$) δ 159.1, 158.6, 142.1, 135. 4, 134.6, 129.4, 128.6, 128.0, 127.0, 125.8, 124.0, 114.4, 114.4, 114.3, 111.6, 107.8, 99.5, 86.9, 85.0, 75.1, 69.7, 69.4, 68.7, 55.3, 38.0, 30.0, 26.6, 25.9; **HRMS** (ESI) calculated for [$C_{36}H_{34}FeNaO_2$] [M+Na]$^+$: 577.1801, found: 577.1806; **IR** (KBr, cm^{-1}) 2927, 1601, 1508, 1275, 1069, 810, 750, 500.

7.32 1-((2-ferrocene-5-(trifluoromethoxy)phenyl)ethynyl)-2-(cyclohexylmethoxy)naphthalene (1bf)

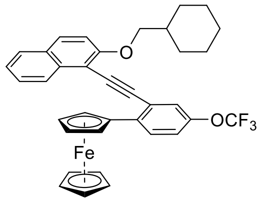

Isolated **1bf** in 85% yield as orange-yellow solid.

^{1}H NMR (400 MHz, $CDCl_3$) δ 8.24 (d, *J* = 8.4 Hz, 1H), 7.81 – 7.76 (m, 2H), 7.68 (d, *J* = 8.4 Hz, 1H), 7.51 – 7.46 (m, 2H), 7.37 (t, *J* = 7.6 Hz, 1H), 7.24 (d, *J* = 1.2 Hz, 1H), 7.15 – 7.12 (m, 1H), 5.12 – 5.06 (m, 2H), 4.39 – 4.33 (m, 2H), 4.09 (s, 5H), 4.02 (d, *J* = 6.0 Hz, 2H), 2.02 – 1.91 (m, 3H), 1.81 – 1.69 (m, 3H), 1.33 – 1.13 (m, 5H); **^{13}C NMR** (100 MHz, $CDCl_3$) δ 159.2, 146.7, 139. 5, 134.5, 130.4, 130.4,

128.5, 128.1, 127.3, 125.8, 125.5, 124.2, 123.0, 120.5(q, *J* = 257 Hz), 120.5, 114.1, 106.6, 97.9, 89.7, 84.0, 75.1, 69.8, 69.5, 68.9, 38.0, 30.0, 26.5, 25.8; **HRMS** (ESI) calculated for [$C_{36}H_{31}F_3FeNaO_2$] $[M+Na]^+$: 631.1518, found: 631.1526; **IR** (KBr, cm^{-1}) 2928, 1508, 1260, 1219, 1169, 810, 750, 500.

7.33 1-((2-ferrocene-6-methylphenyl)ethynyl)-2-(cyclohexylmethoxy)naphthalene (1bg)

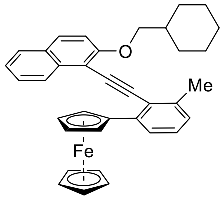

Isolated **1bg** in 69% yield as orange-yellow solid.

^{1}H NMR (400 MHz, $CDCl_3$) δ 8.23 (d, *J* = 8.4 Hz, 1H), 7.77 (t, *J* = 8.0 Hz, 2H), 7.61 (d, *J* = 7.2 Hz, 1H), 7.45 – 7.39 (m, 1H), 7.37 – 7.32 (m, 1H), 7.25 – 7.15 (m, 3H), 5.05 – 5.03 (m,, 2H), 4.35 – 4.29 (m, 2H), 4.10 (s, 5H), 4.00 (d, *J* = 6.4 Hz, 2H), 2.68 (s, 3H), 2.02 – 1.90 (m, 3H), 1.79 – 1.68 (m, 3H), 1.32 – 1.07 (m, 5H); **^{13}C NMR** (100 MHz, $CDCl_3$) δ 158.6, 141.2, 140.8, 134.5, 129.7, 128.4, 127.9, 127.3, 127.2, 127.1, 127.0, 126.0, 124.0, 122.1, 113.9, 107.4, 97.7, 92.8, 86.3, 75.0, 70.2, 69.6, 68.3, 37.9, 30.1, 26.5, 25.8, 21.9; **HRMS** (ESI) calculated for [$C_{36}H_{34}FeNaO$] $[M+Na]^+$: 561.1852, found: 561.1861; **IR** (KBr, cm^{-1}) 2924, 2375, 1508, 1275, 1210, 818, 750, 500.

7.34 6-bromo-1-((2-ferrocenephenyl)ethynyl)-2-(cyclohexylmethoxy)naphthalene (1bh)

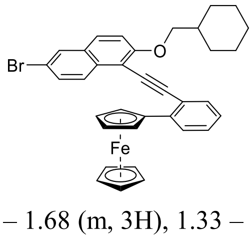

Isolated **1bh** in 72% yield as orange-yellow solid.

^{1}H NMR (400 MHz, $CDCl_3$) δ 8.13 (d, *J* = 8.8 Hz, 1H), 7.91 (d, *J* = 2.0 Hz, 1H), 7.68 (t, *J* = 9.2 Hz, 2H), 7.62 (d, *J* = 7.6 Hz, 1H), 7.52 (dd, *J* = 8.8, 2.0 Hz, 1H), 7.33 – 7.27 (m, 1H), 7.25 – 7.17 (m, 2H), 5.10 – 5.06 (m, 2H), 4.38 – 4.32 (m, 2H), 4.09 (s, 5H), 4.01 (d, *J* = 6.4 Hz, 2H), 2.01 – 1.90 (m, 3H), 1.82 – 1.68 (m, 3H), 1.33 – 1.14 (m, 5H); **^{13}C NMR** (100 MHz, $CDCl_3$) δ 158.9, 140.6, 133.9, 133.1, 130.3, 129.9, 129.6, 129.3, 128.7, 128.0, 127.7, 125.7, 121.5, 117.8, 115.2, 107.6, 99.9, 87.6, 85.2, 75.1, 69.7, 69.5, 68.7, 38.0, 29.9, 26.5, 25.9; **HRMS** (ESI) calculated for [$C_{35}H_{31}BrFeNaO$] $[M+Na]^+$: 625.0802, found: 625.0817; **IR** (KBr, cm^{-1}) 2922, 2372, 1497, 1274, 1070, 810, 758, 500.

7.35 7-bromo-1-((2-ferrocenephenyl)ethynyl)-2-(cyclohexylmethoxy)naphthalene (1bi)

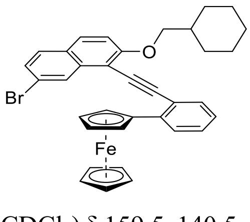

Isolated **1bi** in 73% yield as orange-yellow solid.

^{1}H NMR (400 MHz, $CDCl_3$) δ 8.48 (s, 1H), 7.74 – 7.68 (m, 2H), 7.66 – 7.58 (m, 2H), 7.44 – 7.43 (m, 1H), 7.32 – 7.28 (m, 1H), 7.24 – 7.20 (m, 2H), 5.11 – 5.10 (m, 2H), 4.41 – 4.40 (m, 2H), 4.10 (s, 5H), 4.11 (d, *J* = 6.0 Hz, 2H), 2.03 – 1.91 (m, 3H), 1.80 – 1.70 (m, 3H), 1.35 – 1.14 (m, 5H); **^{13}C NMR** (100 MHz, $CDCl_3$) δ 159.5, 140.5, 135.7, 134.1, 129.7, 129.6, 129.1, 127.9, 127.7, 127.5, 126.9, 125.6, 121.9, 121.4, 114.2, 106.6, 100.0, 87.6, 84.9, 74.9, 70.0, 69.3, 69.0, 38.0, 29.900, 26.5, 25.8; **HRMS** (ESI) calculated for [$C_{35}H_{31}BrFeNaO$] $[M+Na]^+$: 625.0802, found: 625.0805; **IR** (KBr, cm^{-1}) 2922, 2370, 1497, 1275, 1070, 810, 758, 500.

7.36 7-(benzyloxy)-1-((2-ferrocenephenyl)ethynyl)-2-(cyclohexylmethoxy)naphthalene (1bj)

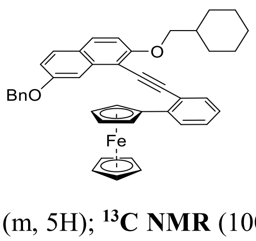

Isolated **1bj** in 79% yield as orange-yellow solid.

^{1}H NMR (400 MHz, $CDCl_3$) δ 7.74 – 7.66 (m, 4H), 7.60 (dd, *J* = 7.8, 1.2 Hz, 1H), 7.47 – 7.42 (m, 2H), 7.38 – 7.28 (m, 4H), 7.24– 7.20 (m, 1H), 7.12 – 7.07 (m, 2H), 5.17 – 5.15 (m, 2H), 5.07 (s, 2H), 4.31 – 4.28 (m, 2H), 4.07 (s, 5H), 4.04 (d, *J* = 6.4 Hz, 2H), 2.05 – 1.93 (m, 3H), 1.83 – 1.70 (m, 3H), 1.37 – 1.16 (m, 5H); **^{13}C NMR** (100 MHz, $CDCl_3$) δ 159.4, 158.2, 140.3, 136.9, 136.1, 134.0, 129.7, 129.6, 129.1,

128.5, 127.9, 127.8, 127.7, 125.6, 124.1, 121.6, 117.3, 111.6, 106.4, 105.1, 99.4, 88.5, 85.0, 74.9, 70.0, 69.8, 69.5, 68.7, 38.0, 30.0, 26.6, 25.9; **HRMS** (ESI) calculated for [$C_{42}H_{38}FeNaO_2$] [M+Na]$^+$: 653.2114, found: 653.2107; **IR** (KBr, cm^{-1}) 3061, 1590, 1508, 1265, 1068, 810, 750, 500.

7.37 3-(benzyloxy)-1-((2-ferrocenephenyl)ethynyl)-2-(cyclohexylmethoxy)naphthalene (1bk)

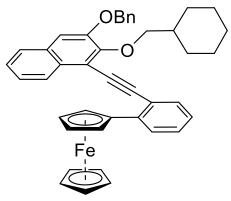

Isolated **1bk** in 80% yield as orange-yellow solid.

^{1}H NMR (400 MHz, $CDCl_3$) δ 8.26 – 8.21 (m, 1H), 7.74 (d, *J* = 7.2 Hz, 1H), 7.71 – 7.64 (m, 2H), 7.53 (d, *J* = 7.2 Hz, 2H), 7.47 – 7.30 (m, 7H), 7.24 – 7.22 (m, 1H), 5.24 (s, 2H), 5.10 – 5.08 (m, 2H), 4.37 – 4.32 (m, 2H), 4.11 (s, 5H), 4.07 (d, *J* = 6.4 Hz, 2H), 1.96 – 1.84 (m, 3H), 1.70 – 1.64 (m, 3H), 1.23 – 1.03 (m, 5H); **^{13}C NMR** (100 MHz, $CDCl_3$) δ 151.7, 151.2, 140.8, 136.7, 133.9, 130.6, 129.6, 129.3, 128.5, 128.0, 128.0, 127.6, 126.7, 126.0, 125.6, 125.6, 124.7, 121.3, 114.1, 109.9, 99.1, 87.8, 85.0, 79.8, 70.8, 69.7, 69.6, 68.7, 38.8, 29.9, 26.6, 25.9; **HRMS** (ESI) calculated for [$C_{42}H_{38}FeNaO_2$] [M+Na]$^+$: 653.2114, found: 653.2106; **IR** (KBr, cm^{-1}) 2924, 2372, 1450, 1250, 1007, 831, 750, 500.

7.38 methyl 5-((2-ferrocenephenyl)ethynyl)-6-(cyclohexylmethoxy)-1-naphthoate (1bl)

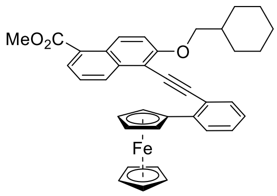

Isolated **1bl** in 84% yield as orange-yellow solid.

^{1}H NMR (400 MHz, $CDCl_3$) δ 8.93 (d, *J* = 9.6 Hz, 1H), 8.54 (d, *J* = 8.4 Hz, 1H), 8.07 (dd, *J* = 7.2, 1.2 Hz, 1H), 7.71 (dd, *J* = 7.6, 0.8 Hz, 1H), 7.65 (dd, *J* = 7.7, 1.2 Hz, 1H), 7.51 – 7.48 (m, 1H), 7.36 (s, 1H), 7.34 – 7.29 (m, 1H), 7.25 – 7.21 (m, 1H), 5.13 – 5.10 (m, 2H), 4.36 – 4.34 (m, 2H), 4.10 (s, 5H), 4.07 (d, *J* = 6.0 Hz, 2H), 4.00 (s, 3H), 1.9– 1.70 (m, 3H), 2.00 – 1.94 (m, 3H), 1.37 – 1.22 (m, 5H); **^{13}C NMR** (100 MHz, $CDCl_3$) δ 168.0, 158.7, 140.5, 135.3, 133.9, 131.0, 129.2, 128.4, 127.9, 127.8, 127.2, 126.4, 125.7, 125.6, 121.5, 115.5, 107.7, 99.9, 88.0, 85.2, 74.9, 69.7, 69.5, 68.6, 52.2, 37.9, 29.9, 26.5, 25.9; **HRMS** (ESI) calculated for [$C_{37}H_{34}FeNaO_3$] [M+Na]$^+$: 605.1750, found: 605.1756; **IR** (KBr, cm^{-1}) 2928, 1717, 1508, 1265, 1099, 810, 752, 495.

7.39 5-((2-ferrocenephenyl)ethynyl)-6-(cyclohexylmethoxy)-1,2,3,4-tetrahydronaphthalene (1bm)

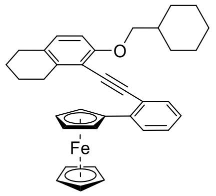

Isolated **1bm** in 71% yield as orange-yellow solid.

^{1}H NMR (400 MHz, $CDCl_3$) δ 7.62 (d, *J* = 8.0 Hz, 1H), 7.54 – 7.52 (m,, 1H), 7.27 – 7.22 (m, 1H), 7.17 – 7.13 (m,, 1H), 6.97 (d, *J* = 8.4 Hz, 1H), 6.68 (d, *J* = 8.4 Hz, 1H), 5.11 (s, 2H), 4.30 (s, 2H), 4.08 (s, 5H), 3.84 (d, *J* = 6.2 Hz, 2H), 2.92 (t, *J* = 6.0 Hz, 2H), 2.69 (t, *J* = 6.0 Hz, 2H), 1.97 – 1.85 (m, 3H), 1.81 – 1.66 (m, 7H), 1.31 – 1.10 (m, 5H); **^{13}C NMR** (100 MHz, $CDCl_3$) δ 158.2, 140.7, 140.1, 134.2, 129.6, 129.3, 128.8, 127.6, 125.5, 121.7, 112.6, 109.3, 99.1, 89.1, 85.0, 74.2, 69.7, 69.3, 68.6, 37.7, 30.0, 29.1, 28.7, 26.6, 25.9, 23.1; **HRMS** (ESI) calculated for [$C_{35}H_{36}FeNaO$] [M+Na]$^+$: 551.2008, found: 551.2014; **IR** (KBr, cm^{-1}) 2960, 1590, 1508, 1270, 1065, 810, 750, 500.

7.40 methyl (*S*)-2-(5-((2-ferrocenephenyl)ethynyl)-6-(cyclohexylmethoxy)naphthalen-2-yl)propanoate (1bn)

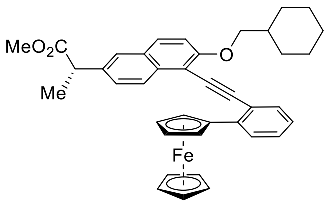

Isolated **1bn** in 87% yield as orange-yellow solid.

^{1}H NMR (400 MHz, $CDCl_3$) δ 8.27 (d, *J* = 8.8 Hz, 1H), 7.75 (d, *J* = 9.2 Hz, 1H), 7.70 – 7.62 (m, 3H), 7.46 – 7.43 (m, 1H), 7.31– 7.27 (m, 1H), 7.24 (d, *J* = 4.8 Hz, 1H), 7.23 – 7.19 (m, 1H), 5.15 – 5.10 (m, 2H), 4.37 – 4.32 (m, 2H), 4.09 (s, 5H), 4.03 (d, *J* = 6.2 Hz, 2H), 3.87 (q, *J* = 7.2 Hz, 1H), 3.67 (s, 3H), 2.03 – 1.92 (m, 3H), 1.82 – 1.69 (m, 3H), 1.59 (d, *J* = 7.2 Hz, 3H), 1.34 – 1.13 (m, 5H); **^{13}C NMR** (100 MHz, $CDCl_3$) δ 175.0, 158.8, 140.4, 136.2, 134.0, 133.8, 129.7, 129.1, 128.6, 127.8, 127.0, 126.2, 126.2, 125.6, 121.6, 114.6, 107.4, 99.5, 88.3, 85.1, 75.1, 69.7, 69.4, 68.7, 52.1, 45.3, 38.0, 30.0, 26.5, 25.9, 18.5; **HRMS** (ESI) calculated for [$C_{39}H_{38}FeNaO_3$] [M+Na]$^+$: 633.2063, found: 633.2059; **IR** (KBr, cm^{-1}) 2926, 1740, 1591, 1460, 1277, 1070, 810, 750, 500.

7.41 1-((2-ferrocene-5-(trifluoromethyl)phenyl)ethynyl)-2-(methyl)naphthalene (1bo)

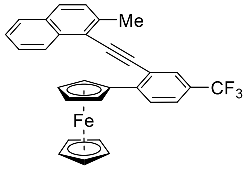

Isolated **1bo** in 85% yield as orange-yellow solid.

^{1}H NMR (500 MHz, $CDCl_3$) δ 8.36 (d, *J* = 8.5 Hz, 1H), 7.88 (s, 1H), 7.84 (dd, *J* = 8.0, 3.0 Hz, 2H), 7.78 (d, *J* = 8.5 Hz, 1H), 7.58 – 7.52 (m, 2H), 7.51 – 7.45 (m, 1H), 7.40 (d, *J* = 8.0 Hz, 1H), 5.10 – 5.05 (m, 2H), 4.43 – 4.38 (m, 2H), 4.12 (s, 5H), 2.72 (s, 3H); **^{13}C NMR** (125 MHz, $CDCl_3$) δ 144.8, 139.6, 133.6 (q, *J* = 3.8 Hz), 130.7, 130.6, 129.8, 128.6, 128.2, 127.9 (q, *J* = 32.5 Hz), 127.4 (q, *J* = 26.9 Hz), 127.0, 125.9 (q, *J* = 61.0 Hz), 125.1, 124.4 (q, *J* = 3.5 Hz), 123.0, 121.8, 119.1, 97.9, 91.6, 83.5, 69.9, 69.9, 69.3, 21.7; **HRMS** (ESI) calculated for [$C_{30}H_{21}F_3Fe$] [M+Na]$^+$: 517.0838, found: 517.0836; **IR** (KBr, cm^{-1}) 3005, 2318, 1500, 1277, 1126, 808, 750, 500.

7.42 4-(2-(cyclohexylmethoxy)naphthalen-1-yl)-2,3-dihydro-1H-cyclopenta[a]naphthalene (5b)

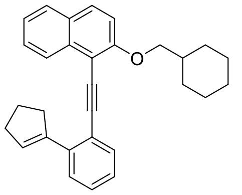

Isolated **5b** in 98% yield as white solid.

^{1}H NMR (400 MHz, $CDCl_3$) δ 8.34 (d, *J* = 8.8 Hz, 1H), 7.78 (dd, *J* = 8.8, 3.4 Hz, 2H), 7.68 (dd, *J* = 7.6, 1.2 Hz, 1H), 7.53 – 7.50 (m, 1H), 7.39 – 7.32 (m, 2H), 7.31 – 7.27 (m, 1H), 7.24 – 7.21 (m, 2H), 6.76 – 6.71 (m, 1H), 4.03 (d, *J* = 6.4 Hz, 2H), 2.94 – 2.87 (m, 2H), 2.66 – 2.56 (m, 2H), 2.09 –1.96 (m, 5H), 1.80 – 1.69 (m, 3H), 1.33 – 1.14 (m, 5H); **^{13}C NMR** (100 MHz, $CDCl_3$) δ 158.8, 142.0, 139.2, 134.7, 133.8, 131.0, 129.8, 128.6, 128.0, 127.9, 127.6, 127.1, 126.3, 125. 5, 124.1, 121.6, 114.4, 107.4, 99.4, 88.2, 75.2, 38.0, 35.6, 34.0, 29.9, 26.6, 25.9, 23.6; **HRMS** (ESI) calculated for [$C_{30}H_{30}NaO$] [M+Na]$^+$: 429.2189, found: 429.2187.

8. General procedure for Simultaneous Construction of Axial and Planar Chirality in Ferrocene Derivatives by Gold/TY-Phos-Catalyzed Asymmetric Hydroarylation.

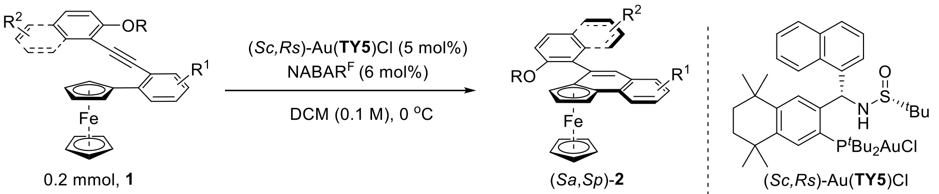

Unless otherwise noted, all reactions were carried out under a nitrogen atmosphere. In a dried Schlenk tube, after the solution of (*Sc*,*Rs*)-Au(**TY5**)Cl (5 mol%, 8.2 mg) and NaBAr^F (6 mol%, 10.6 mg, cas:

79060-88-1, *Energy Chemical*, white powder) in DCM (0.5 mL) was stirred at room temperature for 15 min. Then the above catalyst solution was added to the solution of **1**, **4-5** (0.2 mmol) in DCM (1.5 mL) at 0 °C. The reaction was determined by TLC analysis, after the **1**, **4-5** was consumed completely. Solvent was removed in a rotary evaporator, purified by flash column chromatography on silica gel (Hexane/DCM= 10:1 to 5:1) to afford the desired product **2**, **6**, **7**.

Note: The mixture of (+)- and (-)-**2bz**、(+)- and (-)-**3** with different concentration to gain the HPLC time, all HPLC time difference are less than one minute.

8.1 (*Sa*,*Sp*)-4-(1-(2-methoxy)naphthyl)naphtho[1,2-a]ferrocene. ((*Sa*,*Sp*)-2aa)

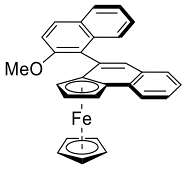

Isolated (-)-**2aa** in 83% yield, 5:1 d.r., as pink solid.

^{1}H NMR (500 MHz, THF-*d_8*) δ 8.11 (s, 1H), 7.97 (s, 1H), 7.82 (s, 1H), 7.63 (s, 2H), 7.47 (s, 1H), 7.39 (s, 1H), 7.26 – 7.07 (m, 4H), 5.37 (s, 1H), 4.02 (s, 5H), 3.74 (s, 5H); **^{13}C NMR** (125 MHz, THF-*d_8*) δ 155.0, 135.7, 135.6, 134.4, 133.4, 130.0, 129.8, 129.3, 128.4, 127.7, 126.9, 126.4, 125.6, 123.82, 123.76, 123.3, 114.1, 87.4, 83.8, 70.0, 69.7, 65.1, 61.7, 56.1; **HRMS** (ESI) calculated for [$C_{29}H_{22}FeNaO$] [M+Na]$^+$: 465.0913, found: 465.0922; **IR** (KBr, cm^{-1}) 2924, 2320, 1748, 1508, 1265, 1061, 808, 500; **[α]$_D^{20}$** = -1157.1 (c = 0.1, $CHCl_3$); **HPLC conditions**: Daicel Chiralpak ADH column (98:2 hexane: 2-propanol, 0.5 mL/min, 254 nm); tr (minor) = 15.1 min, tr (major) = 12.6 min, 85% ee.

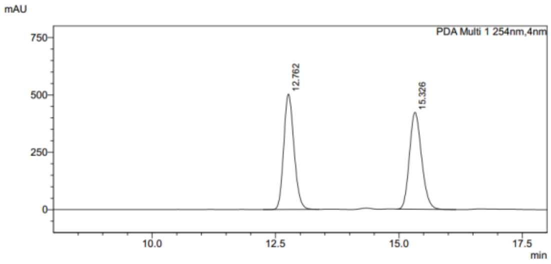

<Peak Table>
PDA Ch1 254nm

Peak#	Ret. Time	Area	Height	Height%	Area%
1	12.762	7280393	502609	54.327	50.182
2	15.326	7227492	422545	45.673	49.818
Total		14507885	925154	100.000	100.000

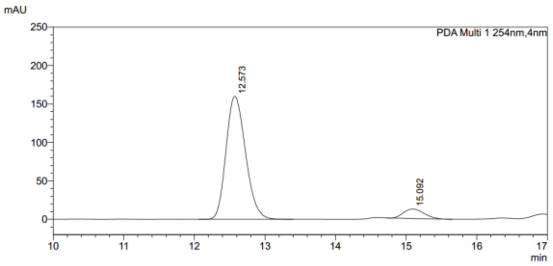

<Peak Table>
PDA Ch1 254nm

Peak#	Ret. Time	Area	Height	Height%	Area%
1	12.573	3069352	159949	92.919	92.633
2	15.092	244103	12189	7.081	7.367
Total		3313454	172138	100.000	100.000

8.2 (*Sa*,*Rp*)-4-(1-(2-methoxy)naphthyl)naphtho[1,2-a]ferrocene. ((*Sa*,*Rp*)-2aa)

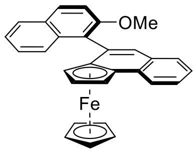

Isolated diastereoisomer of **2aa** in 13% yield as pink solid.

^{1}H NMR (500 MHz, THF-*d_8*) δ 8.18 (s, 1H), 8.11 (s, 1H), 7.98 (s, 1H), 7.90 (s, 1H), 7.64 (s, 1H), 7.50 -7.35 (m, 5H), 7.18 (s, 1H), 5.40 (s, 1H), 4.33 (s, 1H), 4.17 (s, 1H), 3.68 (s, 8H); **^{13}C NMR** (125 MHz, THF-*d_8*) δ 155.3, 136.3, 135.5, 134.2, 133.1, 130.4, 130.0, 129.2, 128.8, 127.4, 127.0, 126.6, 126.2, 125.6, 124.0, 123.9, 123.4, 114.0, 87.8, 83.9, 69.6, 69.4, 65.1, 61.7, 56.1; **IR** (KBr, cm^{-1}) 2924, 2320, 1748, 1508, 1265, 1020, 810, 500; **HRMS** (ESI) calculated for [$C_{29}H_{22}FeNaO$] [M+Na]$^+$: 465.0913, found: 465.0920; ; **IR** (KBr, cm^{-1})

8.3 (*Sa*,*Sp*)-4-(1-(2-ethoxy)naphthyl)naphtho[1,2-a]ferrocene. ((*Sa*,*Sp*)-2ab)

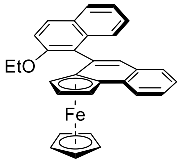

Isolated (-)-**2ab** in 85% yield, 6:1 d.r., as pink solid.

^{1}H NMR (500 MHz, THF-*d_8*) δ 8.11 (d, *J* = 9.0 Hz, 1H), 7.94 (d, *J* = 9.5 Hz, 1H), 7.81 (d, *J* = 8.0 Hz, 1H), 7.66 (d, *J* = 7.5 Hz, 1H), 7.59 (d, *J* = 9.0 Hz, 1H), 7.47 (t, *J* = 7.5 Hz, 1H), 7.39 (t, *J* = 7.5 Hz, 1H), 7.26 – 7.18 (m, 3H), 7.14 – 7.08 (m, 1H), 5.38 (d, *J* = 1.5 Hz, 1H), 4.43 – 4.37 (m, 1H), 4.32 – 4.25 (m, 1H), 4.10 – 4.04 (m, 2H), 3.75 (s, 5H), 1.40 (t, *J* = 7.0 Hz, 3H); **^{13}C NMR** (125 MHz, THF-*d_8*) δ 154.7, 135.9, 135.7, 134.5, 133.7,

130.1, 130.1, 129.4, 128.6, 128.0, 127.1, 126.8, 126.5, 125.9, 124.1, 124.0, 123.9, 115.8, 87.6, 84.0, 70.3, 69.9, 65.7, 65.5, 61.9, 15.6; **HRMS** (ESI) calculated for [$C_{30}H_{24}FeNaO$] $[M+Na]^+$: 479.1069, found: 479.1081; **[α]$_D$^{20}** = -2658.8 (c = 0.1, $CHCl_3$); **IR** (KBr, cm^{-1}) 2960, 2400, 1700, 1500, 1265, 1057, 750, 500; **HPLC conditions**: Daicel Chiralpak ADH column (98:2 hexane: 2-propanol, 0.5 mL/min, 254 nm); tr (minor) = 12.1 min, tr (major) = 11.0 min; 89% ee.

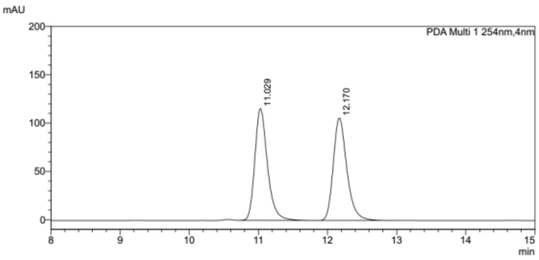

<Peak Table>
PDA Ch1 254nm

Peak#	Ret. Time	Area	Height	Height%	Area%
1	11.029	1470646	115895	52.186	50.272
2	12.170	1454705	106187	47.814	49.728
Total		2925351	222083	100.000	100.000

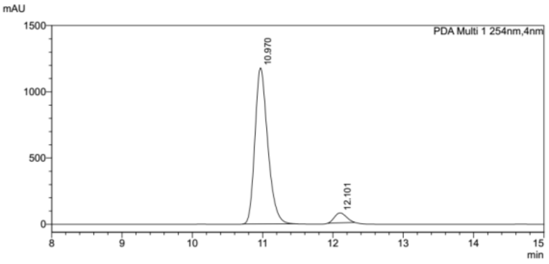

<Peak Table>
PDA Ch1 254nm

Peak#	Ret. Time	Area	Height	Height%	Area%
1	10.970	14944418	1178712	94.023	94.439
2	12.101	880033	74929	5.977	5.561
Total		15824451	1253641	100.000	100.000

8.4 (*Sa,Sp*)-4-(1-(2-propoxy)naphthyl)naphtho[1,2-a]ferrocene. ((*Sa,Sp*)-2ac)

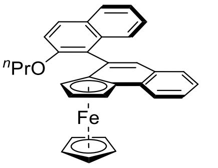

Isolated (-)-**2ac** in 87% yield, 8:1 d.r., as pink solid.

^{1}H NMR (500 MHz, THF-d_8) δ 8.11 (s, 1H), 7.94 (s, 1H), 7.81 (s, 1H), 7.62 (d, J = 15.5 Hz, 2H), 7.68 - 7.55 (m, 2H), 7.24 (d, J = 12.5 Hz, 3H), 7.11 (s, 1H), 5.38 (s, 1H), 4.36 (s, 1H), 4.09 (s, 2H), 3.75 (s, 5H), 3.58 (s, 1H), 1.81 (s, 2H), 1.03 (s, 3H); **^{13}C NMR** (125 MHz, THF-d_8) δ 154.8, 135.7, 134.5, 133.6, 130.1, 129.3, 128.6, 128.2 127.1, 126.8, 126.5, 125.9, 124.0, 124.0, 115.8, 87.5, 84.0, 71.7, 70.2, 69.9, 65.5, 62.0, 23.8, 11.1; **HRMS** (ESI) calculated for [$C_{31}H_{26}FeNaO$] $[M+Na]^+$: 493.1226, found: 493.1236; **IR** (KBr, cm^{-1}) 2963, 2311, 1717, 1511, 1263, 1055, 750, 501; **[α]$_D$^{20}** = -2628.2 (c = 0.1, $CHCl_3$); **HPLC conditions**: Daicel Chiralpak ADH column (98:2 hexane: 2-propanol, 0.5 mL/min, 254 nm); tr (minor) = 10.5 min, tr (major) = 11.0 min; 89% ee.

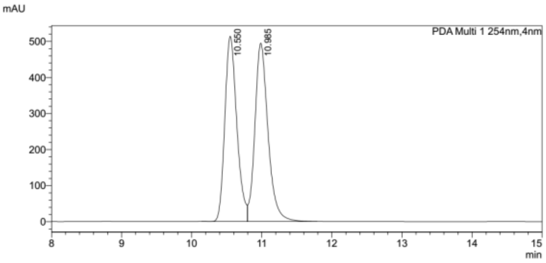

<Peak Table>
PDA Ch1 254nm

Peak#	Ret. Time	Area	Height	Height%	Area%
1	10.550	6108673	513764	50.950	48.774
2	10.985	6415774	494608	49.050	51.226
Total		12524447	1008372	100.000	100.000

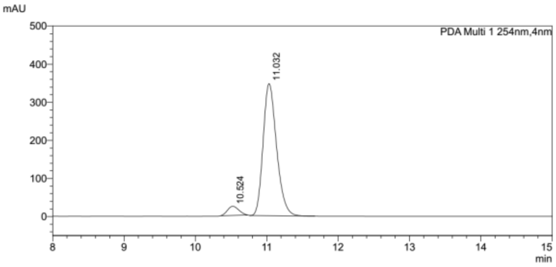

<Peak Table>
PDA Ch1 254nm

Peak#	Ret. Time	Area	Height	Height%	Area%
1	10.524	267379	24018	6.475	5.562
2	11.032	4539862	346895	93.525	94.438
Total		4807241	370913	100.000	100.000

8.5 (*Sa,Sp*)-4-(1-(2-butoxy)naphthyl)naphtho[1,2-a]ferrocene. ((*Sa,Sp*)-2ad)

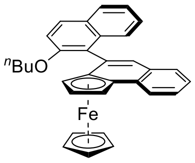

Isolated (-)-**2ad** in 91% yield, 10:1 d.r., as pink solid.

^{1}H NMR (500 MHz, THF-d_8) δ 8.11 (d, J = 6.5 Hz, 1H), 7.94 (d, J = 8.5 Hz, 1H), 7.82 (d, J = 7.0 Hz, 1H), 7.65 – 7.59 (m, 2H), 7.47 (s, 1H), 7.40 (s, 1H), 7.28 – 7.19 (m, 3H), 7.11 (s, 1H), 5.38 (s, 1H), 4.38 (s, 1H), 4.19 (d, J = 6.0 Hz, 1H), 4.08 (d, J = 11.5 Hz, 2H), 3.75 (s, 5H), 1.87 – 1.74 (m, 2H), 1.57 – 1.46 (m, 2H), 0.92 (s, 3H); **^{13}C NMR** (125 MHz, THF-d_8) δ 154.7, 135.6, 135.5, 134.3, 133.4, 129.9, 129.9, 129.1, 128.4, 128.0, 126.9, 126.7, 126.3, 125.7, 123.9, 123.8, 123.7, 115.6, 87.4, 83.8, 70.0, 69.7, 69.6, 65.3, 61.8, 32.4, 19.9, 13.9; **HRMS** (ESI) calculated for [$C_{32}H_{28}FeNaO$] $[M+Na]^+$: 507.1382, found: 507.1386; **IR** (KBr, cm^{-1}) 2959, 2320, 1683, 1506, 1265, 1022, 750, 495; **[α]$_D$^{20}** = -2539.5 (c = 0.1, $CHCl_3$); **HPLC conditions**:

Daicel Chiralpak ADH column (98:2 hexane: 2-propanol, 0.5 mL/min, 254 nm); tr (minor) = 10.0 min, tr (major) = 11.4 min; 90% ee.

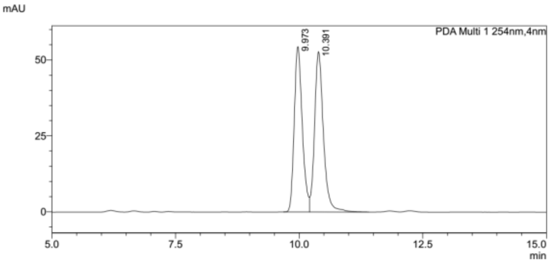

<Peak Table>
PDA Ch1 254nm

Peak#	Ret. Time	Area	Height	Height%	Area%
1	9.973	617051	54442	50.764	48.427
2	10.391	657124	52804	49.236	51.573
Total		1274175	107247	100.000	100.000

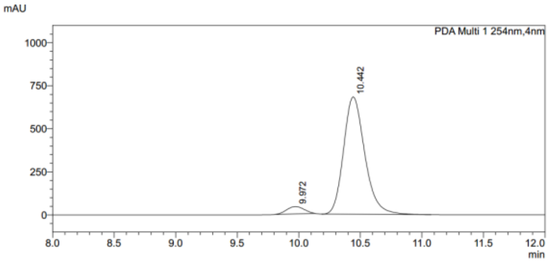

<Peak Table>
PDA Ch1 254nm

Peak#	Ret. Time	Area	Height	Height%	Area%
1	9.972	424626	43234	5.961	4.935
2	10.442	8180256	682069	94.039	95.065
Total		8604882	725303	100.000	100.000

8.6 (*Sa,Sp*)-4-(1-(2-isopropoxy)naphthyl)naphtho[1,2-a]ferrocene. ((*Sa,Sp*)-2ae)

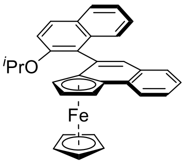

Isolated (-)-**2ae** in 83% yield, 5:1 d.r., as pink solid.

^{1}H NMR (500 MHz, THF-d_8) δ 8.11 (d, *J* = 8.0 Hz, 1H), 7.93 (d, *J* = 9.0 Hz, 1H), 7.80 (d, *J* = 8.0 Hz, 1H), 7.66 (d, *J* = 7.5 Hz, 1H), 7.60 (d, *J* = 9.0 Hz, 1H), 7.50 – 7.45 (m, 1H), 7.41 – 7.39 (m, 1H), 7.26 – 7.20 (m, 2H), 7.17 (d, *J* = 8.5 Hz, 1H), 7.11 – 7.07 (m, 1H), 5.38 – 5.35 (m, 1H), 4.98 – 4.92 (m, 1H), 4.10 – 4.03 (m, 2H), 3.77 (s, 5H), 1.43 (d, *J* = 6.0 Hz, 3H), 1.39 (d, *J* = 6.0 Hz, 3H); **^{13}C NMR** (125 MHz, THF-d_8) δ 153.6, 136.0, 135.7, 134.7, 133.6, 130.0, 130.0, 129.3, 128.5, 127.9, 127.1, 126.6, 126.4, 125.9, 124.7, 124.1, 124.0, 116.9, 87.8, 84.0, 71.8, 70.3, 69.9, 65.5, 61.9, 23.4, 22.7; **HRMS** (ESI) calculated for [$C_{31}H_{26}FeNaO$] [M+Na]$^+$: 493.1226, found: 493.1223; **IR** (KBr, cm^{-1}) 2976, 2310, 1700, 1506, 1263, 1112, 750, 501; **[α]$_D^{20}$** = -2785.3 (c = 0.1, $CHCl_3$); **HPLC conditions**: Daicel Chiralpak ADH column (98:2 hexane: 2-propanol, 0.5 mL/min, 254 nm); tr (minor) = 13.7 min, tr (major) = 11.4 min; 88% ee.

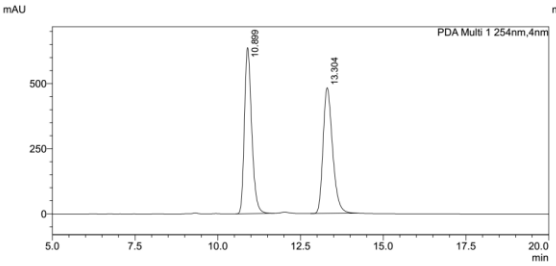

<Peak Table>
PDA Ch1 254nm

Peak#	Ret. Time	Area	Height	Height%	Area%
1	10.899	9746549	638031	56.927	50.162
2	13.304	9683463	482748	43.073	49.838
Total		19430013	1120779	100.000	100.000

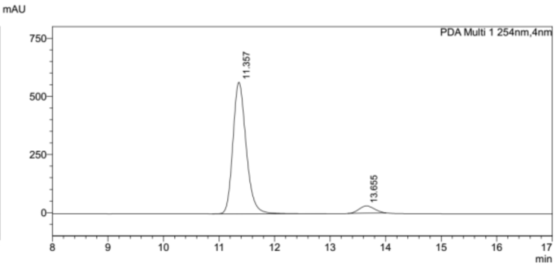

<Peak Table>
PDA Ch1 254nm

Peak#	Ret. Time	Area	Height	Height%	Area%
1	11.357	9380297	565990	94.798	93.986
2	13.655	600177	31060	5.202	6.014
Total		9980474	597050	100.000	100.000

8.7 (*Sa,Sp*)-4-(1-(2-(naphthalen-2-ylmethoxy))naphthyl)naphtho[1,2-a]ferrocene. ((*Sa,Sp*)-2af)

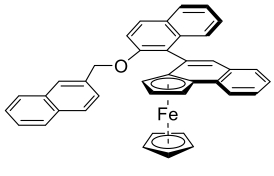

Isolated (-)-**2af** in 85% yield, 6:1 d.r., as pink solid.

^{1}H NMR (500 MHz, $CDCl_3$) δ 8.10 (d, *J* = 7.5 Hz, 1H), 7.97 – 7.87 (m, 2H), 7.85 – 7.74 (m, 3H), 7.63 (dd, *J* = 27.0, 8.0 Hz, 2H), 7.57 – 7.38 (m, 6H), 7.37 –7.27 (m, 3H), 7.23 – 7.16 (m, 1H), 5.53 (s, 2H), 5.35 (s, 1H), 4.21 (s, 1H), 4.12 (s, 1H), 3.74 (s, 5H); **^{13}C NMR** (125 MHz, $CDCl_3$) δ 153.3, 134.9, 134.7, 134.7, 133.5, 133.2, 132.9, 132.4, 129.4, 129.2, 128.7, 128.2, 127.9, 127.8, 127.6, 127.5, 126.6, 126.2, 126.1, 126.1, 125.9, 125.9, 125.2, 124.9, 123.7, 123.2, 115.3, 86.7, 83.0, 71.2, 69.3, 69.2, 64.6, 61.1; **HRMS** (ESI) calculated for [$C_{39}H_{28}FeNaO$] [M+Na]$^+$: 591.1382, found: 591.1387; **IR** (KBr, cm^{-1}) 3055, 2305, 1695, 1508, 1265, 1020, 750, 490; **[α]$_D^{20}$** = -1863.1 (c = 0.5, $CHCl_3$); **HPLC conditions**: Daicel Chiralpak ADH column (95:5 hexane: 2-propanol, 0.5 mL/min, 254 nm); tr (minor) = 14.6 min, tr (major) = 18.1 min; 88% ee.

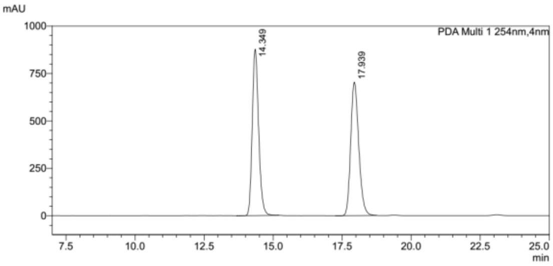

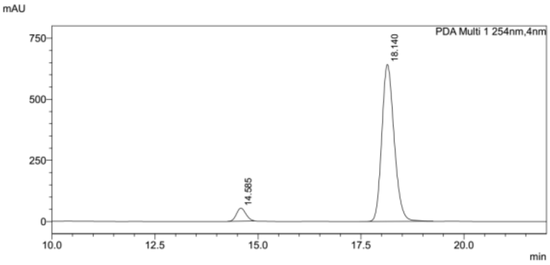

<Peak Table>
PDA Ch1 254nm

Peak#	Ret. Time	Area	Height	Height%	Area%
1	14.349	14059278	877270	55.470	49.583
2	17.939	14296028	704261	44.530	50.417
Total		28355306	1581531	100.000	100.000

<Peak Table>
PDA Ch1 254nm

Peak#	Ret. Time	Area	Height	Height%	Area%
1	14.585	853217	53394	7.680	5.987
2	18.140	13398403	641836	92.320	94.013
Total		14251620	695231	100.000	100.000

8.8 (*Sa,Sp*)-4-(1-(2-benzyloxy)naphthyl)naphtho[1,2-a]ferrocene. ((*Sa,Sp*)-2ag)

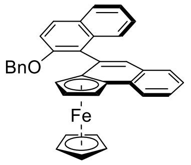

Isolated (-)-**2ag** in 78% yield, 10:1 d.r., as pink solid.

^{1}H NMR (400 MHz, $CDCl_3$) δ 8.07 (d, J = 7.6 Hz, 1H), 7.93 (d, J = 9.0 Hz, 1H), 7.83 (d, J = 8.0 Hz, 1H), 7.66 (d, J = 7.6 Hz, 1H), 7.57 – 7.49 (m, 2H), 7.47 – 7.40 (m, 3H), 7.32 – 7.25 (m, 5H), 7.24 – 7.13 (m, 2H), 5.37 (s, 2H), 5.32 (d, J = 1.2 Hz, 1H), 4.16 (s, 1H), 4.11 (t, J = 2.4 Hz, 1H), 3.71 (s, 5H); **^{13}C NMR** (100 MHz, $CDCl_3$) δ 153.3, 137.2, 134.8, 134.7, 133.6, 132.4, 129.3, 129.2, 128.6, 128.4, 127.8, 127.4, 126.4, 126.2, 126.1, 125.2, 123.8, 123.7, 123.2, 115.4, 86.7, 82.9, 71.4, 69.4, 69.2, 64.6, 61.0; **HRMS** (ESI) calculated for [$C_{35}H_{26}FeNaO$] $[M+Na]^+$: 541.1226, found: 541.1234; **IR** (KBr, cm^{-1}) 2924, 2320, 1700, 1508, 1265, 1055, 750, 495; **2ag-[α]$_D^{20}$** = -2492.3 (c = 0.1, $CHCl_3$); *ent*-**2ag-[α]$_D^{20}$** = 2790.7 (c = 0.1, $CHCl_3$); **HPLC conditions**: Daicel Chiralpak ADH column (95:5 hexane: 2-propanol, 0.5 mL/min, 254 nm); **2ag** [tr (minor) = 14.1 min, tr (major) = 11.4 min; 91% ee]; *ent*-**2ag** [tr (minor) = 11.5 min, tr (major) = 14.2 min; 92% ee].

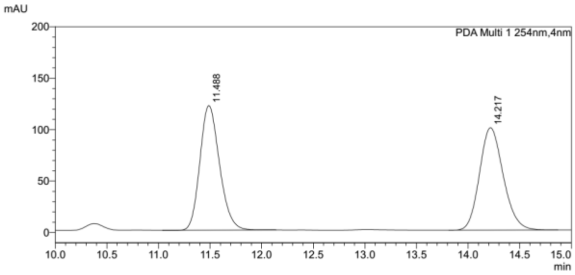

<Peak Table>
PDA Ch1 254nm

Peak#	Ret. Time	Area	Height	Height%	Area%
1	11.488	1598818	121253	54.906	50.077
2	14.217	1593894	99586	45.094	49.923
Total		3192712	220839	100.000	100.000

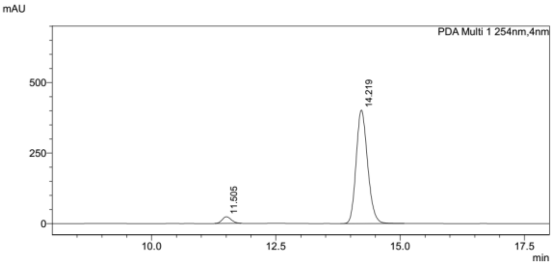

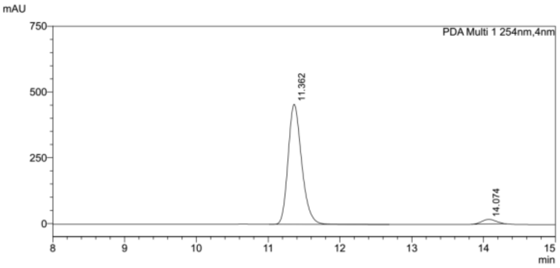

<Peak Table>
PDA Ch1 254nm

Peak#	Ret. Time	Area	Height	Height%	Area%
1	11.505	300938	23536	5.524	4.461
2	14.219	6445633	402525	94.476	95.539
Total		6746571	426061	100.000	100.000

<Peak Table>
PDA Ch1 254nm

Peak#	Ret. Time	Area	Height	Height%	Area%
1	11.362	6010646	456987	96.223	95.986
2	14.074	251388	17940	3.777	4.014
Total		6262034	474927	100.000	100.000

8.9 (*Sa,Sp*)-4-(1-(2-cyclopropylmethoxy)naphthyl)naphtho[1,2-a]ferrocene. ((*Sa,Sp*)-2ah)

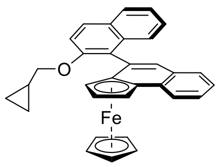

Isolated (-)-**2ah** in 90% yield, 10:1 d.r., as pink solid.

^{1}H NMR (400 MHz, $CDCl_3$) δ 8.09 (d, *J* = 7.6 Hz, 1H), 7.92 (d, *J* = 8.8 Hz, 1H), 7.82 (d, *J* = 8.4 Hz, 1H), 7.67 (d, *J* = 7.4 Hz, 1H), 7.54 – 7.47 (m, 2H), 7.47 – 7.41 (m, 1H), 7.31 – 7.27 (m, 2H), 7.25 (d, *J* = 1.6 Hz, 1H), 7.19 – 7.13 (m, 1H), 5.35 – 5.32 (m, 1H), 4.20 – 4.14 (m, 2H), 4.12 (t, *J* = 2.4 Hz, 1H), 4.03 – 3.96 (m, 1H), 3.83 (s, 5H), 1.27 – 1.26 (m, 1H), 0.57 – 0.51 (m, 2H), 0.34 – 0.51 (m, 2H); **^{13}C NMR** (100 MHz, $CDCl_3$) δ 153.8, 134.9, 134.8, 133.6, 132.7, 129.2, 129.0, 128.6, 127.8, 127.1, 126.4, 126.2, 126.0, 125.2, 123.7, 123.6, 123.2, 115.5, 86.8, 82.9, 74.7, 69.7, 69.3, 64.8, 61.1, 10.8, 3.9, 3.4; **HRMS** (ESI) calculated for [$C_{32}H_{26}FeNaO$] $[M+Na]^+$: 505.1226, found: 505.1231; **IR** (KBr, cm^{-1}) 2924, 2310, 1744, 1508, 1263, 1044, 750, 500 ; **$[\alpha]_D^{20}$** = -2699.9 (c = 0.1, $CHCl_3$); **HPLC conditions**: Daicel Chiralpak ADH column (98:2 hexane: 2-propanol, 0.5 mL/min, 254 nm); tr (minor) = 12.0 min, tr (major) = 12.8 min; 91% ee.

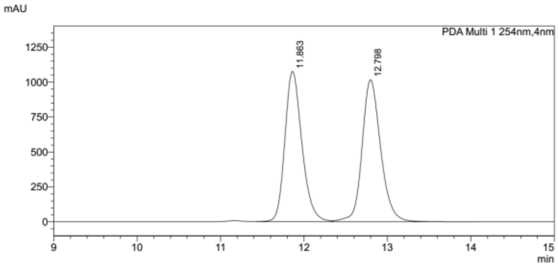

<Peak Table>
PDA Ch1 254nm

Peak#	Ret. Time	Area	Height	Height%	Area%
1	11.863	15322110	1076169	51.414	49.260
2	12.798	15782755	1016963	48.586	50.740
Total		31104865	2093131	100.000	100.000

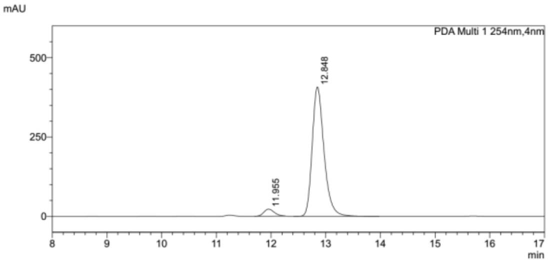

<Peak Table>
PDA Ch1 254nm

Peak#	Ret. Time	Area	Height	Height%	Area%
1	11.955	291711	22242	5.182	4.553
2	12.848	6115685	406979	94.818	95.447
Total		6407396	429222	100.000	100.000

8.10 (*Sa,Sp*)-4-(1-(2-cyclobutylmethoxy)naphthyl)naphtho[1,2-a]ferrocene. ((*Sa,Sp*)-2ai)

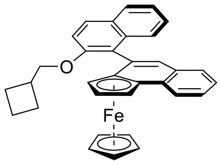

Isolated (-)-**2ai** in 91% yield, >20:1 d.r., as pink solid.

^{1}H NMR (400 MHz, $CDCl_3$) δ 8.08 (d, *J* = 8.0 Hz, 1H), 7.92 (d, *J* = 9.2 Hz, 1H), 7.82 (d, *J* = 8.4 Hz, 1H), 7.66 (d, *J* = 7.6 Hz, 1H), 7.52 – 7.49 (m, 2H), 7.44 (t, *J* = 7.6 Hz, 1H), 7.30 – 7.22 (m, 3H), 7.18 – 7.14 (m, 1H), 5.33 (d, *J* = 1.2 Hz, 1H), 4.31 – 4.27 (m, 1H), 4.13 – 4.10 (m, 2H), 3.94 (t, *J* = 8.8 Hz, 1H), 3.79 (s, 5H), 2.44 – 2.33 (m, 1H), 1.84 – 1.72 (m, 2H), 1.63 – 1.60 (m, 2H), 1.55 – 1.47 (m, 2H); **^{13}C NMR** (100 MHz, $CDCl_3$) δ 153.9, 134.7, 134.6, 133.5, 132.6, 129.2, 128.9, 128.6, 127.8, 127.5, 126.4, 126.2, 125.9, 125.2, 123.4, 123.2, 123.0, 115.0, 86.6, 83.0, 74.0, 69.5, 69.2, 64.8, 61.1, 39.3, 30.1, 29.3, 25.5, 25.4; **HRMS** (ESI) calculated for [$C_{33}H_{28}FeNaO$] $[M+Na]^+$: 519.1382, found: 519.1393; **IR** (KBr, cm^{-1}) 2924, 2320, 1748, 1508, 1260, 1020, 750, 490; **$[\alpha]_D^{20}$** = -3014.1 (c = 0.1, $CHCl_3$); **HPLC conditions**: Daicel Chiralpak ADH column (98:2 hexane: 2-propanol, 0.5 mL/min, 254 nm); tr (minor) = 9.0 min, tr (major) = 11.3 min; 91% ee.

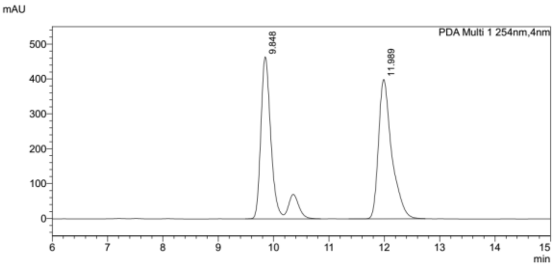

<Peak Table>
PDA Ch1 254nm

Peak#	Ret. Time	Area	Height	Height%	Area%
1	9.848	6641787	464725	53.764	50.065
2	11.989	6624417	399650	46.236	49.935
Total		13266204	864375	100.000	100.000

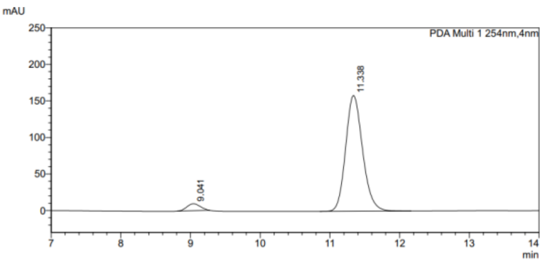

<Peak Table>
PDA Ch1 254nm

Peak#	Ret. Time	Area	Height	Height%	Area%
1	9.041	127357	9531	5.671	4.532
2	11.338	2682691	158549	94.329	95.468
Total		2810048	168081	100.000	100.000

8.11 (*Sa,Sp*)-4-(1-(2-cycloamylmethoxy)naphthyl)naphtho[1,2-a]ferrocene. ((*Sa,Sp*)-2aj)

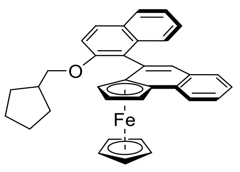

Isolated (-)-**2aj** in 92% yield, >20:1 d.r., as pink solid.

^{1}H NMR (400 MHz, $CDCl_3$) δ 8.08 (d, *J* = 7.8 Hz, 1H), 7.92 (d, *J* = 8.8 Hz, 1H), 7.82 (d, *J* = 8.8 Hz, 1H), 7.66 (d, *J* = 7.6 Hz, 1H), 7.52 – 7.49 (m, 2H), 7.46 – 7.42 (m, 1H), 7.29 – 7.26 (m, 3H), 7.18 – 7.13 (m, 1H), 5.33 (d, *J* = 1.2 Hz, 1H), 4.31 – 4.27 (m, 1H), 4.13 – 4.09 (m, 2H), 3.93 (t, *J* = 8.4 Hz, 1H), 3.79 (s, 5H), 2.44 – 2.31 (m, 1H), 1.81 – 1.72 (m, 2H), 1.62 – 1.59 (m, 2H), 1.52 – 1.44 (m, 2H), 1.35– 1.30 (m, 2H); **^{13}C NMR** (100 MHz, $CDCl_3$) δ 153.9, 134.7, 134.6, 133.5, 132.6, 129.2, 128.9, 128.5, 127.8, 127.5, 126.4, 126.2, 125.9, 125.2, 123.4, 123.2, 123.0, 115.0, 86.6, 83.0, 74.0, 69.5, 69.2, 64.8, 61.1, 39.3, 30.1, 29.3, 25. 5, 25.4; **HRMS** (ESI) calculated for [$C_{34}H_{30}FeNaO$] $[M+Na]^+$: 533.1539, found: 533.1540; **IR** (KBr, cm^{-1}) 2953, 2320, 1744, 1508, 1265, 1055, 750, 500; **$[\alpha]_D^{20}$** = -2522.9 (c = 0.1, $CHCl_3$); **HPLC conditions**: Daicel Chiralpak ADH column (98:2 hexane: 2-propanol, 0.5 mL/min, 254 nm); tr (minor) = 9.8 min, tr (major) = 11.8 min; 87% ee.

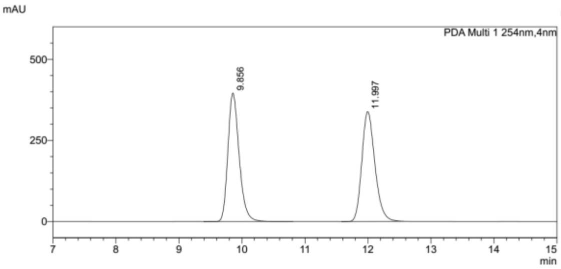

<Peak Table>
PDA Ch1 254nm

Peak#	Ret. Time	Area	Height	Height%	Area%
1	9.856	4911975	396319	53.935	50.146
2	11.997	4883402	338494	46.065	49.854
Total		9795377	734813	100.000	100.000

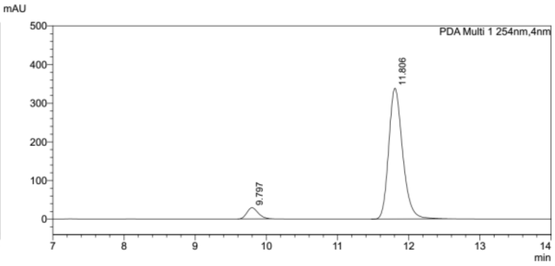

<Peak Table>
PDA Ch1 254nm

Peak#	Ret. Time	Area	Height	Height%	Area%
1	9.797	324003	29126	7.915	6.570
2	11.806	4607857	338842	92.085	93.430
Total		4931860	367968	100.000	100.000

8.12 (*Sa,Sp*)-4-(1-(2-(2-ethylbutoxy))naphthyl)naphtho[1,2-a]ferrocene. ((*Sa,Sp*)-2ak)

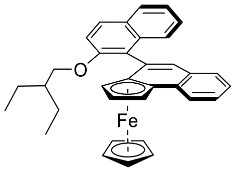

Isolated (-)-**2ak** in 90% yield, >20:1 d.r., as pink solid.

^{1}H NMR (400 MHz, $CDCl_3$) δ 8.07 (d, *J* = 7.6 Hz, 1H), 7.93 (d, *J* = 8.8 Hz, 1H), 7.82 (d, *J* = 8.0 Hz, 1H), 7.64 (d, *J* = 7.6 Hz, 1H), 7.54 – 7.48 (m, 2H), 7.43 (t, *J* = 7.6 Hz, 1H), 7.34 – 7.26 (m, 3H), 7.20 – 7.13 (m, 1H), 5.32 (s, 1H), 4.28 – 4.24 (m, 1H), 4.12 (d, *J* = 14.0 Hz, 2H), 3.99 (t, *J* = 8.8 Hz, 1H), 3.79 (s, 5H), 1.77 – 1.67 (m, 1H), 1.59 – 1.50 (m, 1H), 1.49 – 1.38 (m, 3H), 0.95 – 0.85 (m, 6H); **^{13}C NMR** (100 MHz, $CDCl_3$) δ 153.9, 134.7, 134.5, 133.4, 132.5, 129.2, 128.9, 128.5, 127.8, 127.5, 126.4, 126.2, 125.9, 125.2, 123.4, 123.2, 123.1, 114.9, 86.6, 83.0, 71.6, 69.4, 69.2, 64.9, 61.2, 41.0, 23.1, 11.4, 10.5; **HRMS** (ESI) calculated for [$C_{34}H_{32}FeNaO$] $[M+Na]^+$: 535.1695, found: 535.1697; **IR** (KBr, cm^{-1}) 2961, 2320, 1744, 1508, 1265, 1022, 750, 490; **$[\alpha]_D^{20}$** = -2355.1 (c = 0.1, $CHCl_3$); **HPLC conditions**: Daicel Chiralpak ADH column (98:2 hexane: 2-propanol, 0.5 mL/min, 254 nm); tr (minor) = 8.6 min, tr (major) = 9.6 min; 88% ee.

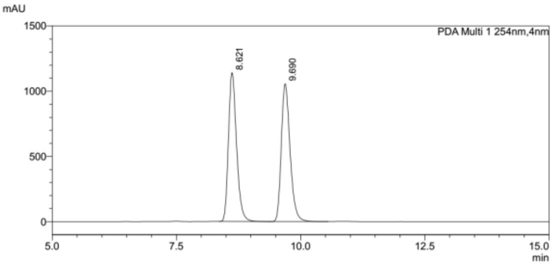

<Peak Table>
PDA Ch1 254nm

Peak#	Ret. Time	Area	Height	Height%	Area%
1	8.621	12985068	1142168	51.933	49.927
2	9.690	13022800	1057127	48.067	50.073
Total		26007868	2199295	100.000	100.000

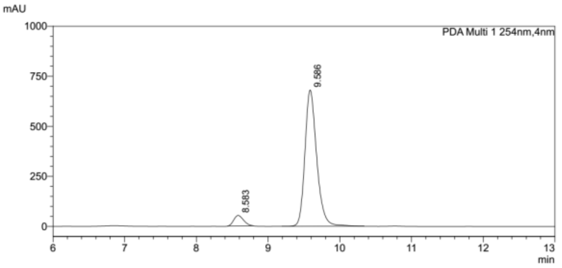

<Peak Table>
PDA Ch1 254nm

Peak#	Ret. Time	Area	Height	Height%	Area%
1	8.583	499614	52677	7.179	6.039
2	9.586	7773056	681044	92.821	93.961
Total		8272671	733721	100.000	100.000

8.13 (*Sa,Sp*)-4-(1-(2-cyclohexylmethoxy)naphthyl)naphtho[1,2-a]ferrocene. ((*Sa,Sp*)-2al)

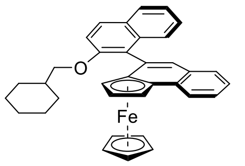

Isolated (-)-**2al** in 91% yield, >20:1 d.r., as pink solid.

^{1}H NMR (500 MHz, THF-*d$_8$*) δ 8.11 (d, *J* = 8.0 Hz, 1H), 7.94 (d, *J* = 9.5 Hz, 1H), 7.81 (d, *J* = 8.5 Hz, 1H), 7.64 (d, *J* = 7.5 Hz, 1H), 7.60 (d, *J* = 9.0 Hz, 1H), 7.47 (t, *J* = 7.0 Hz, 1H), 7.40 (t, *J* = 7.0 Hz, 1H), 7.27 – 7.22 (m, 3H), 7.12 (t, *J* = 7.5 Hz, 1H), 5.38 (s, 1H), 4.25 – 4.22 (m, 1H), 4.11 – 4.06 (m, 2H), 3.98 – 3.91 (m, 1H), 3.76 (s, 5H), 2.01 (d, *J* = 11.0 Hz, 1H), 1.83 (d, *J* = 10.5 Hz, 2H), 1.70 – 1.57 (m, 3H), 1.24 – 1.09 (m, 5H); **^{13}C NMR** (125 MHz, THF-*d$_8$*) δ 154.8, 135.6 135.4, 134.3, 133.3, 129.9, 129.9, 129.0, 128.4, 128.3, 126.9, 126.7, 126.3, 125.7, 123.9, 123.9, 123.8, 115.9, 87.3, 83.9, 75.5, 70.0, 69.7, 65.3, 61.8, 38.9, 31.0, 30.5, 27.2, 26.5, 26.3; **HRMS** (ESI) calculated for [$C_{35}H_{30}FeNaO$] [M+Na]$^+$: 547.1695, found: 547.1702; **IR** (KBr, cm^{-1}) 2924, 2320, 1690, 1508, 1263, 1020, 750, 490; **2al-[α]$_D$^{20}** = -2378.3 (c = 0.1, $CHCl_3$); *ent*-**2al-[α]$_D$^{20}** = 2550.8 (c = 0.1, $CHCl_3$); **HPLC conditions**: Daicel Chiralpak ADH column (98:2 hexane: 2-propanol, 0.5 mL/min, 254 nm); **2al** [tr (minor) = 9.9 min, tr (major) = 11.1 min; 93% ee]; *ent*-**2al** [tr (minor) = 11.1 min, tr (major) = 9.9 min; 91% ee].

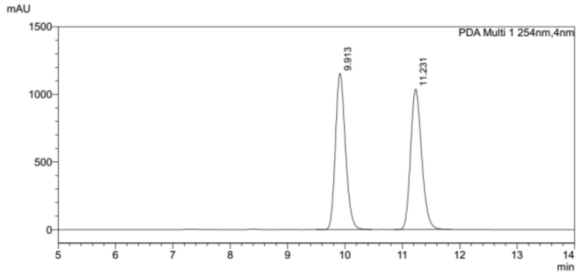

<Peak Table>
PDA Ch1 254nm

Peak#	Ret. Time	Area	Height	Height%	Area%
1	9.913	13939742	1154087	52.617	49.988
2	11.231	13946648	1039282	47.383	50.012
Total		27886390	2193369	100.000	100.000

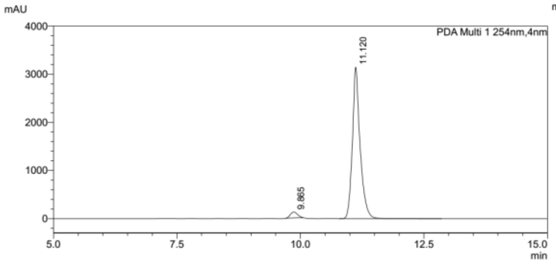

<Peak Table>
PDA Ch1 254nm

Peak#	Ret. Time	Area	Height	Height%	Area%
1	9.865	1244346	122484	3.750	3.528
2	11.120	34028361	3144050	96.250	96.472
Total		35272706	3266534	100.000	100.000

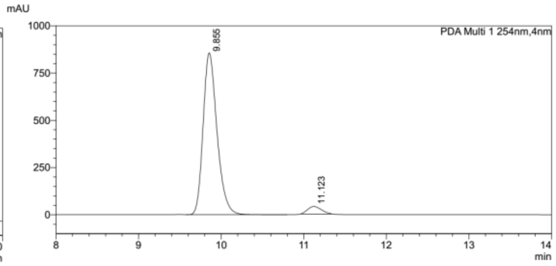

<Peak Table>
PDA Ch1 254nm

Peak#	Ret. Time	Area	Height	Height%	Area%
1	9.855	10027891	857462	95.528	95.561
2	11.123	465786	40145	4.472	4.439
Total		10493677	897607	100.000	100.000

8.14 (*Sa,Sp*)-4-(1-(2-cyclohexylmethoxy)naphthyl)-7-fluoro-naphtho[1,2-a]ferrocene. ((*Sa,Sp*)-2am)

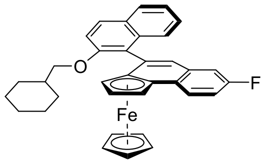

Isolated (-)-**2am** in 89% yield, >20:1 d.r., as pink solid.

^{1}H NMR (400 MHz, $CDCl_3$) δ 8.03 – 8.01 (m, 1H), 7.93 (d, *J* = 9.2 Hz, 1H), 7.82 (d, *J* = 8.6 Hz, 1H), 7.51 (d, *J* = 9.2 Hz, 1H), 7.33 – 7.27 (m, 2H), 7.24– 7.23 (m, 2H), 7.20 (s, 1H), 7.19 – 7.14 (m, 1H), 5.31– 5.29 (m, 1H), 4.19 (dd, *J* = 9.0, 5.2 Hz, 1H), 4.15 – 4.12 (m, 1H), 4.10 (t, *J* = 2.4 Hz, 1H), 3.90 (t, *J* = 8.4 Hz, 1H), 3.79 (s, 5H), 1.96 (d, *J* = 12.8 Hz, 1H), 1.84 – 1.77 (m, 2H), 1.72 – 1.64 (m, 3H), 1.20 – 1.05 (m, 5H); **^{13}C NMR** (100 MHz, $CDCl_3$) δ 160.9 (d, *J* = 242.7 Hz), 153.9, 136.6, 133.9 (d, *J* = 8.2 Hz), 133.3, 130.7 (d, *J* = 2.1 Hz), 129.4, 128.8, 127.8, 126.7 (d, *J* = 3.1 Hz), 126.0, 124.7 (d, *J* = 8.4 Hz), 123.5, 122.6, 114.9, 114.3 (d, *J* = 23.0 Hz), 113.4 (d, *J* = 21.0 Hz), 86.0, 83.0, 74.9, 69.5, 69.5, 69.3, 64. 9, 61.0, 38.0, 30.3, 29.8, 26.5, 25.7, 25.5; **HRMS** (ESI) calculated for [$C_{35}H_{31}FFeNaO$] [M+Na]$^+$: 565.1601, found: 565.1599; **IR** (KBr, cm^{-1}) 2924, 2320, 1750, 1508, 1261, 1020, 750, 500; **[α]$_D$^{20}** = -2771.7 (c = 0.1, $CHCl_3$); **HPLC conditions**: Daicel Chiralpak ODH column (90:10 hexane: 2-propanol,

0.8 mL/min, 254 nm); tr (minor) = 5.8 min, tr (major) = 5.1 min; 92% ee.

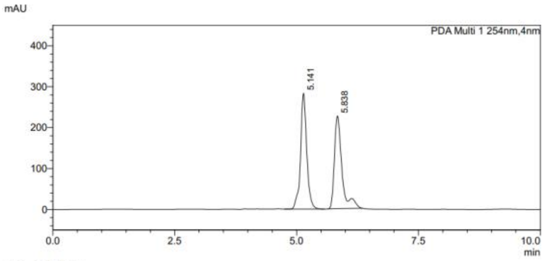

<Peak Table>
PDA Ch1 254nm

Peak#	Ret. Time	Area	Height	Height%	Area%
1	5.141	2516544	283670	55.553	50.657
2	5.838	2451277	226959	44.447	49.343
Total		4967821	510629	100.000	100.000

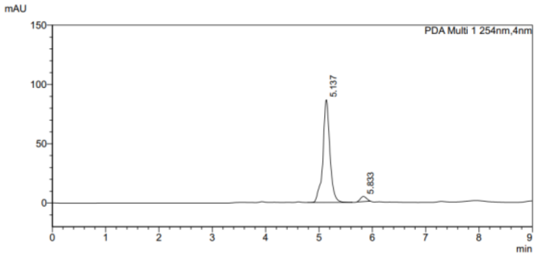

<Peak Table>
PDA Ch1 254nm

Peak#	Ret. Time	Area	Height	Height%	Area%
1	5.137	789090	86520	95.320	95.852
2	5.833	34147	4248	4.680	4.148
Total		823237	90768	100.000	100.000

8.15 (*Sa,Sp*)-4-(1-(2-cyclohexylmethoxy)naphthyl)-7-chloro-naphtho[1,2-a]ferrocene. ((*Sa,Sp*)-2an)

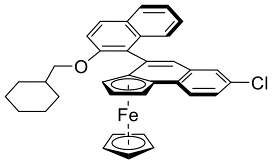

Isolated (-)-**2an** in 91% yield, >20:1 d.r., as pink solid.

^{1}H NMR (400 MHz, $CDCl_3$) δ 7.99 (d, *J* = 8.4 Hz, 1H), 7.93 (d, *J* = 8.8 Hz, 1H), 7.82 (d, *J* = 8.0 Hz, 1H), 7.61 (d, *J* = 2.0 Hz, 1H), 7.50 (d, *J* = 9.2 Hz, 1H), 7.47– 7.44 (m, 1H), 7.29 (d, *J* = 6.8 Hz, 1H), 7.24 – 7.21 (m, 1H), 7.18– 7.16 (m, 2H), 5.32 – 5.29 (m, 1H), 4.20 – 4.15 (m, 2H), 4.14 – 4.12 (m, 1H), 3.92 – 3.86 (m, 1H), 3.79 (s, 5H), 1.95 (d, *J* = 12.8 Hz, 1H), 1.85 – 1.75 (m, 2H), 1.68 – 1.63 (m, 3H), 1.20– 1.04 (m, 5H); **^{13}C NMR** (100 MHz, $CDCl_3$) δ 153.9, 136.6, 133.8, 133.3, 133.2, 130.6, 129.5, 128.8, 127.8, 127.6, 126.6, 126.4, 126.0, 125.9, 124.5, 123.5, 122.6, 114.9, 86.3, 82.4, 74.9, 69.6, 69.6, 65.1, 61.3, 37.9, 30.3, 29.9, 26.5, 25.7, 25.5; **HRMS** (ESI) calculated for $[C_{35}H_{31}ClFeNaO]$ $[M+Na]^+$: 581.1306, found: 581.1300; **IR** (KBr, cm^{-1}) 2924, 2320, 1748, 1508, 1263, 1086, 750, 500; **$[\alpha]_D^{20}$** = -2732.8 (c = 0.1, $CHCl_3$); **HPLC conditions**: Daicel Chiralpak ODH column (95:5 hexane: 2-propanol, 0.5 mL/min, 254 nm); tr (minor) = 10.4 min, tr (major) = 8.9 min; 88% ee.

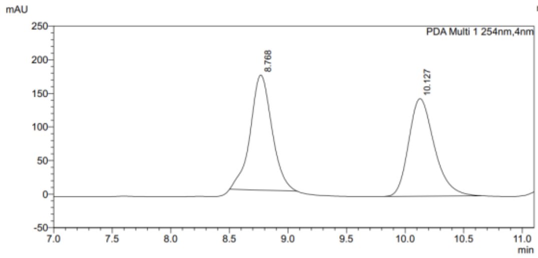

<Peak Table>
PDA Ch1 254nm

Peak#	Ret. Time	Area	Height	Height%	Area%
1	8.768	2216986	171729	54.185	50.044
2	10.127	2213105	145205	45.815	49.956
Total		4430091	316934	100.000	100.000

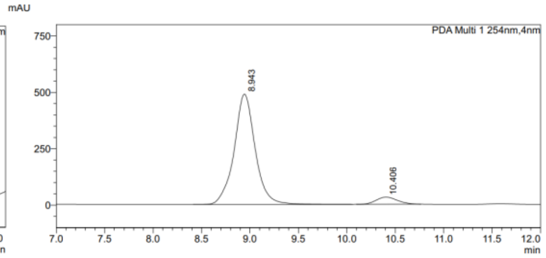

<Peak Table>
PDA Ch1 254nm

Peak#	Ret. Time	Area	Height	Height%	Area%
1	8.943	7402785	488889	93.910	93.793
2	10.406	489884	31703	6.090	6.207
Total		7892669	520592	100.000	100.000

8.16 (*Sa,Sp*)-4-(1-(2-cyclohexylmethoxy)naphthyl)-7-bromo-naphtho[1,2-a]ferrocene. ((*Sa,Sp*)-2ao)

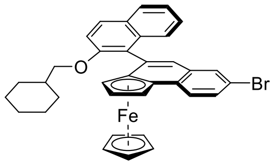

Isolated (-)-**2ao** in 90% yield, >20:1 d.r., as pink solid.

^{1}H NMR (400 MHz, $CDCl_3$) δ 7.93 (d, *J* = 8.8 Hz, 2H), 7.82 (d, *J* = 8.2 Hz, 1H), 7.77 (d, *J* = 1.8 Hz, 1H), 7.60 – 757 (m, 1H), 7.51 (d, *J* = 9.2 Hz, 1H), 7.31 – 7.26 (m, 1H), 7.24 – 7.20 (m, 1H), 7.19 – 7.14 (m, 2H), 5.33 – 5.29 (m, 1H), 4.21 – 4.17 (m, 1H), 4.17 – 4.12 (m, 2H), 3.90 (t, *J* = 8.4 Hz, 1H), 3.80 (s, 5H), 1.95 (d, *J* = 12.8 Hz, 1H), 1.85 – 1.76 (m, 2H), 1.74 – 1.64 (m, 3H), 1.22 – 1.04 (m, 5H); **^{13}C NMR** (100 MHz, $CDCl_3$) δ 153.9, 136.5, 134.1, 133.6, 133.3, 130.6, 129.5, 129.3, 128.8, 127.8, 126.2, 126.0, 125.9, 124.7, 123.5, 122.5, 118.6, 114.9, 86.3, 82.3, 74.9, 69.7, 69.6, 65.1, 61.3, 37.9, 30.3, 29.8, 26.5, 25.7, 25.5; **HRMS** (ESI) calculated for $[C_{35}H_{31}BrFeNaO]$ $[M+Na]^+$: 625.0802, found: 625.0802; **IR** (KBr, cm^{-1}) 2924, 2312, 1750, 1502, 1261, 1049, 750, 490; **$[\alpha]_D^{20}$** = -2185.9 (c = 0.1, $CHCl_3$); **HPLC conditions**: Daicel Chiralpak ODH column (90:10 hexane: 2-propanol, 0.8 mL/min, 254

nm); tr (minor) = 5.8 min, tr (major) = 5.3 min; 88% ee.

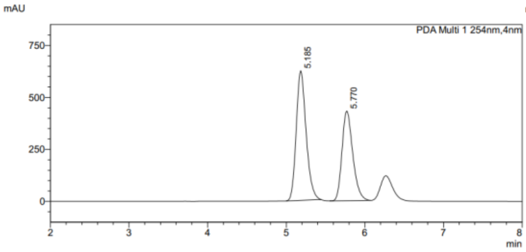

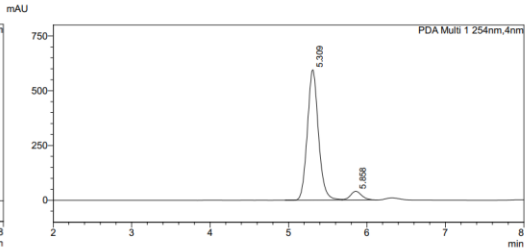

<Peak Table>
PDA Ch1 254nm

Peak#	Ret. Time	Area	Height	Height%	Area%
1	5.185	5449246	623060	59.074	56.997
2	5.770	4111374	431656	40.926	43.003
Total		9560620	1054715	100.000	100.000

<Peak Table>
PDA Ch1 254nm

Peak#	Ret. Time	Area	Height	Height%	Area%
1	5.309	5846265	594853	93.783	93.759
2	5.858	389154	39436	6.217	6.241
Total		6235419	634289	100.000	100.000

8.17 (*Sa*,*Sp*)-4-(1-(2-cyclohexylmethoxy)naphthyl)-8-fluoro-naphtho[1,2-a]ferrocene. ((*Sa*,*Sp*)-2ap)

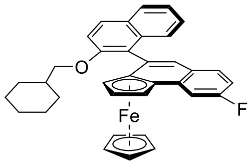

Isolated (-)-**2ap** in 91% yield, >20:1 d.r., as pink solid.

^{1}H NMR (400 MHz, $CDCl_3$) δ 7.92 (d, *J* = 9.0 Hz, 1H), 7.82 (d, *J* = 8.0 Hz, 1H), 7.73 – 7.69 (m, 1H), 7.62 – 7.58 (m, 1H), 7.50 (d, *J* = 8.8 Hz, 1H), 7.29 – 7.21 (m, 3H), 7.20 – 7.11 (m, 2H), 5.26 (s, 1H), 4.21 – 4.15 (m, 2H), 4.13 (d, *J* = 2.2 Hz, 1H), 3.88 (t, *J* = 8.4 Hz, 1H), 3.80 (s, 5H), 1.94 (d, *J* = 12.4 Hz, 1H), 1.85 – 1.75 (m, 2H), 1.70– 1.60 (m, 3H), 1.21 – 1.03 (m, 5H); **^{13}C NMR** (100 MHz, $CDCl_3$) δ 161.6 (d, *J* = 245.3 Hz), 153.9, 136.8 (d, *J* = 8.7 Hz), 133.5 (d, *J* = 2.9 Hz), 133.4, 130.1 (d, *J* = 8.8 Hz), 129.3, 128.9, 128.8, 127.8, 126.8, 126.0 (d, *J* = 8.5 Hz), 123.4, 122.8, 115.0, 113.2 (d, *J* = 22.9 Hz), 108.7 (d, *J* = 21.9 Hz), 86.8, 82.2 (d, *J* = 3.4 Hz), 74.9, 69.5, 65.1, 61.4, 38.0, 30.3, 29.8, 26.5, 25.8, 25.5; **HRMS** (ESI) calculated for $[C_{35}H_{31}FFeNaO]$ $[M+Na]^+$: 565.1601, found: 565.1616; **IR** (KBr, cm^{-1}) 2924, 2320, 1748, 1508, 1261, 1022, 750, 500; **$[\alpha]_D^{20}$** = -2016.5 (c = 0.1, $CHCl_3$); **HPLC conditions**: Daicel Chiralpak ADH column (98:2 hexane: 2-propanol, 0.5 mL/min, 254 nm); tr (minor) = 10.2 min, tr (major) = 11.1 min; 92% ee.

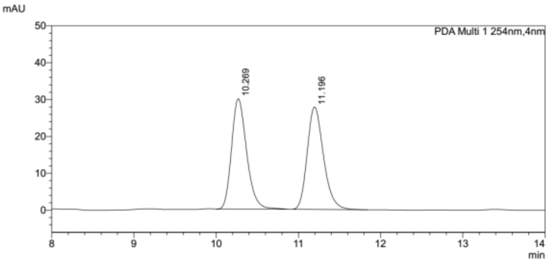

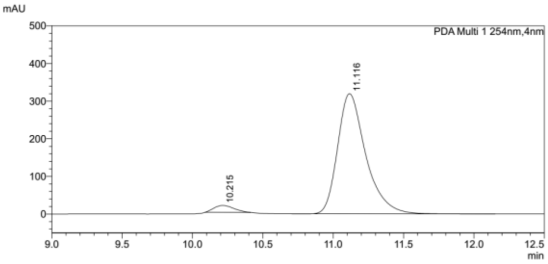

<Peak Table>
PDA Ch1 254nm

Peak#	Ret. Time	Area	Height	Height%	Area%
1	10.269	384248	29975	51.859	50.018
2	11.196	383967	27826	48.141	49.982
Total		768215	57801	100.000	100.000

<Peak Table>
PDA Ch1 254nm

Peak#	Ret. Time	Area	Height	Height%	Area%
1	10.215	179957	18274	5.402	3.878
2	11.116	4459973	320031	94.598	96.122
Total		4639931	338305	100.000	100.000

8.18 (*Sa*,*Sp*)-4-(1-(2-cyclohexylmethoxy)naphthyl)-8-chloro-naphtho[1,2-a]ferrocene. ((*Sa*,*Sp*)-2aq)

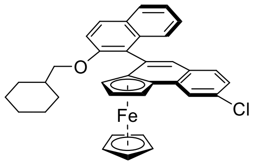

Isolated (-)-**2aq** in 90% yield, >20:1 d.r., as pink solid.

^{1}H NMR (400 MHz, $CDCl_3$) δ 8.03 (s, 1H), 7.92 (d, *J* = 8.0 Hz, 1H), 7.82 (d, *J* = 6.8 Hz, 1H), 7.56 – 7.48 (m, 2H), 7.37 (d, *J* = 6.4 Hz, 1H), 7.30 – 7.13 (m, 4H), 5.29 (s, 1H), 4.17 (s, 2H), 4.14 (s, 1H), 3.87 (d, *J* = 8.8 Hz, 1H), 3.81 (s, 5H), 1.94 (d, *J* = 9.2 Hz, 1H), 1.79 (s, 2H), 1.65 (d, *J* = 12 Hz, 3H), 1.24 – 1.03 (m, 5H); **^{13}C NMR** (100 MHz, $CDCl_3$) δ 153.9, 136.4, 135.1, 133.3, 131.9, 130.8, 129.6, 129.4, 128.8, 127.8, 126.7, 126.0, 125.5, 123.5, 122.7, 122.7, 114.9, 86.7, 81.9, 74.8, 69.7, 69.6, 65.1, 61.3, 38.0, 30.3, 29.8, 26.5, 25.7, 25.5; **HRMS** (ESI) calculated for $[C_{35}H_{31}ClFeNaO]$ $[M+Na]^+$: 581.1306, found: 581.1306; **IR** (KBr, cm^{-1}) 2922, 2320, 1750, 1508, 1261, 1020, 750, 501; **$[\alpha]_D^{20}$** = -2727.1 (c = 0.1, $CHCl_3$); **HPLC conditions**: Daicel Chiralpak ADH column (98:2 hexane: 2-propanol, 0.5 mL/min, 254 nm); tr (minor) = 10.1 min, tr (major) = 11.8 min; 92% ee.

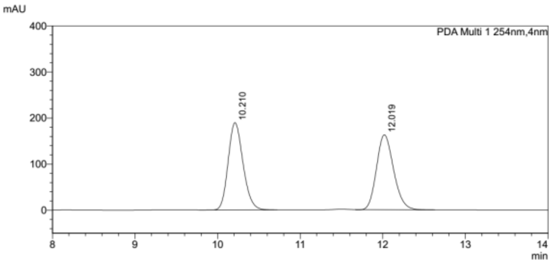

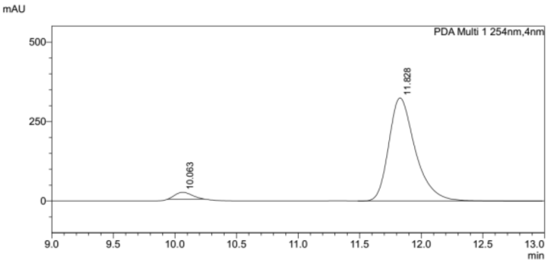

<Peak Table>
PDA Ch1 254nm

Peak#	Ret. Time	Area	Height	Height%	Area%
1	10.210	2376650	189691	53.793	50.269
2	12.019	2351217	162938	46.207	49.731
Total		4727867	352630	100.000	100.000

<Peak Table>
PDA Ch1 254nm

Peak#	Ret. Time	Area	Height	Height%	Area%
1	10.063	202760	21375	6.180	4.011
2	11.828	4851994	324472	93.820	95.989
Total		5054754	345847	100.000	100.000

8.19 (*Sa,Sp*)-4-(1-(2-cyclohexylmethoxy)naphthyl)-8-bromo-naphtho[1,2-a]ferrocene. ((*Sa,Sp*)-2ar)

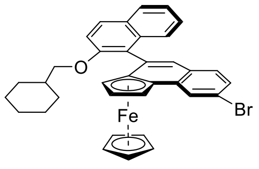

Isolated (-)-**2ar** in 90% yield, >20:1 d.r., as pink solid.

^{1}H NMR (400 MHz, $CDCl_3$) δ 8.20 (s, 1H), 7.93 (d, *J* = 9.2 Hz, 1H), 7.83 (d, *J* = 8.0 Hz, 1H), 7.54 – 7.48 (m, 3H), 7.31 – 7.27 (m, 1H), 7.24 (d, *J* = 3.2 Hz, 1H), 7.19 – 7.16 (m, 2H), 5.30 (d, *J* = 1.2 Hz, 1H), 4.20 (d, *J* = 5.2 Hz, 1H), 4.16 – 4.14 (m, 2H), 3.89 (t, *J* = 8.4 Hz, 1H), 3.82 (s, 5H), 1.95 (d, *J* = 12.4 Hz, 1H), 1.84 – 1.76 (m, 2H), 1.72 – 1.62 (m, 3H), 1.19 – 1.03 (m, 5H); **^{13}C NMR** (100 MHz, $CDCl_3$) δ 153.9, 136.8, 135.4, 133.3, 131.1, 129.8, 129.4, 128.8, 128.2, 127.8, 126.7, 126.0, 126.0, 125.7, 123.5, 122.7, 120.1, 114.9, 86.6, 81.8, 74.8, 69.8, 69.6, 65.2, 61.3, 38.0, 30.3, 29.8, 26.5, 25.7, 25.5; **HRMS** (ESI) calculated for [$C_{35}H_{31}BrFeNaO$] [M+Na]+: 625.0802, found: 625.0819; **IR** (KBr, cm^{-1}) 2924, 2374, 1745, 1508, 1261, 1020, 750, 490; **$[\alpha]_D^{20}$** = -1241.8 (c = 0.1, $CHCl_3$); **HPLC conditions**: Daicel Chiralpak ADH column (98:2 hexane: 2-propanol, 0.5 mL/min, 254 nm); tr (minor) = 10.3 min, tr (major) = 12.6 min; 99% ee.

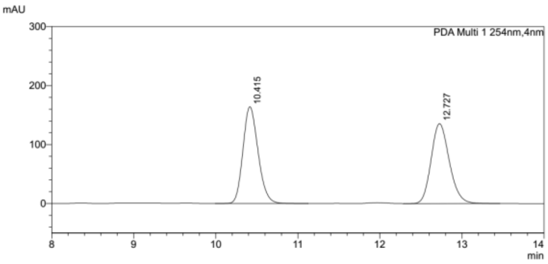

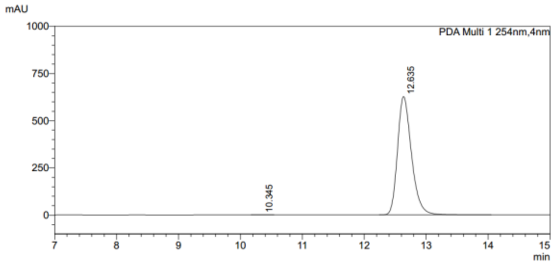

<Peak Table>
PDA Ch1 254nm

Peak#	Ret. Time	Area	Height	Height%	Area%
1	10.415	2106868	164049	54.713	50.151
2	12.727	2094152	135784	45.287	49.849
Total		4201020	299833	100.000	100.000

<Peak Table>
PDA Ch1 254nm

Peak#	Ret. Time	Area	Height	Height%	Area%
1	10.345	2265	218	0.035	0.023
2	12.635	9824565	625814	99.965	99.977
Total		9826830	626033	100.000	100.000

8.20 (*Sa,Sp*)-4-(1-(2-cyclohexylmethoxy)naphthyl)-9-fluoro-naphtho[1,2-a]ferrocene. ((*Sa,Sp*)-2as)

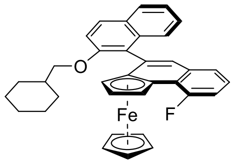

Isolated (-)-**2as** in 91% yield, >20:1 d.r., as pink solid.

^{1}H NMR (500 MHz, $CDCl_3$) δ 8.01 (s, 1H), 7.90 (s, 1H), 7.64 – 7.41 (m, 4H), 7.33 (s, 4H), 5.69 (s, 1H), 4.23 (s, 3H), 3.92 (s, 6H), 2.08 – 1.84 (m, 3H), 1.76 – 1.68 (m, 3H), 1.19 (d, *J* = 26.3 Hz, 5H); **^{13}C NMR** (125 MHz, $CDCl_3$) δ δ 159.9 (d, *J* = 247.7 Hz), 153.9, 136.3, 135.1, 135.1, 133.4, 129.5, 128.9, 127.9, 126.8, 126.1 (d, *J* = 3.8 Hz), 125.4 (d, *J* = 8.4 Hz), 124.1, 123.5, 123.3 (d, *J* = 15.5 Hz), 122.7, 115.0, 112.4 (d, *J* = 21.4 Hz), 86.3, 78.5, 74.9, 70.1, 69.5, 65.7 (d, *J* = 10.7 Hz), 64.7, 38.0, 30.3, 29.9, 26.5, 25.8, 25.6; **HRMS** (ESI) calculated for [$C_{35}H_{31}FFeNaO$] $[M+Na]^+$: 565.1601, found: 565.1607; **IR** (KBr, cm^{-1}) 2924, 2320, 1748, 1508, 1261, 1049, 750, 500; **$[\alpha]_D^{20}$** = -3236.1 (c = 0.1, $CHCl_3$); **HPLC conditions**: Daicel Chiralpak ODH column (98:2 hexane: 2-propanol, 0.5 mL/min, 254 nm); tr (minor) = 12.7 min, tr (major) = 9.1 min; 95% ee.

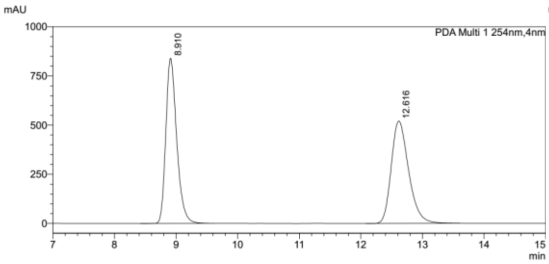

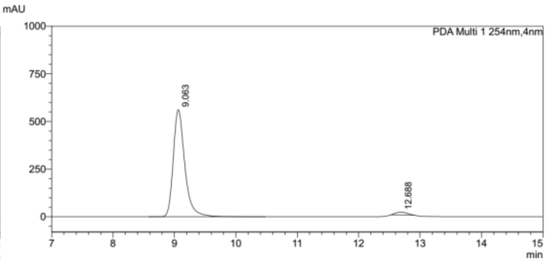

<Peak Table>
PDA Ch1 254nm

Peak#	Ret. Time	Area	Height	Height%	Area%
1	8.910	10274032	840386	61.734	50.128
2	12.616	10221535	520910	38.266	49.872
Total		20495568	1361296	100.000	100.000

<Peak Table>
PDA Ch1 254nm

Peak#	Ret. Time	Area	Height	Height%	Area%
1	9.063	7330180	561932	97.422	97.397
2	12.688	195935	14871	2.578	2.603
Total		7526115	576803	100.000	100.000

8.21 (*Sa,Sp*)-4-(1-(2-cyclohexylmethoxy)naphthyl)-9-chloro-naphtho[1,2-a]ferrocene. ((*Sa,Sp*)-2at)

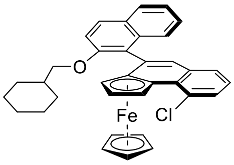

Isolated (-)-**2at** in 91% yield, >20:1 d.r., as pink solid.

^{1}H NMR (400 MHz, $CDCl_3$) δ 7.93 (d, *J* = 9.0 Hz, 1H), 7.82 (d, *J* = 8.0 Hz, 1H), 7.55 (t, *J* = 8.0 Hz, 2H), 7.50 (d, *J* = 8.8 Hz, 1H), 7.33 (d, *J* = 8.0 Hz, 1H), 7.27 (d, *J* = 8.0 Hz, 1H), 7.24 – 7.22 (m, 2H), 7.19 – 7.14 (m, 1H), 6.15 (d, *J* = 1.2 Hz, 1H), 4.21 – 4.15 (m, 3H), 3.89 (d, *J* = 8.4 Hz, 1H), 3.86 (s, 5H), 1.93 (d, *J* = 12.4 Hz, 1H), 1.80 – 1.74 (m, 2H), 1.70 – 1.60 (m, 3H), 1.19 – 1.02 (m, 5H); **^{13}C NMR** (100 MHz, $CDCl_3$) δ 153.9, 136.0, 135.3, 133.3, 132.8, 130.9, 129.4, 128.8, 128.5, 127.8, 127.6, 127.5, 126.0, 125.9, 125.2, 123.5, 122.5, 114.9, 87.4, 80.3, 74.9, 70.2, 69.5, 67.0, 65.2, 37.9, 30.2, 29.8, 26.5, 25.7, 25.5; **HRMS** (ESI) calculated for [$C_{35}H_{31}ClFeNaO$] [M+Na]$^+$: 581.1306, found: 581.1320; **IR** (KBr, cm^{-1}) 2924, 2320, 1748, 1508, 1260, 1020, 750, 500; **[α]$_D^{20}$** = -3080.6 (c = 0.1, $CHCl_3$); **HPLC conditions**: Daicel Chiralpak ODH column (98:2 hexane: 2-propanol, 0.5 mL/min, 254 nm); tr (minor) = 13.9 min, tr (major) = 9.3 min; 92% ee.

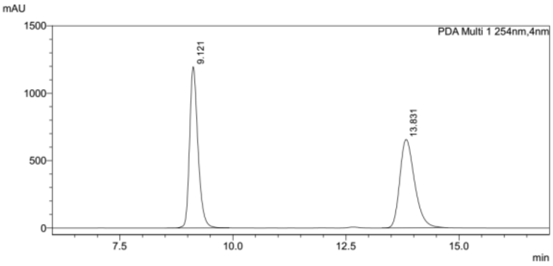

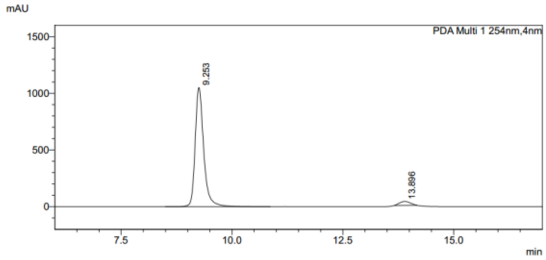

<Peak Table>
PDA Ch1 254nm

Peak#	Ret. Time	Area	Height	Height%	Area%
1	9.121	15446650	1198475	64.588	50.743
2	13.831	14994052	657102	35.412	49.257
Total		30440702	1855577	100.000	100.000

<Peak Table>
PDA Ch1 254nm

Peak#	Ret. Time	Area	Height	Height%	Area%
1	9.253	14058171	1050989	96.863	96.055
2	13.896	577419	34039	3.137	3.945
Total		14635590	1085027	100.000	100.000

8.22 (*Sa,Sp*)-4-(1-(2-cyclohexylmethoxy)naphthyl)-7,8-difluoro-naphtho[1,2-a]ferrocene. ((*Sa,Sp*)-2au)

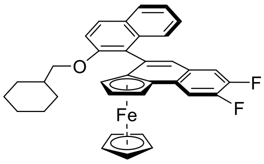

Isolated (-)-**2au** in 92% yield, >20:1 d.r., as pink solid.

^{1}H NMR (400 MHz, $CDCl_3$) δ 7.95 (d, *J* = 9.0 Hz, 1H), 7.87 – 7.79 (m, 2H), 7.52 (d, *J* = 9.2 Hz, 1H), 7.44 – 7.38 (m, 1H), 7.32 – 7.28 (m, 1H), 7.26 – 7.18 (m, 2H), 7.17 (s, 1H), 5.26 – 5.24 (m, 1H), 4.24 – 4.17 (m, 2H), 4.15 (t, *J* = 2.4 Hz, 1H), 3.94 – 3.87 (m, 1H), 3.83 (s, 5H), 1.96 (d, *J* = 12.8 Hz, 1H), 1.85 – 1.78 (m, 2H), 1.75 – 1.64 (m, 3H), 1.24 – 1.05 (m, 5H); **^{13}C NMR** (100 MHz, $CDCl_3$) δ 153.9, 150.4 (dd, *J* = 86.8, 14.1 Hz), 147.9 (dd, *J* = 84.4, 14.1 Hz), 135.6, 133.3, 131.6 (dd, *J* = 6.9, 2.6 Hz), 129.5, 129.1 (dd, *J* = 6.3, 2.5 Hz), 128.8, 127.8, 126.0, 125.9, 123.5, 122.4, 115.5 (d, *J* = 16.9 Hz), 114.9, 110.9 (d, *J* = 17.7 Hz), 86.2, 82.2, 74.8, 69.6, 69.5, 65.1, 61.2, 37.9, 30.3, 29.8, 26.5, 25.7, 25.5; **HRMS** (ESI) calculated for [$C_{35}H_{30}F_2FeNaO$] [M+Na]$^+$: 583.1507, found: 583.1515; **IR** (KBr, cm^{-1}) 2924, 2320, 1748, 1516, 1261, 1022, 750, 500; **[α]$_D^{20}$** = -2235.1 (c = 0.1, $CHCl_3$); **HPLC conditions**: Daicel Chiralpak ADH column (98:2 hexane: 2-propanol, 0.5 mL/min, 254 nm); tr (minor) = 11.1 min, tr (major)

= 12.5 min; 88% ee.

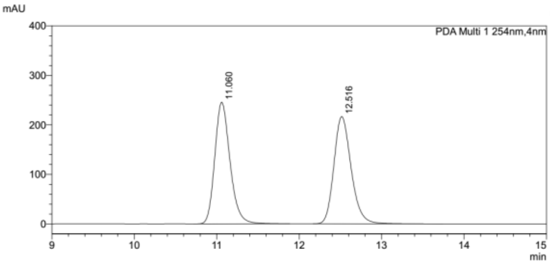

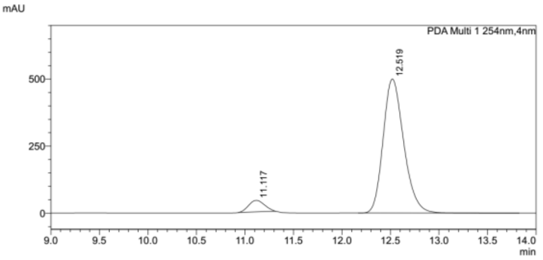

<Peak Table>
PDA Ch1 254nm

Peak#	Ret. Time	Area	Height	Height%	Area%
1	11.060	3153511	245845	53.150	50.017
2	12.516	3151333	216707	46.850	49.983
Total		6304843	462553	100.000	100.000

<Peak Table>
PDA Ch1 254nm

Peak#	Ret. Time	Area	Height	Height%	Area%
1	11.117	493255	43253	7.960	6.243
2	12.519	7407053	500097	92.040	93.757
Total		7900307	543350	100.000	100.000

8.23 (*Sa,Sp*)-4-(1-(2-cyclohexylmethoxy)naphthyl)-9-chloro-7-fluoro-naphtho[1,2-a]ferrocene. ((*Sa,Sp*)-2av)

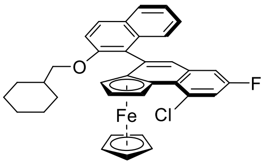

Isolated (-)-**2av** in 92% yield, >20:1 d.r., as pink solid.
^{1}H NMR (500 MHz, $CDCl_3$) δ 8.00 (s, 1H), 7.89 (s, 1H), 7.60 – 7.35 (m, 4H), 7.30 – 7.18 (m, 4H), 6.17 (s, 1H), 4.23 (s, 3H), 3.93 (s, 6H), 2.05 – 1.84 (m, 3H), 1.78 – 1.56 (m, 3H), 1.35 – 1.14 (m, 5H); **^{13}C NMR** (125 MHz, $CDCl_3$) δ 159.4 (d, *J* = 245.2 Hz), 153.9, 138.1, 136.1 (d, *J* = 8.6 Hz), 133.3, 131.6 (d, *J* = 10.0 Hz), 129.7, 129.2, 128.8, 127.9, 126.9, 126.2, 125.9, 123.6, 122.1, 116.5 (d, *J* = 25.7 Hz), 114.9, 112.8 (d, *J* = 20.4 Hz), 86.7, 80.3, 74.9, 70.3, 69.5, 66.7, 65.3, 38.0, 30.3, 29.8, 26.5, 25.8, 25.6; **HRMS** (ESI) calculated for [$C_{35}H_{30}ClFFeNaO$] [M+Na]$^+$: 599.1212, found: 599.1216; **IR** (KBr, cm^{-1}) 2924, 2320, 1750, 1508, 1246, 1010, 750, 500; **[α]$_D^{20}$** = -3393.9 (c = 0.1, $CHCl_3$); **HPLC conditions**: Daicel Chiralpak ADH column (98:2 hexane: 2-propanol, 0.5 mL/min, 254 nm); tr (minor) = 9.4 min, tr (major) = 9.9 min; 87% ee.

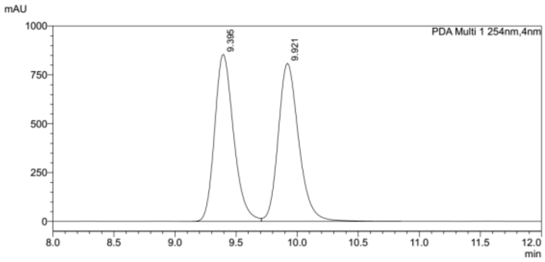

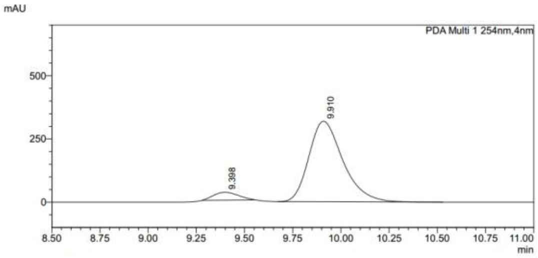

<Peak Table>
PDA Ch1 254nm

Peak#	Ret. Time	Area	Height	Height%	Area%
1	9.395	9267437	854935	51.369	49.536
2	9.921	9441084	809354	48.631	50.464
Total		18708521	1664289	100.000	100.000

<Peak Table>
PDA Ch1 254nm

Peak#	Ret. Time	Area	Height	Height%	Area%
1	9.398	278164	30984	8.854	6.613
2	9.910	3928305	318960	91.146	93.387
Total		4206469	349944	100.000	100.000

8.24 (*Sa,Sp*)-4-(1-(2-cyclohexylmethoxy)naphthyl)-7-trifluoromethyl-naphtho[1,2-a]ferrocene. ((*Sa,Sp*)-2aw)

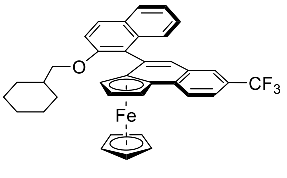

Isolated (-)-**2aw** in 92% yield, >20:1 d.r., as pink solid.
^{1}H NMR (400 MHz, $CDCl_3$) δ 8.15 (d, *J* = 8.2 Hz, 1H), 7.94 (d, *J* = 9.2 Hz, 1H), 7.88 (s, 1H), 7.83 (d, *J* = 8.0 Hz, 1H), 7.71 (d, *J* = 8.2 Hz, 1H), 7.52 (d, *J* = 9.2 Hz, 1H), 7.32 – 7.27 (m, 2H), 7.23 (d, *J* = 7.2 Hz, 1H), 7.20 – 7.15 (m, 1H), 5.41 – 5.36 (m, 1H), 4.23– 4.18 (m, 3H), 3.93 – 3.86 (m, 1H), 3.81 (s, 5H), 1.94 (d, *J* = 11.6 Hz, 1H), 1.82 – 1.76 (m, 2H), 1.74 – 1.62 (m, 3H), 1.22 – 1.04 (m, 5H); **^{13}C NMR** (100 MHz, $CDCl_3$) δ 153.9, 138.1, 136.6, 133.3, 132.1, 129.6, 128.9, 127.9, 127.0, 126.1, 125.9, 125.2 (q, *J* = 4.0 Hz), 123.6, 123.5, 122.5 (q, *J* = 3.4 Hz), 122.4, 114.9, 87.0, 81.6, 74.9, 70.2, 69.6, 65.5, 61.8, 38.0, 30.3, 29.8, 26.4, 25.7, 25.5; **HRMS** (ESI) calculated for [$C_{36}H_{31}F_3FeNaO$] [M+Na]$^+$: 615.1569, found: 615.1560; **IR** (KBr, cm^{-1}) 2926, 2320, 1750, 1508, 1263, 1122, 750, 500; **[α]$_D^{20}$** = -

2211.0 (c = 0.1, $CHCl_3$); **HPLC conditions**: Daicel Chiralpak ODH column (98:2 hexane: 2-propanol, 0.5 mL/min, 254 nm); tr (minor) = 10.3 min, tr (major) = 8.8 min; 90% ee.

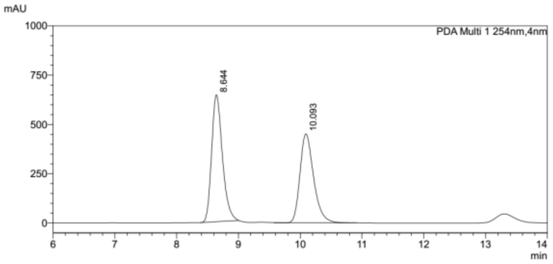

<Peak Table>
PDA Ch1 254nm

Peak#	Ret. Time	Area	Height	Height%	Area%
1	8.644	7788465	643789	58.768	52.897
2	10.093	6935306	451685	41.232	47.103
Total		14723771	1095475	100.000	100.000

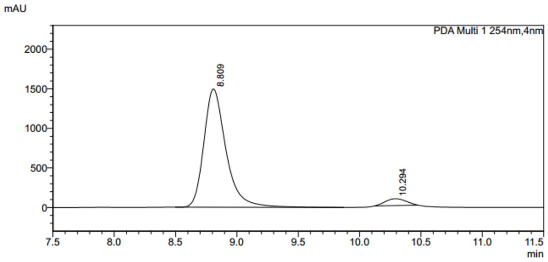

<Peak Table>
PDA Ch1 254nm

Peak#	Ret. Time	Area	Height	Height%	Area%
1	8.809	18789651	1495799	94.557	94.915
2	10.294	1006684	86109	5.443	5.085
Total		19796335	1581908	100.000	100.000

8.25 (*Sa,Sp*)-4-(1-(2-cyclohexylmethoxy)naphthyl)-7-acetyl-naphtho[1,2-a]ferrocene. ((*Sa,Sp*)-2ax)

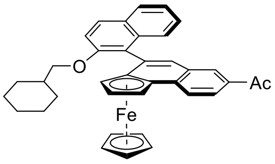

Isolated (-)-**2ax** in 92% yield, >20:1 d.r., as pink solid.

^{1}H NMR (400 MHz, $CDCl_3$) δ 8.24 (s, 1H), 8.15 – 8.07 (m, 2H), 7.95 (d, *J* = 8.8 Hz, 1H), 7.83 (d, *J* = 8.0 Hz, 1H), 7.52 (d, *J* = 8.8 Hz, 1H), 7.32 (s, 1H), 7.28 (d, *J* = 8.0 Hz, 1H), 7.25 (d, *J* = 3.6 Hz, 1H), 7.20 – 7.15 (m, 1H), 5.39 (s, 1H), 4.26 – 4.15 (m, 3H), 3.92 (t, *J* = 8.0 Hz, 1H), 3.81 (s, 5H), 2.71 (s, 3H), 1.96 (d, *J* = 10.8 Hz, 1H), 1.80 (d, *J* = 9.6 Hz,, 2H), 1.69 – 1.56 (m, 3H), 1.20 – 1.04 (m, 5H); **^{13}C NMR** (125 MHz, $CDCl_3$) δ 198.1, 154.0, 140.0, 136.0, 134.0, 133.3, 132.0, 129.6, 129.1, 128.9, 127.9, 127.5, 126.1, 126.0, 125.9, 123.6, 123.4, 122.5, 115.0, 87.4, 81.6, 75.0, 70.5, 69.8, 65.8, 62.1, 38.0, 30.3, 29.9, 26.8, 26.5, 25.8, 25.6; **HRMS** (ESI) calculated for $[C_{37}H_{34}FeNaO_2]$ $[M+Na]^+$: 589.1801, found: 589.1799; **IR** (KBr, cm^{-1}) 2924, 2318, 1750, 1508, 1274, 1022, 750, 490; **$[\alpha]_D^{20}$** = -850.5 (c = 0.1, $CHCl_3$); **HPLC conditions**: Daicel Chiralpak ADH column (98:2 hexane: 2-propanol, 0.5 mL/min, 254 nm); tr (minor) = 17.4 min, tr (major) = 18.2 min; 89% ee.

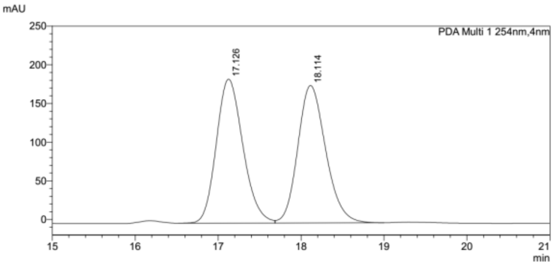

<Peak Table>
PDA Ch1 254nm

Peak#	Ret. Time	Area	Height	Height%	Area%
1	17.126	4159904	186031	51.131	49.964
2	18.114	4165942	177804	48.869	50.036
Total		8325846	363835	100.000	100.000

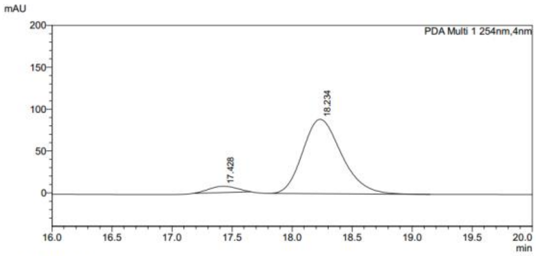

<Peak Table>
PDA Ch1 254nm

Peak#	Ret. Time	Area	Height	Height%	Area%
1	17.428	118873	7418	7.697	5.573
2	18.234	2013952	88956	92.303	94.427
Total		2132825	96375	100.000	100.000

8.26 (*Sa,Sp*)-4-(1-(2-cyclohexylmethoxy)naphthyl)-8-trifluoromethyl-naphtho[1,2-a]ferrocene. ((*Sa,Sp*)-2ay)

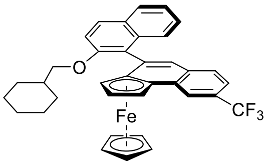

Isolated (-)-**2ay** in 89% yield, >20:1 d.r., as pink solid.

^{1}H NMR (400 MHz, $CDCl_3$) δ 8.31 (s, 1H), 7.94 (d, *J* = 8.8 Hz, 1H), 7.83 (d, *J* = 8.4 Hz, 1H), 7.71 (d, *J* = 8.2 Hz, 1H), 7.64 (dd, *J* = 8.2, 1.2 Hz, 1H), 7.52 (d, *J* = 9.2 Hz, 1H), 7.31 – 7.27 (m, 2H), 7.23 (d, *J* = 8.8 Hz, 1H), 7.20 – 7.14 (m, 1H), 5.40 – 5.39 (m, 1H), 4.22 – 4.16 (m, 3H), 3.91 (t, *J* = 8.4 Hz, 1H), 3.82 (s, 5H), 1.95 (d, *J* = 12.6 Hz, 1H), 1.84 – 1.75 (m, 2H), 1.72 – 1.61 (m, 3H), 1.23 – 1.03 (m, 5H); **^{13}C NMR** (100 MHz, $CDCl_3$) δ 153.8, 138.0, 134.9, 133.2, 129.6, 128.8, 128.7, 127.9, 126.5, 126.1, 125.9, 123.5, 122.3, 121.5 (q, *J* = 3.5 Hz), 120.1 (q, *J* = 8.1, 4.0 Hz), 114.9, 86.5, 82.4, 74.9, 70.0, 69.6, 65.4, 61.6, 48.1, 37.9, 30.3, 29.8, 26.5, 25.7, 25.5; **HRMS** (ESI) calculated for $[C_{36}H_{31}F_3FeNaO]$

[M+Na]+: 615.1569, found: 615.1569; **IR** (KBr, cm^{-1}) 2924, 2320, 1748, 1508, 1261, 1022, 750, 503; **[α]$_D^{20}$** = -2467.5 (c = 0.1, $CHCl_3$); **HPLC conditions**: Daicel Chiralpak ODH column (98:2 hexane: 2-propanol, 0.5 mL/min, 254 nm); tr (minor) = 13.4 min, tr (major) = 8.8 min; 91% ee.

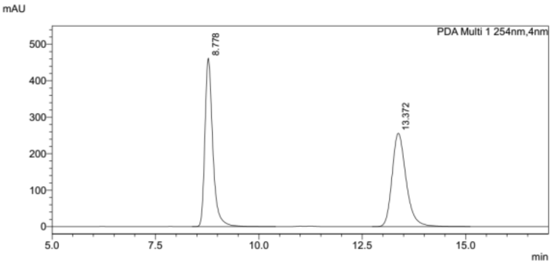

<Peak Table>
PDA Ch1 254nm

Peak#	Ret. Time	Area	Height	Height%	Area%
1	8.778	6107187	461968	64.345	50.252
2	13.372	6045940	255989	35.655	49.748
Total		12153127	717957	100.000	100.000

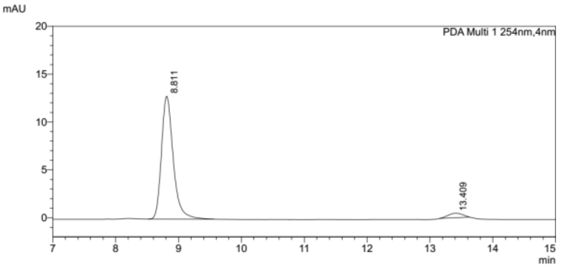

<Peak Table>
PDA Ch1 254nm

Peak#	Ret. Time	Area	Height	Height%	Area%
1	8.811	166945	12841	96.440	95.571
2	13.409	7737	474	3.560	4.429
Total		174682	13315	100.000	100.000

8.27 (*Sa,Sp*)-methyl-4-(1-(2-cyclohexylmethoxy)naphthyl)-7-carboxylate-naphtho[1,2-a]ferrocene. ((*Sa,Sp*)-2az)

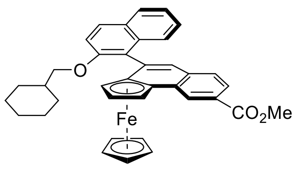

Isolated (-)-**2az** in 90% yield, >20:1 d.r., as pink solid.

Isolated *ent*-**2az** in 91% yield, >20:1 d.r., as pink solid.

^{1}H NMR (400 MHz, $CDCl_3$) δ 8.80 (s, 1H), 8.10 – 8.07 (m, 1H), 7.95 (d, *J* = 8.8 Hz, 1H), 7.84 (d, *J* = 8.4 Hz, 1H), 7.68 (d, *J* = 8.4 Hz, 1H), 7.53 (d, *J* = 9.2 Hz, 1H), 7.33 – 7.26 (m, 3H), 7.22 – 7.16 (m, 1H), 5.49 – 5.48 (m, 1H), 4.23 – 4.17 (m, 3H), 4.05 (s, 3H), 3.91 (t, *J* = 8.4 Hz, 1H), 3.82 (s, 5H), 1.96 (d, *J* = 11.6 Hz, 1H), 1.85 – 1.76 (m, 2H), 1.71 – 1.59 (m, 3H), 1.21 – 1.03 (m, 5H); **^{13}C NMR** (100 MHz, $CDCl_3$) δ 167.6, 153.8, 138.5, 136.2, 134.5, 133.2, 129.5, 128.8, 128.2, 127.8, 127.5, 126.9, 126.0, 125.9, 125.8, 125.0, 123.5, 122.5, 114.8, 86.2, 82.9, 74.8, 69.9, 69.6, 65.3, 61.8, 52.2, 38.0, 30.3, 29.8, 26.4, 25.7, 25.5; **HRMS** (ESI) calculated for [$C_{37}H_{34}FeNaO_3$] [M+Na]+: 605.1750, found: 605.1741; **IR** (KBr, cm^{-1}) 2924, 2320, 1720, 1508, 1261, 1022, 750, 501; **[α]$_D^{20}$** = over range (c = 0.1, $CHCl_3$); **HPLC conditions**: Daicel Chiralpak ADH column (95:5 hexane: 2-propanol, 0.5 mL/min, 254 nm); **2az** [tr (minor) = 10.7 min, tr (major) = 15.3 min; 91% ee]; *ent*-**2az** [tr (minor) = 15.3 min, tr (major) = 10.7 min; 92% ee];

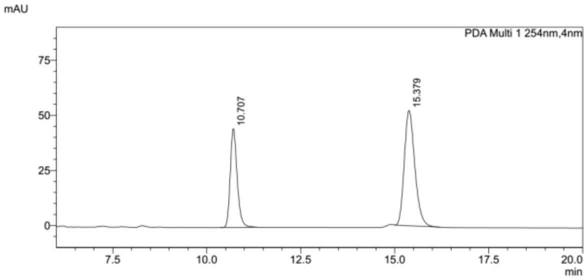

<Peak Table>
PDA Ch1 254nm

Peak#	Ret. Time	Area	Height	Height%	Area%
1	10.707	575851	44835	46.156	36.080
2	15.379	1020201	52303	53.844	63.920
Total		1596052	97138	100.000	100.000

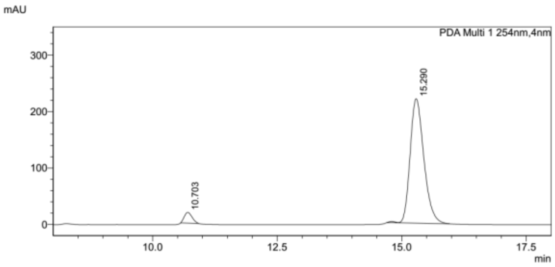

<Peak Table>
PDA Ch1 254nm

Peak#	Ret. Time	Area	Height	Height%	Area%
1	10.703	203795	18894	7.883	4.573
2	15.290	4252536	220795	92.117	95.427
Total		4456332	239688	100.000	100.000

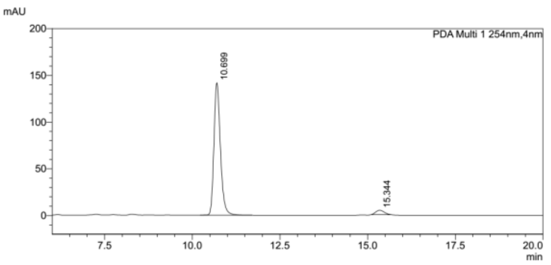

<Peak Table>
PDA Ch1 254nm

Peak#	Ret. Time	Area	Height	Height%	Area%
1	10.699	1817652	141776	96.819	95.886
2	15.344	77980	4659	3.181	4.114
Total		1895632	146435	100.000	100.000

8.28 (*Sa,Sp*)-4-(1-(2-cyclohexylmethoxy)naphthyl)-8-methyl-naphtho[1,2-a]ferrocene. ((*Sa,Sp*)-2ba)

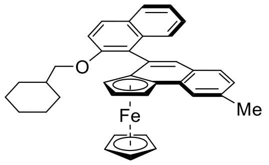

Isolated (-)-**2ba** in 90% yield, >20:1 d.r., as pink solid.

^{1}H NMR (400 MHz, $CDCl_3$) δ 7.91 (d, *J* = 8.8 Hz, 1H), 7.87 (s, 1H), 7.83 – 7.79 (m, 1H), 7.54 (d, *J* = 7.6 Hz, 1H), 7.50 (d, *J* = 9.2 Hz, 1H), 7.27 – 7.24 (m, 3H), 7.23 (s, 1H), 7.18 – 7.13 (m, 1H), 5.30 – 5.28 (m, 1H), 4.20 – 4.15 (m, 1H), 4.13 – 4.12 (m, 1H), 4.09 (t, *J* = 2.4 Hz, 1H), 3.92 – 3.86 (m, 1H), 3.79 (s, 5H), 2.59 (s, 3H), 1.96 (d, *J* = 12.4 Hz, 1H), 1.83 – 1.74 (m, 2H), 1.70 – 1.59 (m, 3H), 1.19 – 1.02 (m, 5H); **^{13}C NMR** (100 MHz, $CDCl_3$) δ 154.0, 136.2, 134.7, 133.5, 133.2, 130.2, 129.1, 128.9, 128.4, 127.7, 127.5, 126.6, 126.2, 125.8, 123.4, 123.3, 115.0, 86.8, 82.9, 74.9, 69.5, 69.1, 64.7, 60.9, 37.9, 30.2, 29.9, 26.5, 25.8 25.6, 21.9; **HRMS** (ESI) calculated for $[C_{36}H_{34}FeNaO]$ $[M+Na]^+$: 561.1852, found: 561.1859; **IR** (KBr, cm^{-1}) 2924, 2320, 1750, 1508, 1261, 1022, 750, 490; **$[\alpha]_D^{20}$** = -2433.3 (c = 0.1, $CHCl_3$); **HPLC conditions**: Daicel Chiralpak ADH column (98:2 hexane: 2-propanol, 0.5 mL/min, 254 nm); tr (minor) = 9.8 min, tr (major) = 10.1 min; 91% ee.

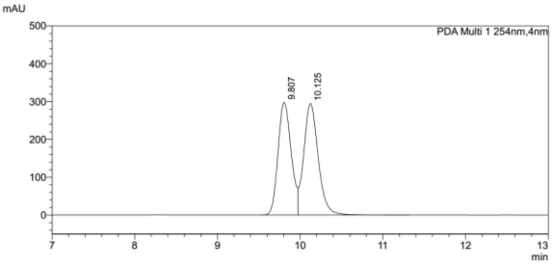

<Peak Table>
PDA Ch1 254nm

Peak#	Ret. Time	Area	Height	Height%	Area%
1	9.807	3301277	297641	50.220	48.060
2	10.125	3567824	295035	49.780	51.940
Total		6869101	592675	100.000	100.000

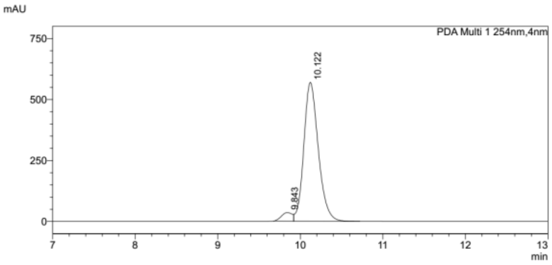

<Peak Table>
PDA Ch1 254nm

Peak#	Ret. Time	Area	Height	Height%	Area%
1	9.843	325440	35095	5.806	4.492
2	10.122	6918744	569343	94.194	95.508
Total		7244184	604438	100.000	100.000

8.29 (*Sa,Sp*)-4-(1-(2-cyclohexylmethoxy)naphthyl)-8-trifluoromethoxy-naphtho[1,2-a]ferrocene. ((*Sa,Sp*)-2bb)

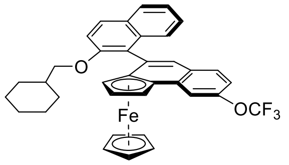

Isolated (-)-**2bb** in 92% yield, >20:1 d.r., as pink solid.

^{1}H NMR (400 MHz, $CDCl_3$) δ 7.96 – 7.82 (m, 3H), 7.63 (s, 1H), 7.51 (s, 1H), 7.30 - 7.15 (m, 5H), 5.31 (s, 1H), 4.18 (s, 3H), 3.89 (s, 1H), 3.81 (s, 5H), 1.94 (s, 1H), 1.83 – 1.64 (m, 5H), 1.22 – 1.05 (m, 5H); **^{13}C NMR** (100 MHz, $CDCl_3$) δ 153.9, 147.6, 136.4, 135.4, 133.4, 130.9, 129.7, 129.4, 128.9, 127.8, 126.5, 126.0, 125.9, 123.5, 122.6, 118.1, 115.0, 114.9, 86.7, 82.0, 74.9, 69.8, 69.6, 65.3, 61.4, 37.9, 30.3, 29.8, 26.5, 25.7, 25.5; **RMS** (ESI) calculated for $[C_{36}H_{31}F_3FeNaO_2]$ $[M+Na]^+$: 631.1518, found: 631.1515; **IR** (KBr, cm^{-1}) 2924, 2320, 1750, 1508, 1261, 1022, 750, 490; **$[\alpha]_D^{20}$** = -2408.1 (c = 0.1, $CHCl_3$); **HPLC conditions**: Daicel Chiralpak ADH column (98:2 hexane: 2-propanol, 0.5 mL/min, 254 nm); tr (minor) = 8.1 min, tr (major) = 9.0 min; 94% ee.

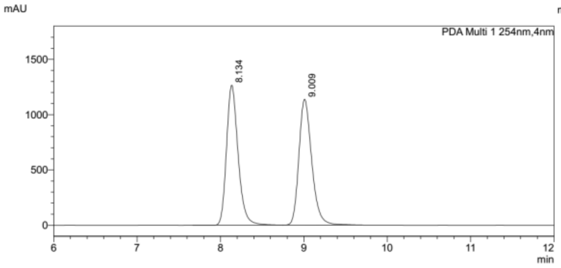

<Peak Table>
PDA Ch1 254nm

Peak#	Ret. Time	Area	Height	Height%	Area%
1	8.134	12189773	1264930	52.671	50.076
2	9.009	12152949	1136631	47.329	49.924
Total		24342722	2401562	100.000	100.000

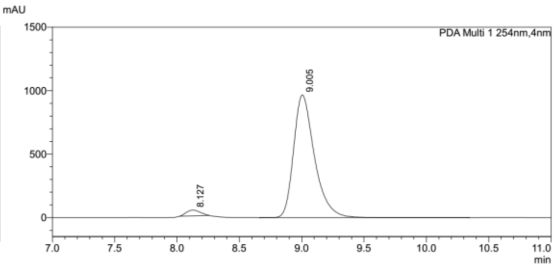

<Peak Table>
PDA Ch1 254nm

Peak#	Ret. Time	Area	Height	Height%	Area%
1	8.127	351822	45388	4.483	3.036
2	9.005	11236766	966955	95.517	96.964
Total		11588588	1012343	100.000	100.000

8.30 (*Sa,Sp*)-4-(1-(2-cyclohexylmethoxy)naphthyl)-7-methyl-naphtho[1,2-a]ferrocene. ((*Sa,Sp*)-2bc)

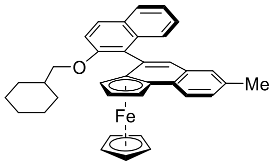

Isolated (-)-**2bc** in 91% yield, >20:1 d.r., as pink solid.

^{1}H NMR (400 MHz, $CDCl_3$) δ 7.97 (d, *J* = 8.0 Hz, 1H), 7.91 (d, *J* = 8.8Hz, 1H), 7.81 (d, *J* = 8.0 Hz, 1H), 7.50 (d, *J* = 9.2 Hz, 1H), 7.44 (s, 1H), 7.34 (d, *J* = 8.0 Hz, 1H), 7.26 (d, *J* = 7.8 Hz, 2H), 7.22 (d, *J* = 8.0 Hz, 1H), 7.18 – 7.12 (m, 1H), 5.29 (s, 1H), 4.19 – 4.16 (m, 1H), 4.13 – 4.07 (m, 2H), 3.90 (t, *J* = 8.4 Hz, 1H), 3.78 (s, 5H), 2.49 (s, 3H), 1.97 (d, *J* = 12.4 Hz, 1H), 1.85 – 1.75 (m, 2H), 1.71 – 1.59 (m, 3H), 1.22 – 1.04 (m, 5H); **^{13}C NMR** (100 MHz, $CDCl_3$) δ 153.9, 134.7, 134.5, 133.5, 132.6, 131.9, 129.1, 128.9, 128.6, 127.8, 127.7, 127.5, 126.2, 125.9, 123.4, 123.3, 123.1, 115.0, 86.4, 83.3, 75.0, 69.5, 68.9, 64.6, 60.9, 37.9, 30.3, 29.9, 26.5, 25.8, 25.6, 21.5; **HRMS** (ESI) calculated for [$C_{36}H_{34}FeNaO$] $[M+Na]^+$: 561.1852, found: 561.1860; **IR** (KBr, cm^{-1}) 2924, 2320, 1748, 1508, 1261, 1022, 750, 490; **$[\alpha]_D^{20}$** = -2488.3 (c = 0.1, $CHCl_3$); **HPLC conditions**: Daicel Chiralpak ADH column (98:2 hexane: 2-propanol, 0.5 mL/min, 254 nm); tr (minor) = 9.6 min, tr (major) = 14.1 min; 90% ee.

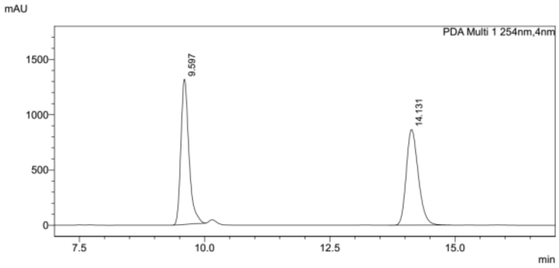

<Peak Table>
PDA Ch1 254nm

Peak#	Ret. Time	Area	Height	Height%	Area%
1	9.597	14668522	1314612	60.294	50.418
2	14.131	14425037	865726	39.706	49.582
Total		29093559	2180338	100.000	100.000

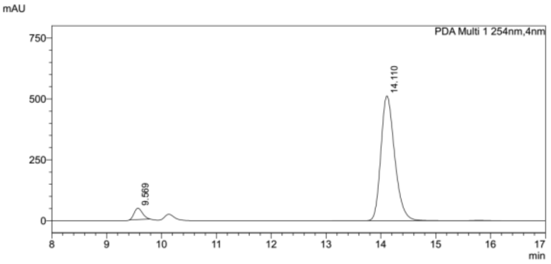

<Peak Table>
PDA Ch1 254nm

Peak#	Ret. Time	Area	Height	Height%	Area%
1	9.569	488775	46662	8.345	5.154
2	14.110	8993742	512475	91.655	94.846
Total		9482517	559136	100.000	100.000

8.31 (*Sa,Sp*)-4-(1-(2-cyclohexylmethoxy)naphthyl)-7-tert-butyl-naphtho[1,2-a]ferrocene. ((*Sa,Sp*)-2bd)

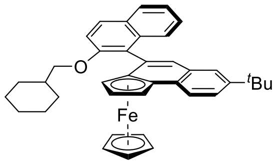

Isolated (-)-**2bd** in 90% yield, >20:1 d.r., as pink solid.

^{1}H NMR (400 MHz, $CDCl_3$) δ 8.00 (d, *J* = 8.0 Hz, 1H), 7.92 (d, *J* = 9.2 Hz, 1H), 7.82 (d, *J* = 8.0 Hz, 1H), 7.63 (d, *J* = 1.8 Hz, 1H), 7.58 – 7.55 (m, 1H), 7.50 (d, *J* = 9.2 Hz, 1H), 7.30 (d, *J* = 8.8 Hz, 2H), 7.25 (d, *J* = 10.2 Hz, 1H), 7.19 – 7.13 (m, 1H), 5.33 – 5.27 (m, 1H), 4.22 – 4.19 (m, 1H), 4.15 – 4.10 (m, 1H), 4.08 (t, *J* = 2.4 Hz, 1H), 3.91 – 3.86 (m, 1H), 3.79 (s, 5H), 1.98 (d, *J* = 10.2 Hz, 1H), 1.83 – 1.76 (m, 2H), 1.71 – 1.61 (m, 3H), 1.43 (s, 9H), 1.20 – 1.05 (m, 5H); **^{13}C NMR** (100 MHz, $CDCl_3$) δ 153.9, 148.0, 134.0, 133.5, 132.2, 131.9, 129.1, 128.9, 128.4, 127.7, 126.3, 125.8, 124.8, 124.1, 123.4, 122.9, 115.0, 86.4, 83.1, 74.9, 69.3, 68.9, 64.5, 60.9, 38.1, 34.7, 31.5, 30.3, 29.8, 26.5, 25.9, 25.6; **HRMS** (ESI) calculated for [$C_{39}H_{40}FeNaO$] $[M+Na]^+$: 603.2321, found: 603.2319; **IR** (KBr, cm^{-1}) 2924, 2312, 1750, 1508, 1261, 1022, 750, 490; **$[\alpha]_D^{20}$** = -2033.8 (c = 0.1, $CHCl_3$); **HPLC conditions**: Daicel Chiralpak ADH column (98:2 hexane: 2-propanol, 0.5 mL/min, 254 nm); tr (minor) = 7.5 min, tr (major) = 8.4 min; 81% ee.

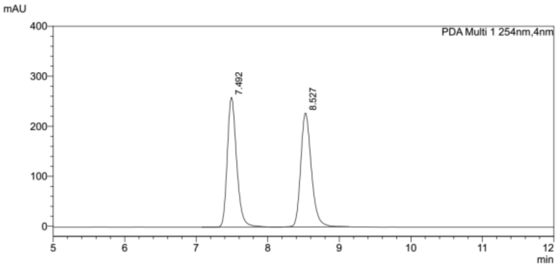

<Peak Table>
PDA Ch1 254nm

Peak#	Ret. Time	Area	Height	Height%	Area%
1	7.492	2364493	259349	53.274	50.024
2	8.527	2362258	227471	46.726	49.976
Total		4726751	486819	100.000	100.000

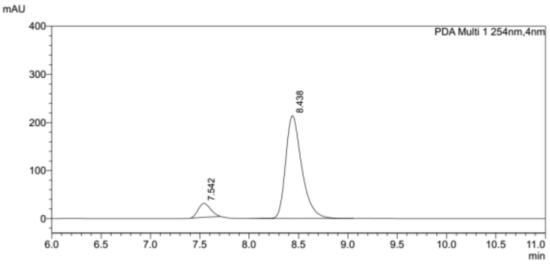

<Peak Table>
PDA Ch1 254nm

Peak#	Ret. Time	Area	Height	Height%	Area%
1	7.542	254658	29191	12.009	9.486
2	8.438	2430041	213881	87.991	90.514
Total		2684700	243071	100.000	100.000

8.32 (*Sa,Sp*)-4-(1-(2-cyclohexylmethoxy)naphthyl)-7-methoxy-naphtho[1,2-a]ferrocene. ((*Sa,Sp*)-2be)

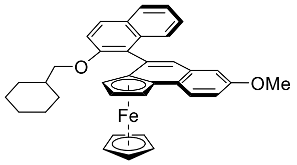

Isolated (-)-**2be** in 93% yield, >20:1 d.r., as pink solid.

^{1}H NMR (400 MHz, $CDCl_3$) δ 7.91 (d, *J* = 9.0 Hz, 1H), 7.81 (d, *J* = 8.0 Hz, 1H), 7.57 (d, *J* = 8.8 Hz, 1H), 7.52 (d, *J* = 2.4 Hz, 1H), 7.50 (d, *J* = 9.2 Hz, 1H), 7.29 (s, 1H), 7.24 (d, *J* = 7.2 Hz, 1H), 7.21 (s, 1H), 7.19 – 7.13 (m, 1H), 7.06 – 7.03 (m, 1H), 5.26 (d, *J* = 1.2 Hz, 1H), 4.20 – 4.16 (m, 1H), 4.14 (s, 1H), 4.11 (d, *J* = 2.0 Hz, 1H), 4.02 (s, 3H), 3.88 (t, *J* = 8.4 Hz, 1H), 3.80 (s, 5H), 1.96 (d, *J* = 12.4 Hz, 1H), 1.84 – 1.76 (m, 2H), 1.71 – 1.60 (m, 3H), 1.20 – 1.02 (m, 5H); **^{13}C NMR** (100 MHz, $CDCl_3$) δ 158.5, 154.0, 136.3, 133.6, 131.3, 129.9, 129.1, 128.9, 127.8, 127.3, 126.5, 126.2, 125.9, 123.4, 123.3, 115.1, 113.3, 106.3, 87.2, 82.6, 74.9, 69.5, 69.2, 64.8, 61.0, 55.6, 38.0, 30.3, 29.9, 26.5, 25.8, 25.6; **HRMS** (ESI) calculated for [$C_{36}H_{34}FeNaO_2$] [M+Na]$^+$: 577.1801, found: 577.1808; **IR** (KBr, cm^{-1}) 2924, 2320, 1750, 1508, 1261, 1022, 750, 500; **[α]$_D$^{20}** = -2523.0 (c = 0.1, $CHCl_3$); **HPLC conditions**: Daicel Chiralpak ADH column (95:5 hexane: 2-propanol, 0.5 mL/min, 254 nm); tr (minor) = 9.6 min, tr (major) = 10.6 min; 93% ee.

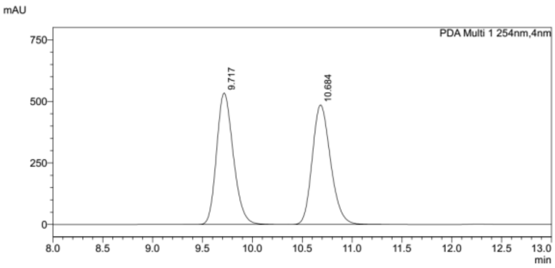

<Peak Table>

PDA Ch1 254nm

Peak#	Ret. Time	Area	Height	Height%	Area%
1	9.717	6241266	534551	52.394	50.137
2	10.684	6207051	485705	47.606	49.863
Total		12448316	1020256	100.000	100.000

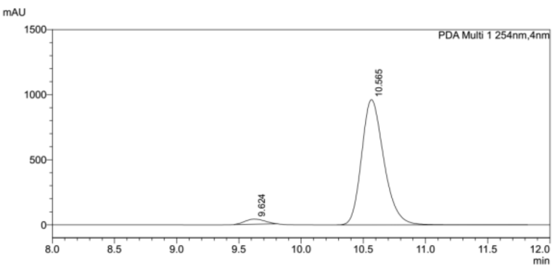

<Peak Table>

PDA Ch1 254nm

Peak#	Ret. Time	Area	Height	Height%	Area%
1	9.624	436926	39353	3.928	3.511
2	10.565	12009286	962537	96.072	96.489
Total		12446213	1001890	100.000	100.000

8.33 (*Sa,Sp*)-4-(1-(2-cyclohexylmethoxy)naphthyl)-7-trifluoromethoxy-naphtho[1,2-a]ferrocene. ((*Sa,Sp*)-2bf)

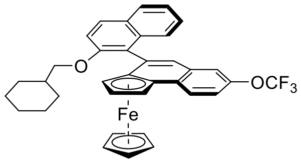

Isolated (-)-**2af** in 92% yield, >20:1 d.r., as pink solid.

^{1}H NMR (400 MHz, $CDCl_3$) δ 8.06 (d, *J* = 8.4 Hz, 1H), 7.93 (d, *J* = 9.2 Hz, 1H), 7.82 (d, *J* = 8.0 Hz, 1H), 7.50 (d, *J* = 9.2 Hz, 1H), 7.46 (s, 1H), 7.35 (d, *J* = 8.8 Hz, 1H), 7.31 – 7.26 (m, 1H), 7.23 (d, *J* = 2.4 Hz, 1H), 7.22 (s, 1H), 7.19 – 7.14 (m, 1H), 5.33 – 5.31 (m, 1H), 4.23 – 4.15 (m, 2H), 4.13 (t, *J* = 2.8 Hz, 1H), 3.87 (t, *J* = 8.4 Hz, 1H), 3.80 (s, 5H), 1.94 (d, *J* = 11.6 Hz, 1H), 1.82 – 1.74 (m, 2H), 1.71 – 1.60 (m, 3H), 1.19 – 1.10 (m, 5H); **^{13}C NMR** (100 MHz, $CDCl_3$) δ 153.9, 146.7, 136.8, 133.4, 133.4, 133.3, 129.5, 128.8, 127.8, 126.7, 126.0, 125.9, 124.5, 123.5, 122.5, 119.8, 119.5, 114.9, 86.3, 82.3, 74.6, 69.7, 69.5, 65.2, 61.4, 38.0, 30.3, 29.8, 26.5, 25.7, 25.4; **HRMS** (ESI) calculated for [$C_{36}H_{31}F_3FeNaO_2$] [M+Na]$^+$: 631.1518, found: 631.1536; **IR** (KBr, cm^{-1}) 2924, 2310, 1750, 1508, 1260, 1020, 750, 490; **[α]$_D$^{20}** = -2236.7 (c = 0.1, $CHCl_3$); **HPLC conditions**: Daicel Chiralpak ODH column (98:2 hexane: 2-propanol, 0.5 mL/min, 254 nm); tr (minor) = 10.0 min, tr (major) = 8.4 min; 92% ee.

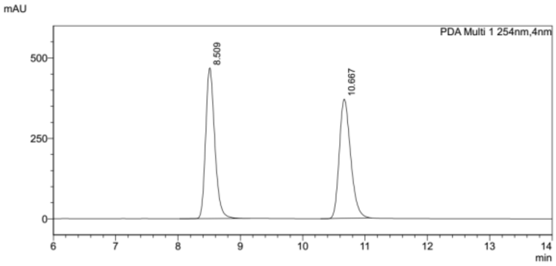

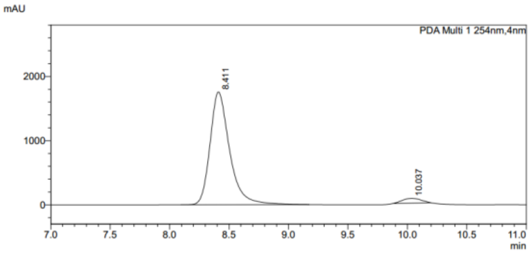

<Peak Table>
PDA Ch1 254nm

Peak#	Ret. Time	Area	Height	Height%	Area%
1	8.509	4760665	468629	55.816	50.342
2	10.667	4695970	370962	44.184	49.658
Total		9456635	839592	100.000	100.000

<Peak Table>
PDA Ch1 254nm

Peak#	Ret. Time	Area	Height	Height%	Area%
1	8.411	20103564	1753550	95.940	96.130
2	10.037	809432	74204	4.060	3.870
Total		20912996	1827754	100.000	100.000

8.34 (*Sa*,*Sp*)-4-(1-(2-cyclohexylmethoxy)naphthyl)-6-methyl-naphtho[1,2-a]ferrocene. ((*Sa*,*Sp*)-2bg)

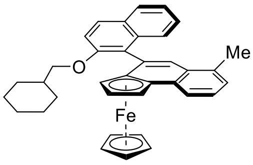

Isolated (-)-**2bg** in 92% yield, >20:1 d.r., as pink solid.

^{1}H NMR (400 MHz, $CDCl_3$) δ 7.95 (dd, *J* = 11.2, 8.8 Hz, 2H), 7.82 (d, *J* = 8.0 Hz, 1H), 7.52 (t, *J* = 4.4 Hz, 2H), 7.41 (t, *J* = 7.6 Hz, 1H), 7.28 (d, *J* = 6.8 Hz, 2H), 7.24 (d, *J* = 1.6 Hz, 1H), 7.19 – 7.13 (m, 1H), 5.34 (s, 1H), 4.20 – 4.08 (m, 3H), 3.91 (t, *J* = 8.4 Hz, 1H), 3.78 (s, 5H), 2.58 (s, 3H), 2.00 (d, *J* = 12.4 Hz, 1H), 1.85 – 1.76 (m, 2H), 1.72 – 1.62 (m, 3H), 1.18 – 1.01 (m, 5H); **^{13}C NMR** (100 MHz, $CDCl_3$) δ 153.8, 135.1, 134.6, 134.4, 133.6, 130.9, 129.2, 128.8, 127.7, 126.8, 126.2, 126.0, 125.9, 123.7, 123.4, 123.3, 121.4, 114.7, 86.4, 83.7, 74.6, 69.5, 69.3, 64.6, 61.3, 37.8, 30.3, 29.8, 26.5, 25.7, 25.5, 19.9; **HRMS** (ESI) calculated for [$C_{36}H_{34}FeNaO$] $[M+Na]^+$: 561.1852, found: 561.1865; **IR** (KBr, cm^{-1}) 2924, 2320, 1750, 1508, 1260, 1020, 750, 490; **$[\alpha]_D^{20}$** = -1850.5 (c = 0.1, $CHCl_3$); **HPLC conditions**: Daicel Chiralpak ODH column (98:2 hexane: 2-propanol, 0.5 mL/min, 254 nm); tr (minor) = 9.1 min, tr (major) = 9.5 min; 20% ee.

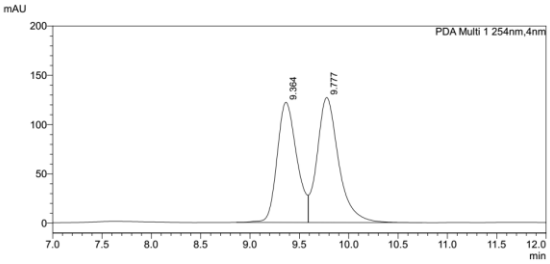

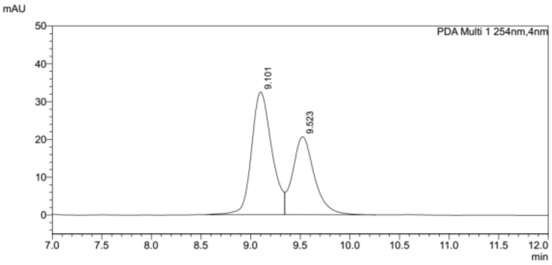

<Peak Table>
PDA Ch1 254nm

Peak#	Ret. Time	Area	Height	Height%	Area%
1	9.364	1702236	121983	49.028	47.020
2	9.777	1917990	126818	50.972	52.980
Total		3620226	248801	100.000	100.000

<Peak Table>
PDA Ch1 254nm

Peak#	Ret. Time	Area	Height	Height%	Area%
1	9.101	465344	32520	61.114	59.794
2	9.523	312897	20692	38.886	40.206
Total		778241	53212	100.000	100.000

8.35 (*Sa*,*Sp*)-4-(1-(2-cyclohexylmethoxy)-6-bromonaphthyl)-naphtho[1,2-a]ferrocene. ((*Sa*,*Sp*)-2bh)

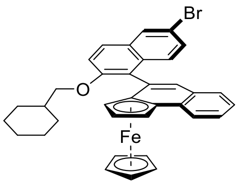

Isolated (-)-**2bh** in 92% yield, >20:1 d.r., as pink solid.

^{1}H NMR (400 MHz, $CDCl_3$) δ 8.07 (s, 1H), 7.97 (s, 1H), 7.82 (s, 1H), 7.64 (s, 1H), 7.52 (s, 2H), 7.44 (s, 1H), 7.24 (s, 1H), 7.20 (s, 1H), 7.16 (s, 1H), 5.33 (s, 1H), 4.11 (s, 3H), 3.89 (s, 1H), 3.78 (s, 5H), 1.95 (s, 1H), 1.82 – 1.56 (m, 5H), 1.28 – 1.06 (m, 5H); **^{13}C NMR** (100 MHz, $CDCl_3$) δ 154.2, 134.7, 133.8, 132.3, 132.0, 129.9, 129.6, 129.1, 128.5, 128.3, 128.0, 127.8, 126.6, 125.3, 123.3, 123.2, 117.2, 115.9, 86.4, 83.0, 74.9, 69.5, 69.3, 64.6, 61.3, 37.9, 30.2, 29.8, 26.5, 25.7, 25.5; **HRMS** (ESI) calculated for [$C_{35}H_{31}BrFeNaO$] $[M+Na]^+$: 625.0802, found: 625.0819; **IR** (KBr, cm^{-1}) 2924, 2320, 1750, 1510, 1261, 1100, 750, 490; **$[\alpha]_D^{20}$** = -1931.4 (c = 0.1, $CHCl_3$); **HPLC conditions**: Daicel Chiralpak ADH column (98:2 hexane: 2-propanol, 0.5 mL/min, 254 nm); tr (minor) = 12.3 min, tr (major) = 15.8 min; 90% ee.

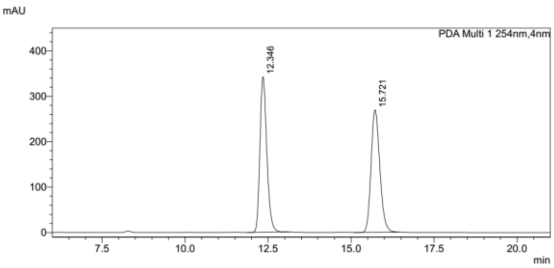

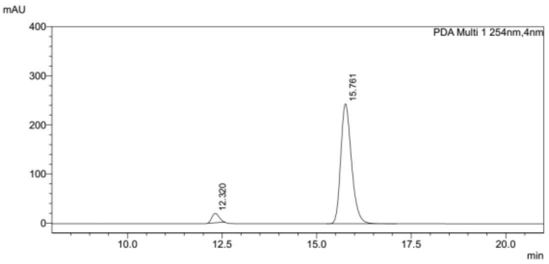

<Peak Table>
PDA Ch1 254nm

Peak#	Ret. Time	Area	Height	Height%	Area%
1	12.346	4949313	342520	55.931	49.969
2	15.721	4955430	269882	44.069	50.031
Total		9904743	612402	100.000	100.000

<Peak Table>
PDA Ch1 254nm

Peak#	Ret. Time	Area	Height	Height%	Area%
1	12.320	256620	19271	7.311	5.052
2	15.761	4822667	244311	92.689	94.948
Total		5079287	263582	100.000	100.000

8.36 (*Sa*,*Sp*)-4-(1-(2-cyclohexylmethoxy)-7-bromonaphthyl)-naphtho[1,2-a]ferrocene. ((*Sa*,*Sp*)-2bi)

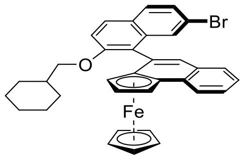

Isolated (-)-**2bi** in 93% yield, >20:1 d.r., as pink solid.

^{1}H NMR (400 MHz, $CDCl_3$) δ 8.10 (d, J = 7.8 Hz, 1H), 7.88 (d, J = 9.0 Hz, 1H), 7.70 – 7.63 (m, 2H), 7.56 – 7.49 (m, 2H), 7.47 – 7.42 (m, 2H), 7.36 (dd, J = 8.8, 2.0 Hz, 1H), 7.25 (s, 1H), 5.37 – 5.34 (m, 1H), 4.18 – 4.16 (m, 1H), 4.15– 4.14 (m (s, 2H), 3.90 (t, J = 8.4 Hz, 1H), 3.79 (s, 5H), 1.96 (d, J = 12.4 Hz, 1H), 1.86 – 1.75 (m, 2H), 1.72 – 1.61 (m, 3H), 1.19– 1.04 (m, 5H); **^{13}C NMR** (100 MHz, $CDCl_3$) δ 154.7, 134.8, 134.7, 133.7, 132.3, 129.4, 129.2, 128.5, 128.1, 127.9, 127.2, 126.9, 126.6, 125.2, 123.2, 122.4, 120.5, 115.2, 86.2, 83.0, 74.8, 69.5, 69.4, 64.6, 61.4, 37.9, 30.2, 29.8, 26.4, 25.7, 25.5; **HRMS** (ESI) calculated for [$C_{35}H_{31}BrFeNaO$] [M+Na]$^+$: 625.0802, found: 625.0803; **IR** (KBr, cm^{-1}) 2924, 2320, 1750, 1508, 1261, 1100, 750, 500; **$[\alpha]_D^{20}$** = -1972.0 (c = 0.1, $CHCl_3$); **HPLC conditions**: Daicel Chiralpak ODH column (98:2 hexane: 2-propanol, 0.5 mL/min, 254 nm); tr (minor) = 10.5 min, tr (major) = 9.4 min; 85% ee.

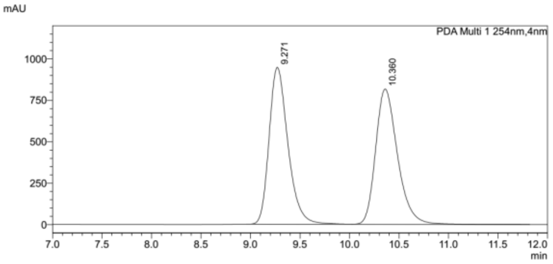

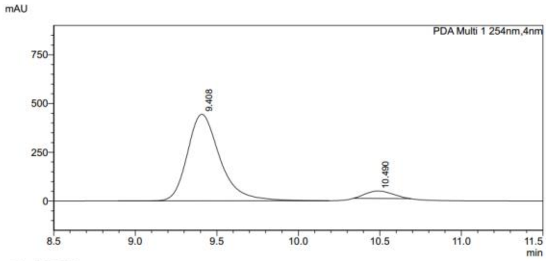

<Peak Table>
PDA Ch1 254nm

Peak#	Ret. Time	Area	Height	Height%	Area%
1	9.271	12539279	947446	53.692	49.980
2	10.360	12549561	817161	46.308	50.020
Total		25088840	1764607	100.000	100.000

<Peak Table>
PDA Ch1 254nm

Peak#	Ret. Time	Area	Height	Height%	Area%
1	9.408	6188620	444549	92.048	93.239
2	10.490	448762	38407	7.952	6.761
Total		6637382	482956	100.000	100.000

8.37 (*Sa*,*Sp*)-4-(1-(2-cyclohexylmethoxy)-7-benzyloxynaphthyl)-naphtho[1,2-a]ferrocene. ((*Sa*,*Sp*)-2bj)

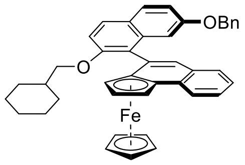

Isolated (-)-**2bj** in 90% yield, >20:1 d.r., as pink solid.

^{1}H NMR (400 MHz, $CDCl_3$) δ 8.10 (d, J = 7.6 Hz, 1H), 7.82 (d, J = 8.8 Hz, 1H), 7.71 (d, J = 8.8 Hz, 1H), 7.64 (d, J = 7.6 Hz, 1H), 7.53 (t, J = 7.0 Hz, 1H), 7.44 (t, J = 7.0 Hz, 1H), 7.34 (d, J = 8.8 Hz, 1H), 7.23 (s, 1H), 7.12 – 7.04 (m, 5H), 7.02– 7.99 (m, 1H), 6.64 (d, J = 2.0 Hz, 1H), 5.33 (d, J = 1.5 Hz, 1H), 4.65– 4.54 (m, 2H), 4.22 – 4.12 (m, 2H), 4.10 (t, J = 2.4 Hz, 1H), 3.87 (t, J = 8.8 Hz, 1H), 3.79 (s, 5H), 1.95 (d, J = 12.0 Hz, 1H), 1.83 – 1.74 (m, 2H), 1.67 – 1.60 (m, 3H), 1.18 – 1.01 (m, 5H); **^{13}C NMR** (100 MHz, $CDCl_3$) δ 156.7, 154.5, 136.7, 134.8, 134.6, 132.6, 129.3, 128.8, 128.5, 128.3, 127.7, 127.6, 127.6, 126.3, 125.1, 124.4, 123.1, 122.1, 116.7, 112.4, 106.1, 86.1, 82.9, 74.7, 69.8, 69.4, 69.3, 64.9, 61.2, 37.9, 30.2, 29.8, 26.5, 25.8, 25.5; **HRMS** (ESI) calculated for [$C_{42}H_{38}FeNaO_2$] [M+Na]$^+$: 653.2114, found: 653.2105; **IR** (KBr, cm^{-1}) 2924, 2374, 1748, 1508, 1261, 1020, 750, 500; **$[\alpha]_D^{20}$** = -1748.5 (c = 0.1, $CHCl_3$); **HPLC conditions**: Daicel Chiralpak ODH column (98:2 hexane: 2-propanol, 0.5 mL/min, 254

nm); tr (minor) = 13.6 min, tr (major) = 12.8 min; 89% ee.

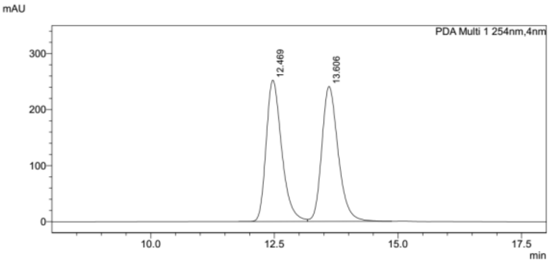

<Peak Table>
PDA Ch1 254nm

Peak#	Ret. Time	Area	Height	Height%	Area%
1	12.469	5505463	252314	51.173	49.762
2	13.606	5558092	240748	48.827	50.238
Total		11063555	493062	100.000	100.000

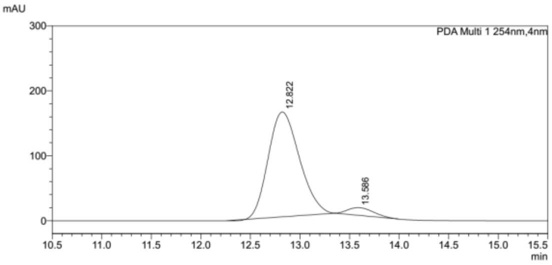

<Peak Table>
PDA Ch1 254nm

Peak#	Ret. Time	Area	Height	Height%	Area%
1	12.822	3547374	161669	93.150	94.298
2	13.586	214502	11889	6.850	5.702
Total		3761876	173558	100.000	100.000

8.38 (*Sa,Sp*)-4-(1-(2-cyclohexylmethoxy)-3-benzyloxynaphthyl)-naphtho[1,2-a]ferrocene. ((*Sa,Sp*)-2bk)

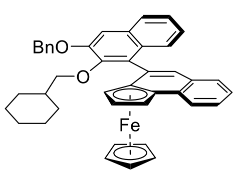

Isolated (-)-**2bk** in 89% yield, >20:1 d.r., as pink solid.

^{1}H NMR (400 MHz, $CDCl_3$) δ 8.08 (d, J = 6.8 Hz, 1H), 7.73 – 7.65 (m, 2H), 7.58 (d, J = 6.0 Hz, 2H), 7.51 (d, J = 6.6 Hz, 1H), 7.44 (s, 3H), 7.37 (s, 2H), 7.33 – 7.27 (m, 1H), 7.24 (s, 1H), 7.15 (d, J = 7.8 Hz, 1H), 7.05 (d, J = 6.4 Hz, 1H), 5.42 – 5.25 (m, 3H), 4.40 (s, 1H), 4.10 (s, 2H), 3.87 (s, 5H), 3.81 (s, 1H), 1.80 – 1.72 (m, 3H), 1.37 – 1.25 (m, 3H), 1.10 – 0.87 (m, 5H); **^{13}C NMR** (100 MHz, $CDCl_3$) δ 151.9, 146.9, 136.8, 135.2, 134.9, 132.2, 130.5, 130.1, 128.7, 128.6, 128.1, 127.8, 126.51, 126.48, 126.3, 125.2, 124.9, 123.7, 123.2, 108.9, 86.8, 82.7, 80.6, 70.8, 69.5, 69.2, 64.7, 61.1, 38.5, 30.2, 30.1, 26.5, 25.8, 25.7; **HRMS** (ESI) calculated for [$C_{42}H_{38}FeNaO_2$] [M+Na]$^+$: 653.2114, found: 653.2102; **IR** (KBr, cm^{-1}) 2924, 2320, 1750, 1508, 1261, 1012, 750, 500; **$[\alpha]_D^{20}$** = -2267.2 (c = 0.1, $CHCl_3$); **HPLC conditions**: Daicel Chiralpak ODH+ODH column (90:10 hexane: 2-propanol, 0.5 mL/min, 254 nm); tr (minor) = 23.0 min, tr (major) = 22.0 min; 89% ee.

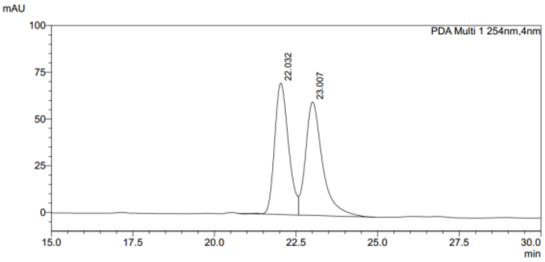

<Peak Table>
PDA Ch1 254nm

Peak#	Ret. Time	Area	Height	Height%	Area%
1	22.032	2071993	70293	53.647	48.507
2	23.007	2199575	60737	46.353	51.493
Total		4271568	131030	100.000	100.000

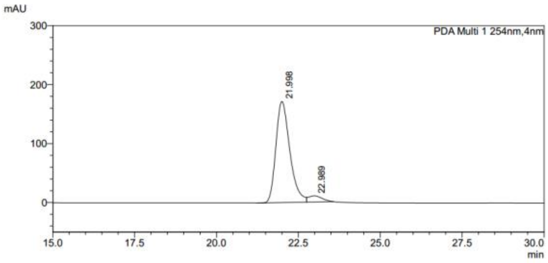

<Peak Table>
PDA Ch1 254nm

Peak#	Ret. Time	Area	Height	Height%	Area%
1	21.998	5073515	171566	94.376	94.477
2	22.989	296611	10225	5.624	5.523
Total		5370126	181790	100.000	100.000

8.39 (*Sa,Sp*)-methyl-4-(1-(2-cyclohexylmethoxy)-6-carboxylate-naphthyl)-naphtho[1,2-a]ferrocene. ((*Sa,Sp*)-2bl)

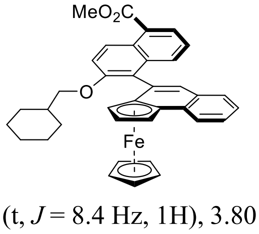

Isolated (-)-**2bl** in 88% yield, >20:1 d.r., as pink solid.

^{1}H NMR (400 MHz, $CDCl_3$) δ 9.03 (d, J = 9.2 Hz, 1H), 8.09 (d, J = 7.6 Hz, 1H), 7.98 – 7.96 (m, 1H), 7.65 (t, J = 9.2 Hz, 2H), 7.57 – 7.49 (m, 2H), 7.49 – 7.42 (m, 1H), 7.30 (s, 1H), 7.20 – 7.16 (m, 1H), 5.35 (d, J = 1.6 Hz, 1H), 4.24 – 4.19 (m, 1H), 4.12 (t, J = 2.4 Hz, 1H), 4.10 – 4.07 (m, 1H), 4.04 (s, 3H), 3.94 (t, J = 8.4 Hz, 1H), 3.80 (s, 5H), 1.98 (d, J = 12.4 Hz, 1H), 1.89 –1.78 (m, 2H), 1.75 –1.63 (m, 3H), 1.21 –1.06 (m, 5H); **^{13}C NMR** (100 MHz, $CDCl_3$) δ 168.4, 154.0, 134.7, 134.1, 134.1, 132.3, 131.4, 128.5, 127.9, 127.7, 127.2, 127.1, 126.5, 125.3, 124.5, 123.3, 123.2, 116.3, 86.7, 83.0, 74.6, 69.5, 69.3, 64.6, 61.2, 52.1, 37.9, 30.2, 29.8, 26.5, 25.7, 25.5; **HRMS** (ESI) calculated for [$C_{37}H_{34}FeNaO_3$] [M+Na]$^+$: 605.1750, found: 605.1758; **IR** (KBr, cm^{-1}) 2924, 2320, 1716, 1508, 1261, 1113, 750, 503; **$[\alpha]_D^{20}$** = -

1683.3 (c = 0.1, $CHCl_3$); **HPLC conditions**: Daicel Chiralpak ADH column (98:2 hexane: 2-propanol, 0.5 mL/min, 254 nm); tr (minor) = 16.6 min, tr (major) = 18.9 min; 90% ee.

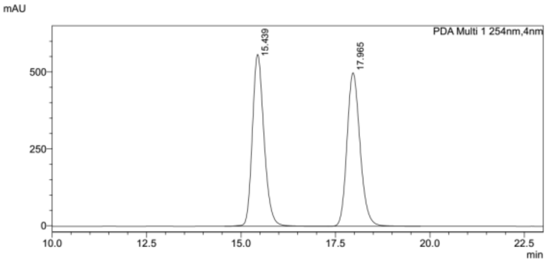

<Peak Table>
PDA Ch1 254nm

Peak#	Ret. Time	Area	Height	Height%	Area%
1	15.439	12003703	558276	52.824	50.018
2	17.965	11995235	498592	47.176	49.982
Total		23998938	1056868	100.000	100.000

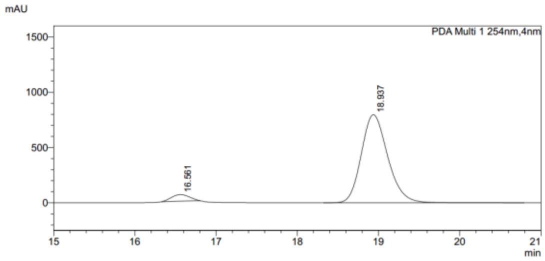

<Peak Table>
PDA Ch1 254nm

Peak#	Ret. Time	Area	Height	Height%	Area%
1	16.561	918081	59573	6.955	4.841
2	18.937	18046158	796984	93.045	95.159
Total		18964239	856557	100.000	100.000

8.40 (*Sa,Sp*)-4-(1-(2-cyclohexylmethoxy)-5,6,7,8-tetrahydronaphthyl)-naphtho[1,2-a]ferrocene. ((*Sa,Sp*)-2bm)

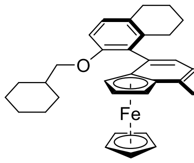

Isolated (-)-**2bm** in 92% yield, >20:1 d.r., as pink solid.

^{1}H NMR (400 MHz, $CDCl_3$) δ 8.04 (d, *J* = 7.6 Hz, 1H), 7.62 (d, *J* = 7.2 Hz, 1H), 7.49 (t, *J* = 7.2 Hz, 1H), 7.42 (t, *J* = 7.2 Hz, 1H), 7.15 (d, *J* = 8.4 Hz, 1H), 7.10 (s, 1H), 6.96 (d, *J* = 8.4 Hz, 1H), 5.33 (s, 1H), 4.44 (s, 1H), 4.20 (s, 1H), 4.05 – 4.02 (m, 1H), 3.80 (s, 5H), 3.74 (t, *J* = 8.4 Hz, 1H), 2.89 – 2.76 (m, 2H), 2.52 – 2.41 (m, 1H), 2.23 – 2.14 (m, 1H), 1.95 (d, *J* = 12.4 Hz, 1H), 1.76 – 1.62 (m, 8H), 1.50 – 1.45 (m, 1H), 1.19 – 0.98 (m, 5H); **^{13}C NMR** (100 MHz, $CDCl_3$) δ 154.7, 137.7, 135.8, 134.4, 132.5, 129.3, 128.9, 128.4, 128.3, 126.4, 126.1, 125.1, 123.1, 110.0, 86.2, 82.8, 74.0, 69.6, 69.1, 64.0, 61.1, 37.6, 30.2, 29.8, 29.3, 27.4, 26.5, 25.7, 25.5, 23.1, 23.0; **HRMS** (ESI) calculated for $[C_{35}H_{36}FeNaO]$ $[M+Na]^+$: 551.2008, found: 551.2014; **IR** (KBr, cm^{-1}) 2926, 2320, 1748, 1508, 1261, 1022, 750, 500; **$[\alpha]_D^{20}$** = -2146.0 (c = 0.1, $CHCl_3$); **HPLC conditions**: Daicel Chiralpak ADH column (98:2 hexane: 2-propanol, 0.5 mL/min, 254 nm); tr (minor) = 7.4 min, tr (major) = 8.5 min; 86% ee.

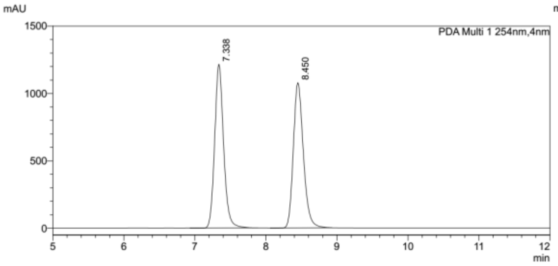

<Peak Table>
PDA Ch1 254nm

Peak#	Ret. Time	Area	Height	Height%	Area%
1	7.338	10631132	1217521	53.029	50.154
2	8.450	10565952	1078421	46.971	49.846
Total		21197085	2295942	100.000	100.000

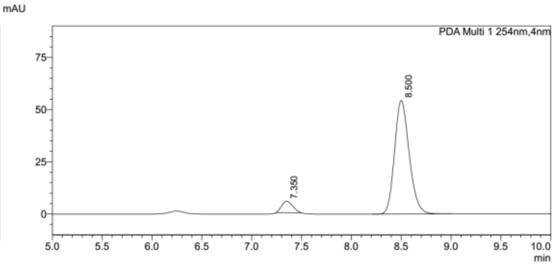

<Peak Table>
PDA Ch1 254nm

Peak#	Ret. Time	Area	Height	Height%	Area%
1	7.350	42476	5480	9.144	7.068
2	8.500	558478	54457	90.856	92.932
Total		600954	59937	100.000	100.000

8.41 (*Sa,Sc,Sp*)-methyl-(4-(1-(2-methoxy)naphthyl)-6-yl)propanoate-naphtho[1,2-a]ferrocene. ((*Sa,Sc,Sp*)-2bn)

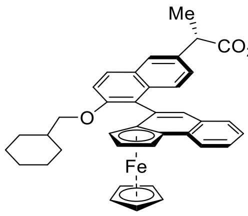

Isolated (-)-**2bn** in 92% yield, >20:1 d.r., as pink solid.

^{1}H NMR (400 MHz, $CDCl_3$) δ 8.08 (d, *J* = 7.6 Hz, 1H), 7.90 (d, *J* = 9.2 Hz, 1H), 7.73 (s, 1H), 7.64 (d, *J* = 7.6 Hz, 1H), 7.52 (d, *J* = 8.8 Hz, 2H), 7.43 (t, *J* = 7.2 Hz, 1H), 7.24 (d, *J* = 2.8 Hz, 2H), 7.15 – 7.08 (m, 1H), 5.34 (d, *J* = 1.2 Hz, 1H), 4.18 (q, *J* = 5.6 Hz, 2H), 4.12 (t, *J* = 2.4 Hz, 1H), 3.89 (t, *J* = 8.4 Hz, 1H), 3.83 (d, *J* = 7.6 Hz, 1H), 3.80 (s, 5H), 3.64 (s, 3H), 1.96 (d, *J* = 12.0 Hz, 1H), 1.85 – 1.74 (m, 2H), 1.71 – 1.60 (m, 3H), 1.54 (d, *J* = 7.2 Hz, 3H), 1.19 – 1.03 (m, 5H); **^{13}C NMR** (100 MHz, $CDCl_3$) δ 175.1, 154.0, 135.5, 135.4, 134.7, 134.4, 132.6, 132.4, 129.1, 128.9,

128.5, 127.6, 126.6 126.4, 126.0, 125.9, 125.8, 125.2, 123.2, 123.1, 115.3, 86.5, 83.0, 75.0, 69.4, 69.2, 64.8, 61.1, 52.0, 45.2, 37.9, 30.2, 29.8, 26.5, 25.7, 25.5, 18.6, 18.4; **HRMS** (ESI) calculated for [$C_{39}H_{38}FeNaO_3$] [M+Na]$^+$: 633.2063, found: 633.2057; **IR** (KBr, cm^{-1}) 2924, 2312, 1738, 1508, 1261, 1100, 750, 501; **[α]$_D^{20}$** = -2095.4 (c = 0.1, $CHCl_3$).

8.42 (*Sa,Sp*)-4-(1-(2-methyl)naphthyl)-7-trifluoromethyl-naphtho[1,2-a]ferrocene. ((*Sa,Sp*)-2bo)

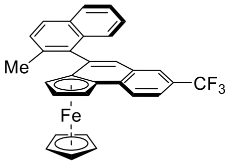

Isolated (-)-**2bo** in 99% yield, >20:1 d.r., as pink solid.

^{1}H NMR (500 MHz, $CDCl_3$) δ 8.27 – 8.20 (m, 2H), 8.05 – 7.98 (m, 2H), 7.95 (d, *J* = 8.5 Hz, 1H), 7.81 (d, *J* = 8.0 Hz, 1H), 7.63 – 7.55 (m, 2H), 7.51 (d, *J* = 8.5 Hz, 1H), 7.29 (s, 1H), 5.52 (d, *J* = 1.0 Hz, 1H), 4.38 (d, *J* = 16.5 Hz, 2H), 3.86 (s, 5H), 2.15 (s, 3H); **^{13}C NMR** (125 MHz, $CDCl_3$) δ 139.8, 137.9, 135.0, 134.1, 132.3 (d, *J* = 6.1 Hz), 131.7, 128.9, 128.1, 127.7, 127.5, 127.3, 127.0, 126.7, 126.1, 125.8 (d, *J* = 1.4 Hz), 125.7 (d, *J* = 4.0 Hz), 125.1, 123.7, 122.8 (d, *J* = 3.5 Hz), 87.6, 81.6, 70.4, 69.4, 64.9, 62.0, 20.6; **HRMS** (ESI) calculated for [$C_{30}H_{21}F_3Fe$] [M+Na]$^+$: 517.0838, found: 517.0836; **IR** (KBr, cm^{-1}) 2924, 2320, 1508, 1388, 1120, 916, 748, 490; **[α]$_D^{20}$** = -1916.1 (c = 0.1, $CHCl_3$); **HPLC conditions**: Daicel Chiralpak IC column (98:2 hexane: 2-propanol, 0.5 mL/min, 254 nm); tr (minor) = 8.0 min, tr (major) = 7.7 min; 78% ee.

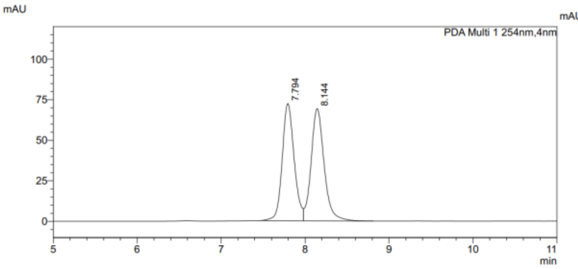

<Peak Table>
PDA Ch1 254nm

Peak#	Ret. Time	Area	Height	Height%	Area%
1	7.794	715300	72305	51.154	49.099
2	8.144	741547	69042	48.846	50.901
Total		1456848	141347	100.000	100.000

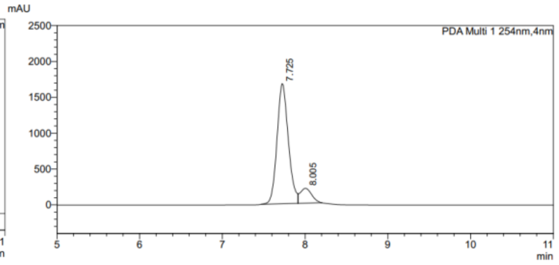

<Peak Table>
PDA Ch1 254nm

Peak#	Ret. Time	Area	Height	Height%	Area%
1	7.725	15979889	1678158	88.964	88.987
2	8.005	1977719	208181	11.036	11.013
Total		17957608	1886339	100.000	100.000

8.43 (*Sp*)-4-(1-phenyl)naphtho[1,2-a]ferrocene. ((*Sp*)-6)

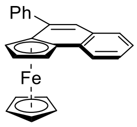

Isolated (-)-**6** in 99% yield as pink solid.

^{1}H NMR (400 MHz, $CDCl_3$) δ 8.03 – 7.99 (m, 1H), 7.88 – 7.84 (m, 2H), 7.69 – 7.65 (m, 1H), 7.56 – 7.50 (m, 2H), 7.49 – 7.39 (m, 3H), 7.23 (s, 1H), 5.34 (dd, *J* = 2.4, 1.0 Hz, 1H), 4.90 (dd, *J* = 2.4, 1.0 Hz, 1H), 4.27 (t, *J* = 2.4 Hz, 1H), 3.75 (s, 5H); **^{13}C NMR** (100 MHz, $CDCl_3$) δ 140.7, 140.6, 134.3, 132.4, 128.8, 128.6, 128.0, 127.6, 126.6, 125.4, 124.0, 123.1, 83.4, 83.3, 69.7, 69.5, 64.2, 61.8; **IR** (KBr, cm^{-1}) 3000, 2340, 1747, 1508, 1275, 750, 480; **HPLC conditions**: Daicel Chiralpak ODH column (90:10 hexane: 2-propanol, 0.5 mL/min, 254 nm); tr (minor) = 11.2 min, tr (major) = 12.7 min; 22% ee.

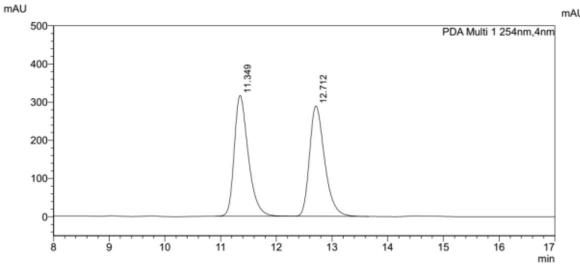

<Peak Table>
PDA Ch1 254nm

Peak#	Ret. Time	Area	Height	Height%	Area%
1	11.349	5530211	316408	52.244	50.317
2	12.712	5460433	289229	47.756	49.683
Total		10990645	605638	100.000	100.000

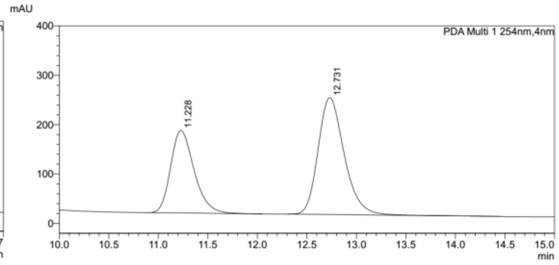

<Peak Table>
PDA Ch1 254nm

Peak#	Ret. Time	Area	Height	Height%	Area%
1	11.228	2762111	167298	41.394	38.774
2	12.731	4361441	236861	58.606	61.226
Total		7123552	404159	100.000	100.000

8.44 (*Sa*)-4-(2-methoxynaphthalen-1-yl)-2,3-dihydro-1H-cyclopenta[a]naphthalene ((*Sa*)-7a)

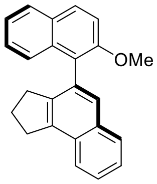

Isolated (-)-**7a** in 99% yield as white solid.

^{1}H NMR (400 MHz, $CDCl_3$) δ 7.91 – 7.82 (m, 4H), 7.63 (s, 1H), 7.53 – 7.48 (m, 1H), 7.45 – 7.41 (m, 1H), 7.39 – 7.35 (m, 2H), 7.34– 7.24 (m, 2H), 3.82 (s, 3H), 3.37 (t, *J* = 7.4 Hz, 2H), 2.86 – 2.75 (m, 1H), 2.64 – 2.54 (m, 1H), 2.25 – 2.07 (m, 2H); **^{13}C NMR** (100 MHz, $CDCl_3$) δ 153.9, 142.2, 139.5, 133.5, 132.9, 131.9, 130.0, 129.1, 129.0, 128.5, 128.2, 127.8, 126.3, 125.7, 125.2, 124.8, 124.4, 124.2, 123.5, 113.6, 56.6, 32.8, 31.5, 24.2; **HRMS** (ESI) calculated for $[C_{24}H_{20}NaO]$ $[M+Na]^+$: 347.1406, found: 347.1405; **IR** (KBr, cm^{-1}) 3005, 2318, 1750, 1508, 1261, 1093, 750, 490; **$[\alpha]_D^{20}$** = -120.9 (c = 0.1, $CHCl_3$); **HPLC conditions**: Daicel Chiralpak ADH column (98:2 hexane: 2-propanol, 0.5 mL/min, 254 nm); tr (minor) = 11.8 min, tr (major) = 10.8 min; 80% ee.

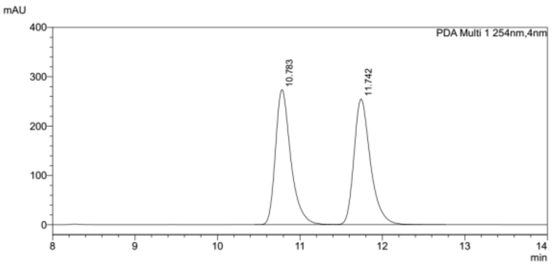

<Peak Table>
PDA Ch1 254nm

Peak#	Ret. Time	Area	Height	Height%	Area%
1	10.783	3536987	273933	51.812	49.933
2	11.742	3546502	254773	48.188	50.067
Total		7083489	528705	100.000	100.000

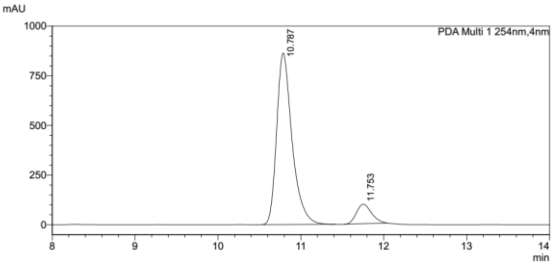

<Peak Table>
PDA Ch1 254nm

Peak#	Ret. Time	Area	Height	Height%	Area%
1	10.787	11160627	863619	89.766	89.915
2	11.753	1251818	98460	10.234	10.085
Total		12412446	962078	100.000	100.000

8.45 (*Sa*)-4-(2-(cyclohexylmethoxy)naphthalen-1-yl)-2,3-dihydro-1H-cyclopenta[a]naphthalene. ((*Sa*)-7b)

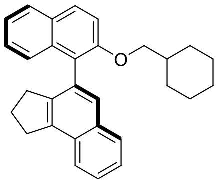

Isolated (-)-**7b** in 99% yield as white solid.

^{1}H NMR (400 MHz, $CDCl_3$) δ 7.81 – 7.40 (m, 4H), 7.54 (s, 1H), 7.46 – 7.40 (m, 1H), 7.39 – 7.29 (m, 2H), 7.28 – 7.13 (m, 3H), 3.75 – 3.65 (m, 2H), 3.27 (t, *J* = 7.4 Hz, 2H), 2.79 – 2.68 (m, 1H), 2.58 – 2.47 (m, 1H), 2.11 – 2.02 (m, 2H), 1.45 (d, *J* = 8.0 Hz, 7H), 1.03 – 0.85 (m, 3H), 0.74 – 0.69 (m, 2H); **^{13}C NMR** (100 MHz, $CDCl_3$) δ 153.7, 142.3, 139.3, 133.6, 133.0, 132.2, 129.9, 129.2, 128.9, 128.5, 128.3, 127.9, 126.2, 125.5, 125.4, 125.1, 124.6, 124.4, 123.5, 115.6, 75.4, 37.7, 33.0, 31.6, 29.7, 29.6, 26.4, 25.7, 25.7, 24.3; **HRMS** (ESI) calculated for $[C_{30}H_{30}NaO]$ $[M+Na]^+$: 429.2189, found: 429.2187; **IR** (KBr, cm^{-1}) 2924, 2318, 1750, 1508, 1261, 1018, 750, 490; **$[\alpha]_D^{20}$** = -94.3 (c = 0.1, $CHCl_3$); **HPLC conditions**: Daicel Chiralpak ADH column (98:2 hexane: 2-propanol, 0.5 mL/min, 254 nm); tr (minor) = 12.3 min, tr (major) = 15.6 min; 95% ee.

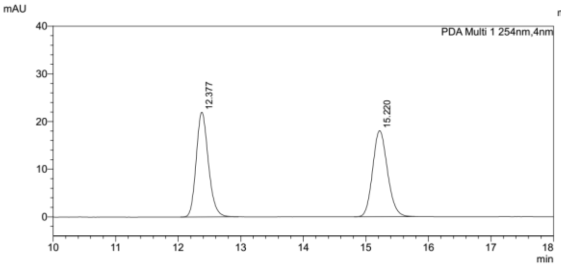

<Peak Table>
PDA Ch1 254nm

Peak#	Ret. Time	Area	Height	Height%	Area%
1	12.377	297659	21995	54.884	50.048
2	15.220	297088	18080	45.116	49.952
Total		594747	40075	100.000	100.000

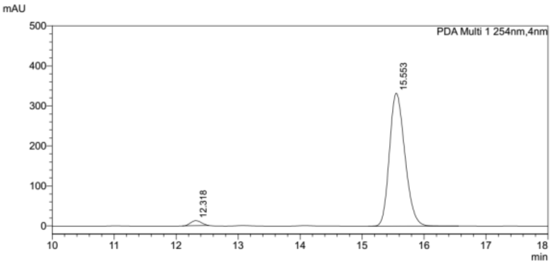

<Peak Table>
PDA Ch1 254nm

Peak#	Ret. Time	Area	Height	Height%	Area%
1	12.318	152935	12649	3.664	2.575
2	15.553	5785654	332550	96.336	97.425
Total		5938589	345198	100.000	100.000

8.46 (*Sa,Sp*)-4-(1-(2-ol)naphthyl)naphtho[1,2-a]ferrocene. ((*Sa,Sp*)-3)

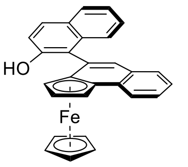

Isolated (-)-**3** in 86% yield as pink solid.

Isolated *ent*-**3** in 87% yield as pink solid

^{1}H NMR (500 MHz, $CDCl_3$) δ 8.12 (d, *J* = 8.0 Hz, 1H), 7.89 (d, *J* = 9.0 Hz, 1H), 7.83 (d, *J* = 78.0 Hz, 1H), 7.69 (d, *J* = 8.0 Hz, 1H), 7.58 (t, *J* = 7.0 Hz, 1H), 7.51 – 7.45 (m, 1H), 7.43 (d, *J* = 9.0 Hz, 1H), 7.35 (s, 1H), 7.32 – 7.26 (m, 2H), 7.21 (dd, *J* = 8.5, 6.5 Hz, 1H), 6.56 (s, 1H), 5.46 (d, *J* = 1.5 Hz, 1H), 4.35 (d, *J* = 1.0 Hz, 1H), 4.23 (t, *J* = 2.5 Hz, 1H), 3.86 (s, 5H); **^{13}C NMR** (125 MHz, $CDCl_3$) δ 150.8, 134.9, 133.6, 133.1, 131.6, 129.8, 129.0, 128.0, 127.8, 127.5, 126.3, 125.7, 125.2, 123.3, 123.3, 118.5, 117.4, 85.4, 83.4, 69.9, 69.4, 64.6, 61.9; **HRMS** (ESI) calculated for $[C_{28}H_{20}NaO]$ $[M+Na]^+$: 451.0756, found: 451.0745; **IR** (KBr, cm^{-1}) 3366, 2947, 2374, 1508, 1261, 1020, 750, 501; **$[\alpha]_D^{20}$** = over range (c = 0.33, $CHCl_3$); **HPLC conditions**: Daicel Chiralpak ADH column (80:20 hexane: 2-propanol, 0.5 mL/min, 254 nm); **6a** [tr (minor) = 15.6 min, tr (major) = 36.8 min; 92% ee]; *ent*-**6a** [tr (minor) = 35.7 min, tr (major) = 15.6 min; 93% ee].

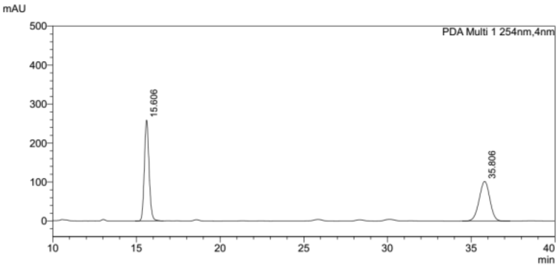

<Peak Table>

PDA Ch1 254nm

Peak#	Ret. Time	Area	Height	Height%	Area%
1	15.606	5062785	258796	71.781	52.866
2	35.806	4513914	101742	28.219	47.134
Total		9576699	360538	100.000	100.000

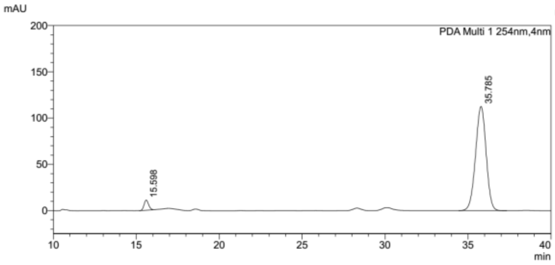

<Peak Table>

PDA Ch1 254nm

Peak#	Ret. Time	Area	Height	Height%	Area%
1	15.598	209518	10957	8.867	4.035
2	35.785	4983096	112607	91.133	95.965
Total		5192613	123564	100.000	100.000

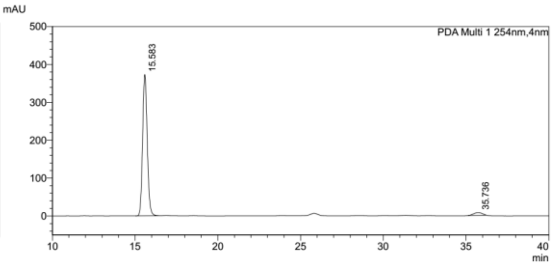

<Peak Table>

PDA Ch1 254nm

Peak#	Ret. Time	Area	Height	Height%	Area%
1	15.583	7241172	373304	98.073	96.485
2	35.736	263838	7333	1.927	3.515
Total		7505009	380638	100.000	100.000

9. Gram-scale synthesis.

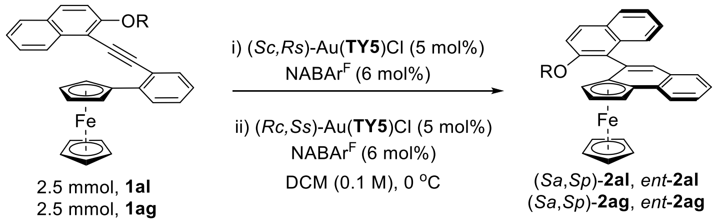

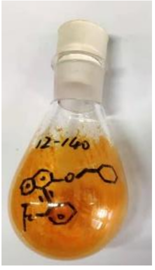

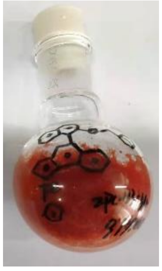

In a dried Schlenk tube, after the solution of Au(**TY5**)Cl (5 mol%, 103 mg) and NaBAr^F (6 mol%, 132.9 mg, cas: 79060-88-1) in DCM (4.0 mL) was stirred at room temperature for 15 min. Then the above catalyst solution was added to the solution of **1al** or **1ag** (2.5 mmol) in DCM (21 mL) at 0 °C. The reaction was determined by TLC analysis, after the **1al** or **1ag** was consumed completely. Solvent was removed in a rotary evaporator, purified by flash column chromatography on silica gel (Hexane/DCM= 10:1) to afford the desired product **2al** or *ent*-**2al** and on silica gel (Hexane/DCM= 3:1) to afford the desired product **2ag** or *ent*-**2ag**.

10. Synthetic applications of the products.

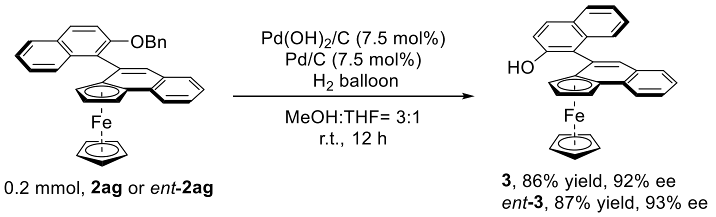

In a 10 ml Schlenk tube, after the solution of $Pd(OH)_2/C$ (7.5 mol%, 21 mg, 10% on carbon, wetted with ca. 50% water, cas: 12135-22-7, *j&k chemical*), Pd/C (7.5 mol%, 16 mg, 10% on activated carbon, reduced, dry powder, cas: 7440-05-3, *j&k chemical*) and **2ag** or *ent*-**2ag** (0.2 mmol, 103.7 mg) in MeOH (3 mL) and THF (1 mL) was stirred at room temperature for 12 h. The reaction was determined by TLC analysis, after the **2ag** or *ent*-**2ag** was consumed completely. Solvent was removed in a rotary evaporator, purified by flash column chromatography on silica gel (Hexane/DCM= 3:1) to afford the desired product **3** or *ent*-**3**.

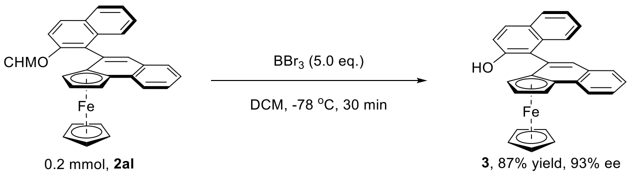

To a solution of **2al** (0.2 mmol, 104 mg) in 4 mL dry DCM at -78 °C for 10 min under N_2, was

added BBr_3 (5.0 equiv, 0.1 ml, cas: 10294-33-4, Purity：99.9%, *Energy Chemical*); The mixture was stirred at -78 °C for 30 min. The organic layers were concentrated, and purified by flash chromatography (Hexane: EA= 10:1) to afford the desired product **3**.

11. ^{1}H and ^{13}C NMR Spectra for new compouds

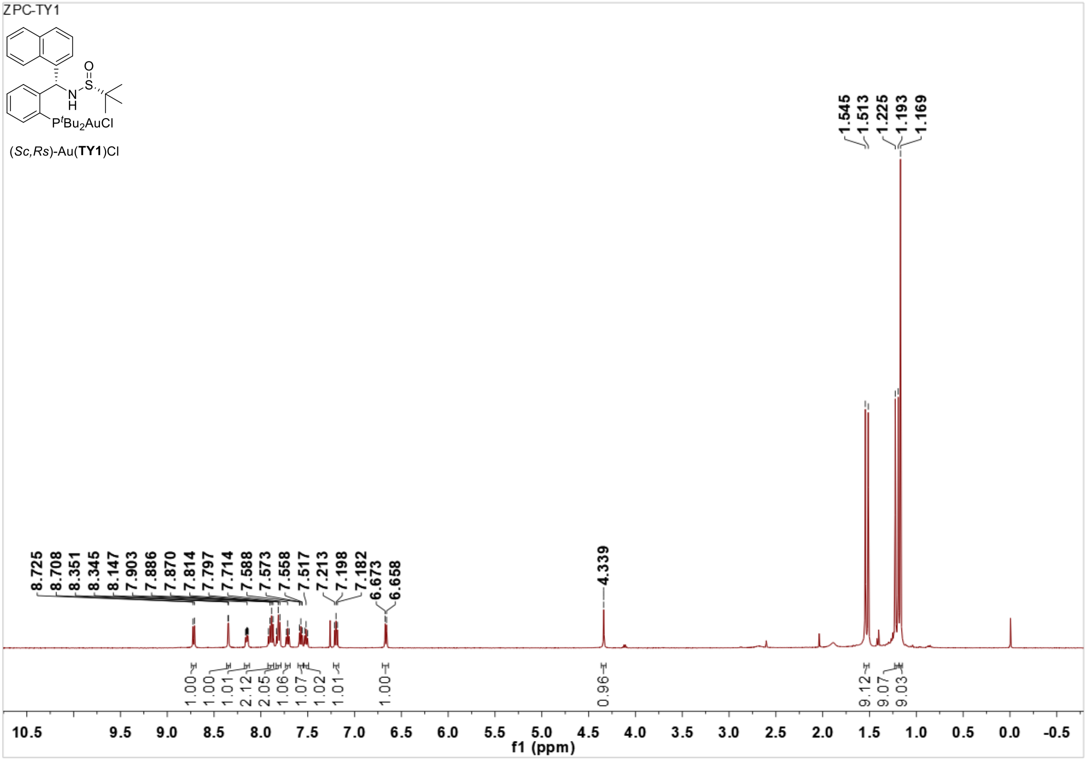

Supplementary Figure 1. ^{1}H NMR (500 MHz, $CDCl_3$) spectra for compound(*Sc,Rs*)-Au(**TY1**)Cl

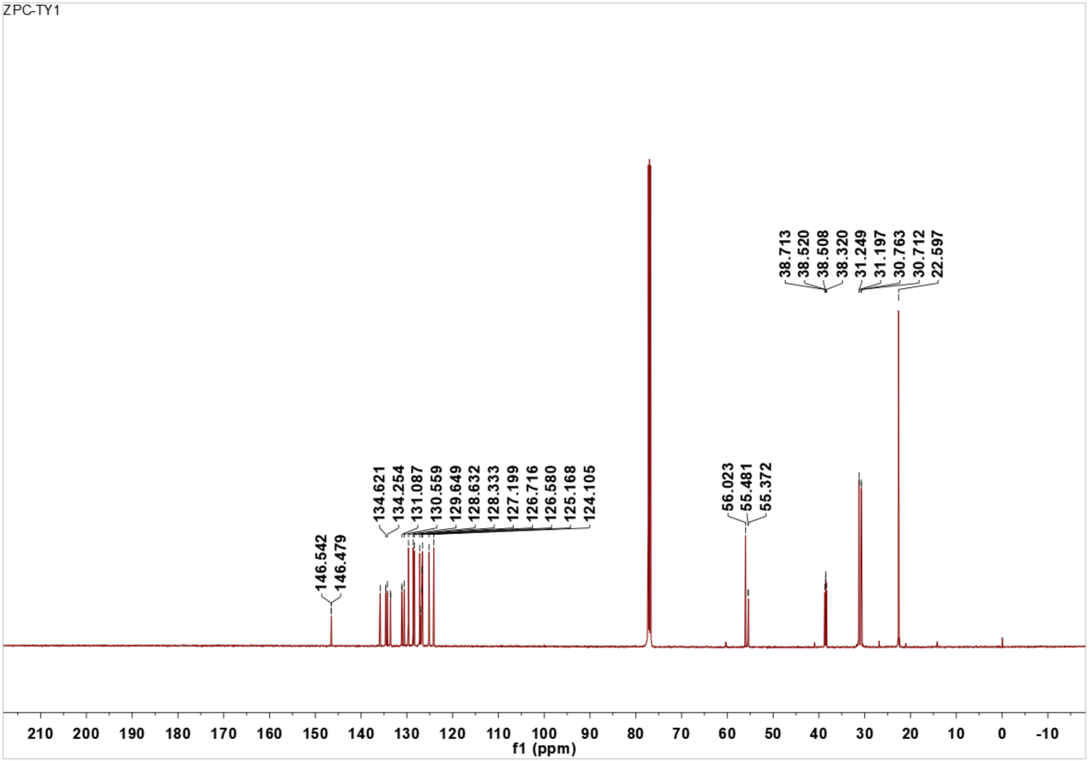

Supplementary Figure 2. ^{13}C NMR (500 MHz, $CDCl_3$) spectra for compound (*Sc,Rs*)-Au(**TY1**)Cl

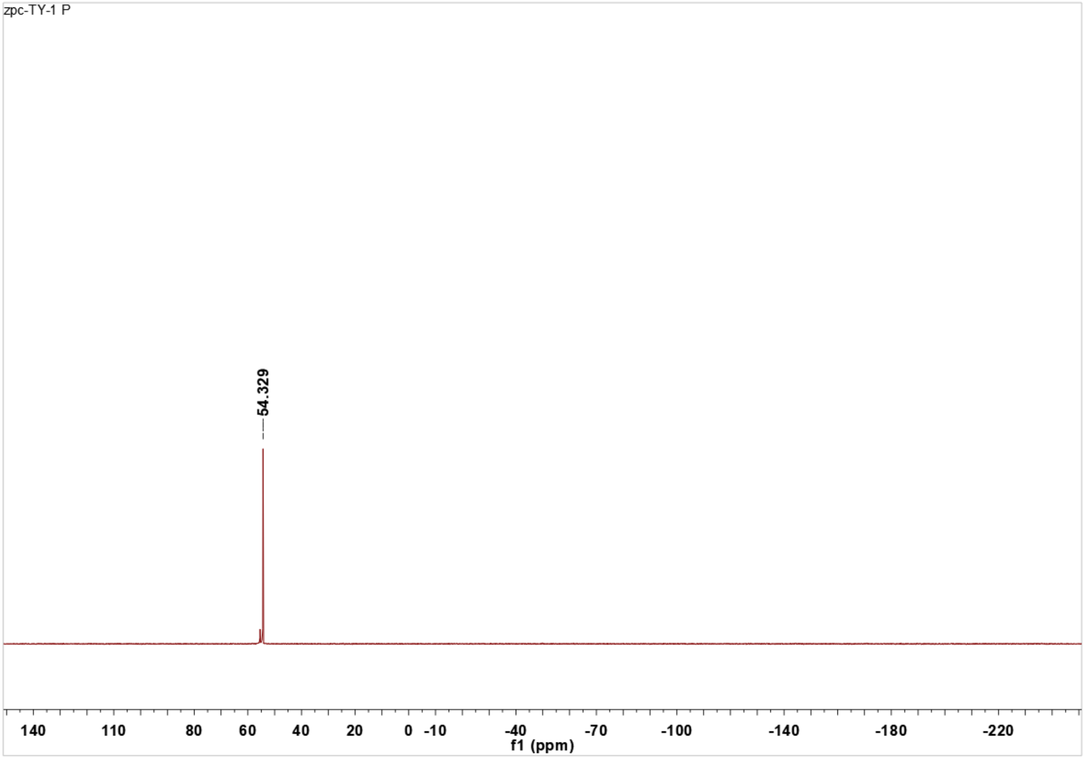

Supplementary Figure 3. ^{31}P NMR (500 MHz, $CDCl_3$) spectra for compound (*Sc,Rs*)-Au(**TY1**)Cl

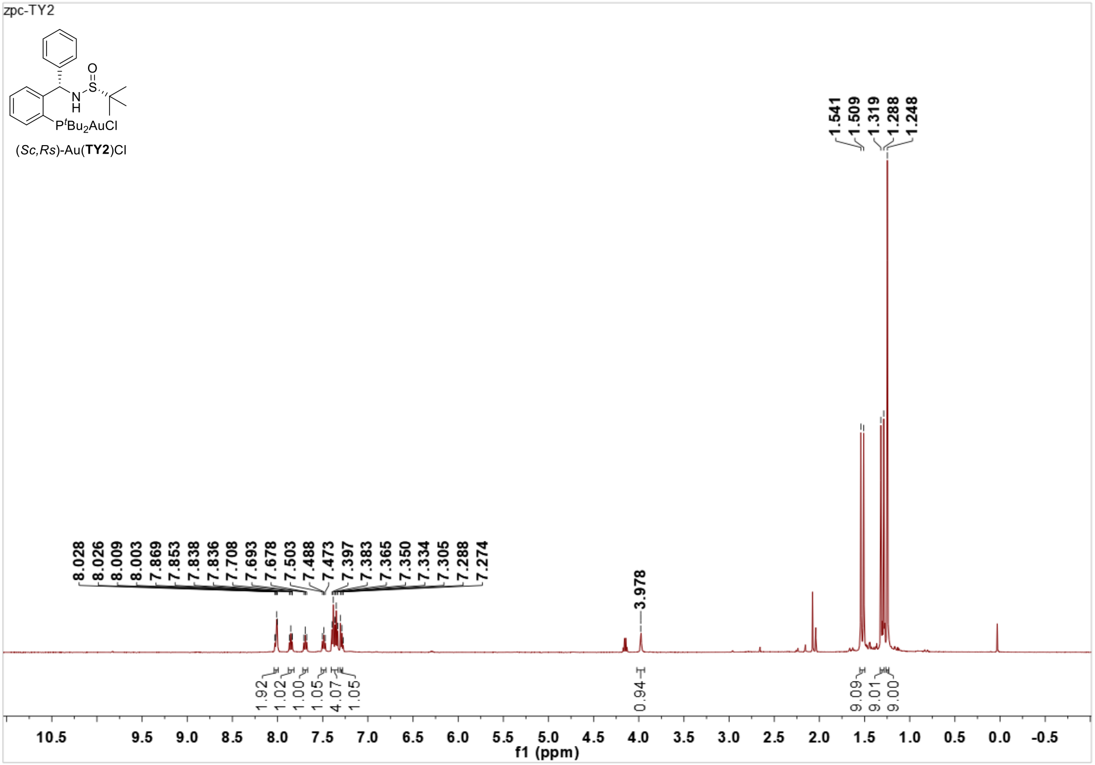

Supplementary Figure 4. ^{1}H NMR (500 MHz, $CDCl_3$) spectra for compound (*Sc,Rs*)-Au(**TY2**)Cl

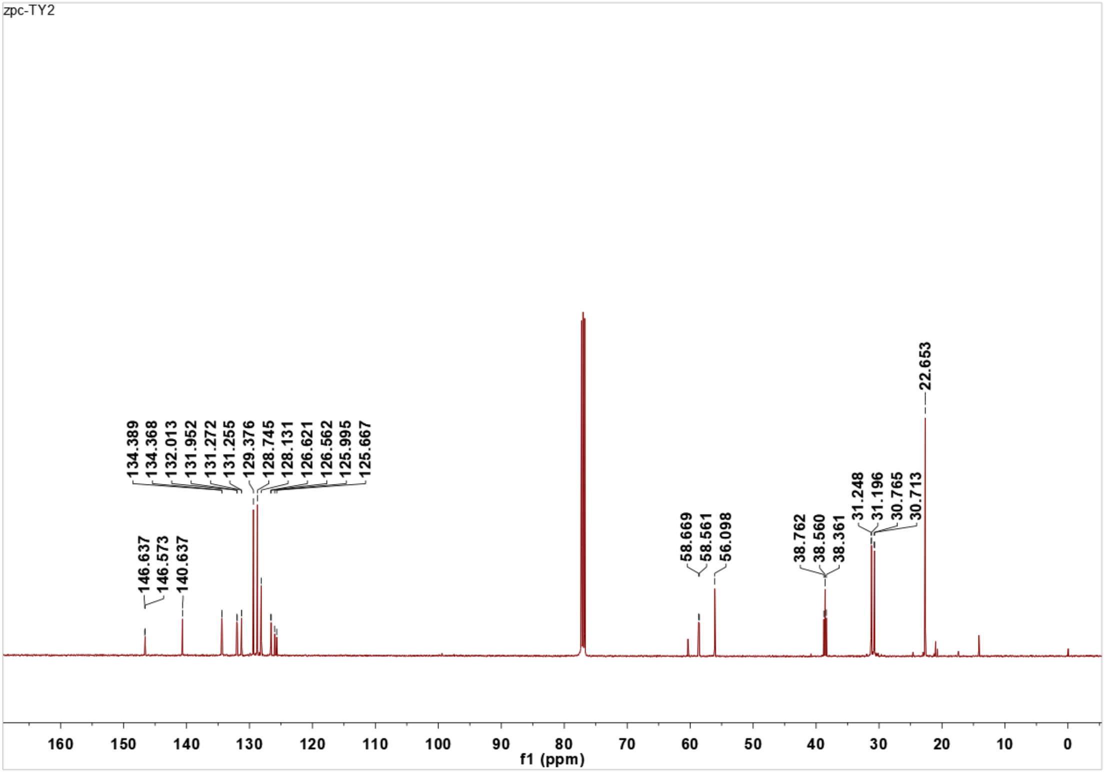

Supplementary Figure 5. ^{13}C NMR (500 MHz, $CDCl_3$) spectra for compound (*Sc,Rs*)-Au(**TY2**)Cl

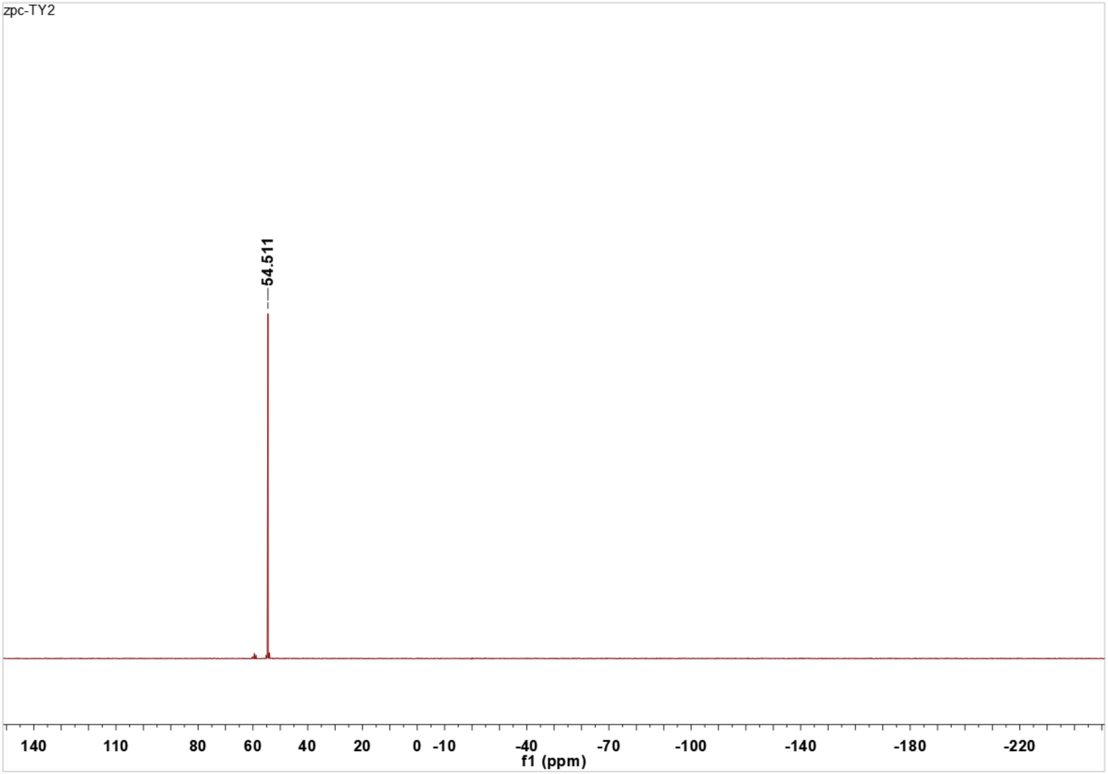

Supplementary Figure 6. ^{31}P NMR (500 MHz, $CDCl_3$) spectra for compound (*Sc,Rs*)-Au(**TY2**)Cl

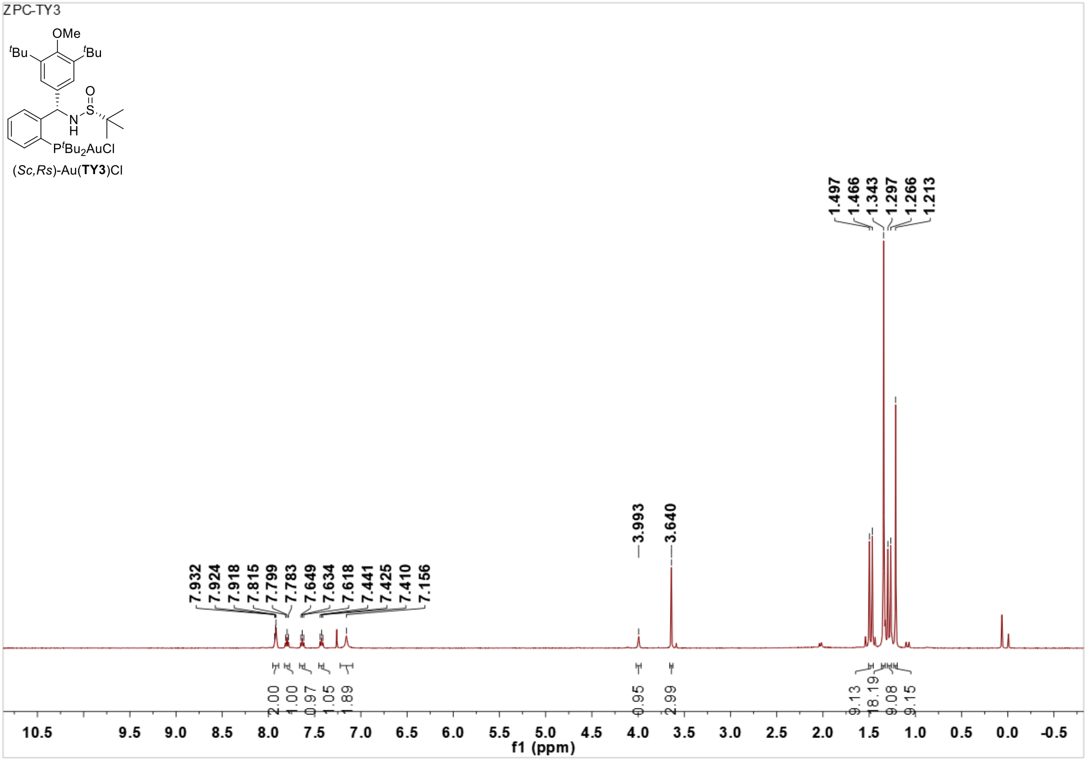

Supplementary Figure 7. ^{1}H NMR (500 MHz, $CDCl_3$) spectra for compound (*Sc,Rs*)-Au(**TY3**)Cl

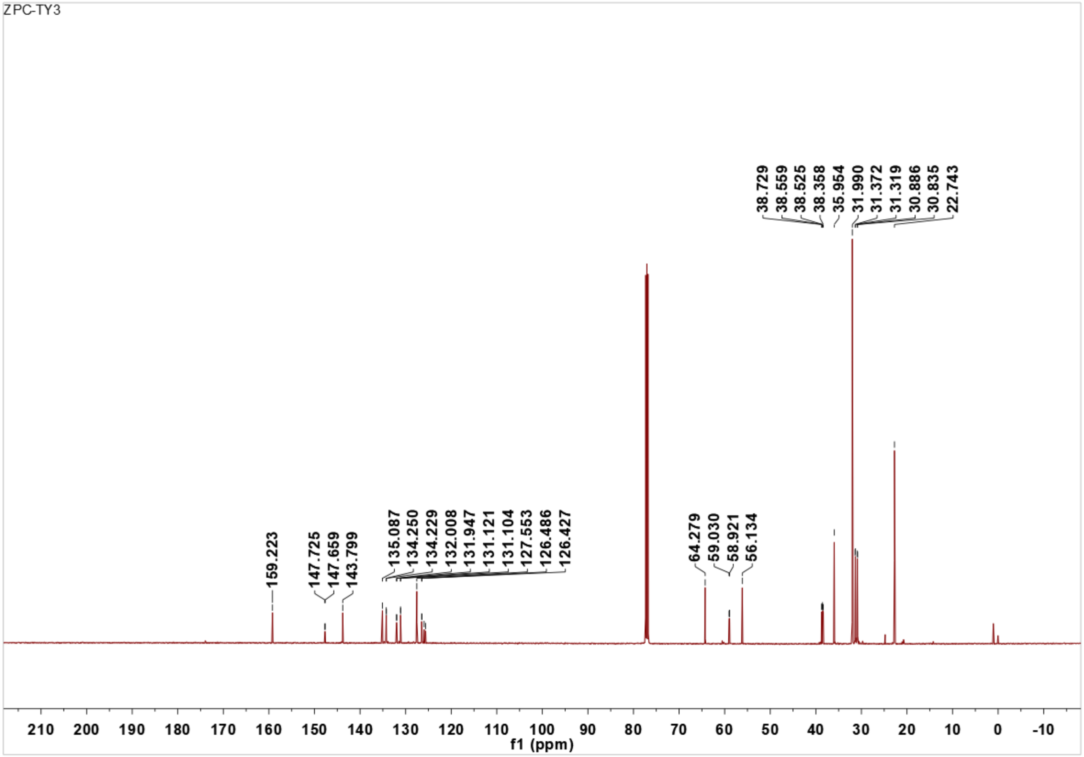

Supplementary Figure 8. ^{13}C NMR (500 MHz, $CDCl_3$) spectra for compound (*Sc,Rs*)-Au(**TY3**)Cl

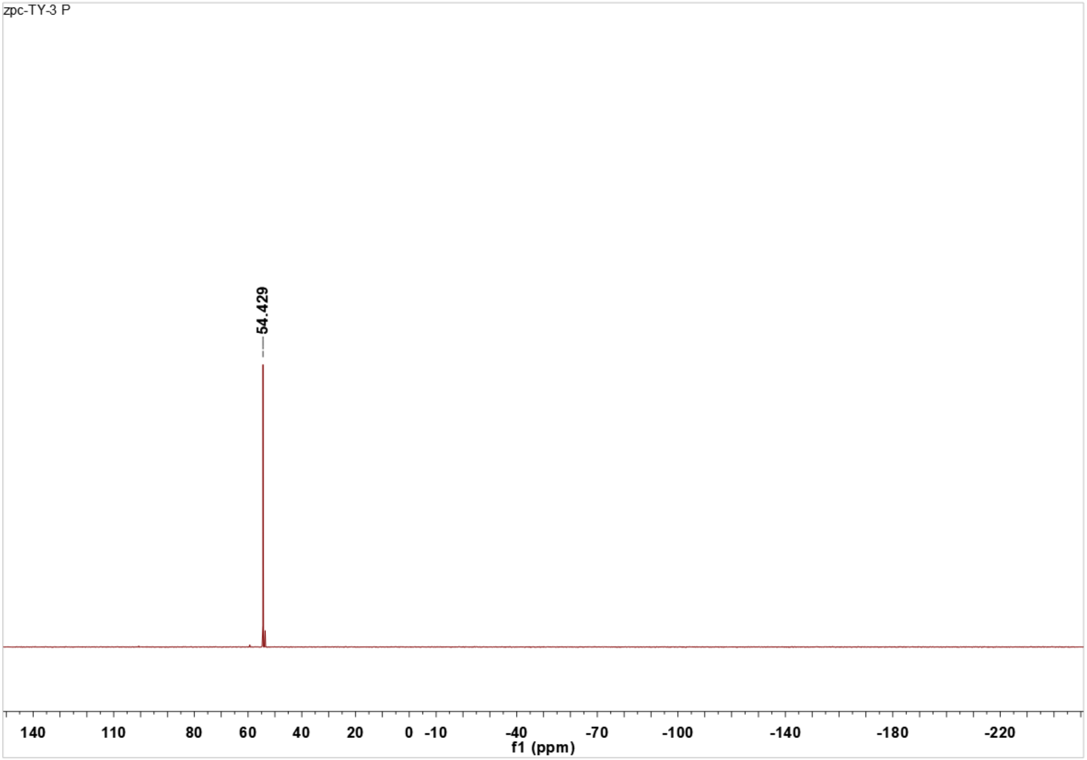

Supplementary Figure 9. ^{31}P NMR (500 MHz, $CDCl_3$) spectra for compound (*Sc,Rs*)-Au(**TY3**)Cl

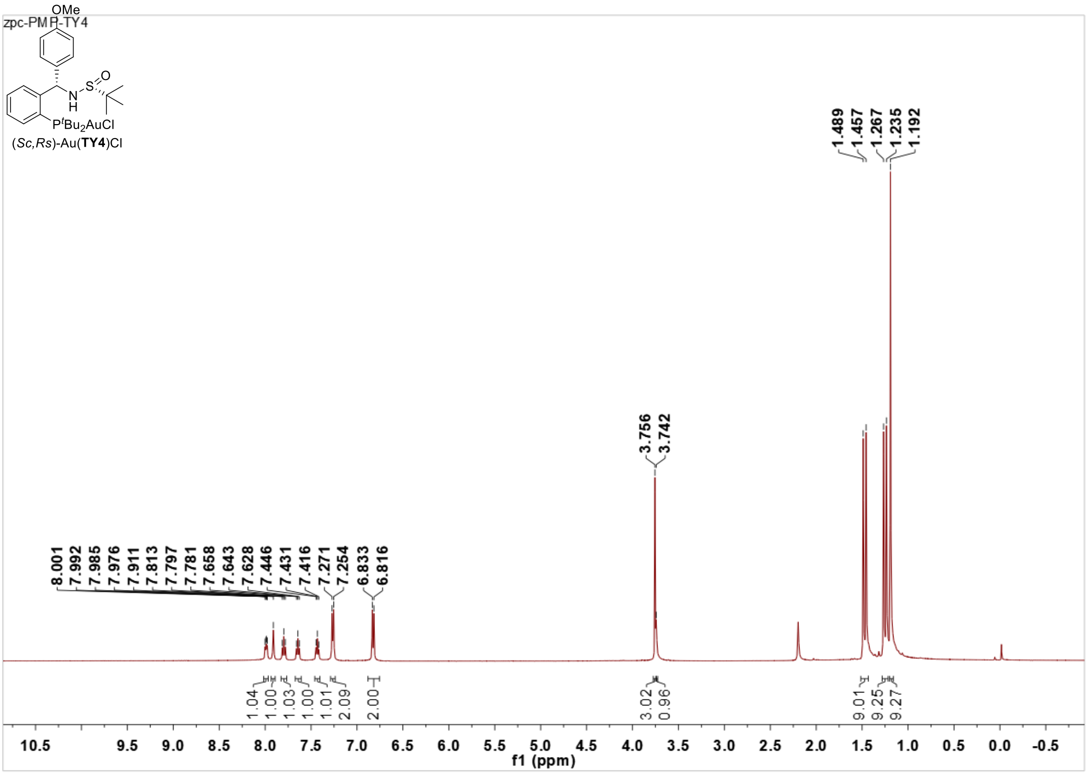

Supplementary Figure 10. ^{1}H NMR (500 MHz, $CDCl_3$) spectra for compound (*Sc,Rs*)-Au(**TY4**)Cl

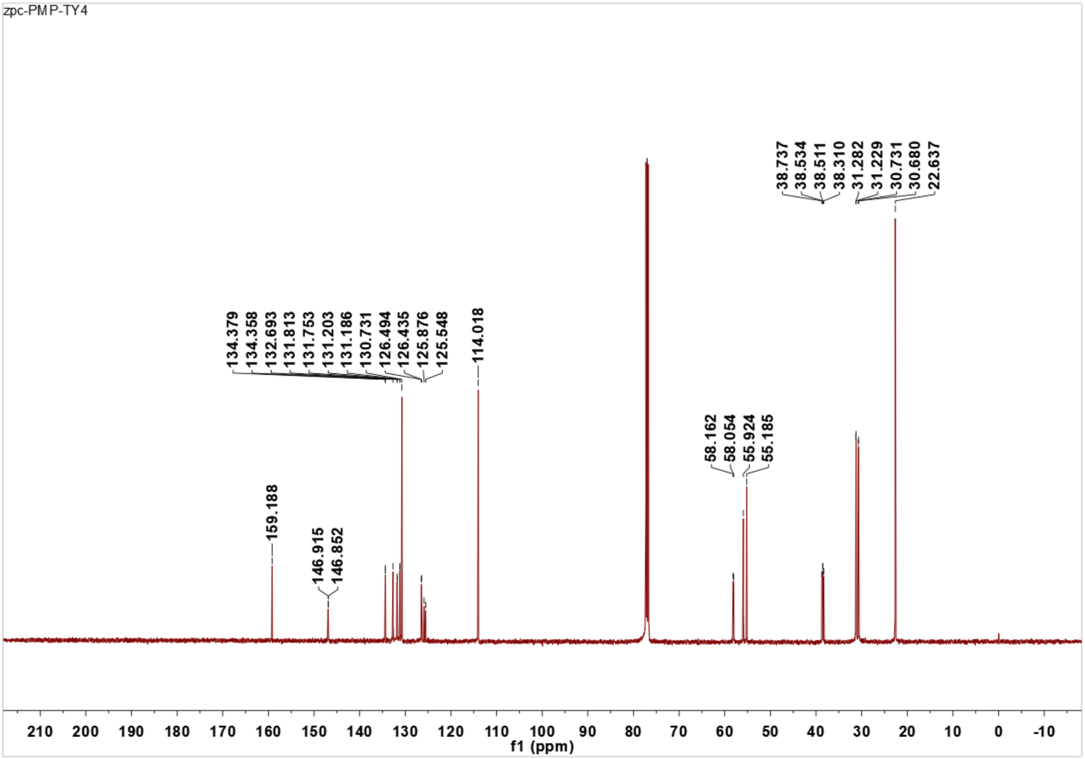

Supplementary Figure 11. ^{13}C NMR (500 MHz, $CDCl_3$) spectra for compound (*Sc,Rs*)-Au(**TY4**)Cl

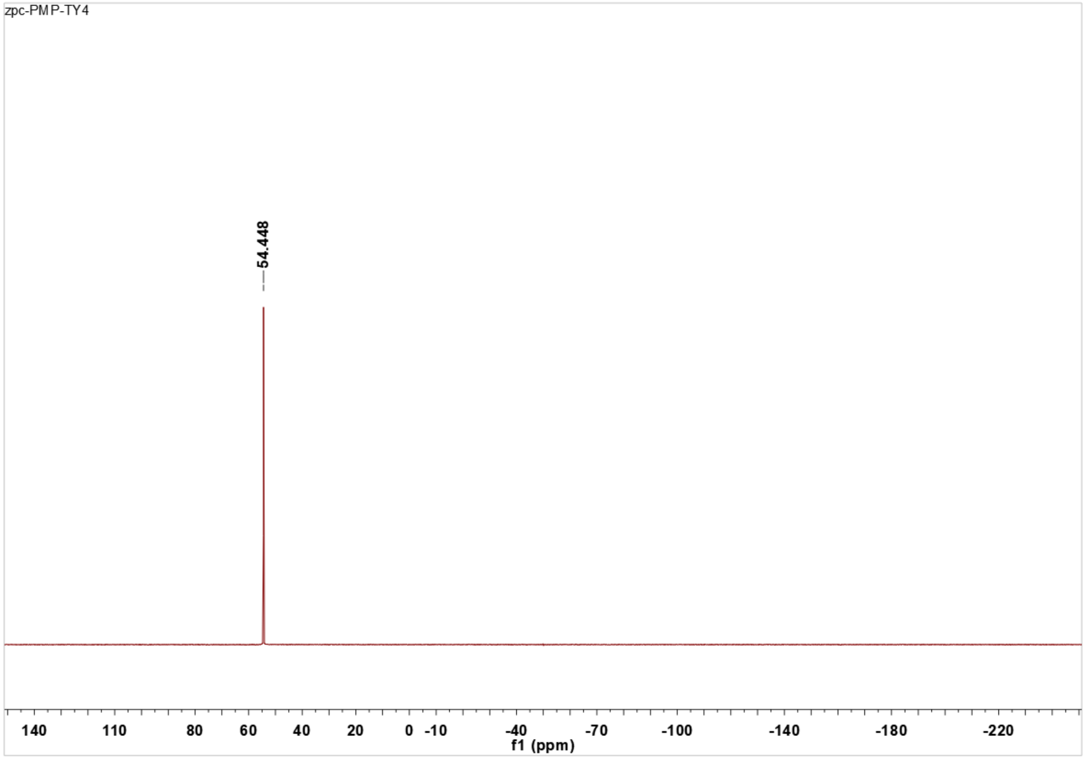

Supplementary Figure 12. ^{31}P NMR (500 MHz, $CDCl_3$) spectra for compound (*Sc,Rs*)-Au(**TY4**)Cl

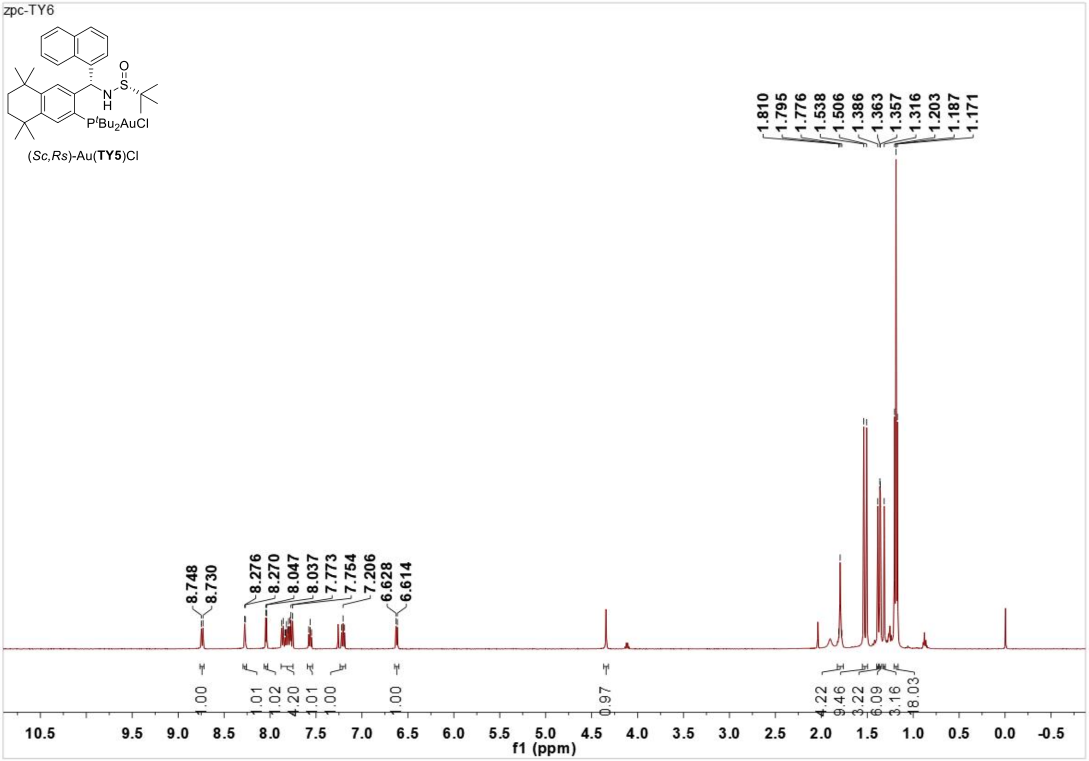

Supplementary Figure 13. ^{1}H NMR (500 MHz, $CDCl_3$) spectra for compound (*Sc,Rs*)-Au(**TY5**)Cl

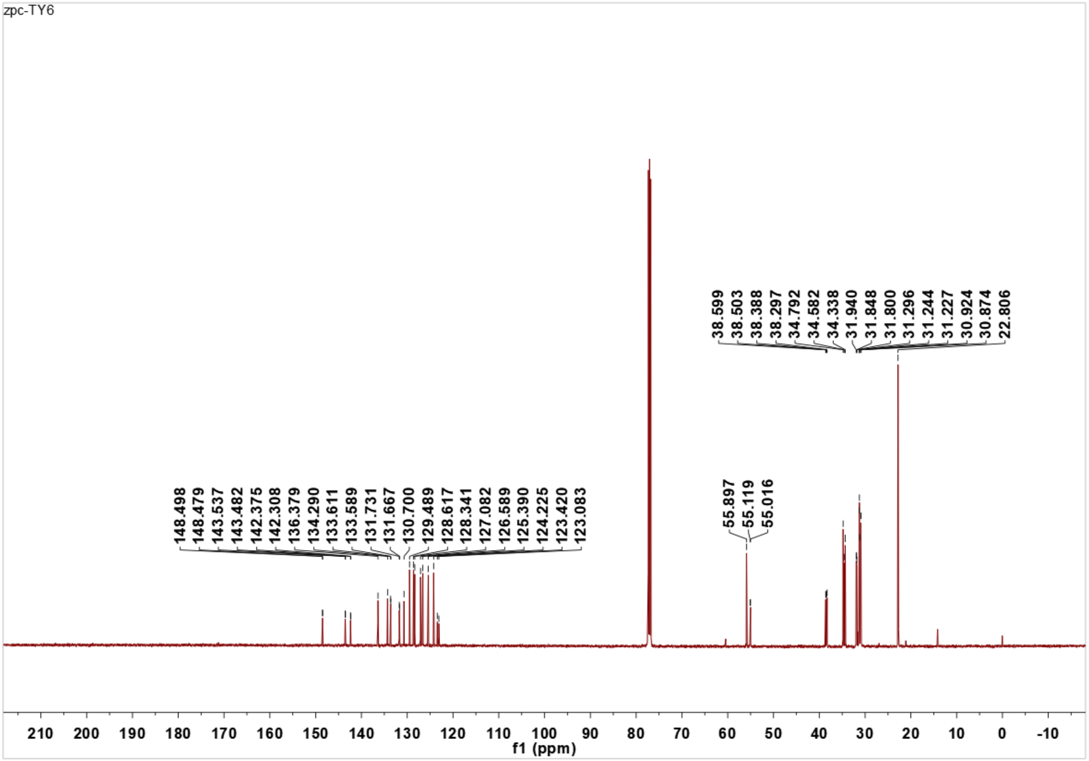

Supplementary Figure 14. ^{13}C NMR (500 MHz, $CDCl_3$) spectra for compound (*Sc,Rs*)-Au(**TY5**)Cl

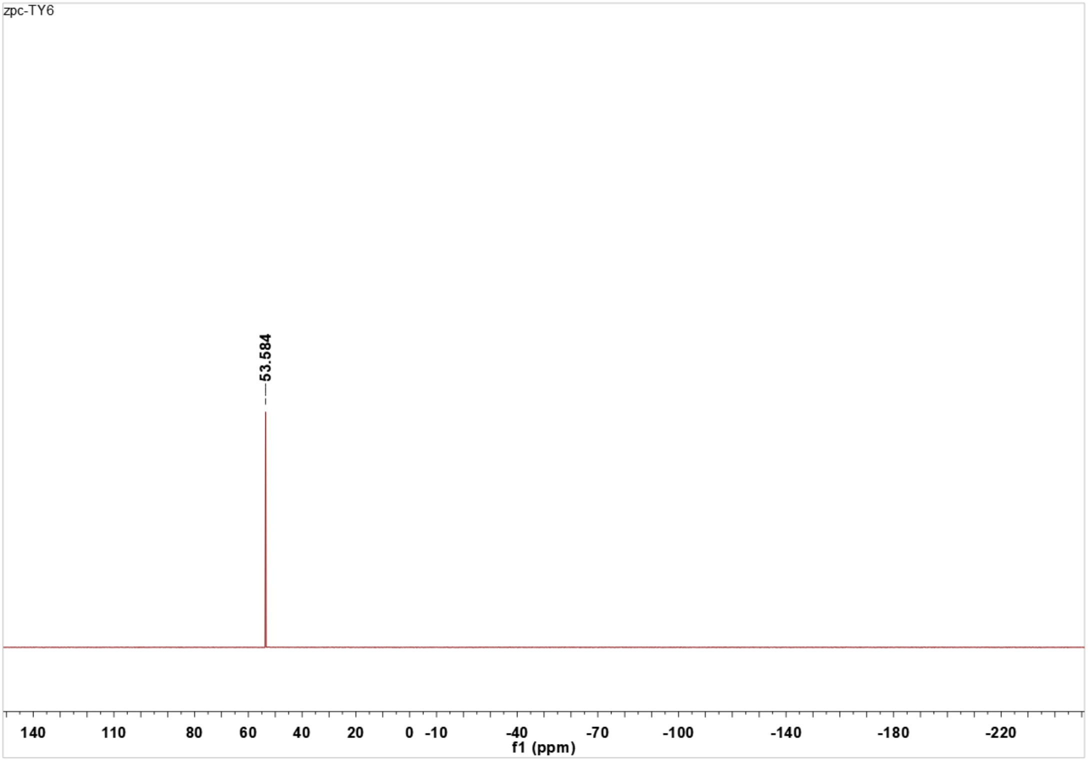

Supplementary Figure 15. ^{31}P NMR (500 MHz, $CDCl_3$) spectra for compound (*Sc,Rs*)-Au(**TY5**)Cl

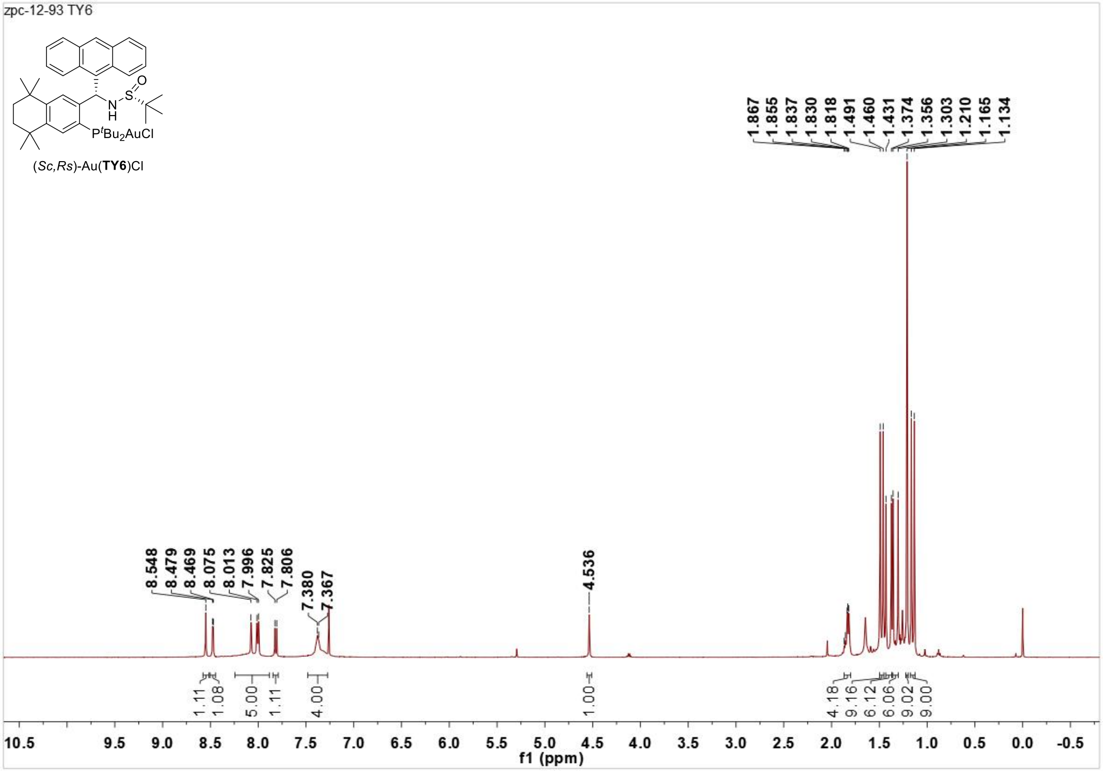

Supplementary Figure 16. ^{1}H NMR (500 MHz, $CDCl_3$) spectra for compound (*Sc,Rs*)-Au(**TY6**)Cl

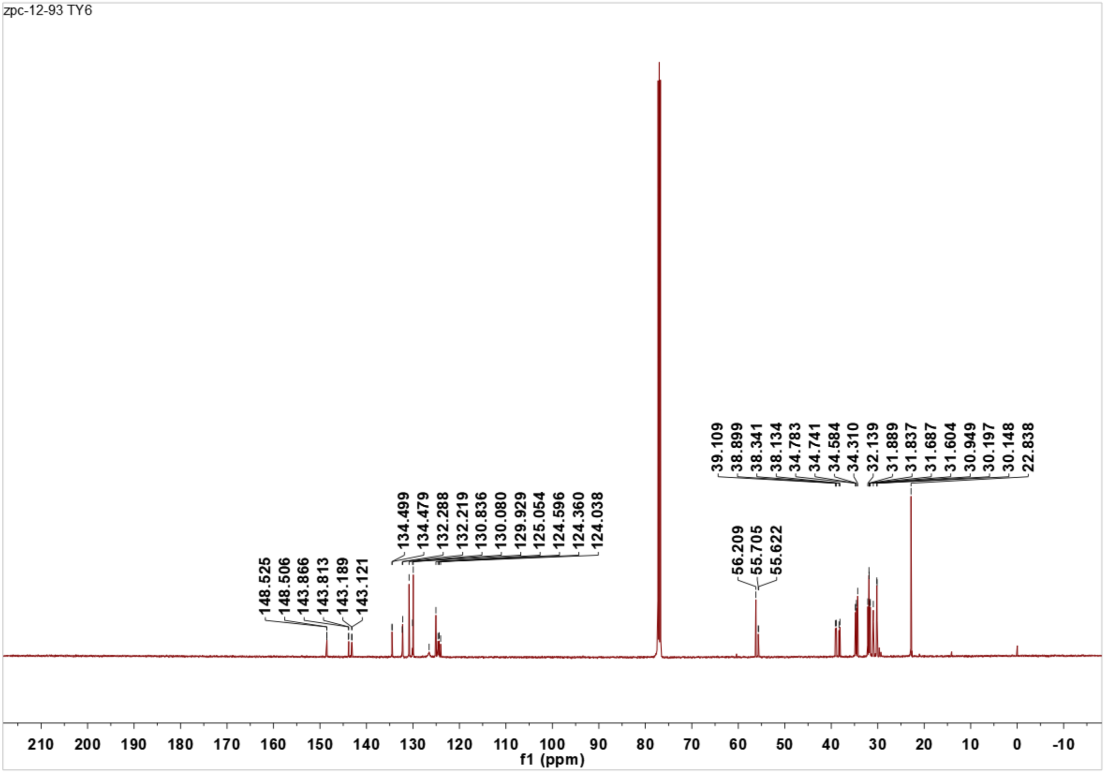

Supplementary Figure 17. ^{13}C NMR (500 MHz, $CDCl_3$) spectra for compound (*Sc,Rs*)-Au(**TY6**)Cl

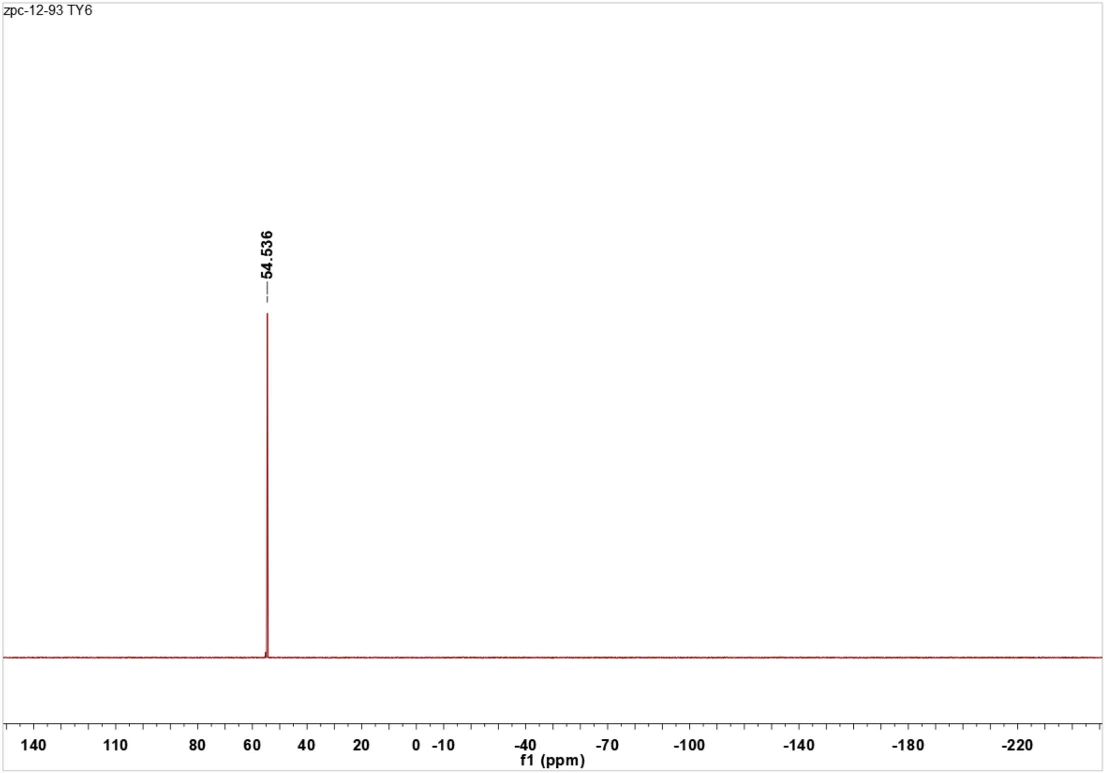

Supplementary Figure 18. ^{31}P NMR (500 MHz, $CDCl_3$) spectra for compound (*Sc,Rs*)-Au(**TY6**)Cl

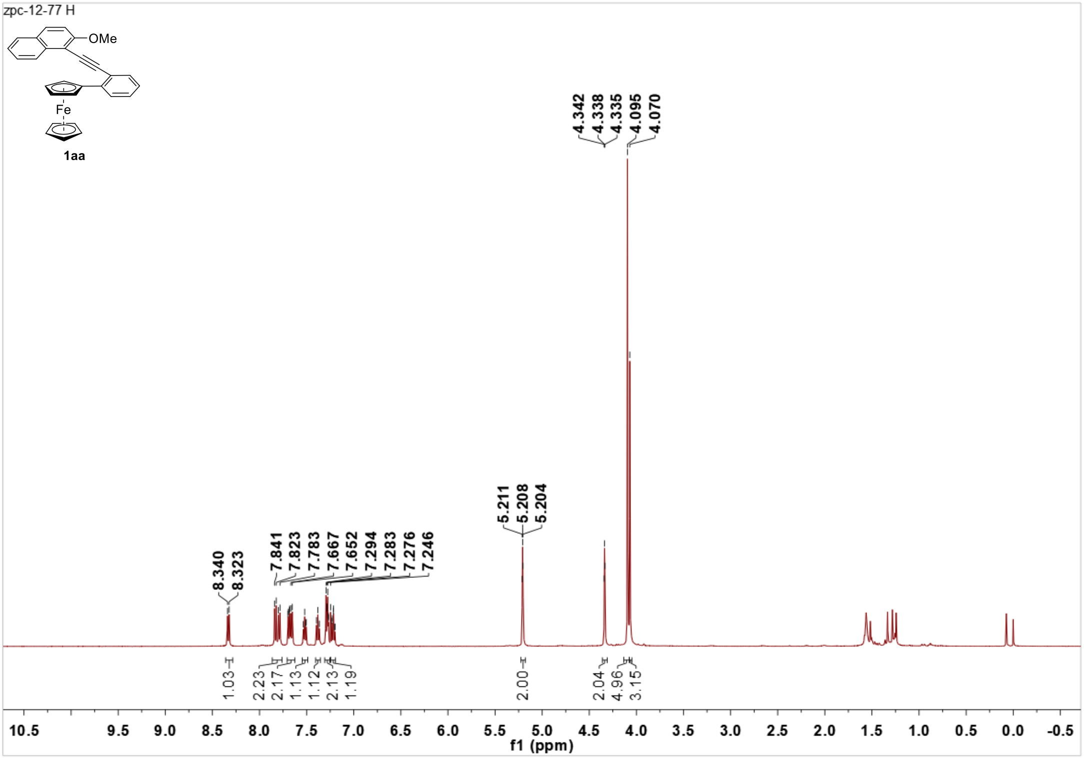

Supplementary Figure 19. ^{1}H NMR (500 MHz, $CDCl_3$) spectra for compound **1aa**

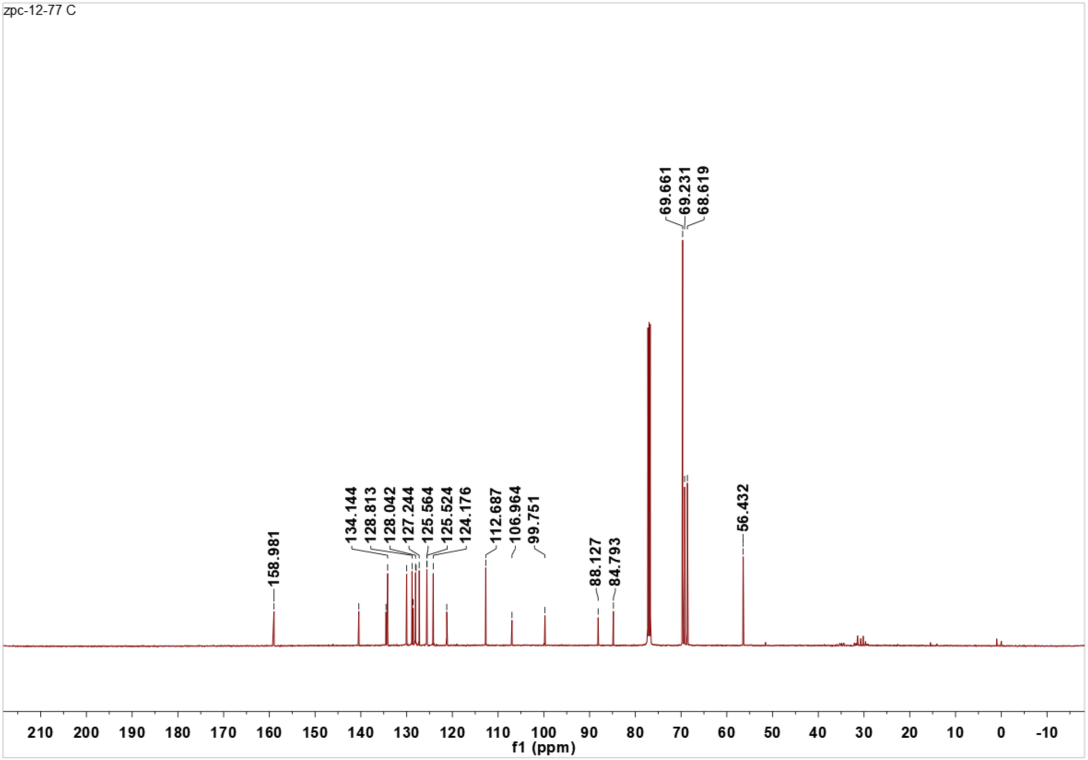

Supplementary Figure 20. ^{13}C NMR (500 MHz, $CDCl_3$) spectra for compound **1aa**

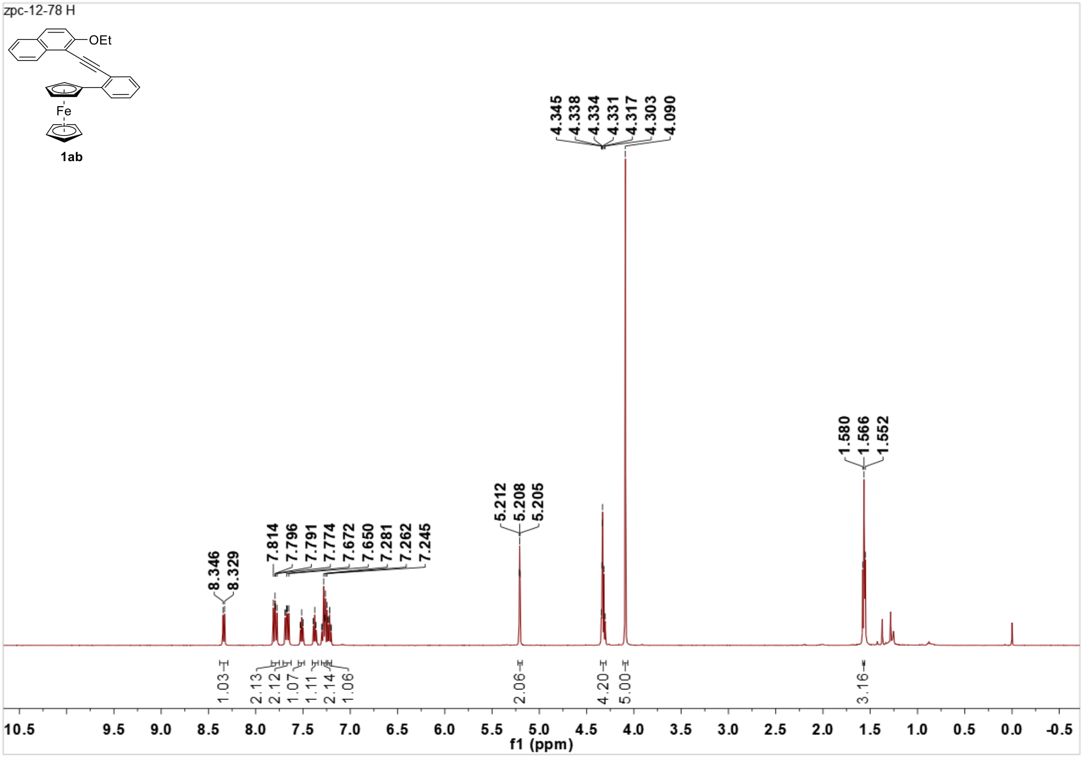

Supplementary Figure 21. ^{1}H NMR (500 MHz, $CDCl_3$) spectra for compound **1ab**

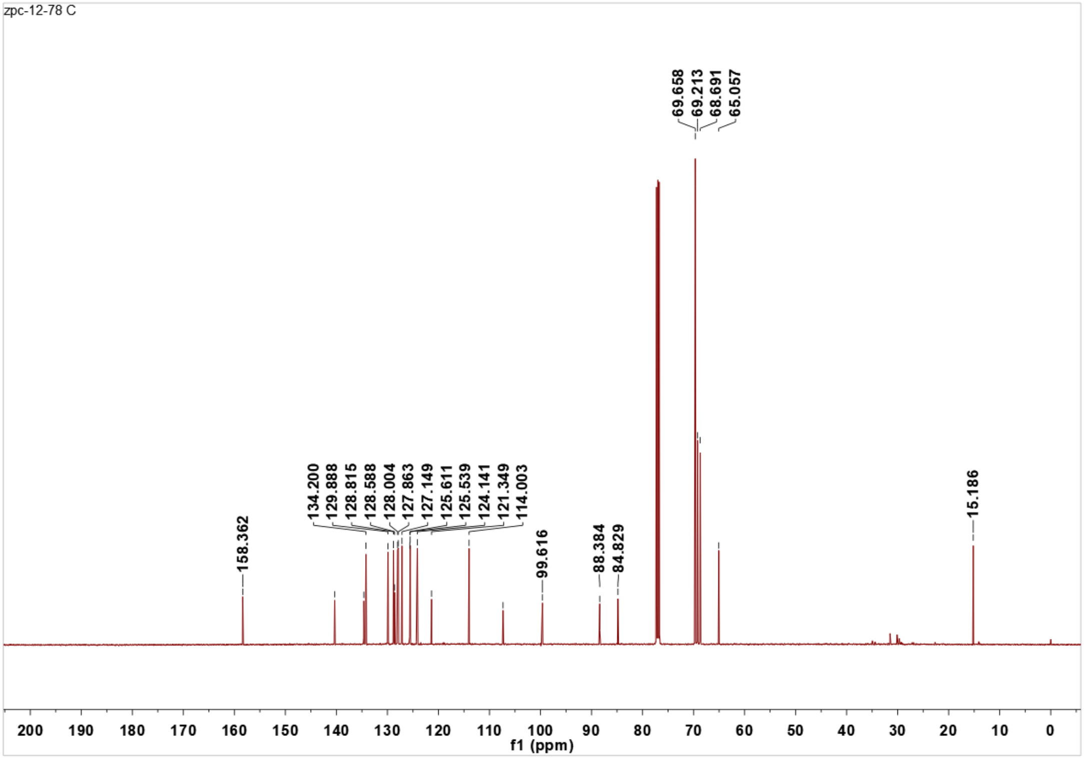

Supplementary Figure 22. ^{13}C NMR (500 MHz, $CDCl_3$) spectra for compound **1ab**

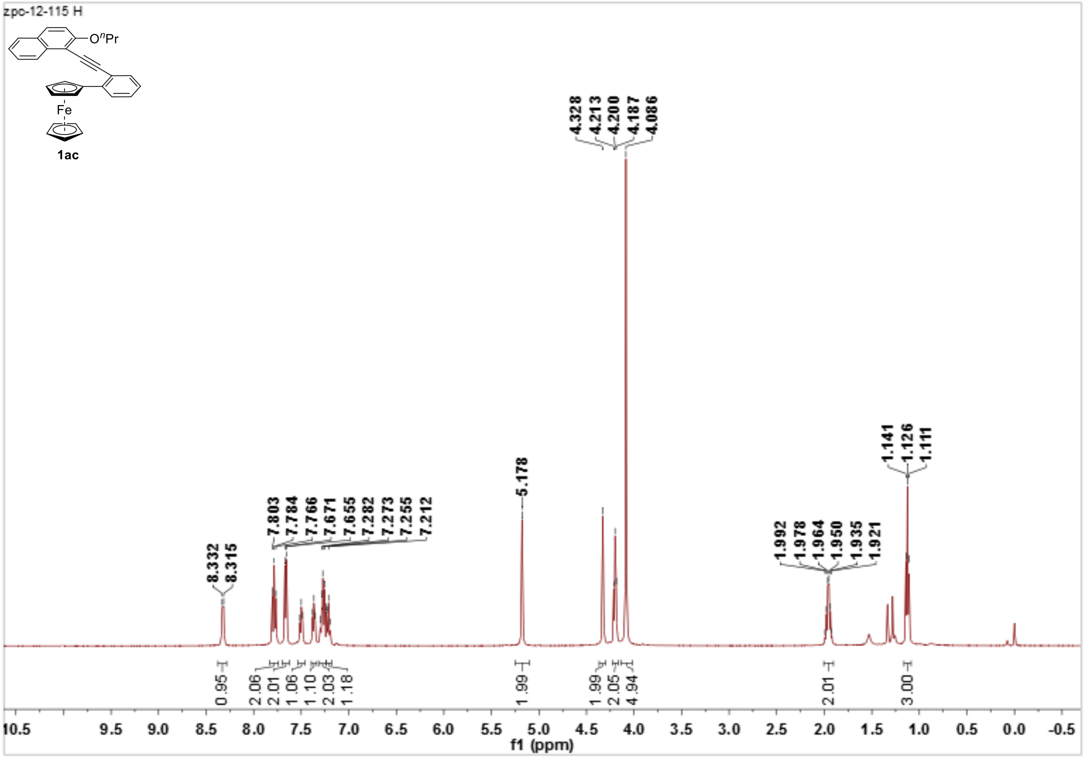

Supplementary Figure 23. ^{1}H NMR (500 MHz, $CDCl_3$) spectra for compound **1ac**

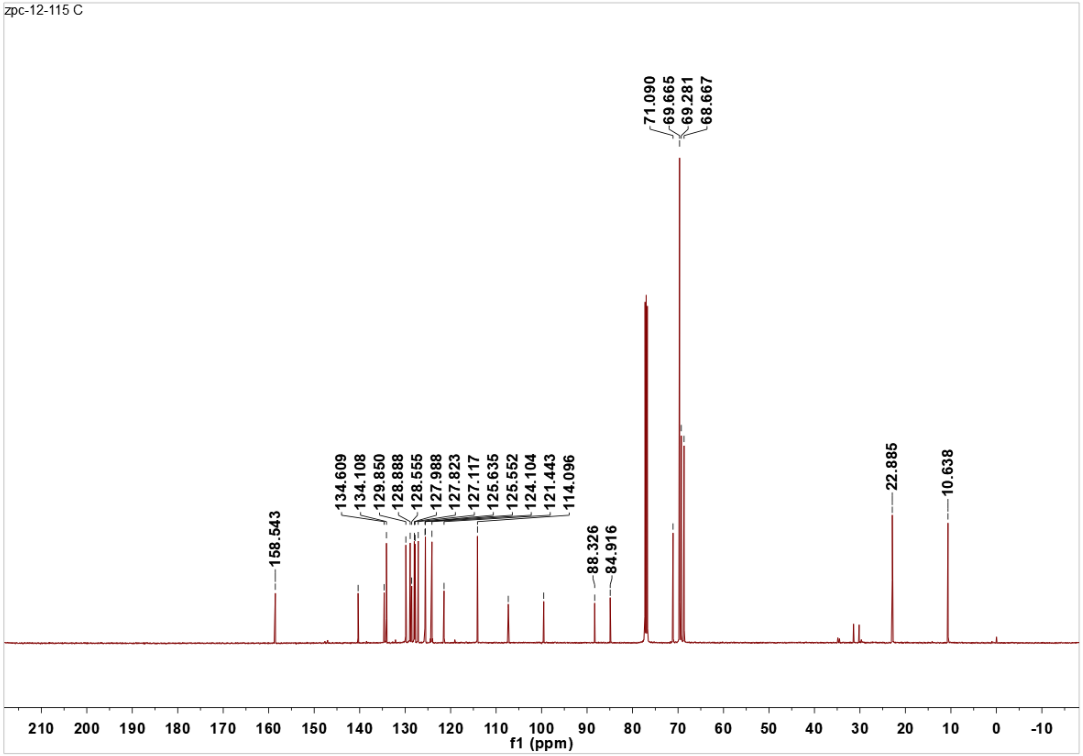

Supplementary Figure 24. ^{13}C NMR (500 MHz, $CDCl_3$) spectra for compound **1ac**

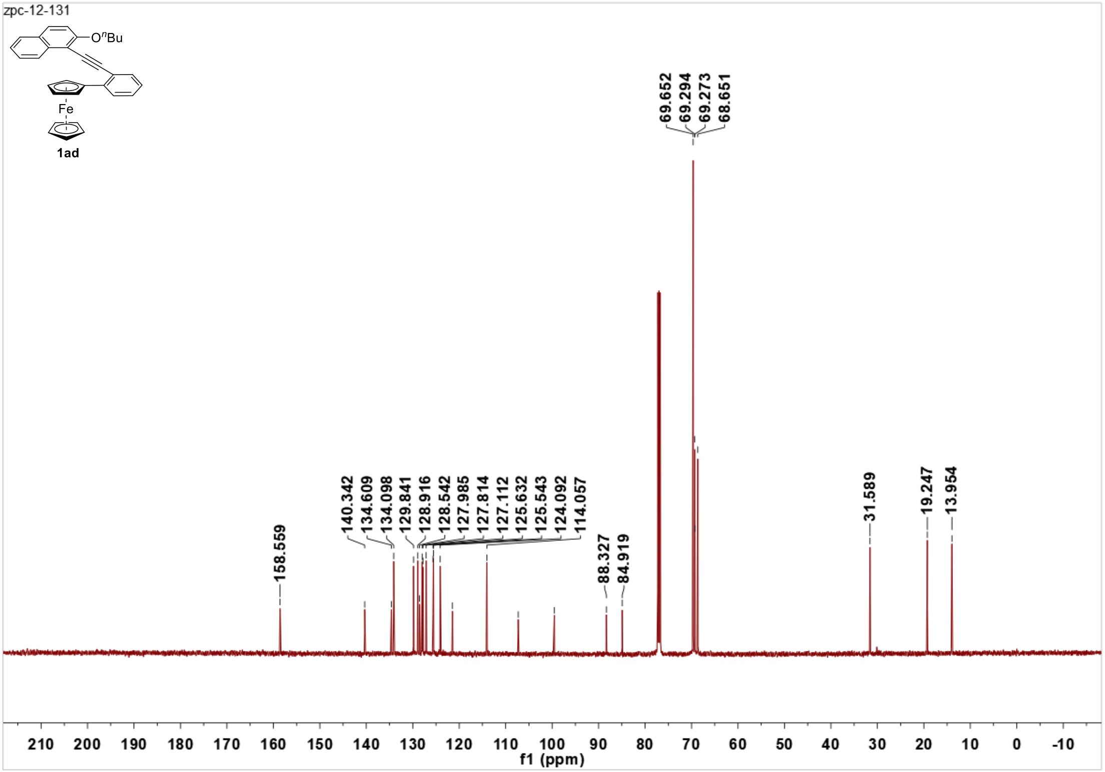

Supplementary Figure 25. ^{1}H NMR (500 MHz, $CDCl_3$) spectra for compound **1ad**

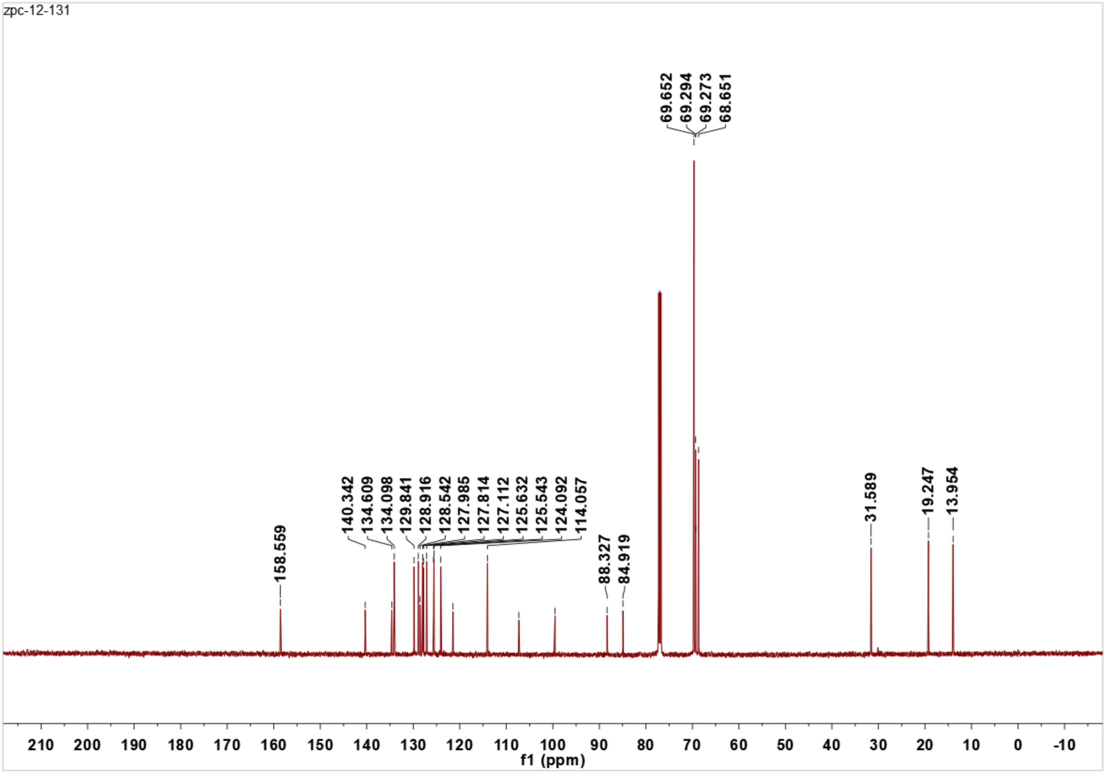

Supplementary Figure 26. ^{13}C NMR (500 MHz, $CDCl_3$) spectra for compound **1ad**

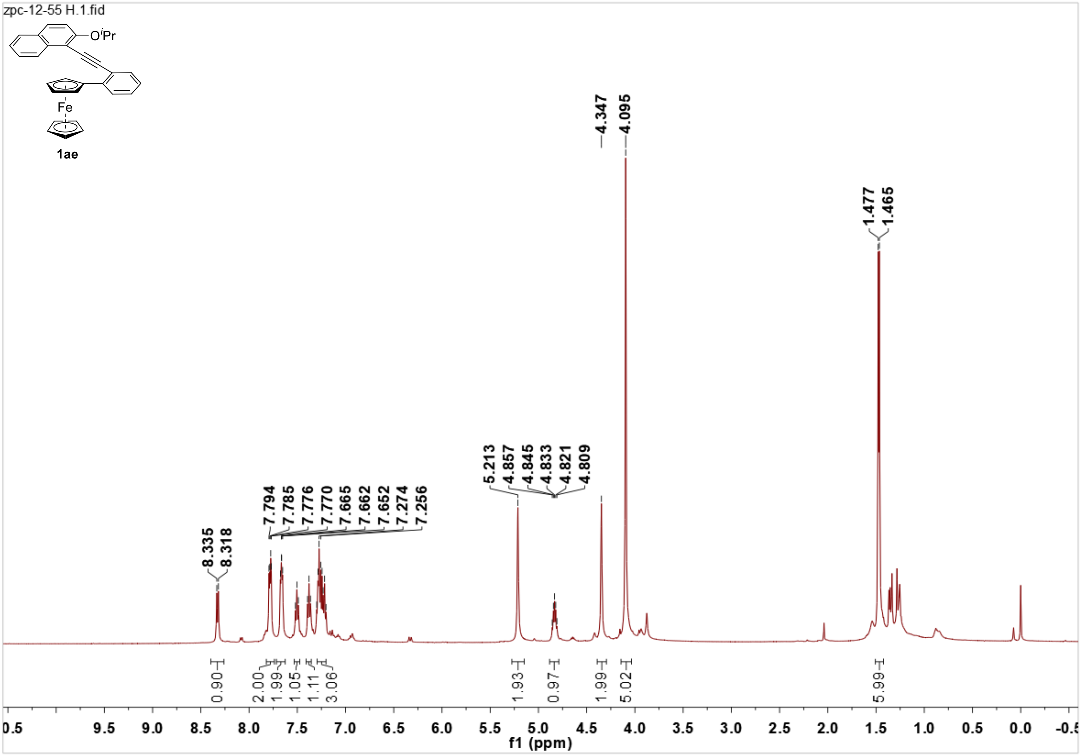

Supplementary Figure 27. ^{1}H NMR (500 MHz, $CDCl_3$) spectra for compound **1ae**

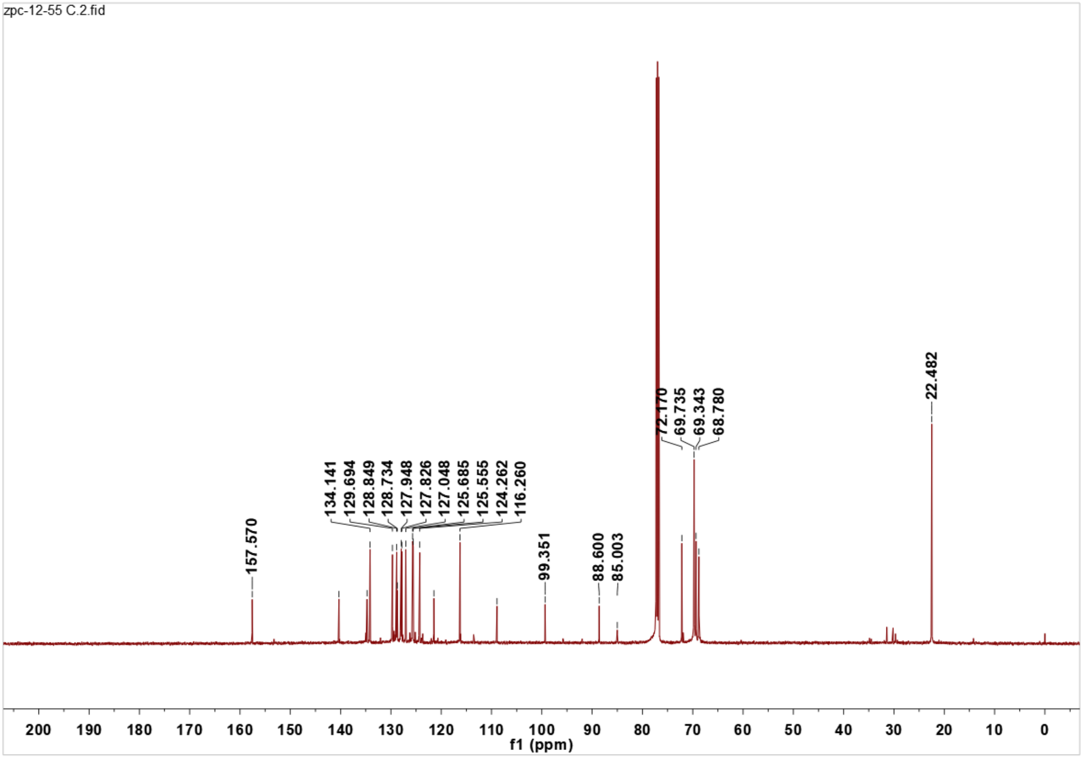

Supplementary Figure 28. ^{13}C NMR (500 MHz, $CDCl_3$) spectra for compound **1ae**

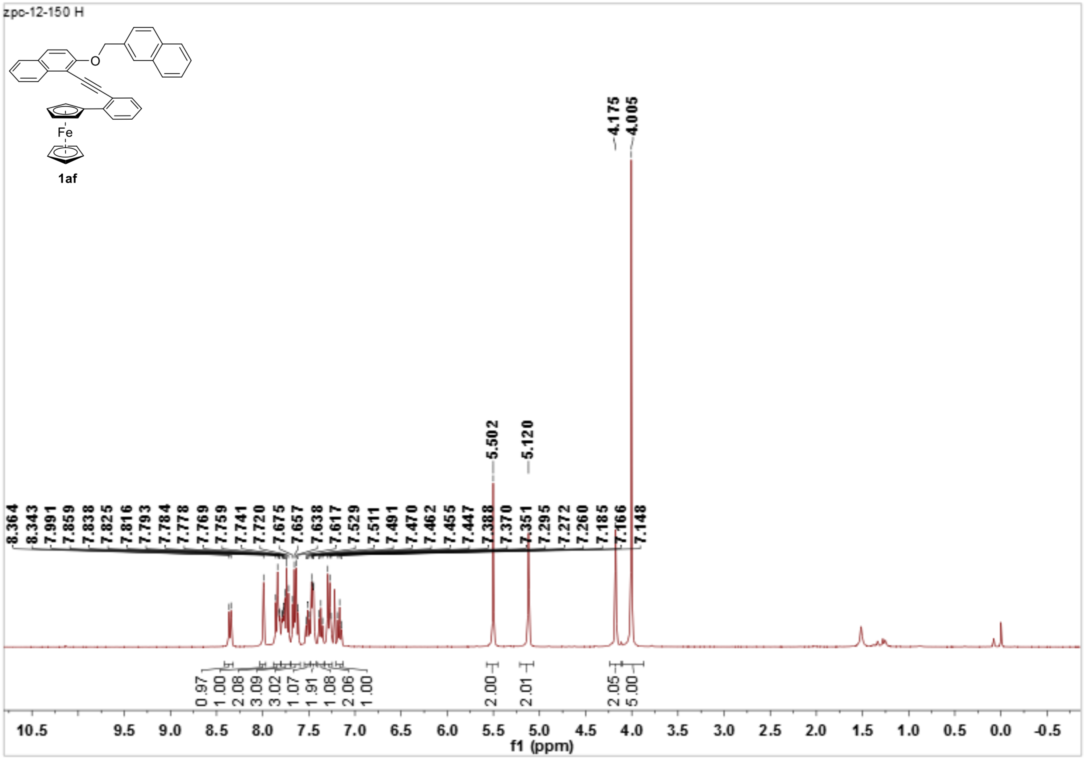

Supplementary Figure 29. ^{1}H NMR (400 MHz, $CDCl_3$) spectra for compound **1af**

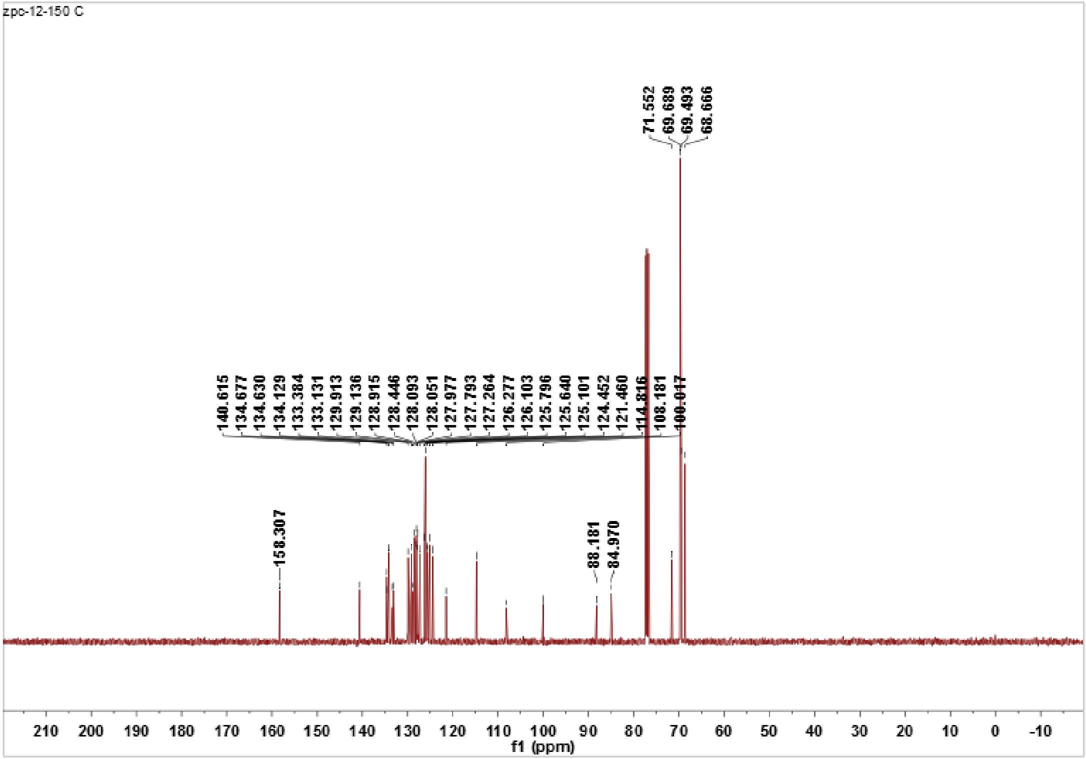

Supplementary Figure 30. ^{13}C NMR (400 MHz, $CDCl_3$) spectra for compound **1af**

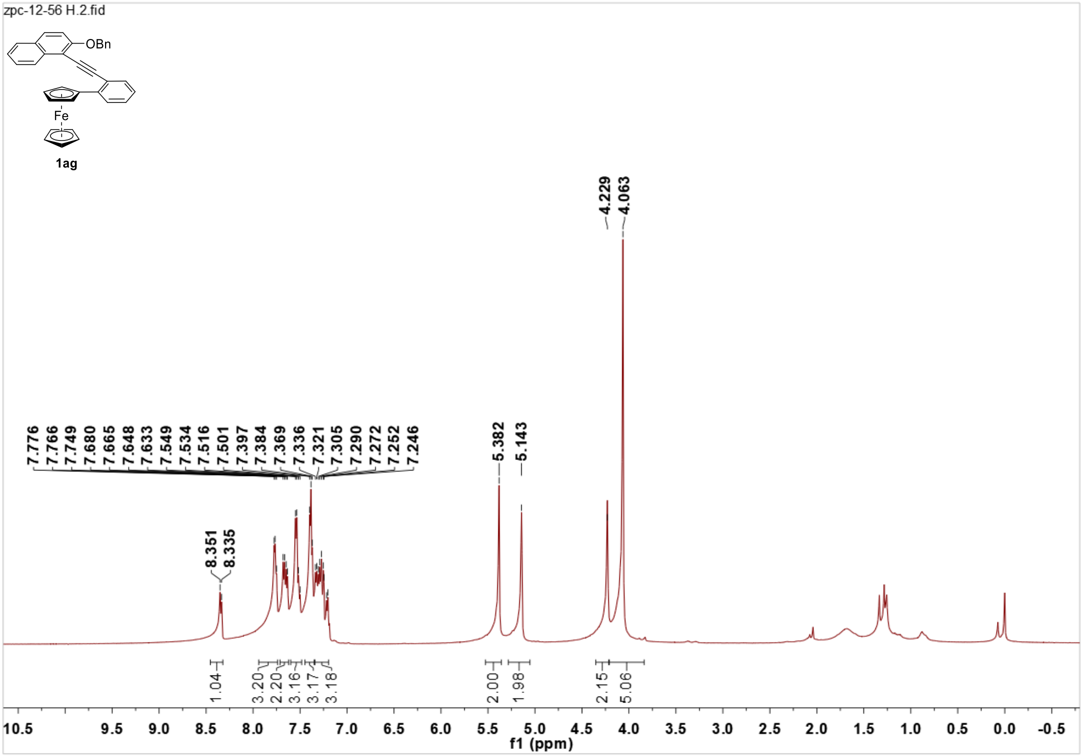

Supplementary Figure 31. ^{1}H NMR (500 MHz, $CDCl_3$) spectra for compound **1ag**

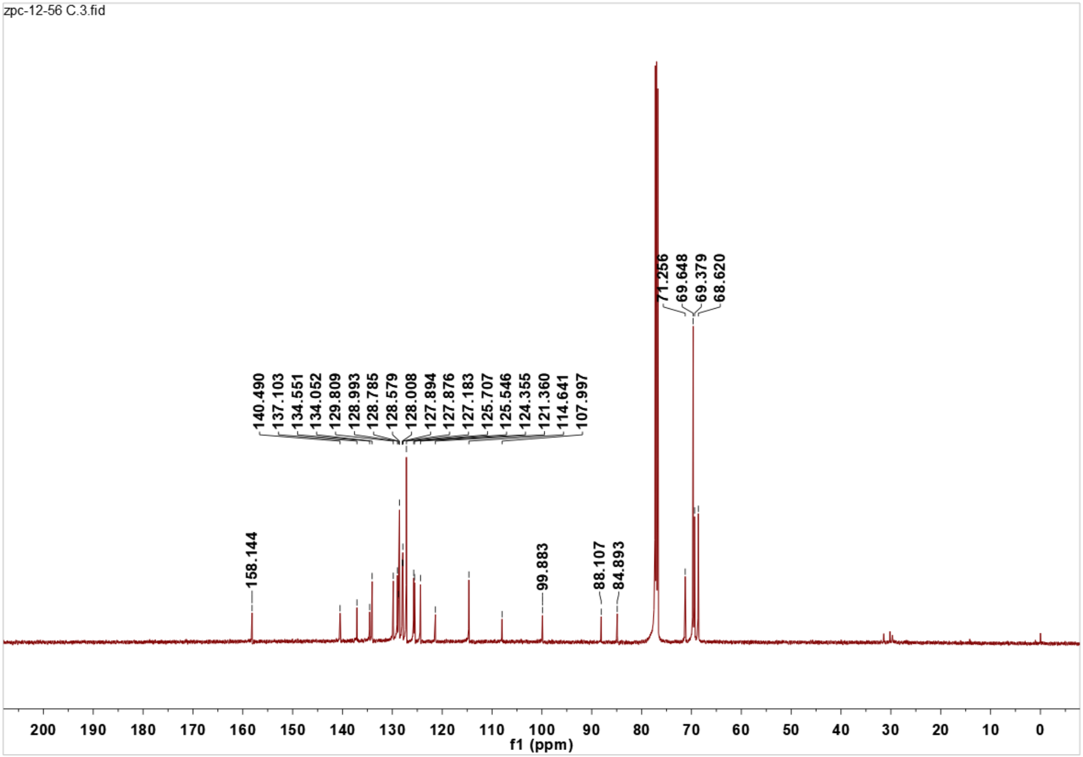

Supplementary Figure 32. ^{13}C NMR (500 MHz, $CDCl_3$) spectra for compound **1ag**

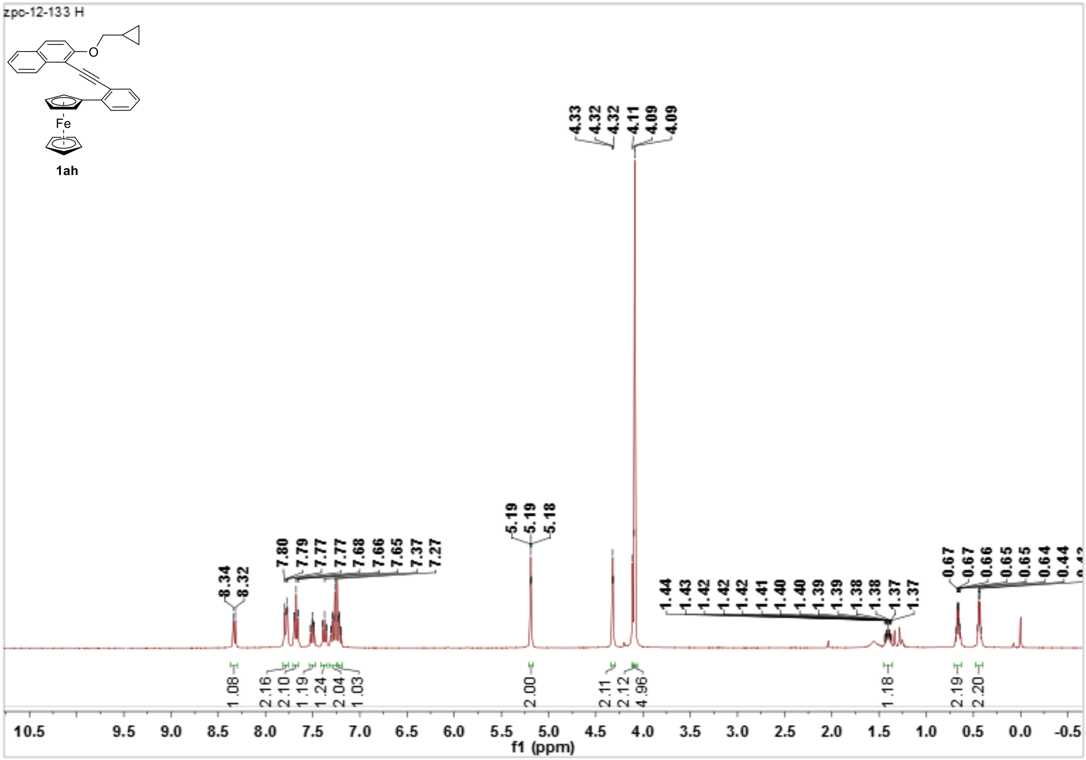

Supplementary Figure 33. ^{1}H NMR (400 MHz, $CDCl_3$) spectra for compound **1ah**

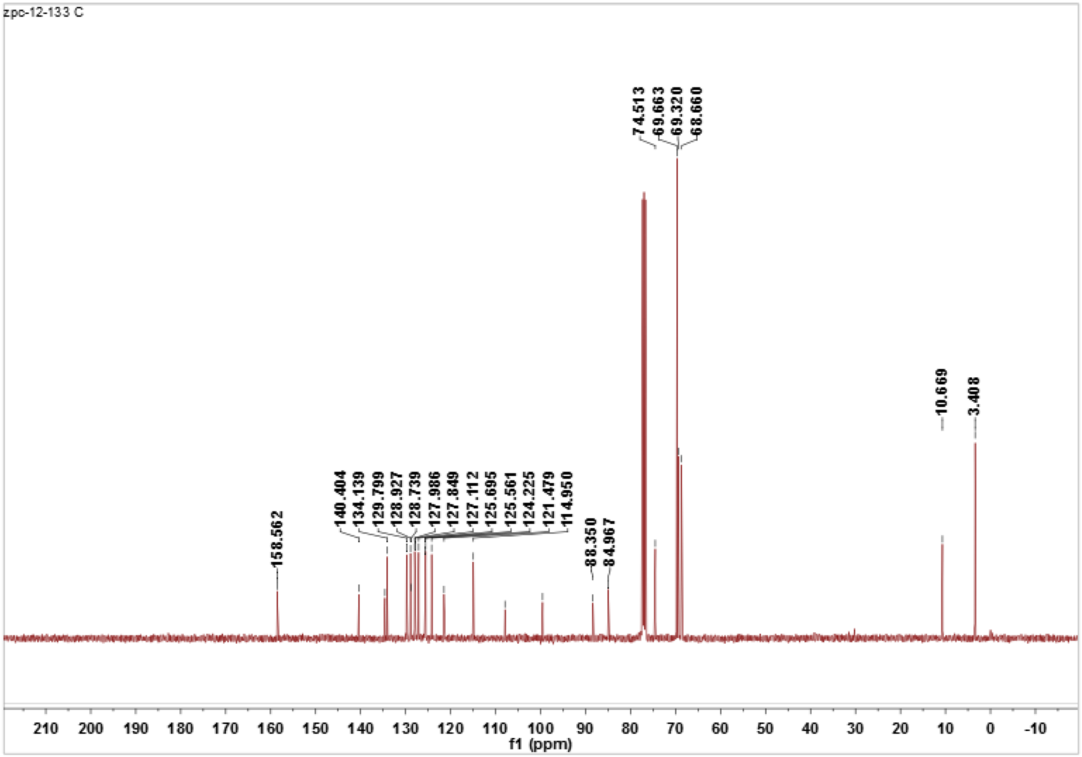

Supplementary Figure 34. ^{13}C NMR (400 MHz, $CDCl_3$) spectra for compound **1ah**

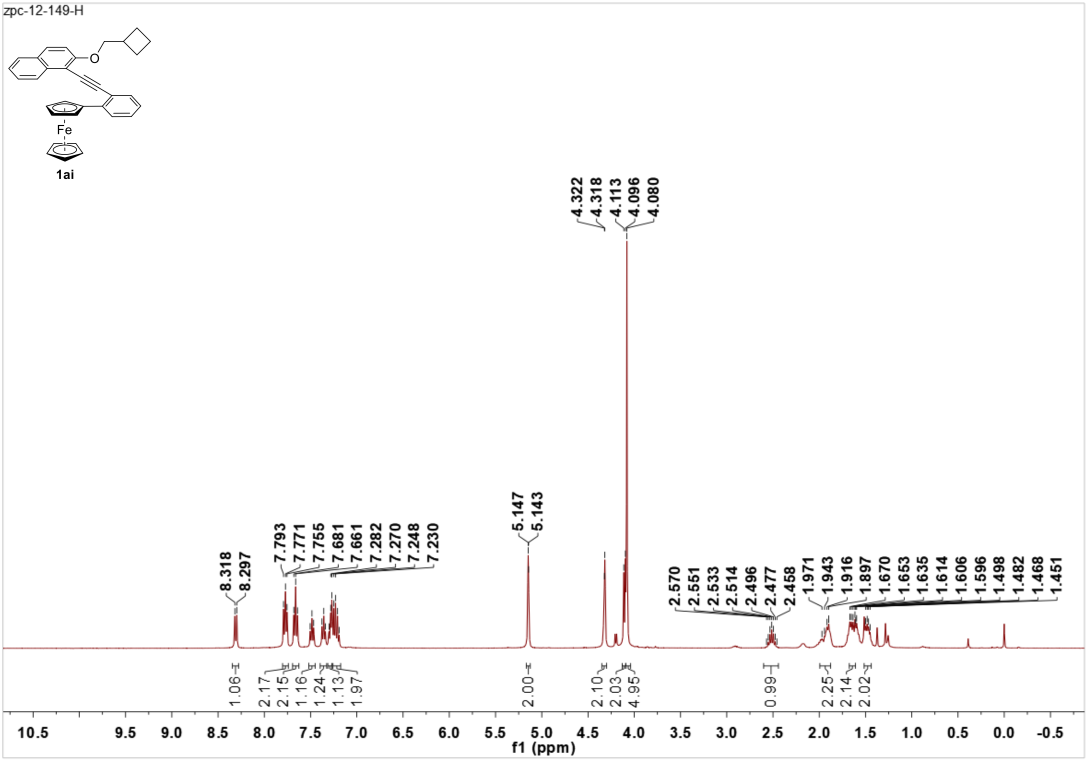

Supplementary Figure 35. ^{1}H NMR (400 MHz, $CDCl_3$) spectra for compound **1ai**

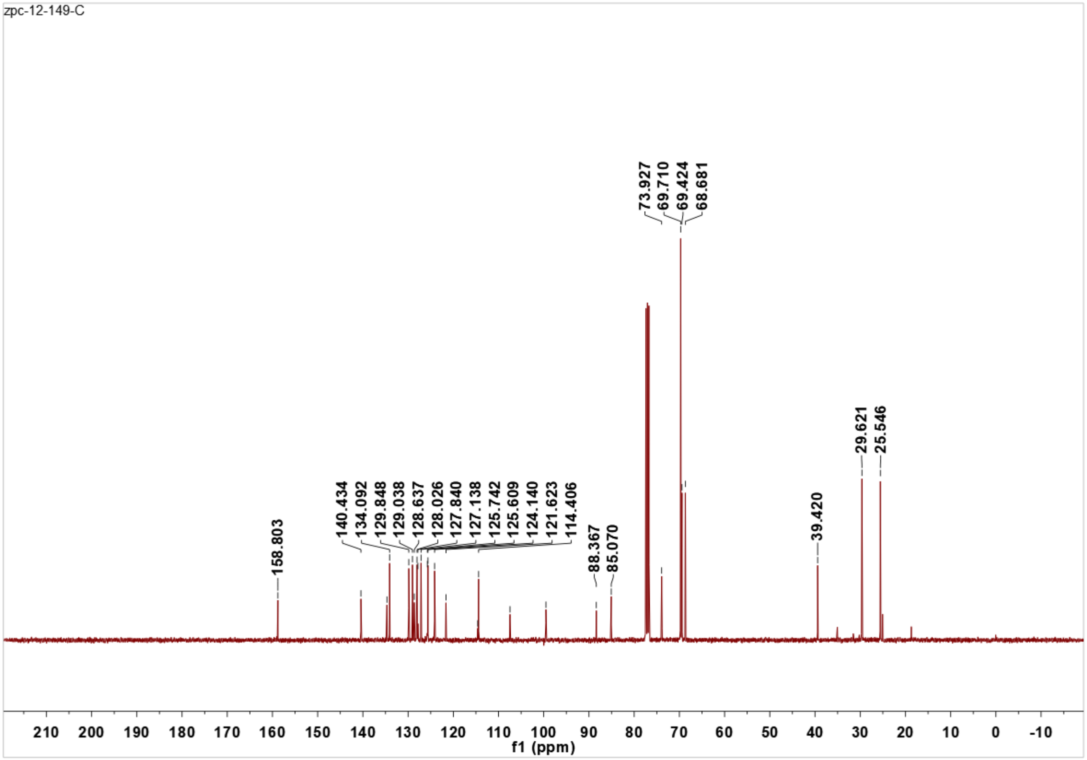

Supplementary Figure 36. ^{13}C NMR (400 MHz, $CDCl_3$) spectra for compound **1ai**

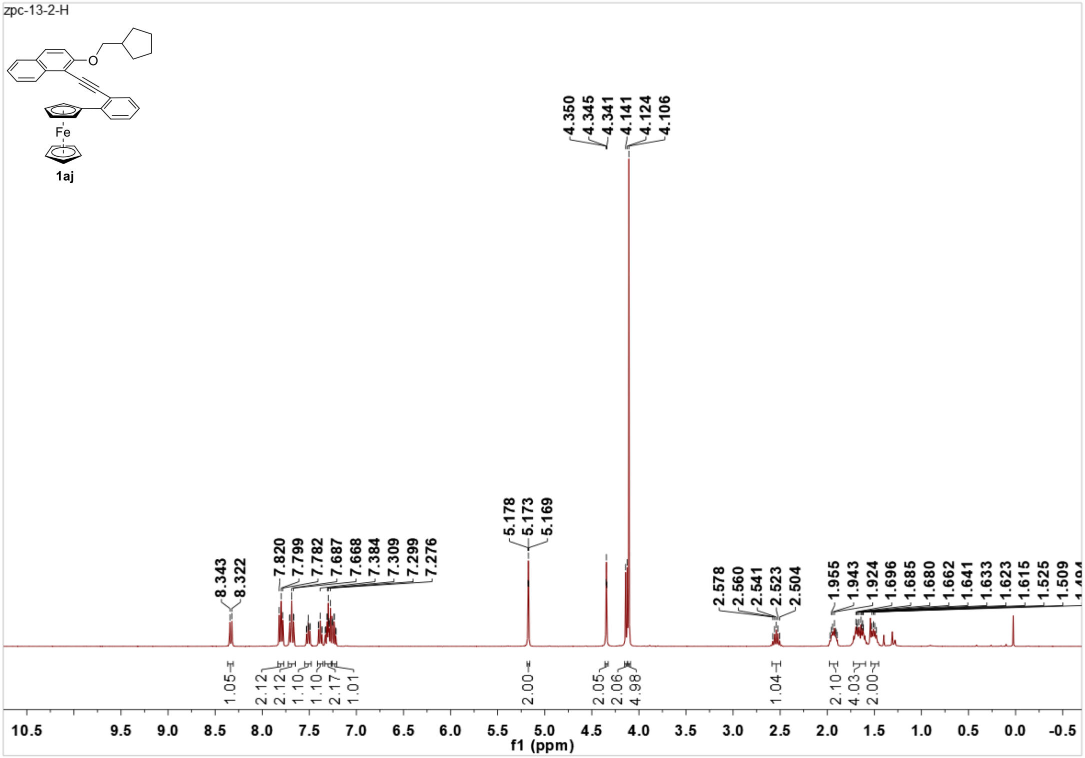

Supplementary Figure 37. ^{1}H NMR (400 MHz, $CDCl_3$) spectra for compound **1aj**

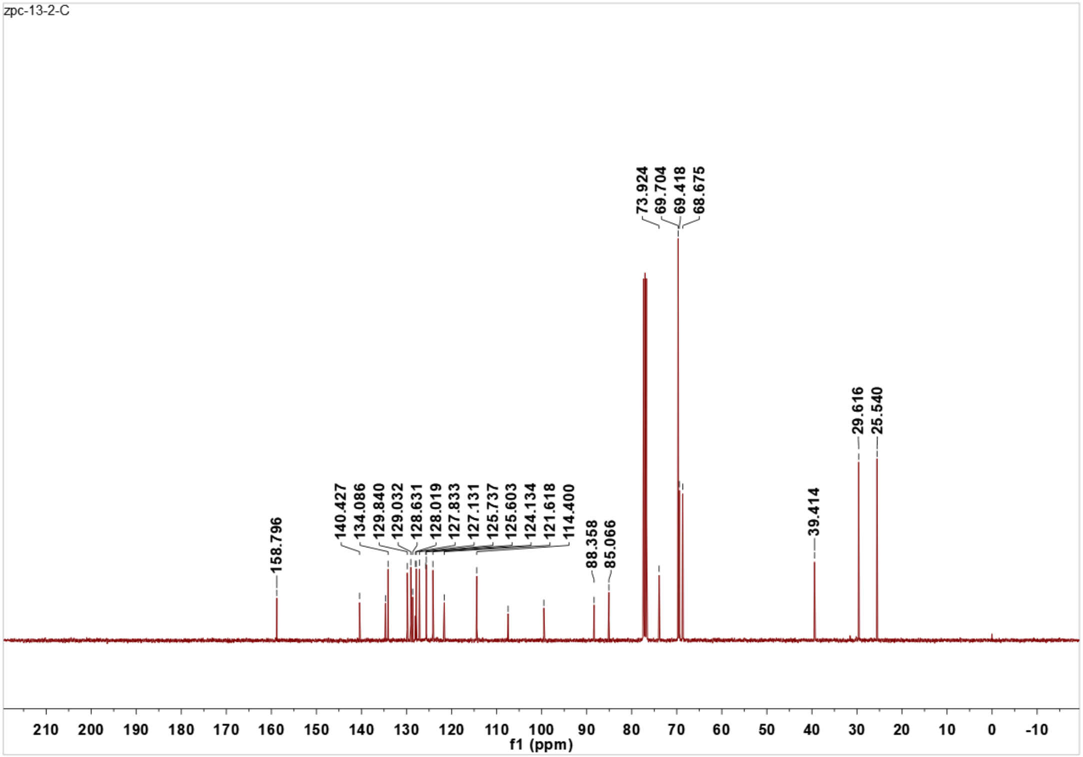

Supplementary Figure 38. ^{13}C NMR (400 MHz, $CDCl_3$) spectra for compound **1aj**

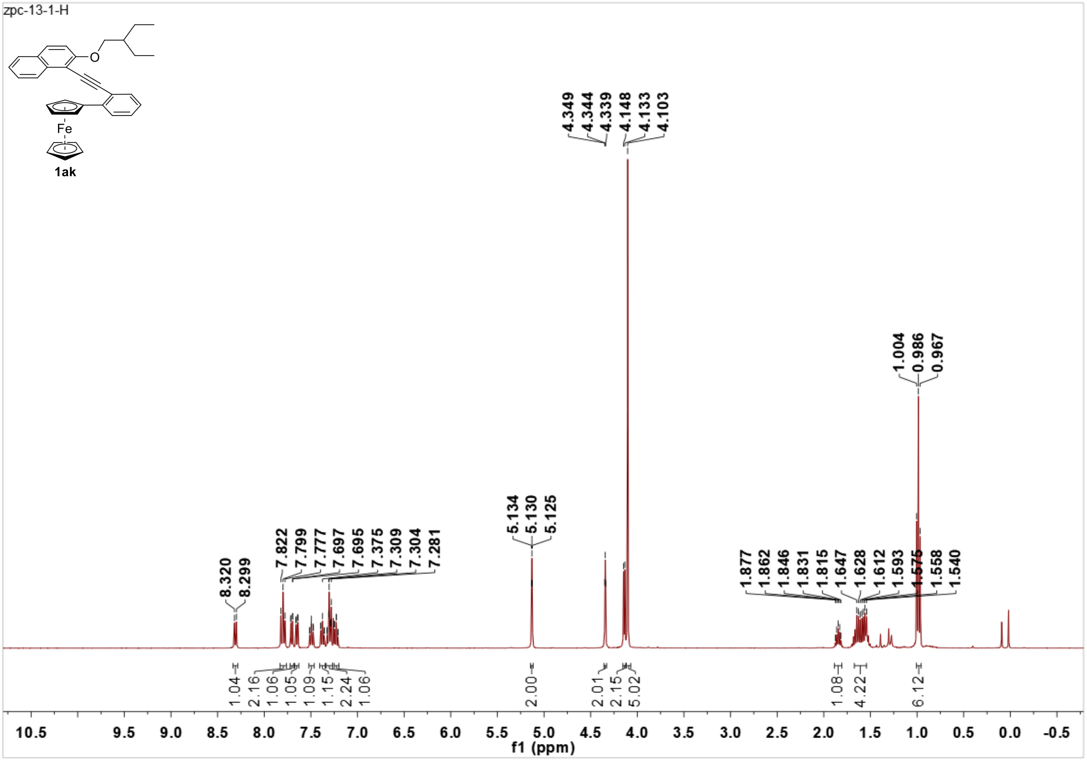

Supplementary Figure 39. ^{1}H NMR (400 MHz, $CDCl_3$) spectra for compound **1ak**

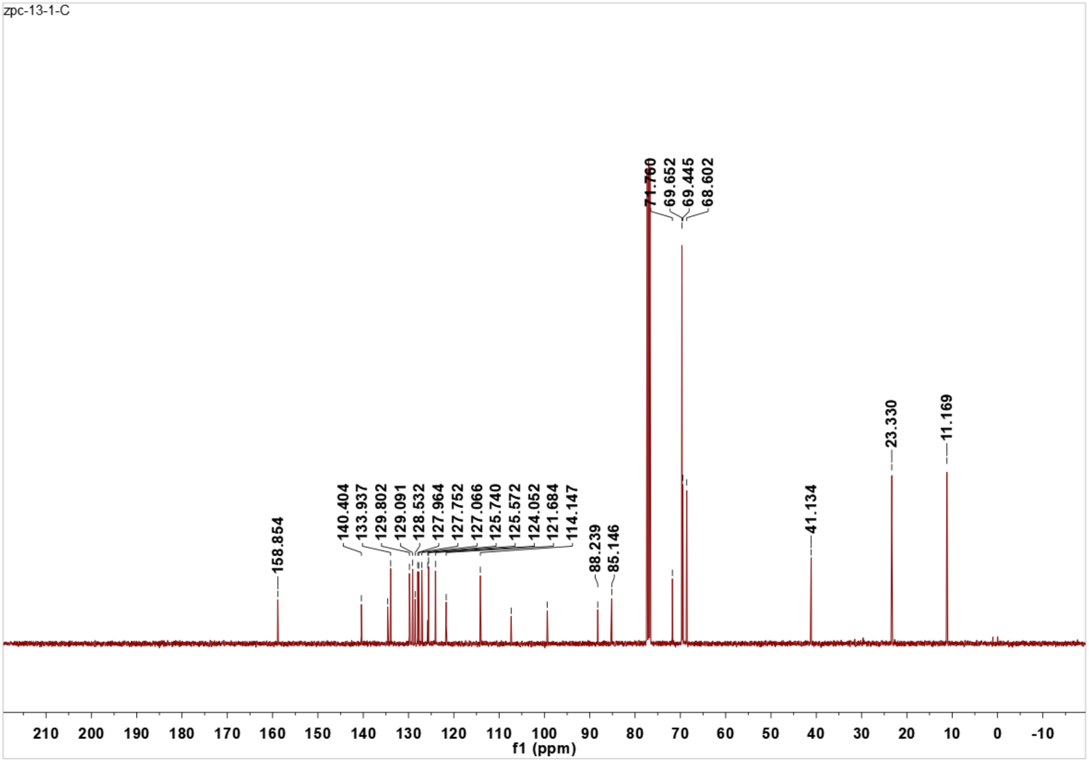

Supplementary Figure 40. ^{13}C NMR (400 MHz, $CDCl_3$) spectra for compound **1ak**

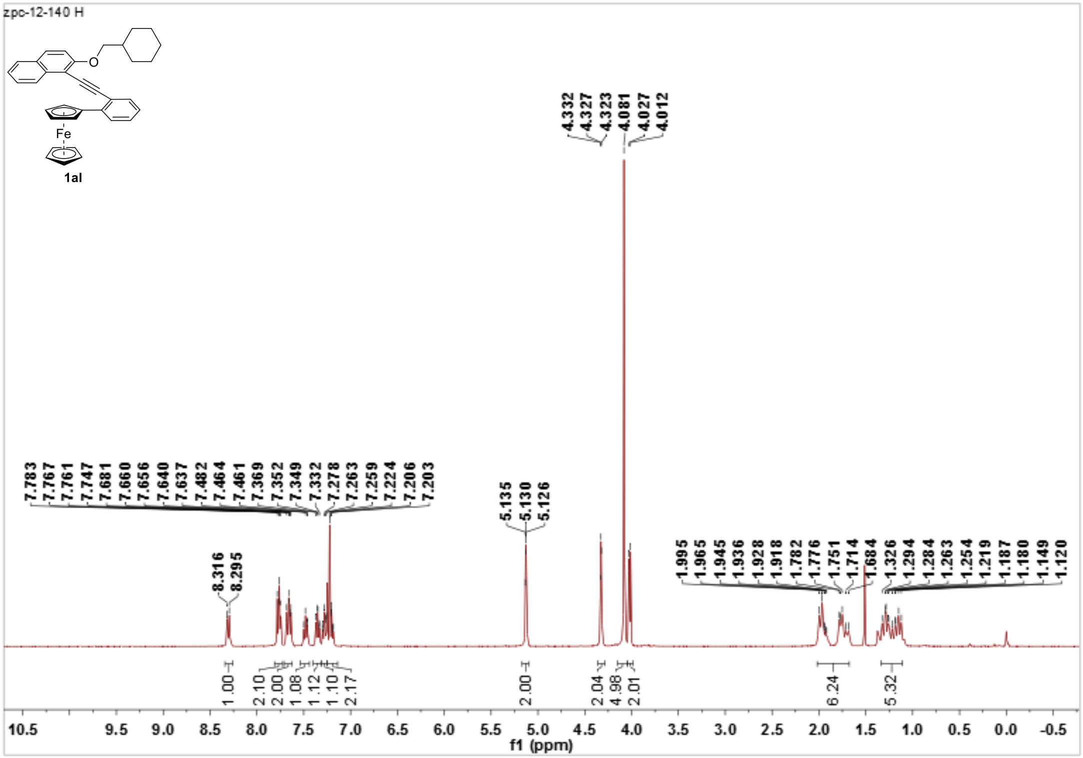

Supplementary Figure 41. ^{1}H NMR (400 MHz, $CDCl_3$) spectra for compound **1al**

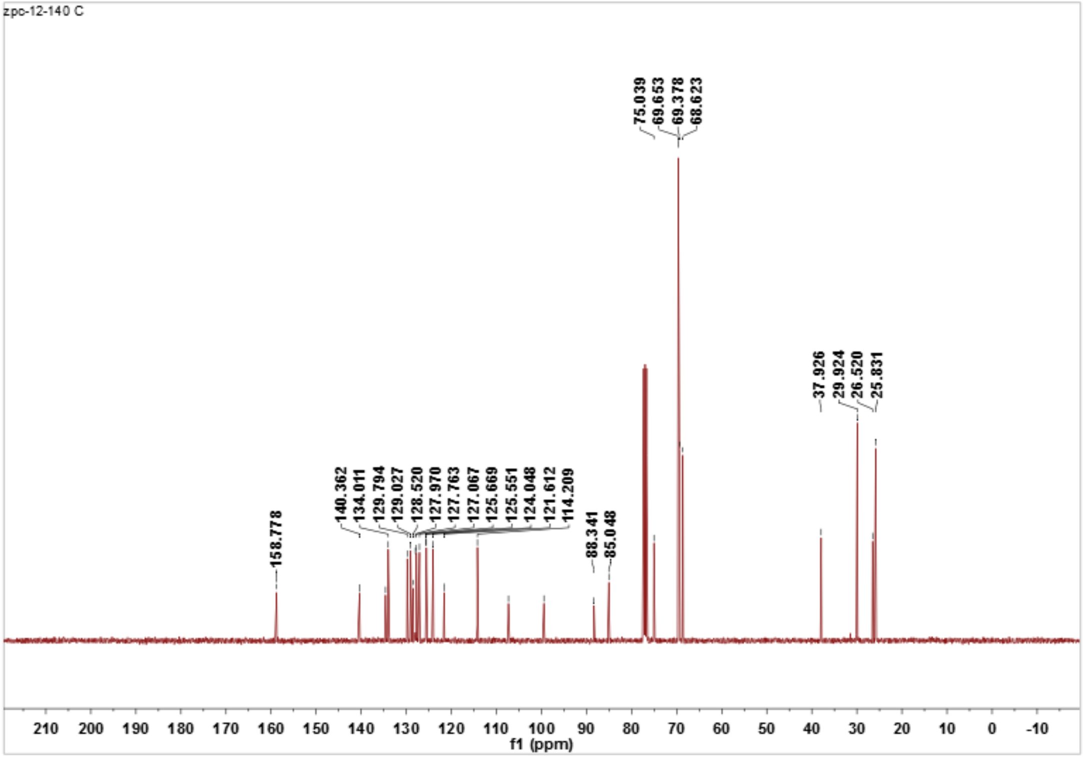

Supplementary Figure 42. ^{13}C NMR (400 MHz, $CDCl_3$) spectra for compound **1al**

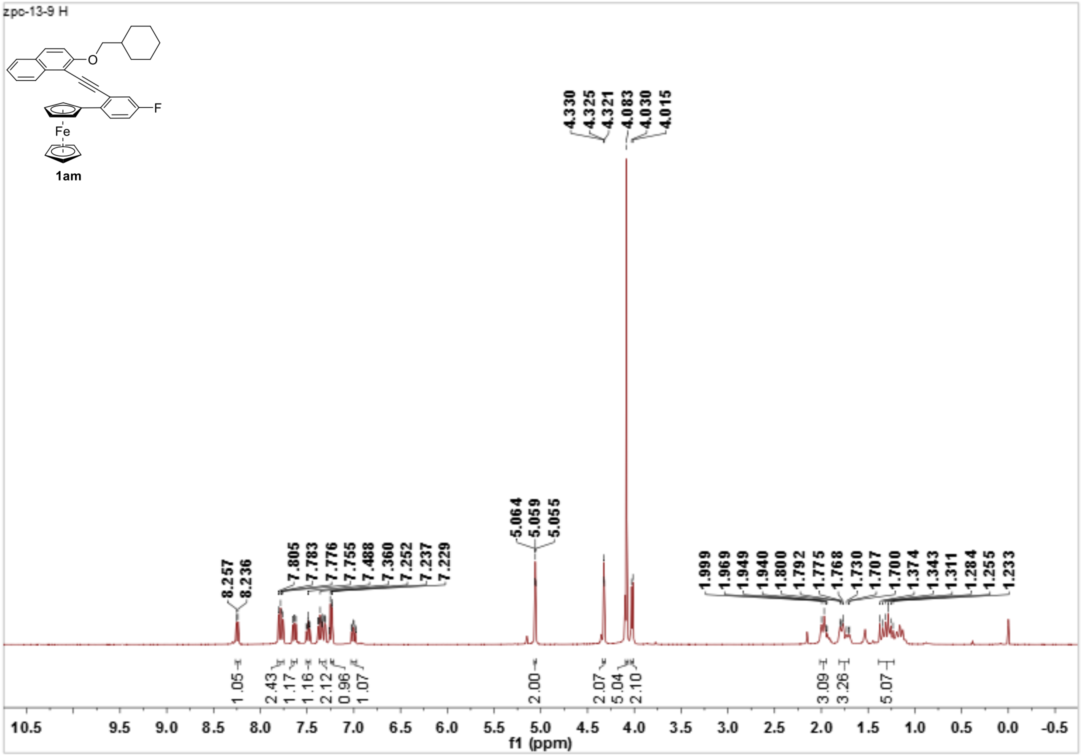

Supplementary Figure 43. ^{1}H NMR (400 MHz, $CDCl_3$) spectra for compound **1am**

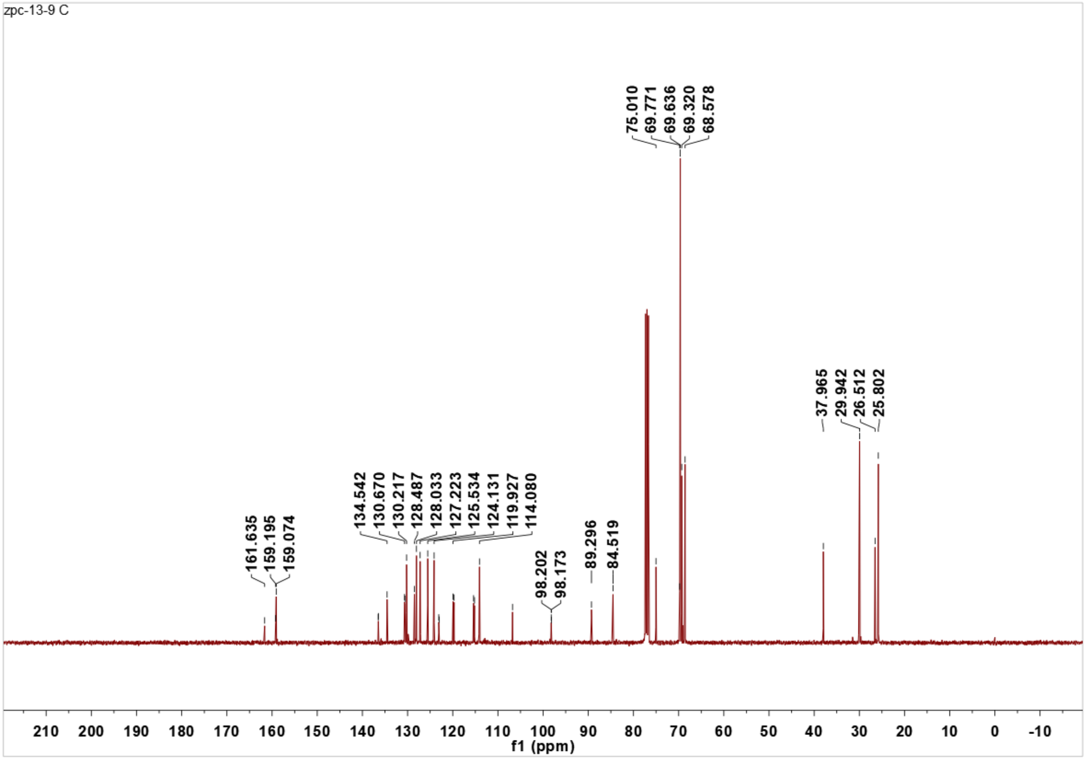

Supplementary Figure 44. ^{13}C NMR (400 MHz, $CDCl_3$) spectra for compound **1am**

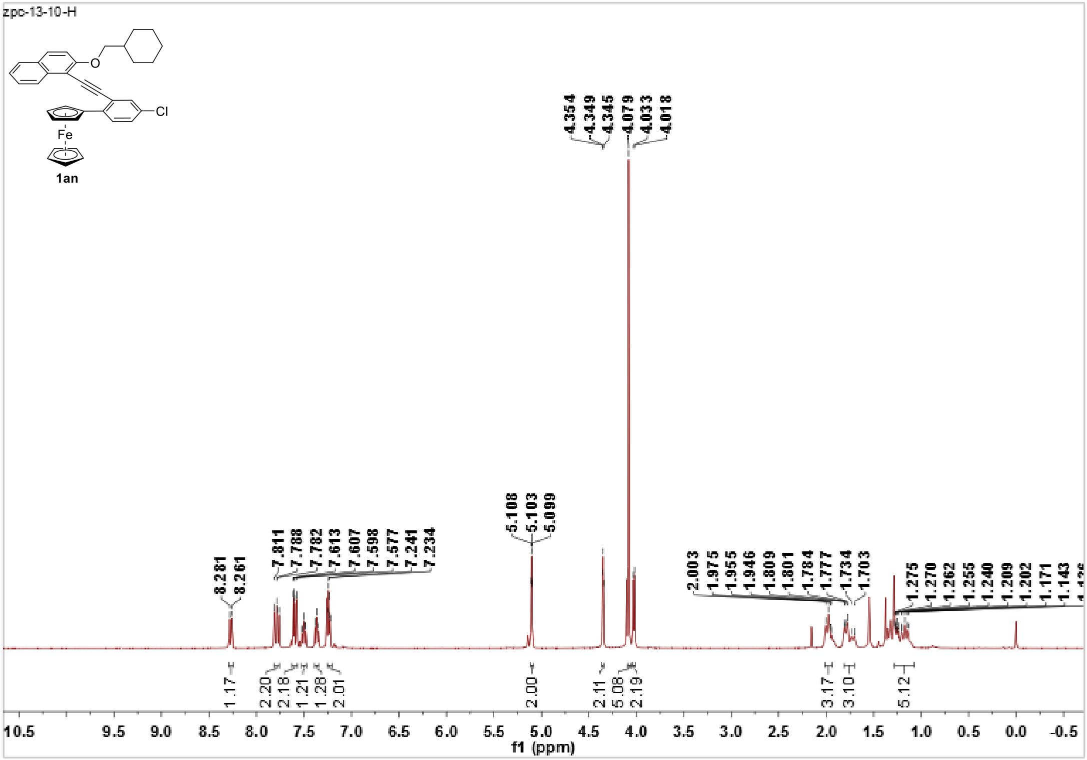

Supplementary Figure 45. ^{1}H NMR (400 MHz, $CDCl_3$) spectra for compound **1an**

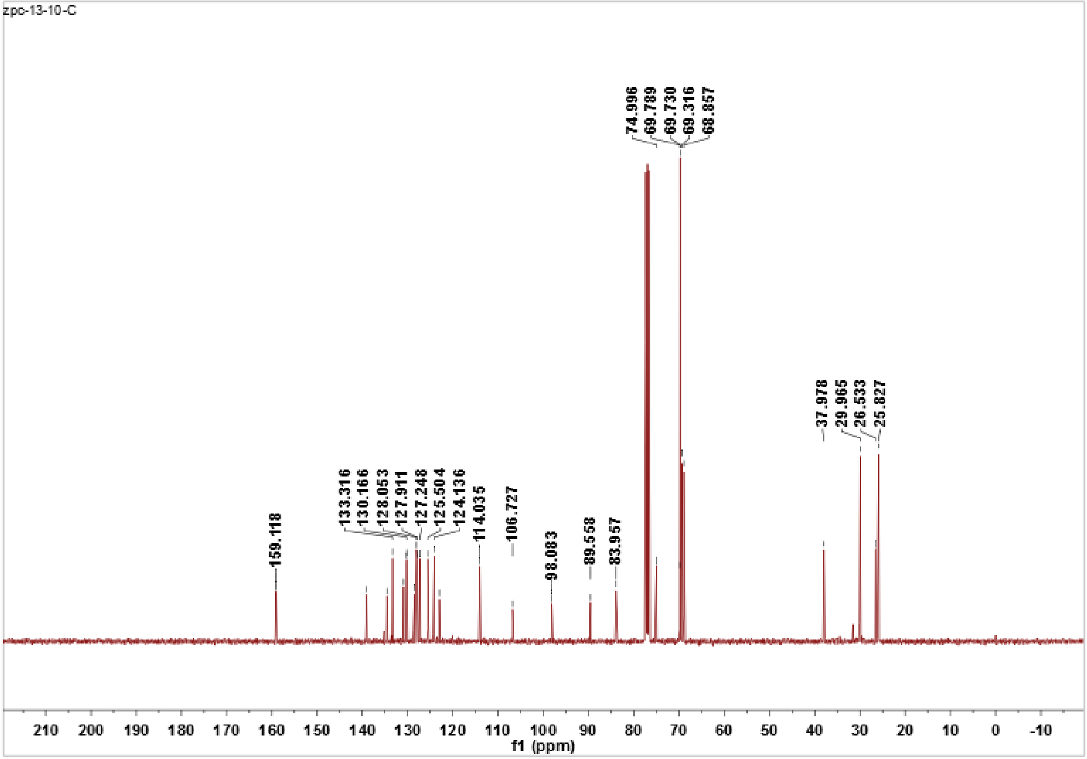

Supplementary Figure 46. ^{13}C NMR (400 MHz, $CDCl_3$) spectra for compound **1an**

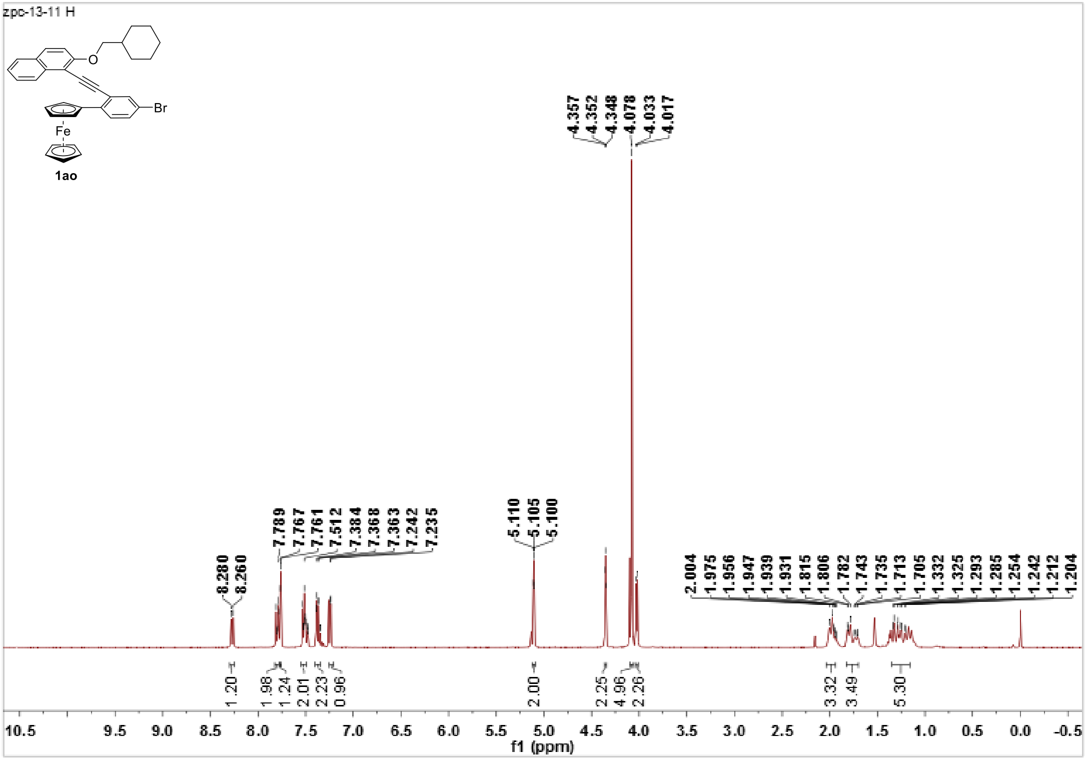

Supplementary Figure 47. ^{1}H NMR (400 MHz, $CDCl_3$) spectra for compound **1ao**

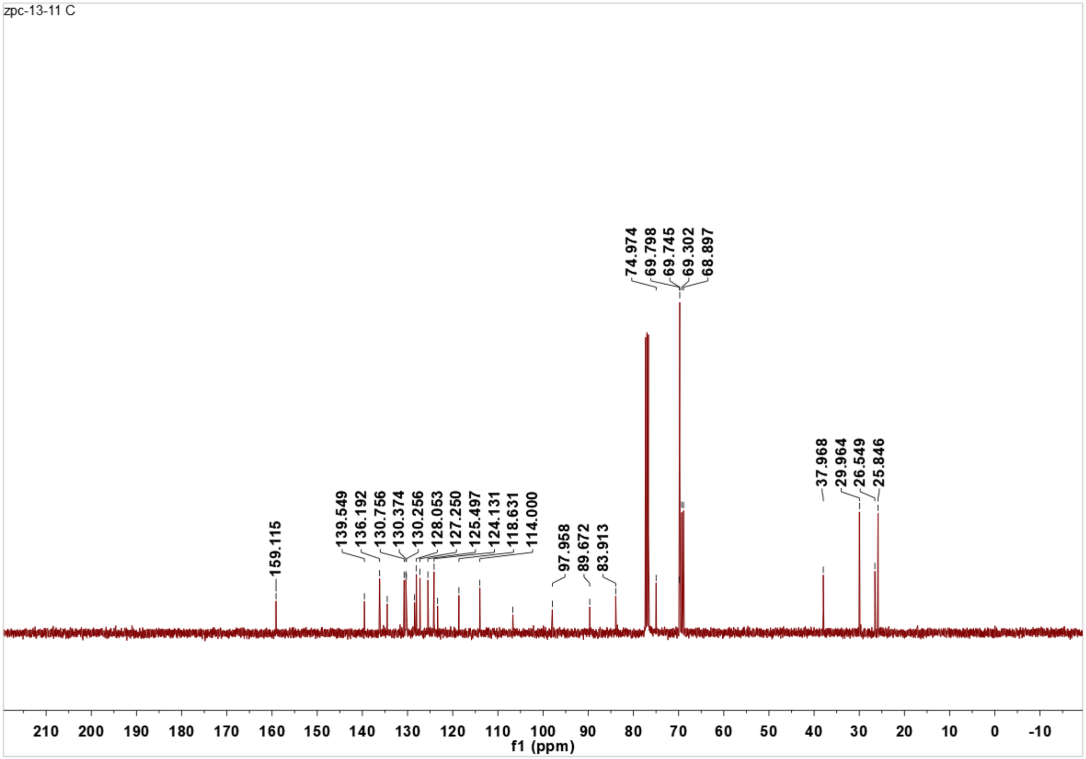

Supplementary Figure 48. ^{13}C NMR (400 MHz, $CDCl_3$) spectra for compound **1ao**

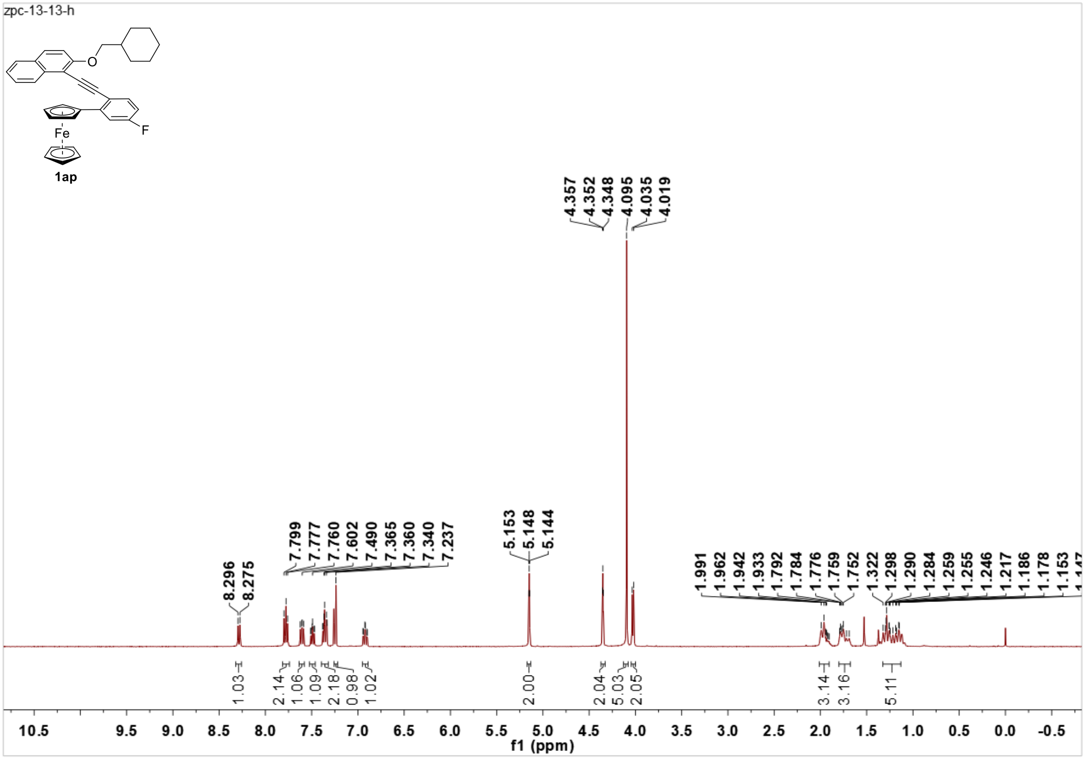

Supplementary Figure 49. ^{1}H NMR (400 MHz, $CDCl_3$) spectra for compound **1ap**

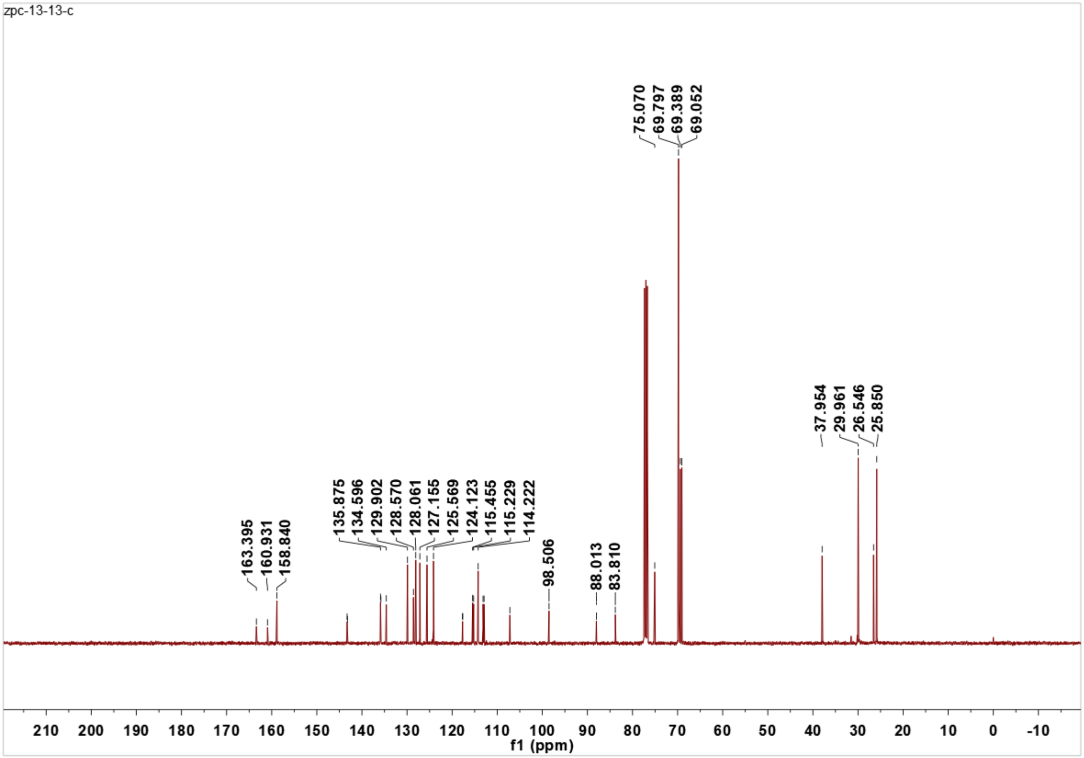

Supplementary Figure 50. ^{13}C NMR (400 MHz, $CDCl_3$) spectra for compound **1ap**

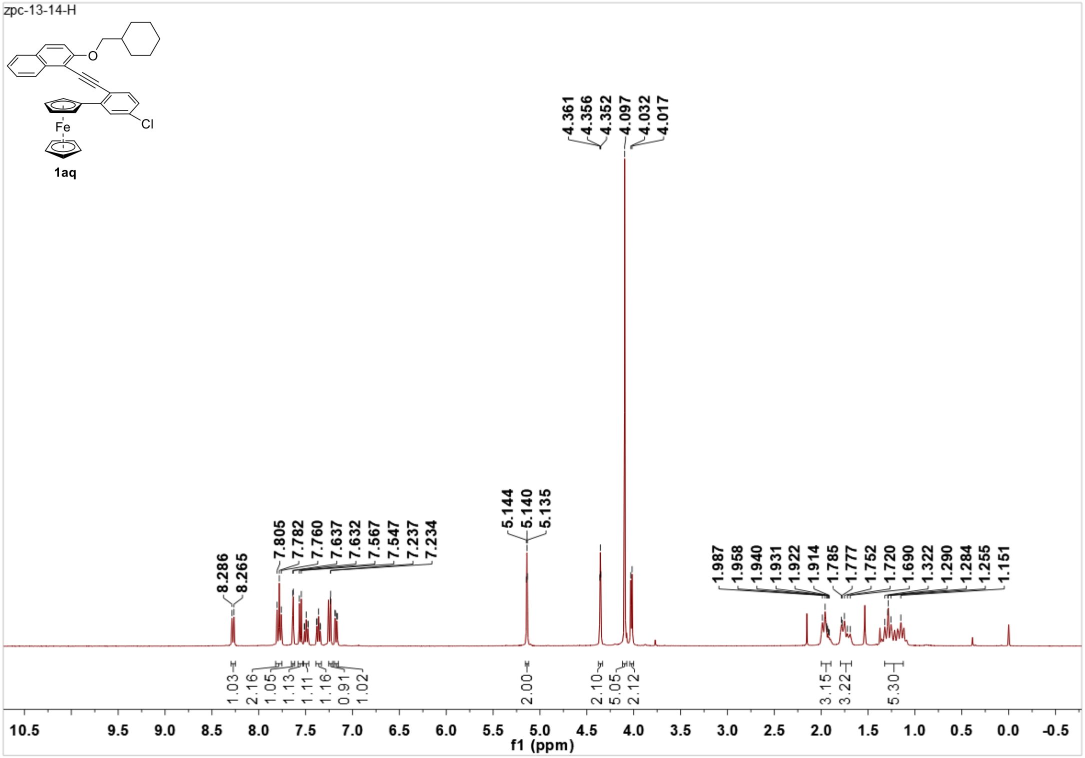

Supplementary Figure 51. ^{1}H NMR (400 MHz, $CDCl_3$) spectra for compound **1aq**

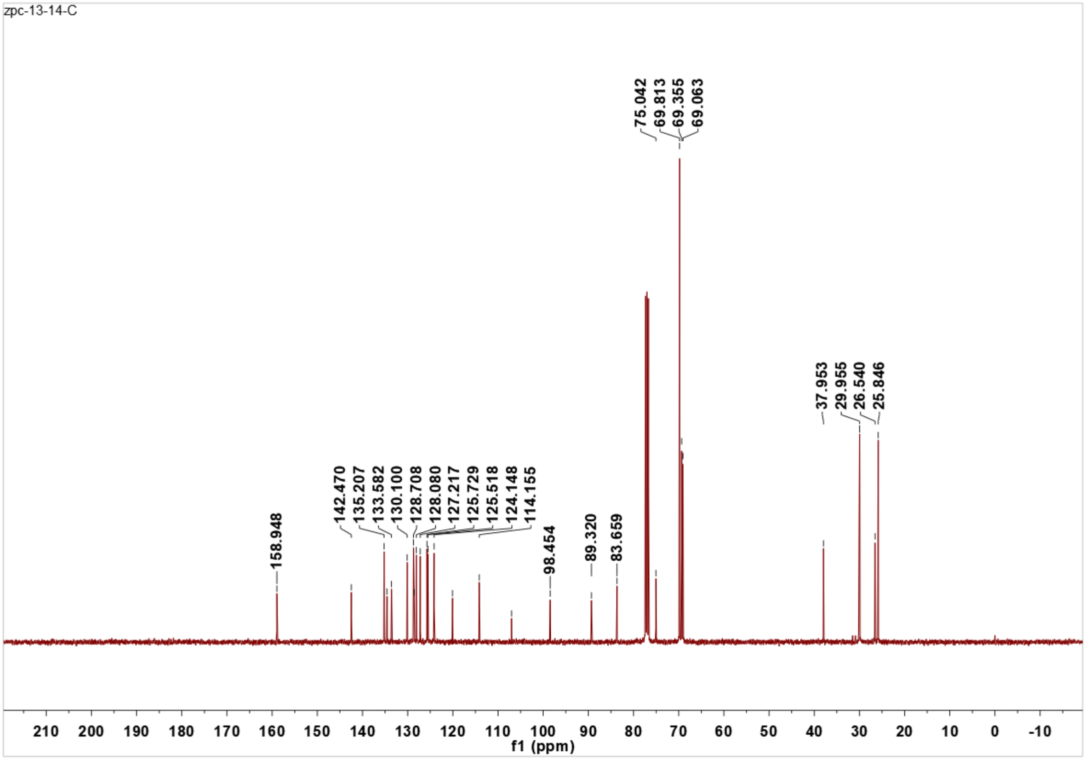

Supplementary Figure 52. ^{13}C NMR (400 MHz, $CDCl_3$) spectra for compound **1aq**

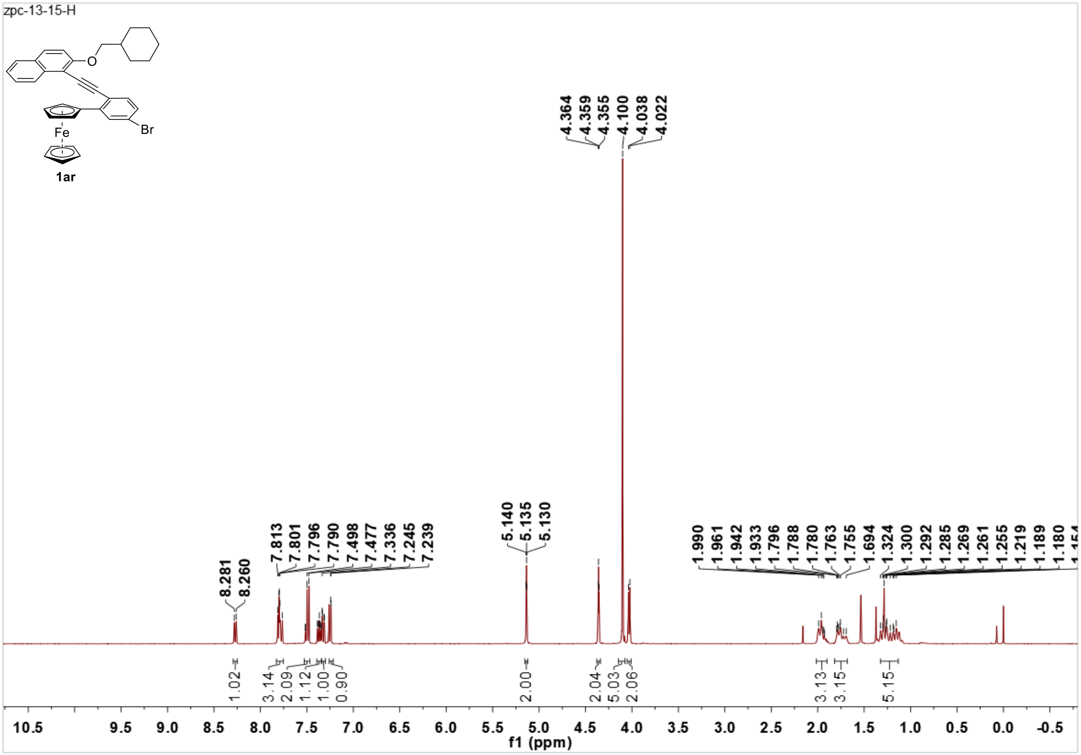

Supplementary Figure 53. ^{1}H NMR (400 MHz, $CDCl_3$) spectra for compound **1ar**

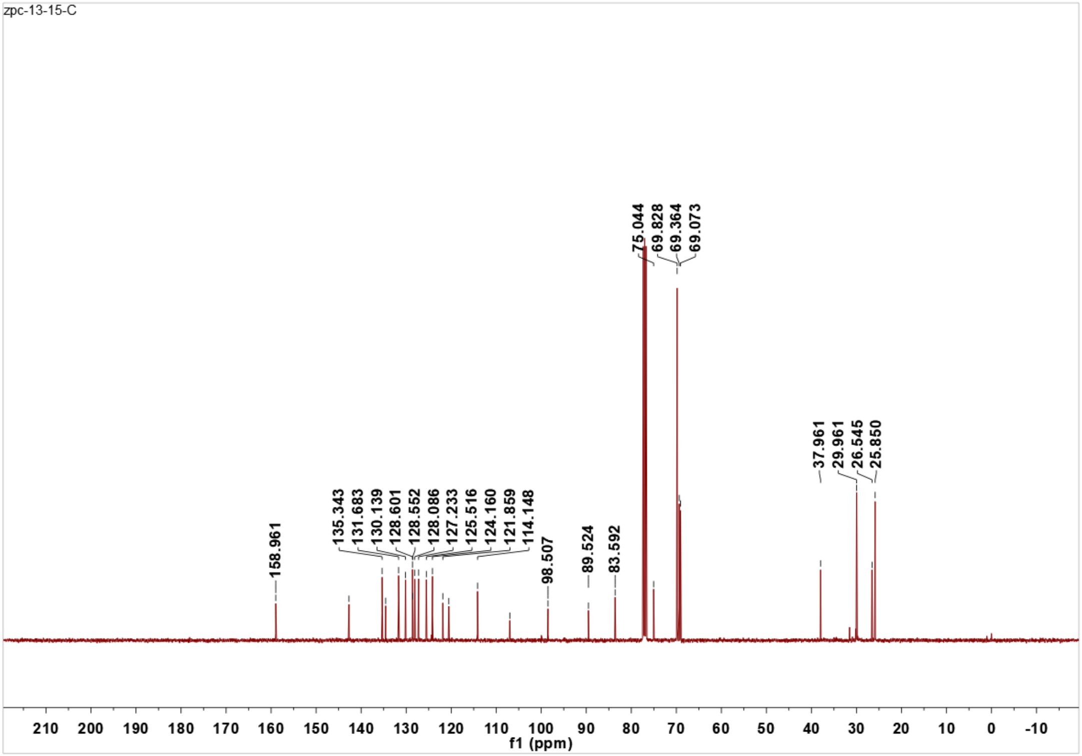

Supplementary Figure 54. ^{13}C NMR (400 MHz, $CDCl_3$) spectra for compound **1ar**

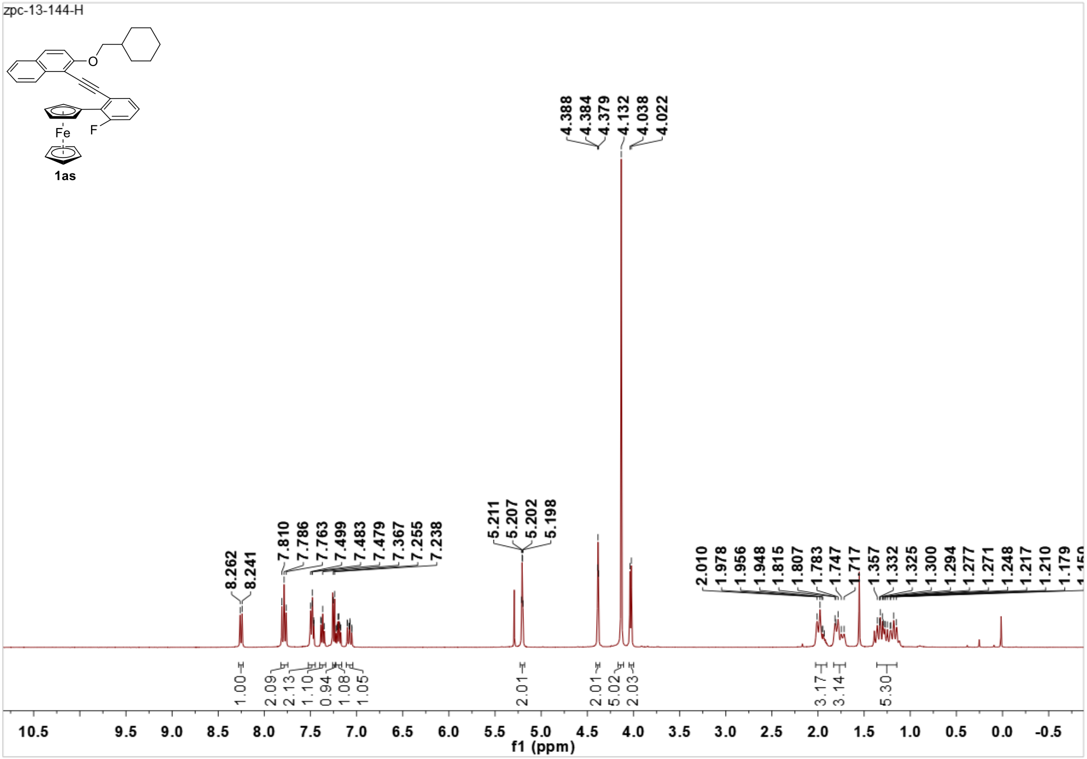

Supplementary Figure 55. ^{1}H NMR (400 MHz, $CDCl_3$) spectra for compound **1as**

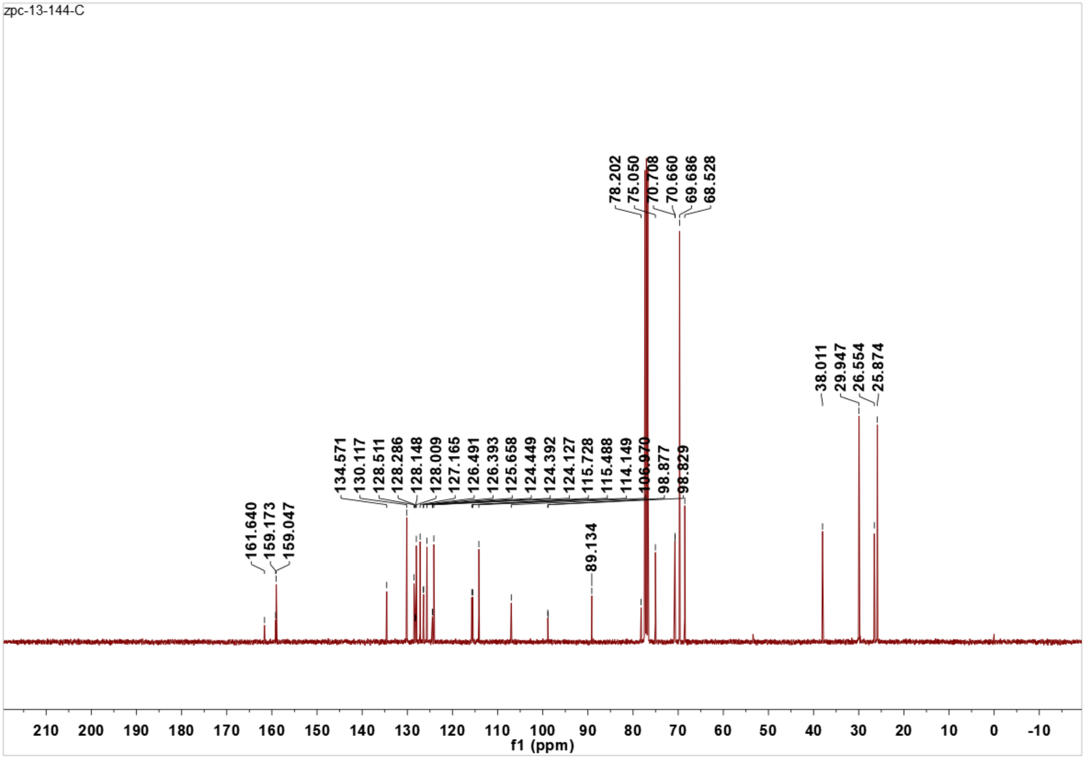

Supplementary Figure 56. ^{13}C NMR (400 MHz, $CDCl_3$) spectra for compound **1as**

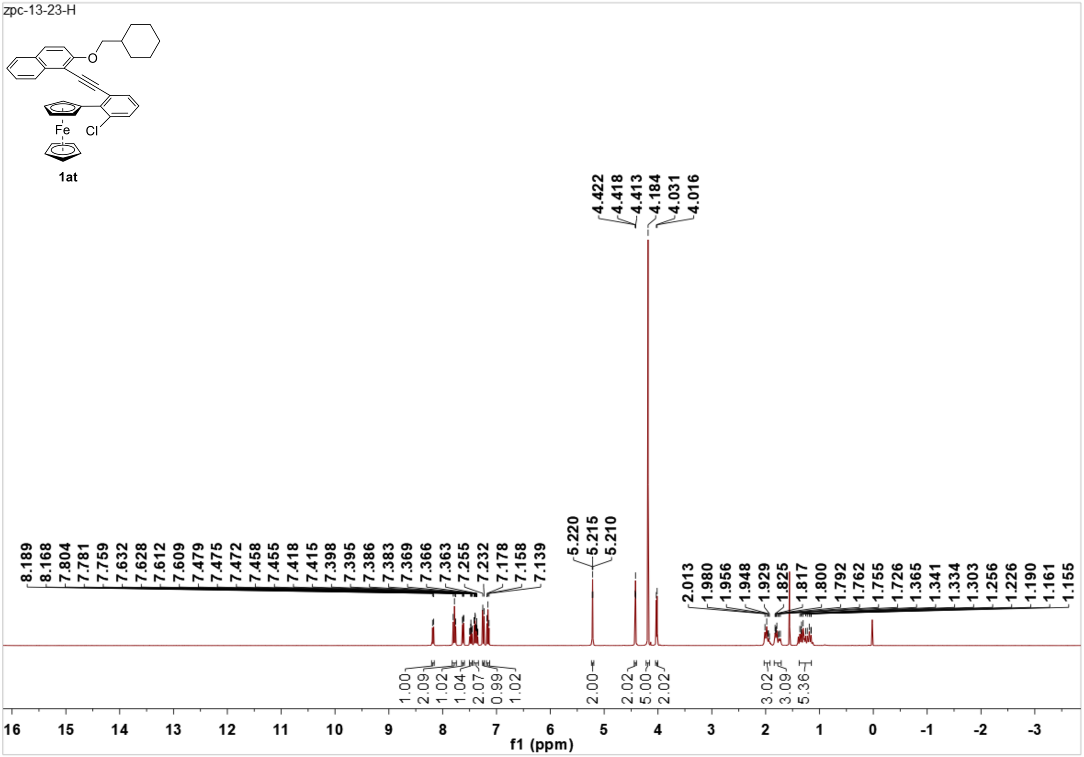

Supplementary Figure 57. ^{1}H NMR (400 MHz, $CDCl_3$) spectra for compound **1at**

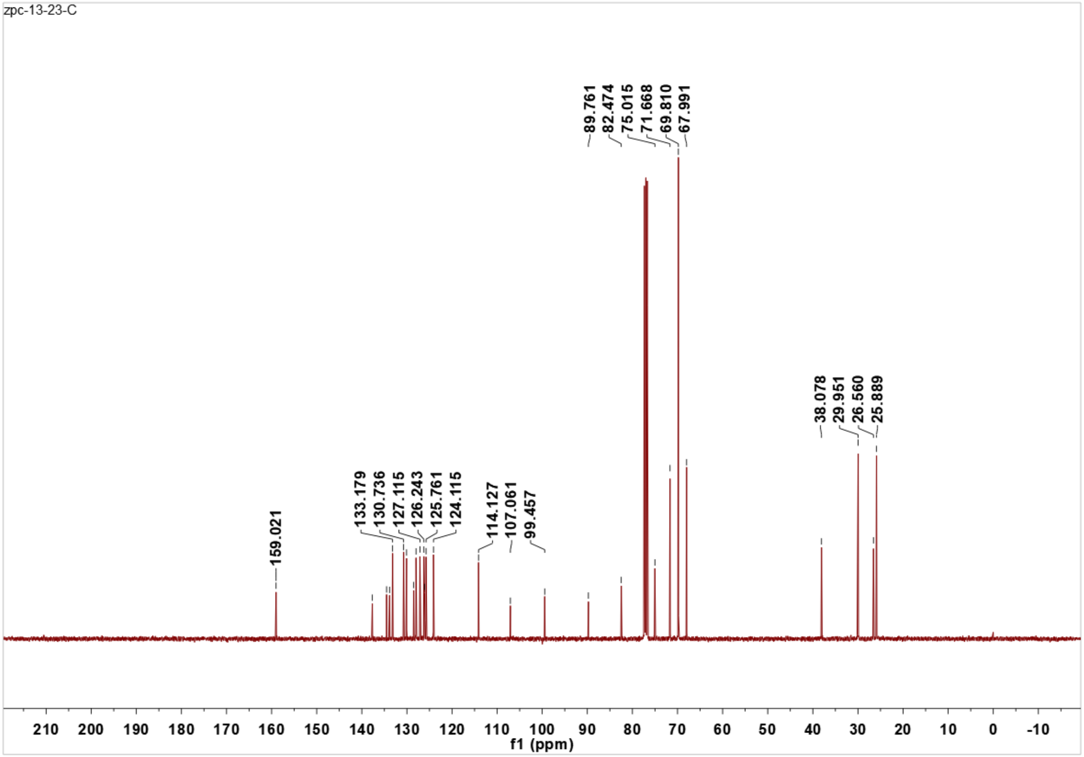

Supplementary Figure 58. ^{13}C NMR (400 MHz, $CDCl_3$) spectra for compound **1at**

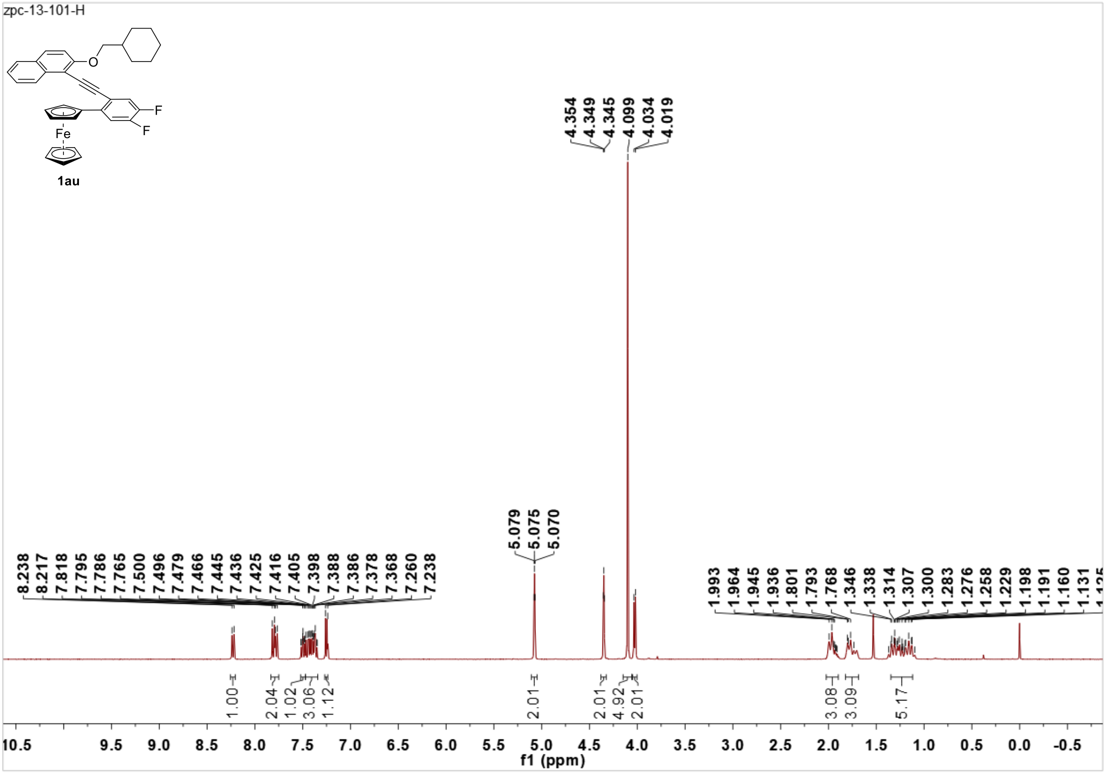

Supplementary Figure 59. ^{1}H NMR (400 MHz, $CDCl_3$) spectra for compound **1au**

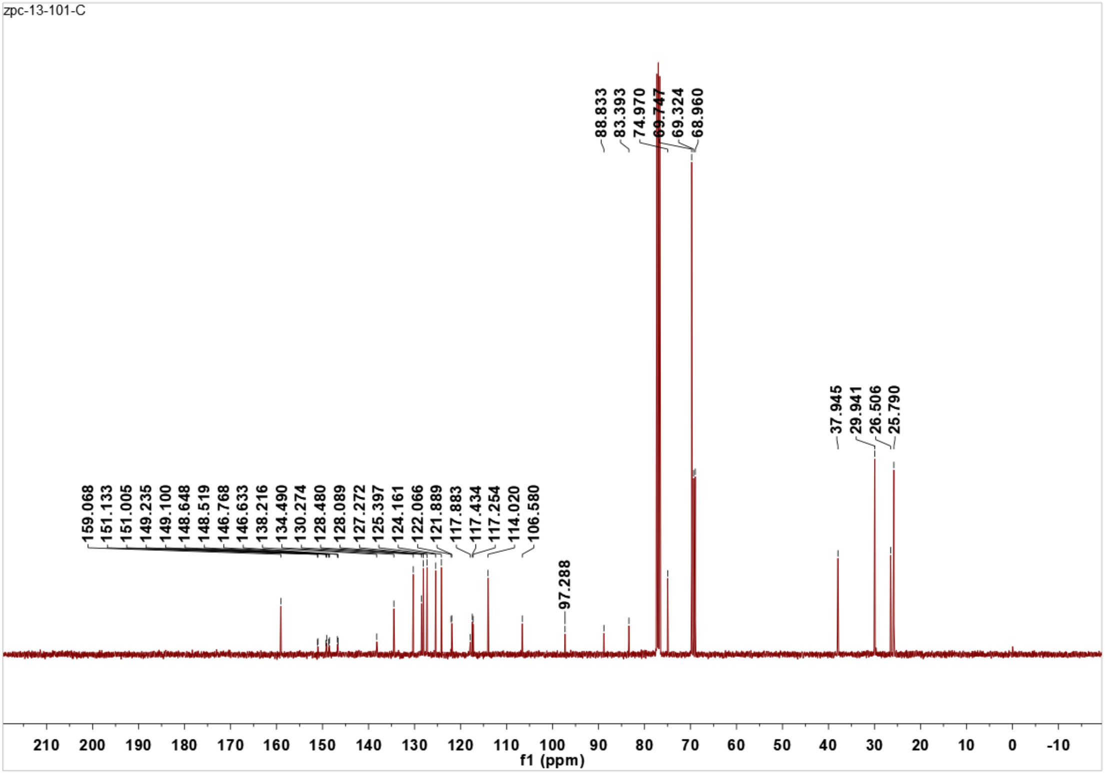

Supplementary Figure 60. ^{13}C NMR (400 MHz, $CDCl_3$) spectra for compound **1au**

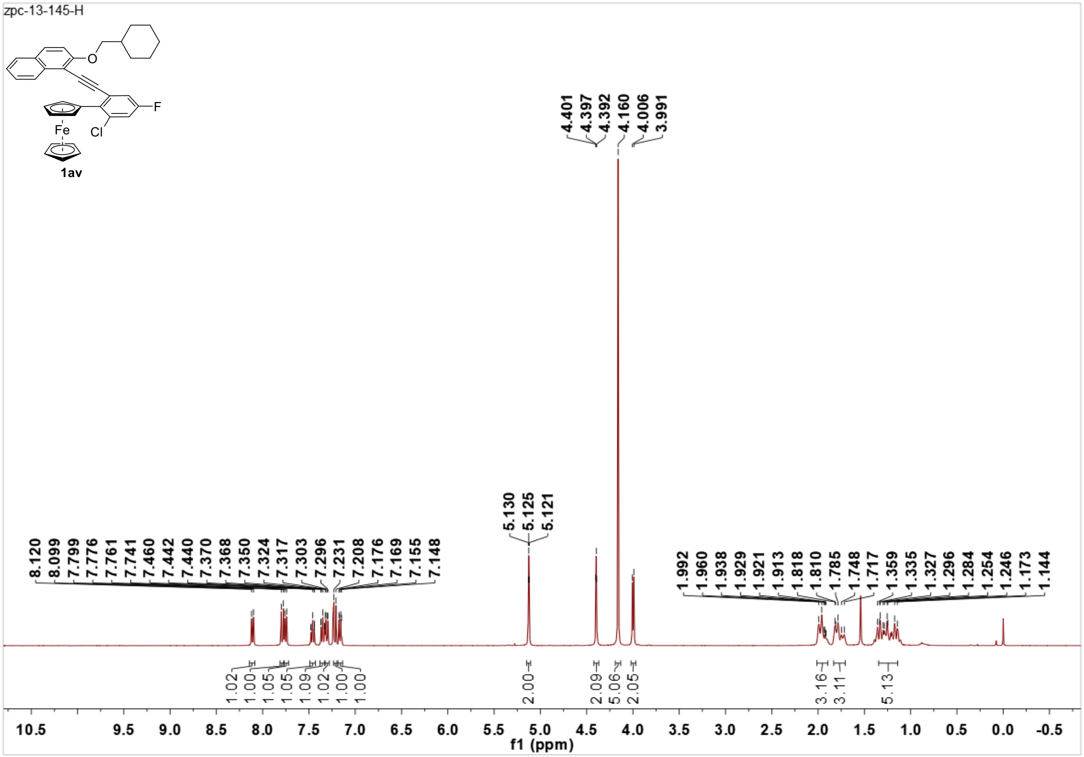

Supplementary Figure 61. ^{1}H NMR (400 MHz, $CDCl_3$) spectra for compound **1av**

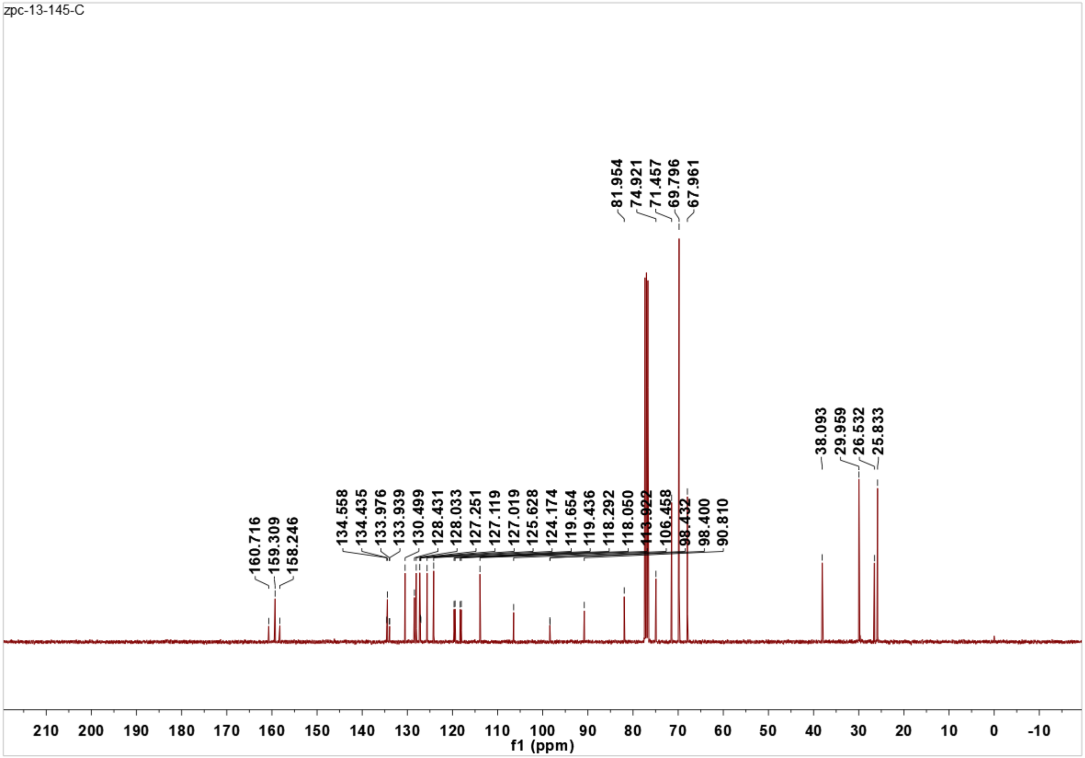

Supplementary Figure 62. ^{13}C NMR (400 MHz, $CDCl_3$) spectra for compound **1av**

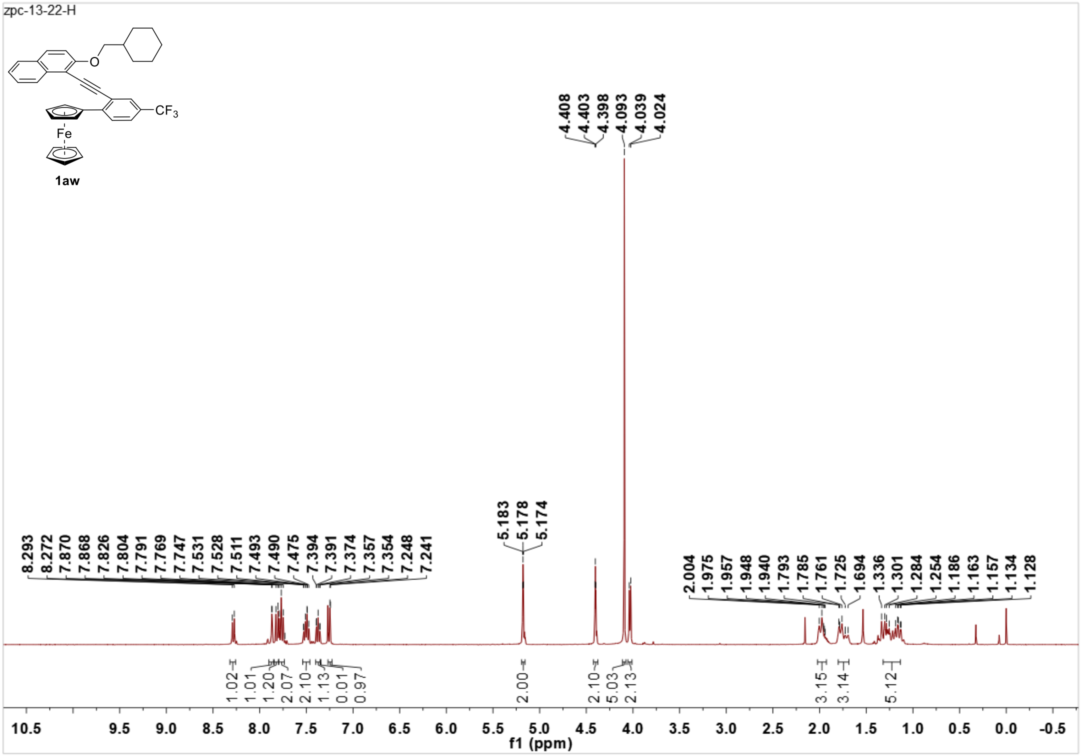

Supplementary Figure 63. ^{1}H NMR (400 MHz, $CDCl_3$) spectra for compound **1aw**

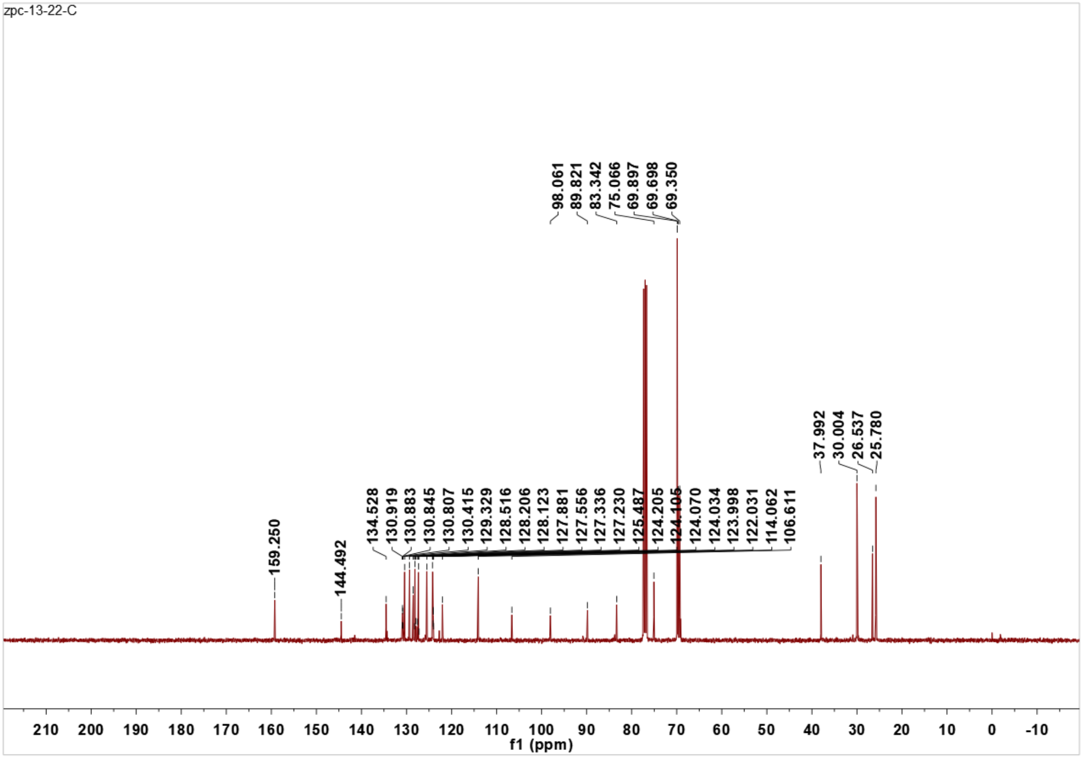

Supplementary Figure 64. ^{13}C NMR (400 MHz, $CDCl_3$) spectra for compound **1aw**

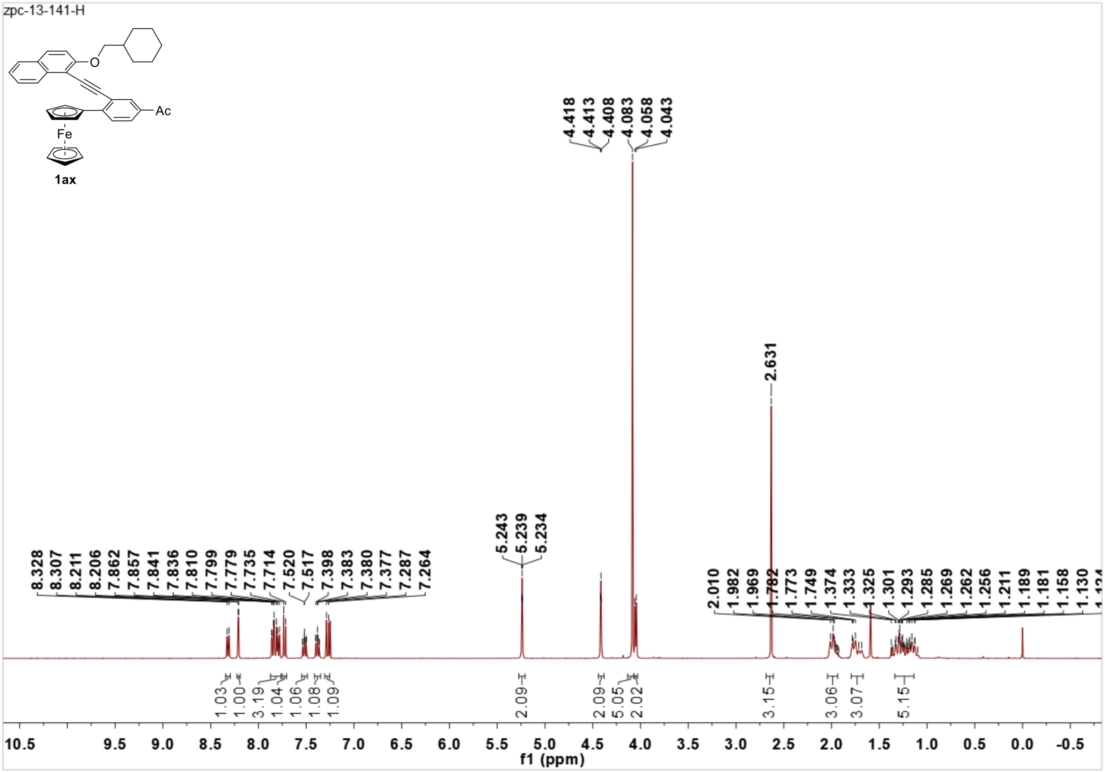

Supplementary Figure 65. ^{1}H NMR (400 MHz, $CDCl_3$) spectra for compound **1ax**

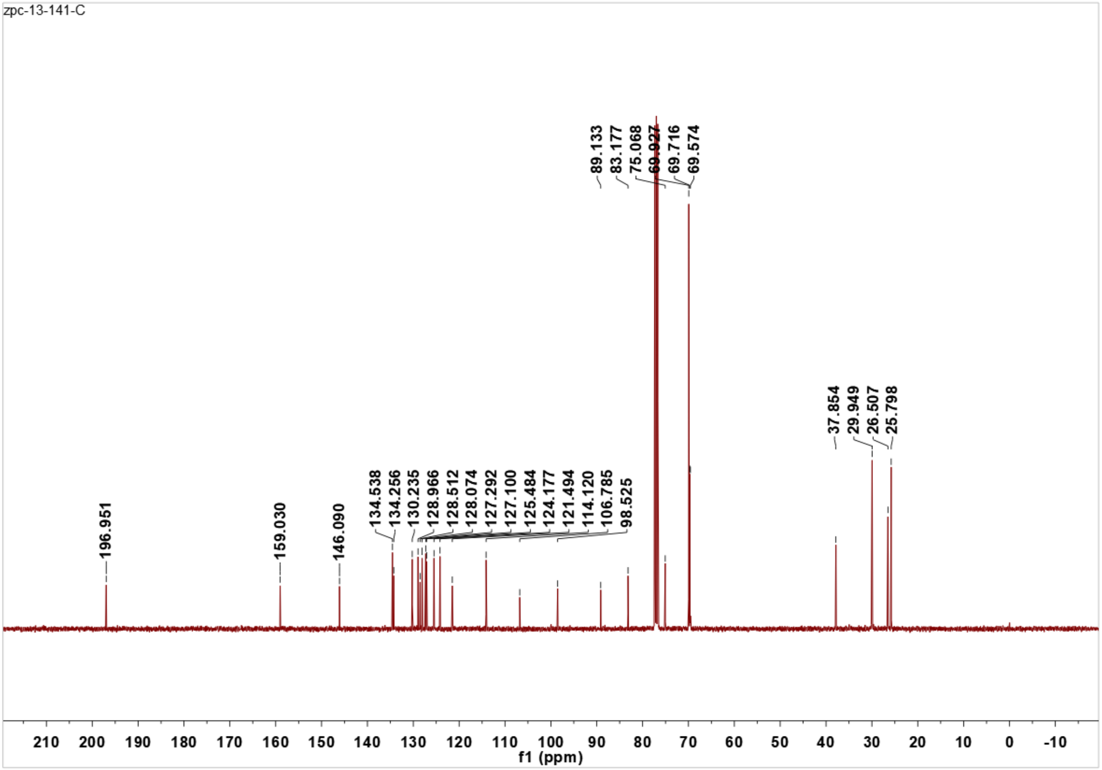

Supplementary Figure 66. ^{13}C NMR (400 MHz, $CDCl_3$) spectra for compound **1ax**

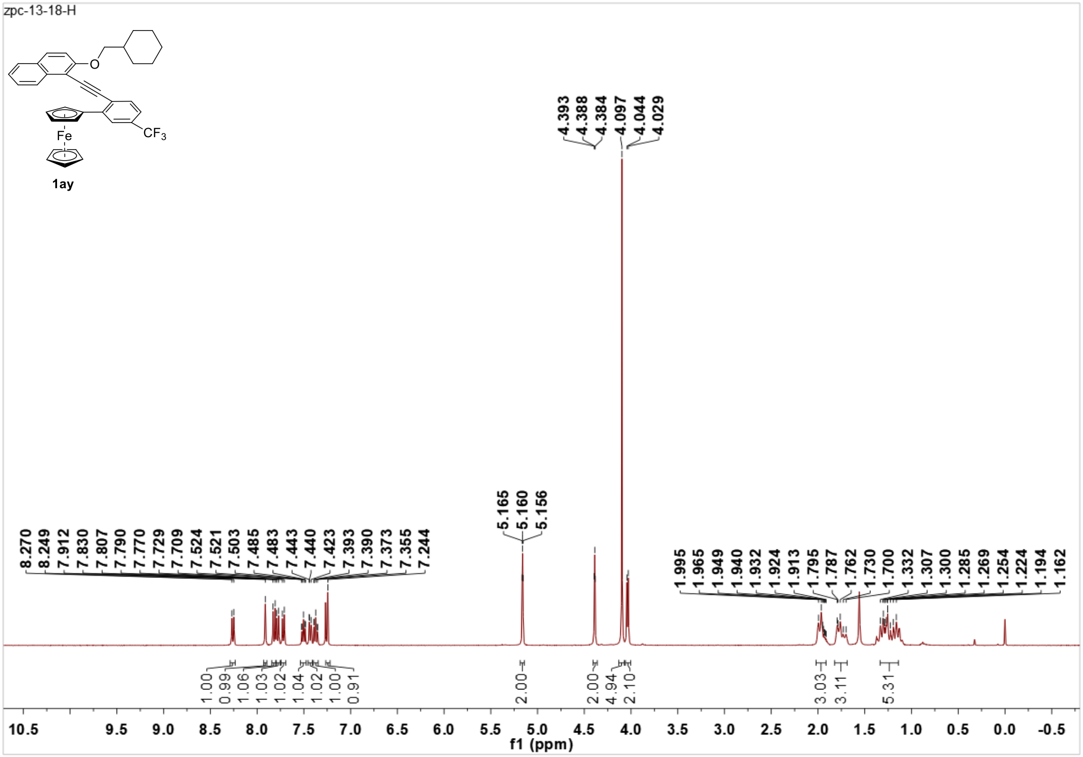

Supplementary Figure 67. ^{1}H NMR (400 MHz, $CDCl_3$) spectra for compound **1ay**

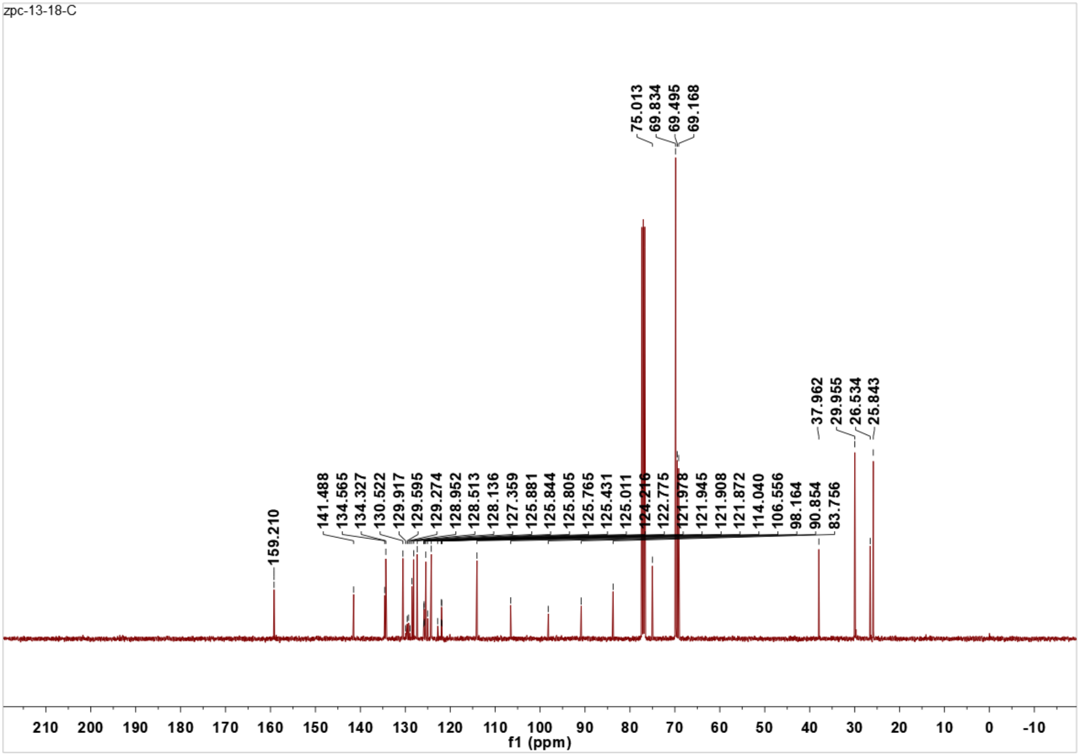

Supplementary Figure 68. ^{13}C NMR (400 MHz, $CDCl_3$) spectra for compound **1ay**

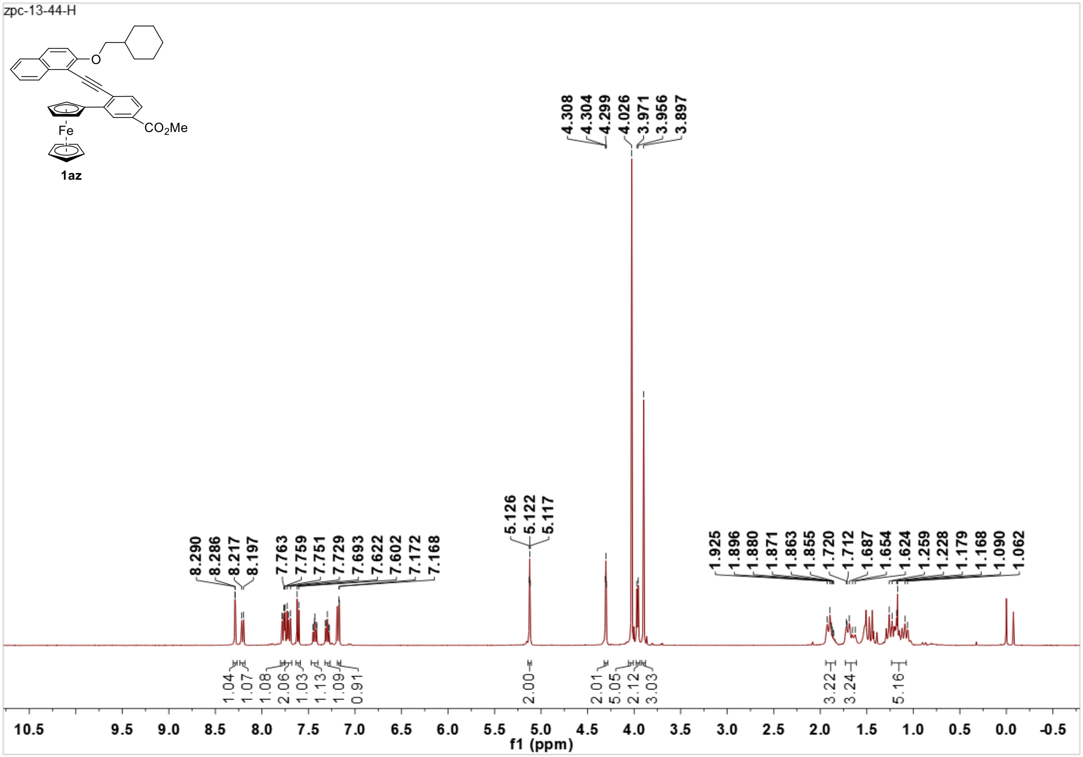

Supplementary Figure 69. ^{1}H NMR (400 MHz, $CDCl_3$) spectra for compound **1az**

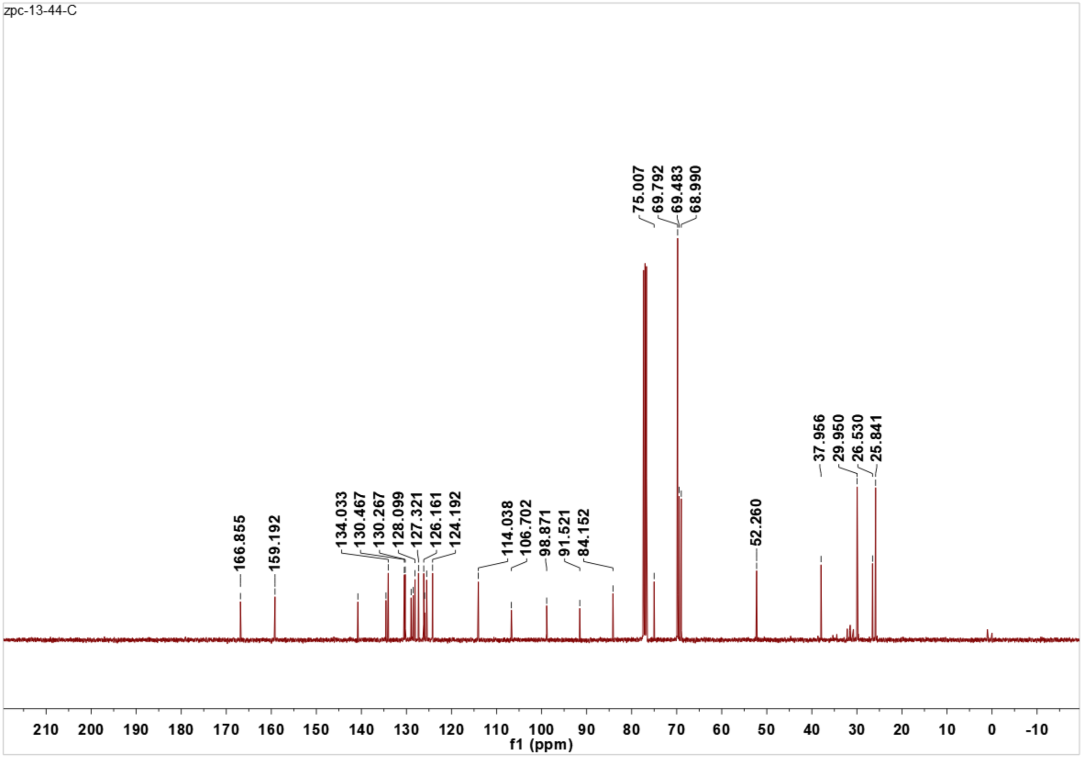

Supplementary Figure 70. ^{13}C NMR (400 MHz, $CDCl_3$) spectra for compound **1az**

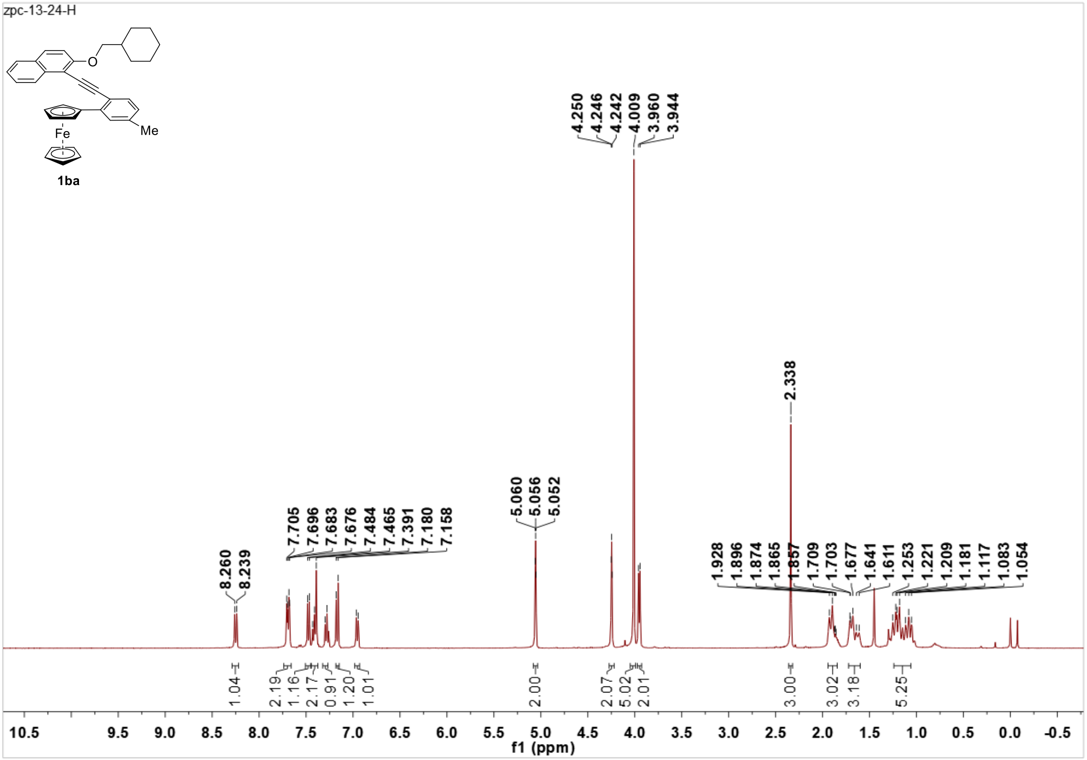

Supplementary Figure 71. ^{1}H NMR (400 MHz, $CDCl_3$) spectra for compound **1ba**

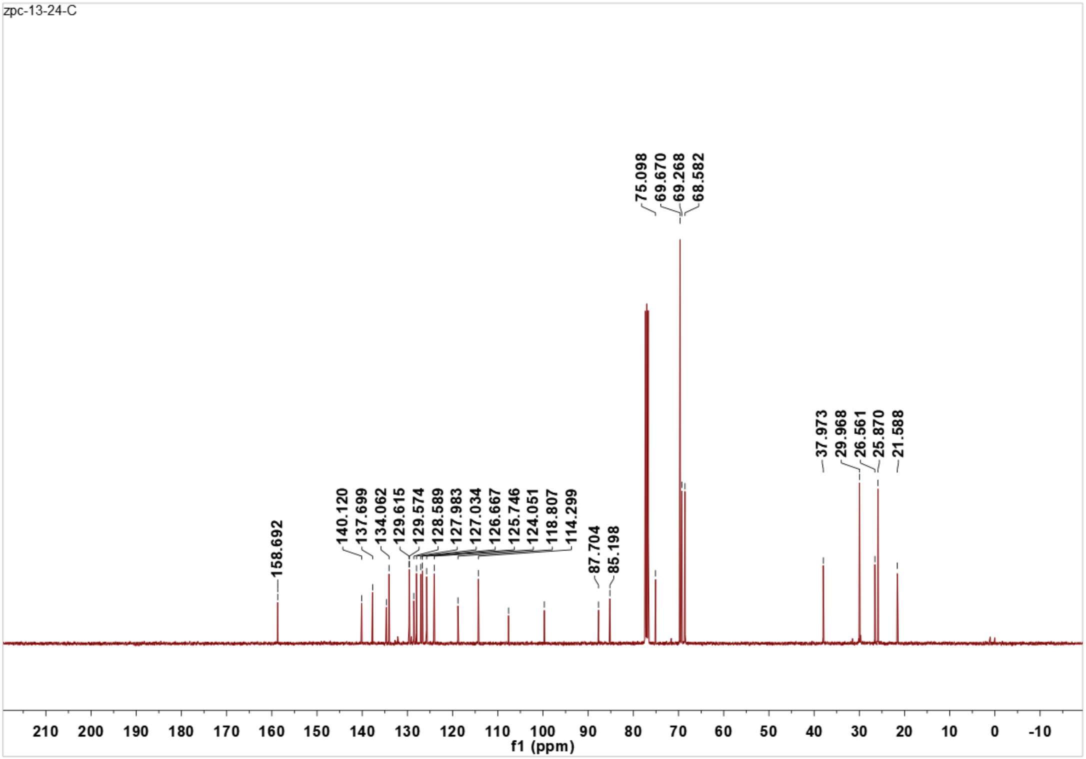

Supplementary Figure 72. ^{13}C NMR (400 MHz, $CDCl_3$) spectra for compound **1ba**

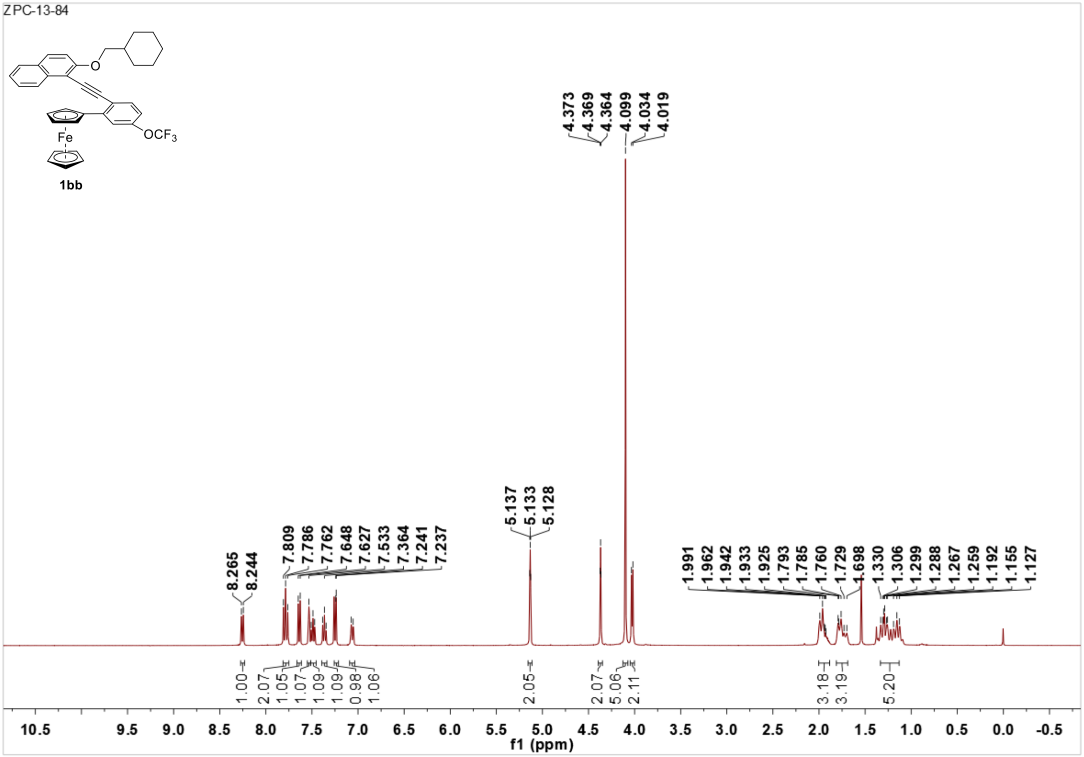

Supplementary Figure 73. ^{1}H NMR (400 MHz, $CDCl_3$) spectra for compound **1bb**

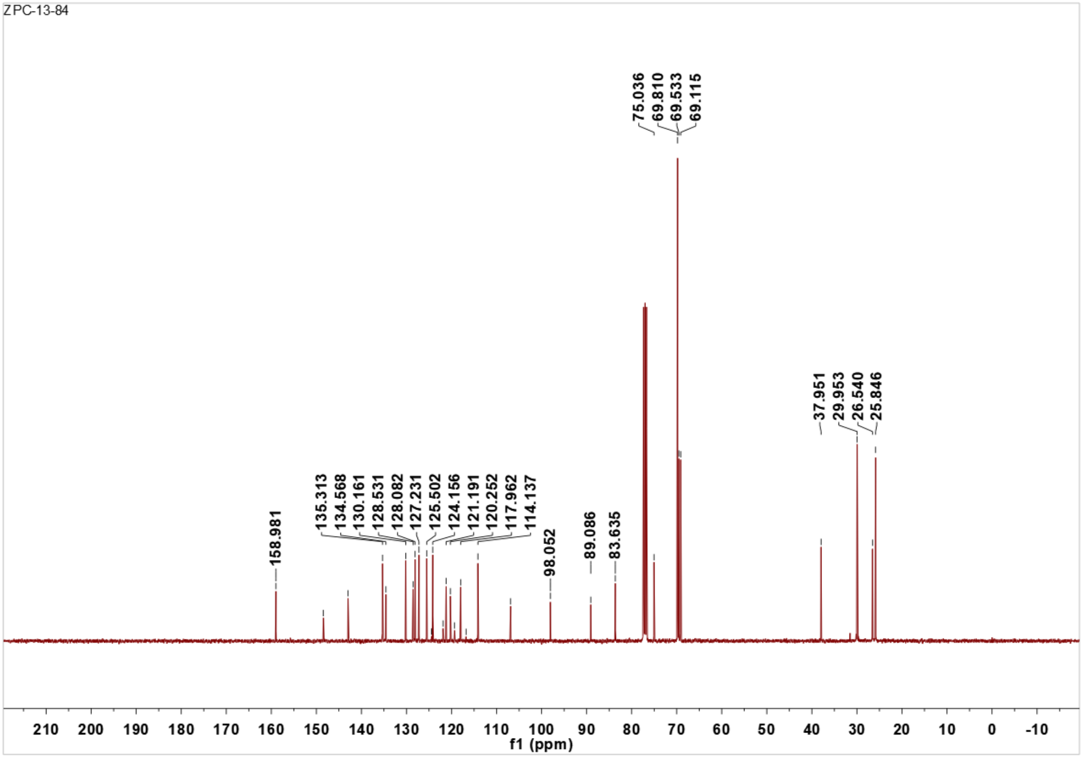

Supplementary Figure 74. ^{13}C NMR (400 MHz, $CDCl_3$) spectra for compound **1bb**

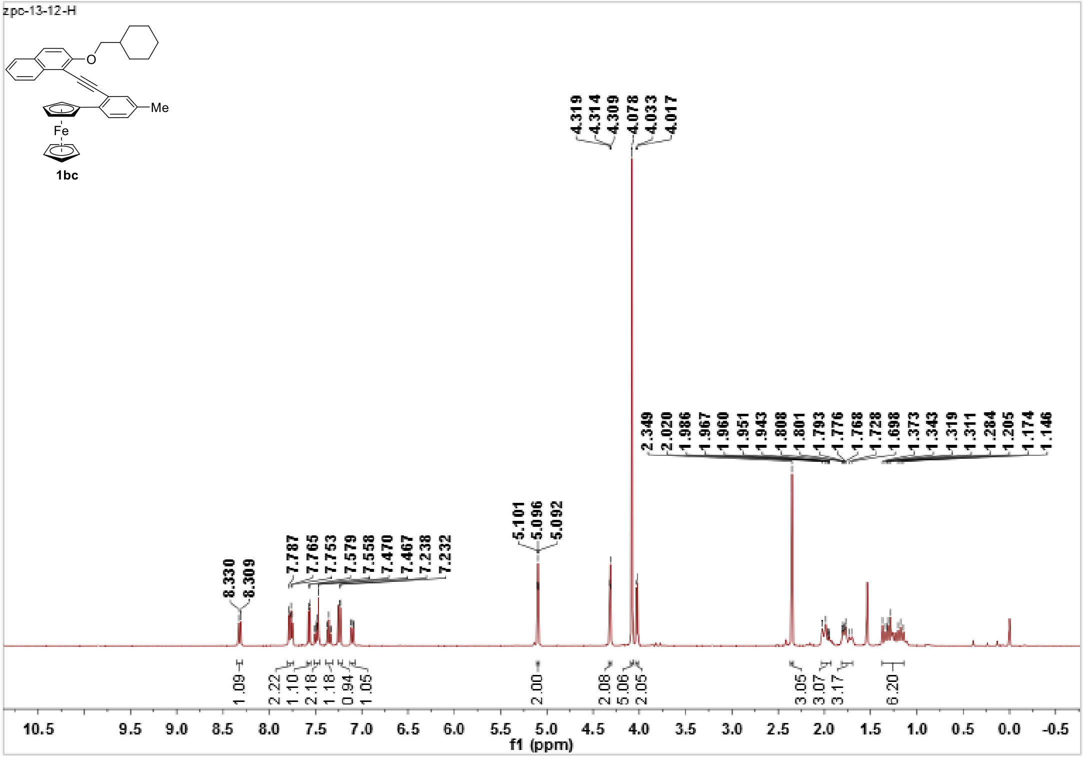

Supplementary Figure 75. ^{1}H NMR (400 MHz, $CDCl_3$) spectra for compound **1bc**

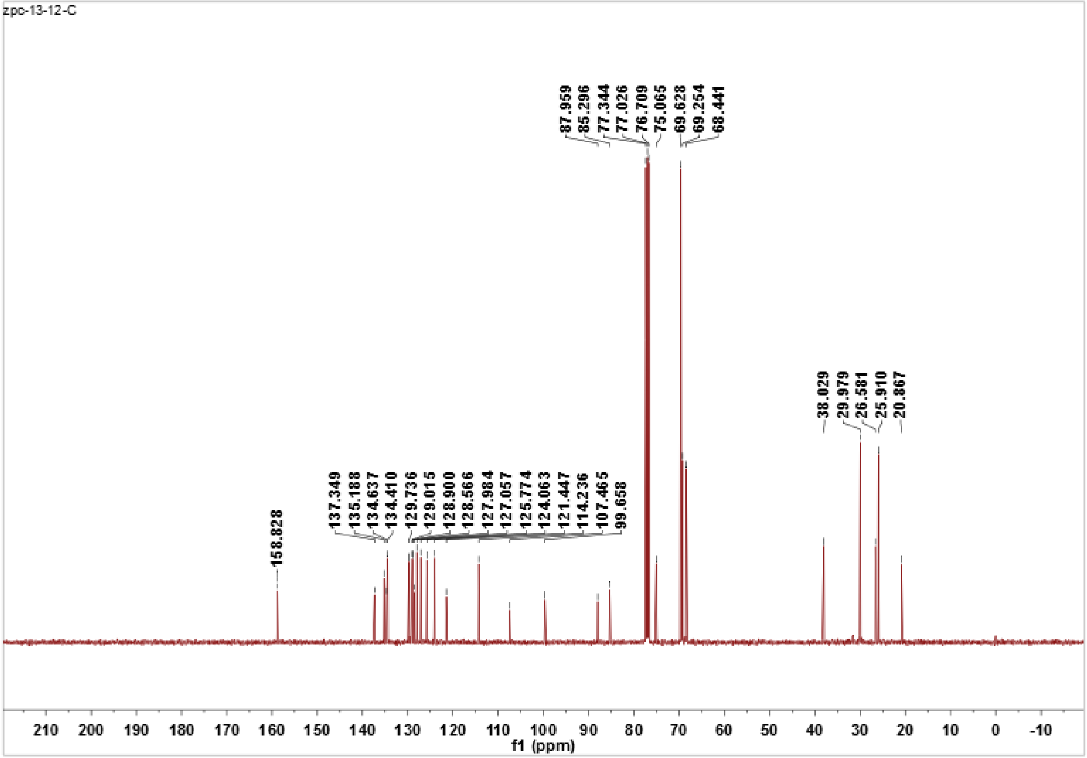

Supplementary Figure 76. ^{13}C NMR (400 MHz, $CDCl_3$) spectra for compound **1bc**

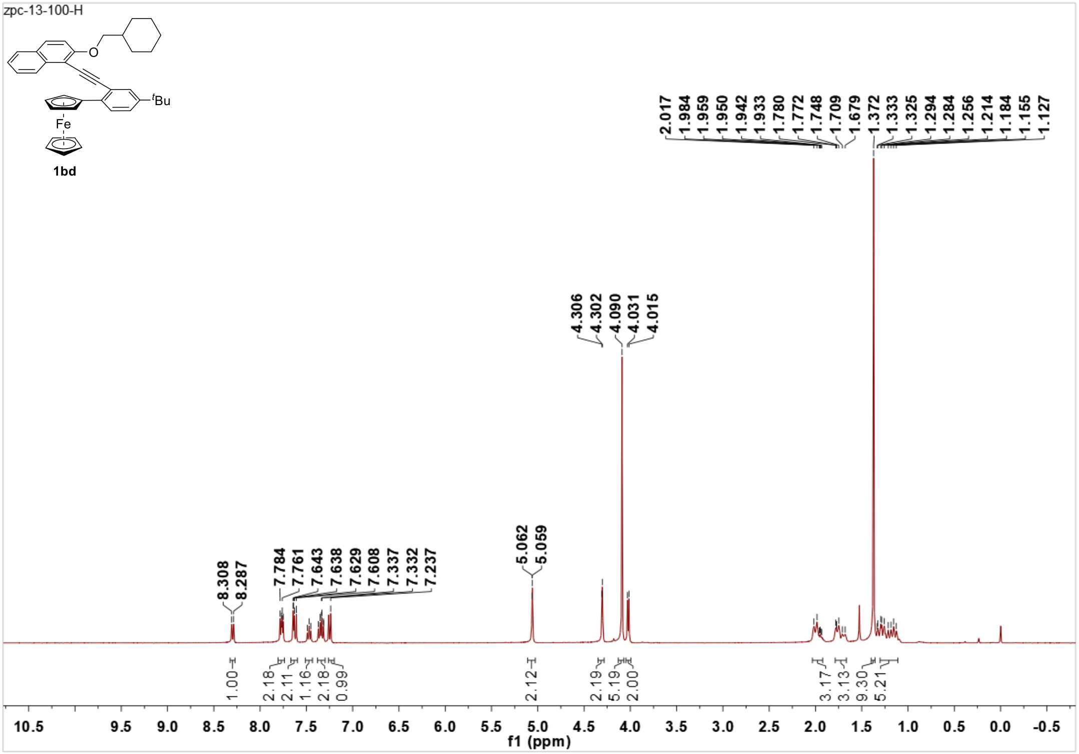

Supplementary Figure 77. ^{1}H NMR (400 MHz, $CDCl_3$) spectra for compound **1bd**

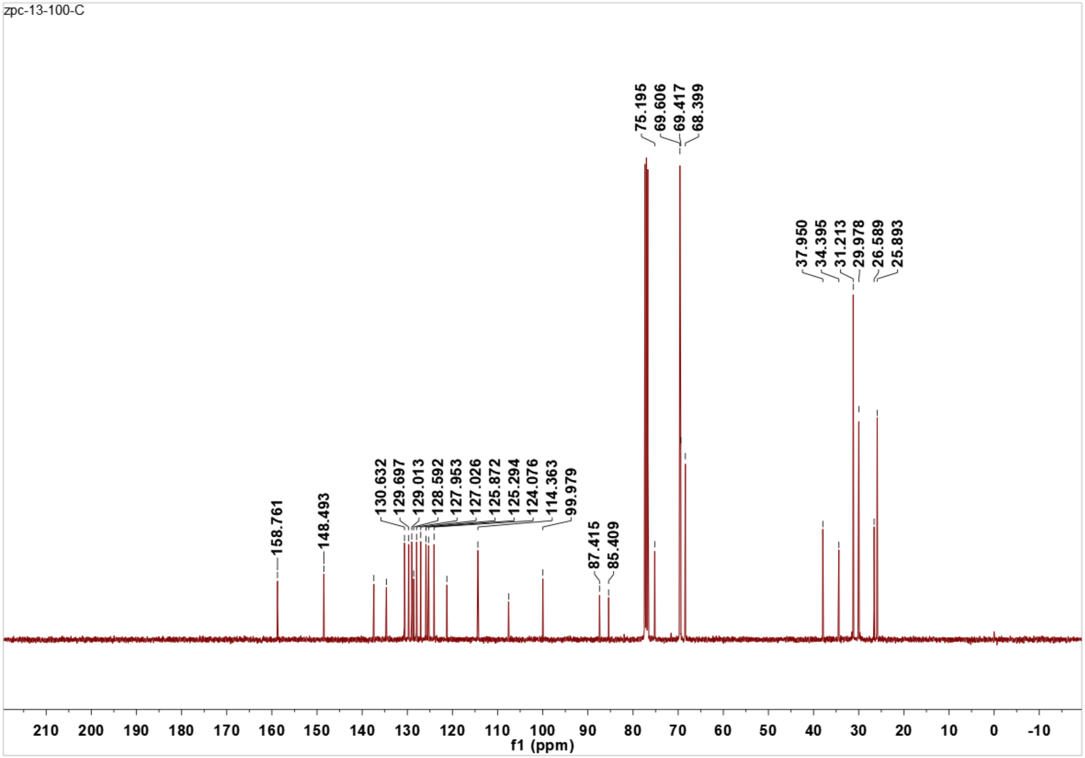

Supplementary Figure 78. ^{13}C NMR (400 MHz, $CDCl_3$) spectra for compound **1bd**

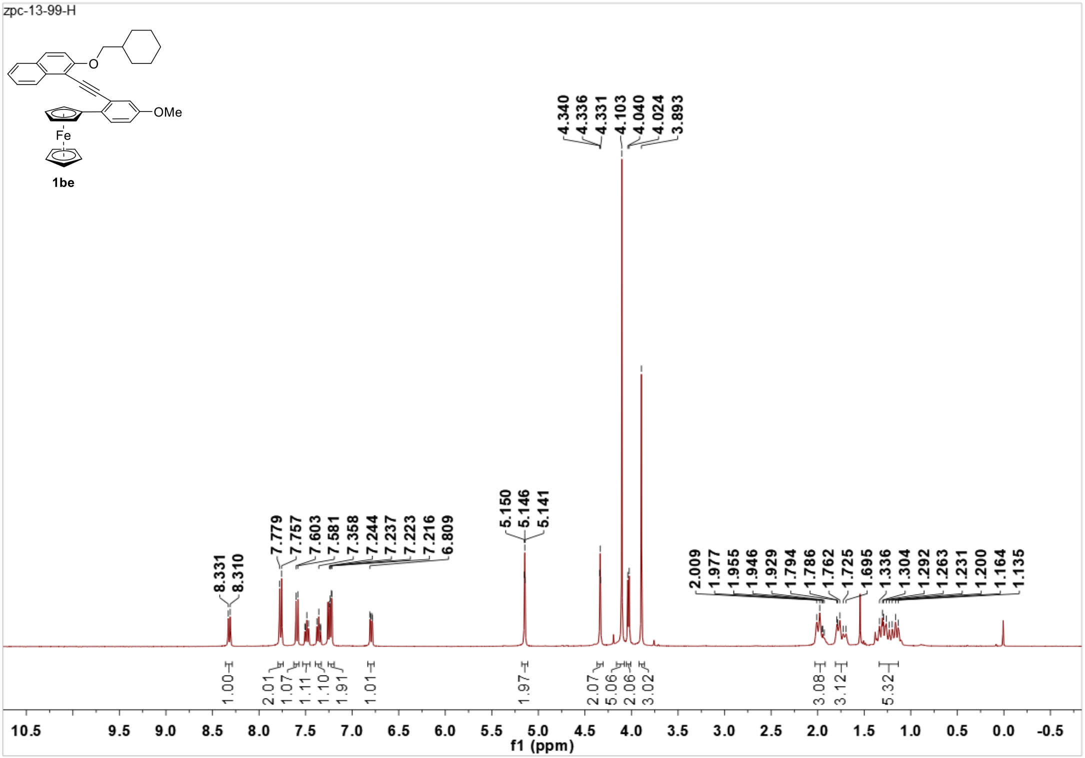

Supplementary Figure 79. ^{1}H NMR (400 MHz, $CDCl_3$) spectra for compound **1be**

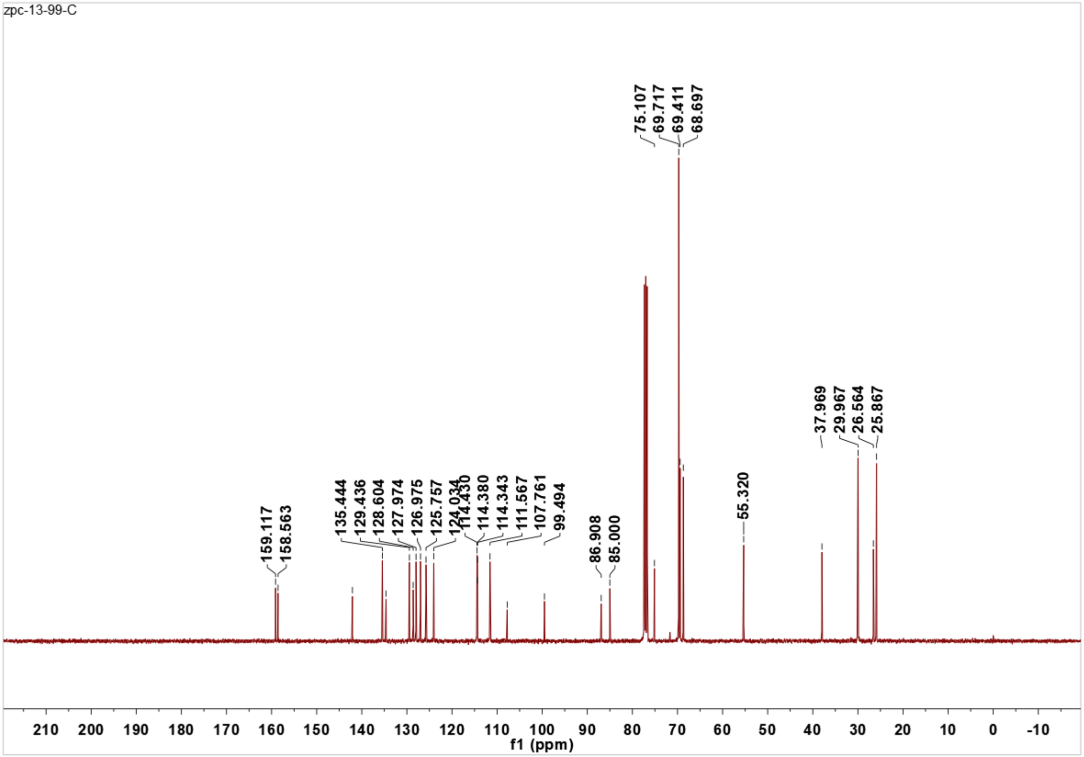

Supplementary Figure 80. ^{13}C NMR (400 MHz, $CDCl_3$) spectra for compound **1be**

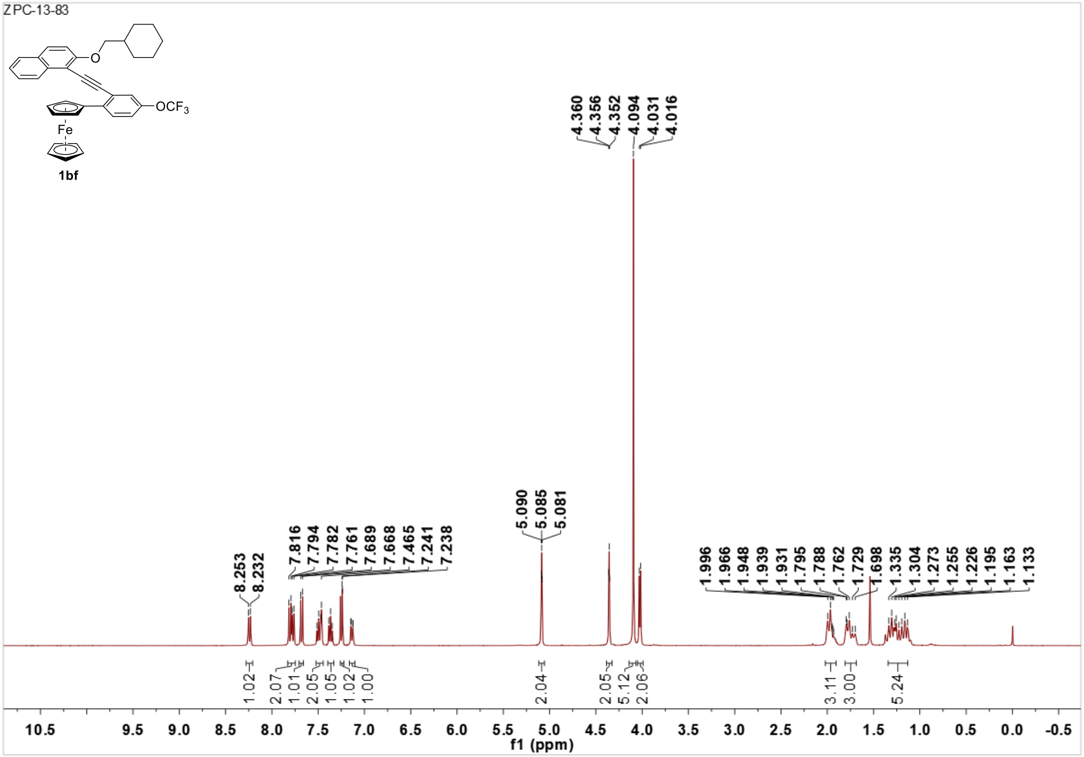

Supplementary Figure 81. ^{1}H NMR (400 MHz, $CDCl_3$) spectra for compound **1bf**

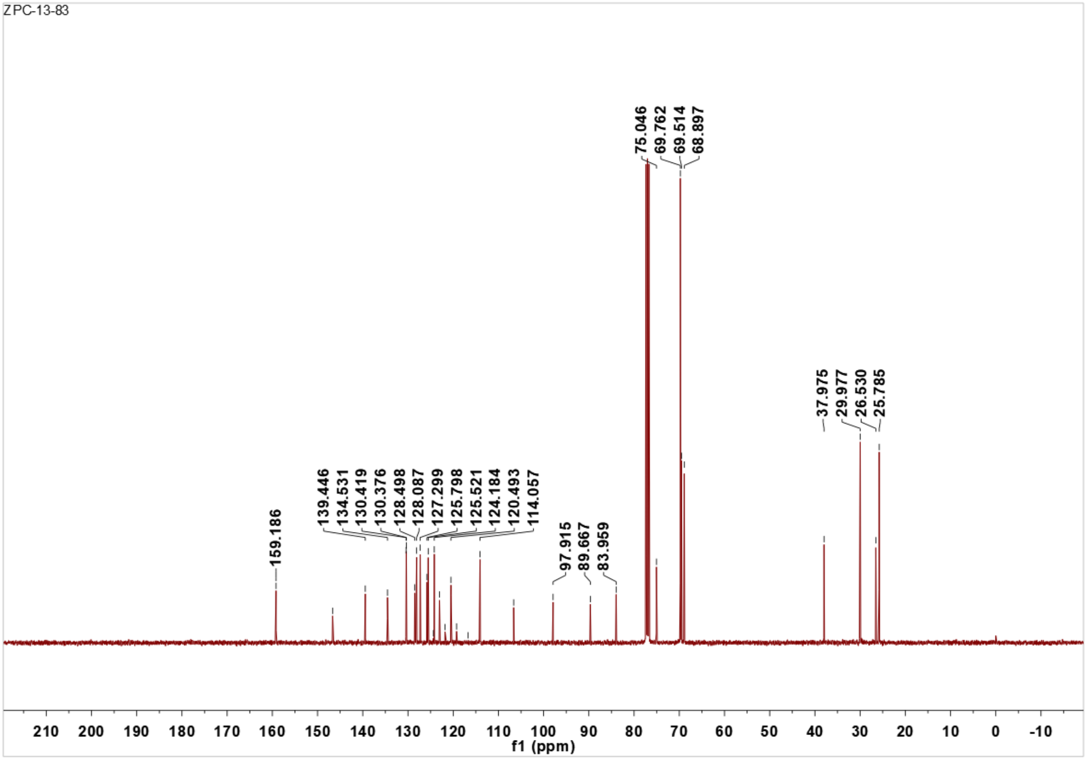

Supplementary Figure 82. ^{13}C NMR (400 MHz, $CDCl_3$) spectra for compound **1bf**

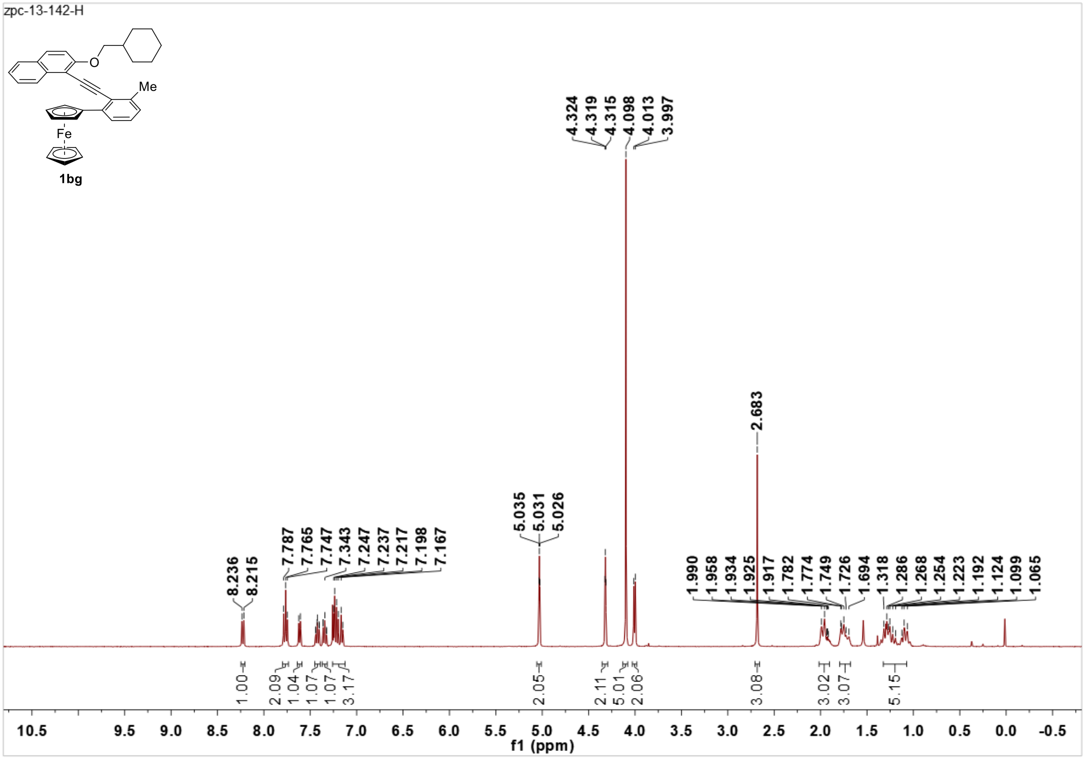

Supplementary Figure 83. ^{1}H NMR (400 MHz, $CDCl_3$) spectra for compound **1bg**

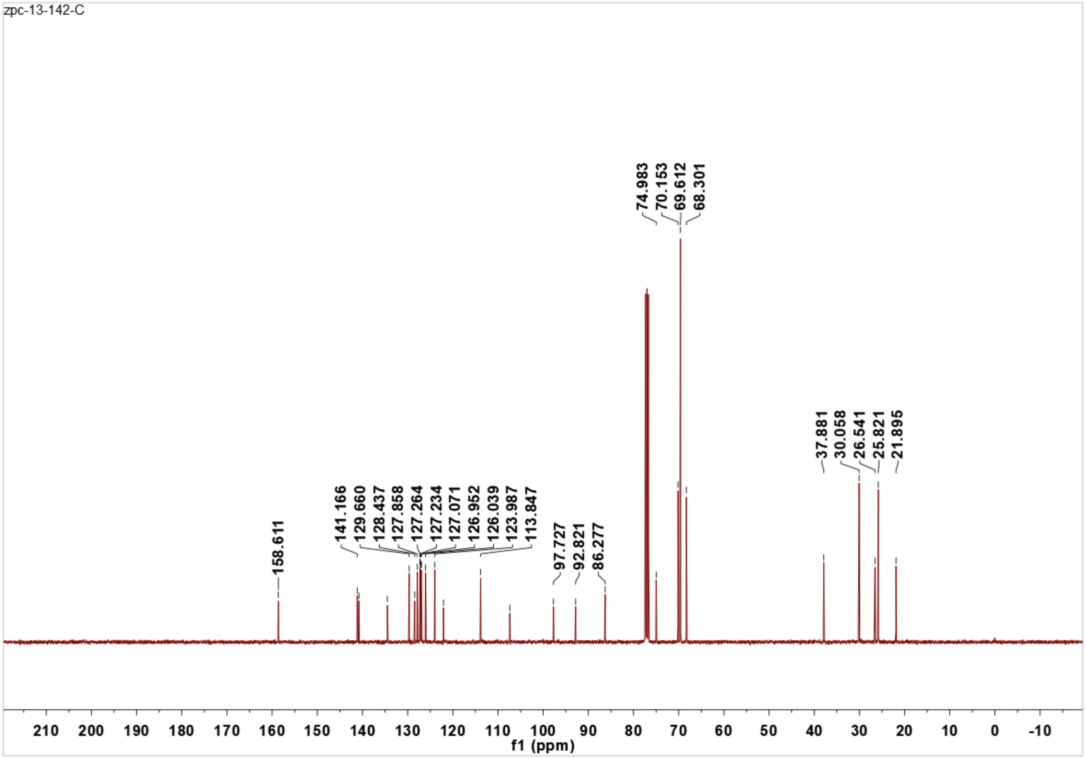

Supplementary Figure 84. ^{13}C NMR (400 MHz, $CDCl_3$) spectra for compound **1bg**

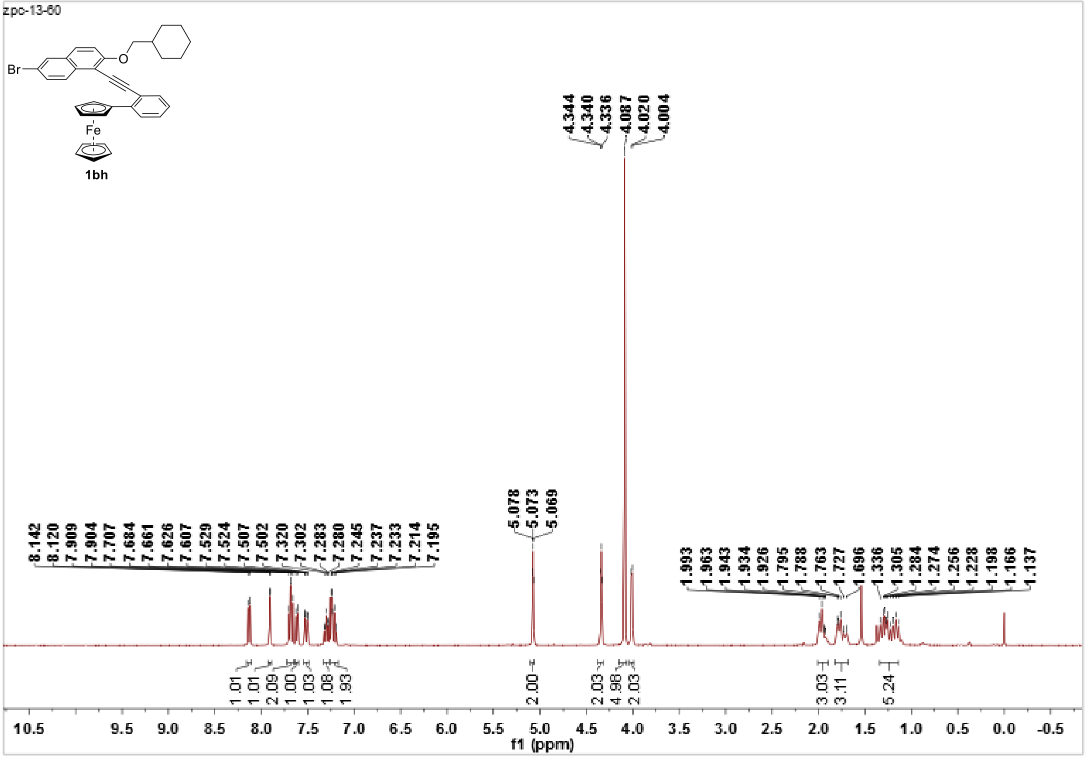

Supplementary Figure 85. ^{1}H NMR (400 MHz, $CDCl_3$) spectra for compound **1bh**

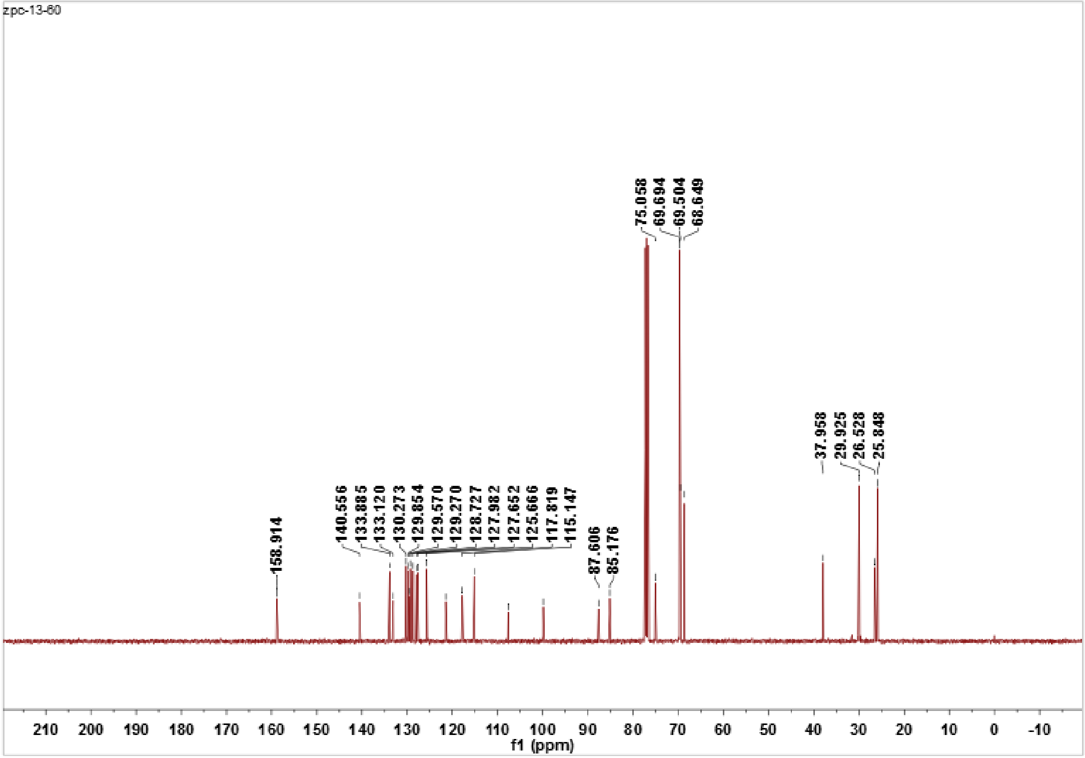

Supplementary Figure 86. ^{13}C NMR (400 MHz, $CDCl_3$) spectra for compound **1bh**

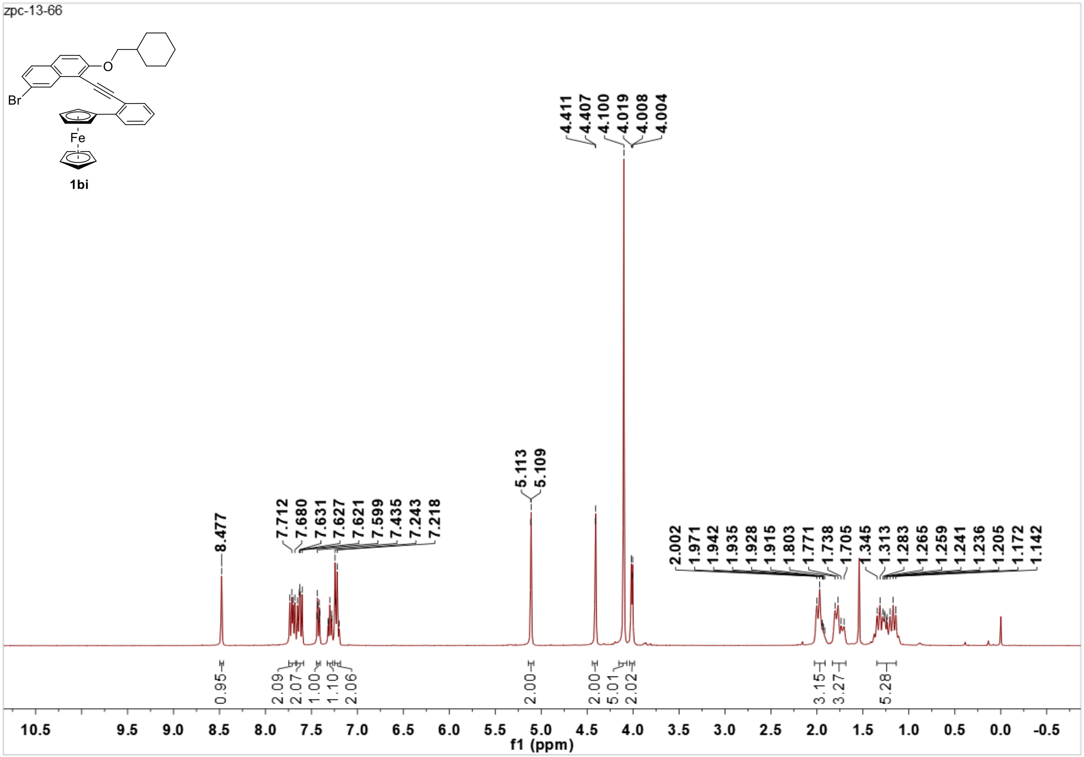

Supplementary Figure 87. ^{1}H NMR (400 MHz, $CDCl_3$) spectra for compound **1bi**

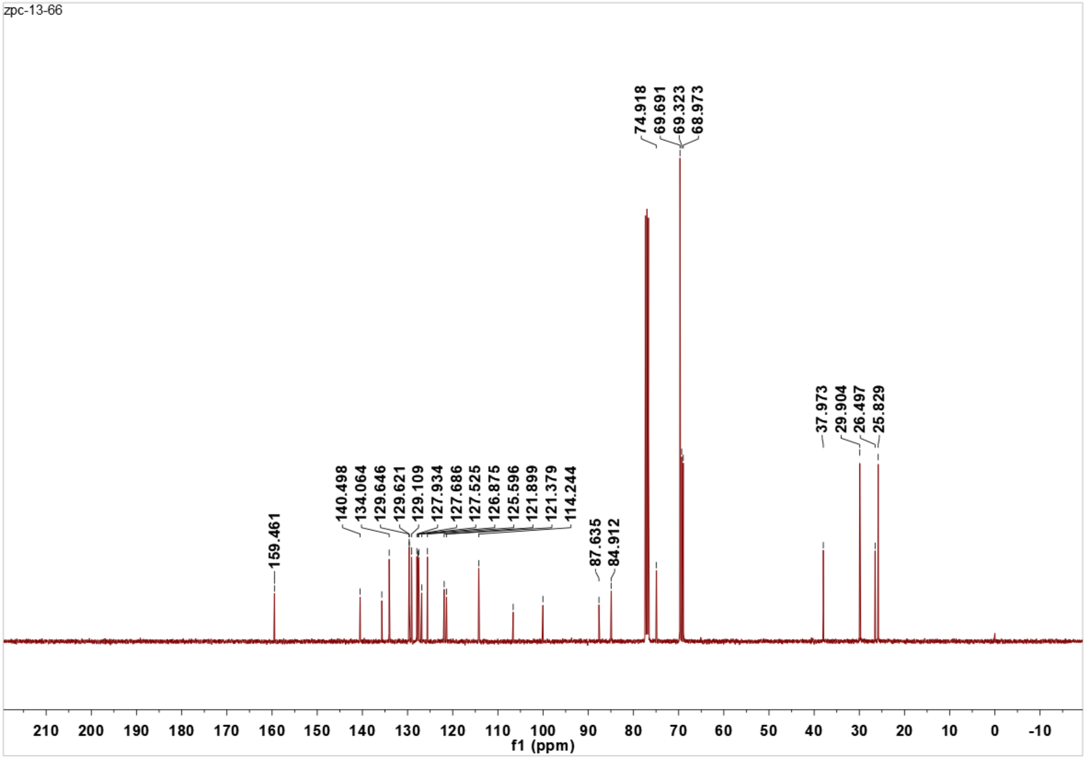

Supplementary Figure 88. ^{13}C NMR (400 MHz, $CDCl_3$) spectra for compound **1bi**

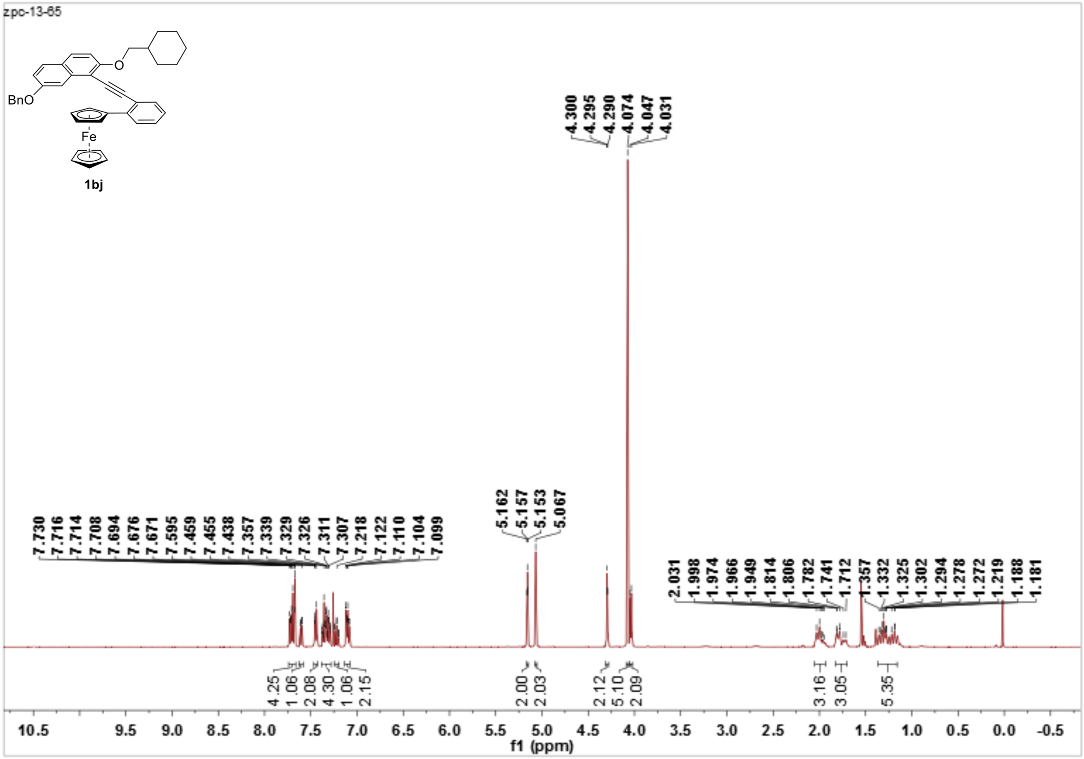

Supplementary Figure 89. ^{1}H NMR (400 MHz, $CDCl_3$) spectra for compound **1bj**

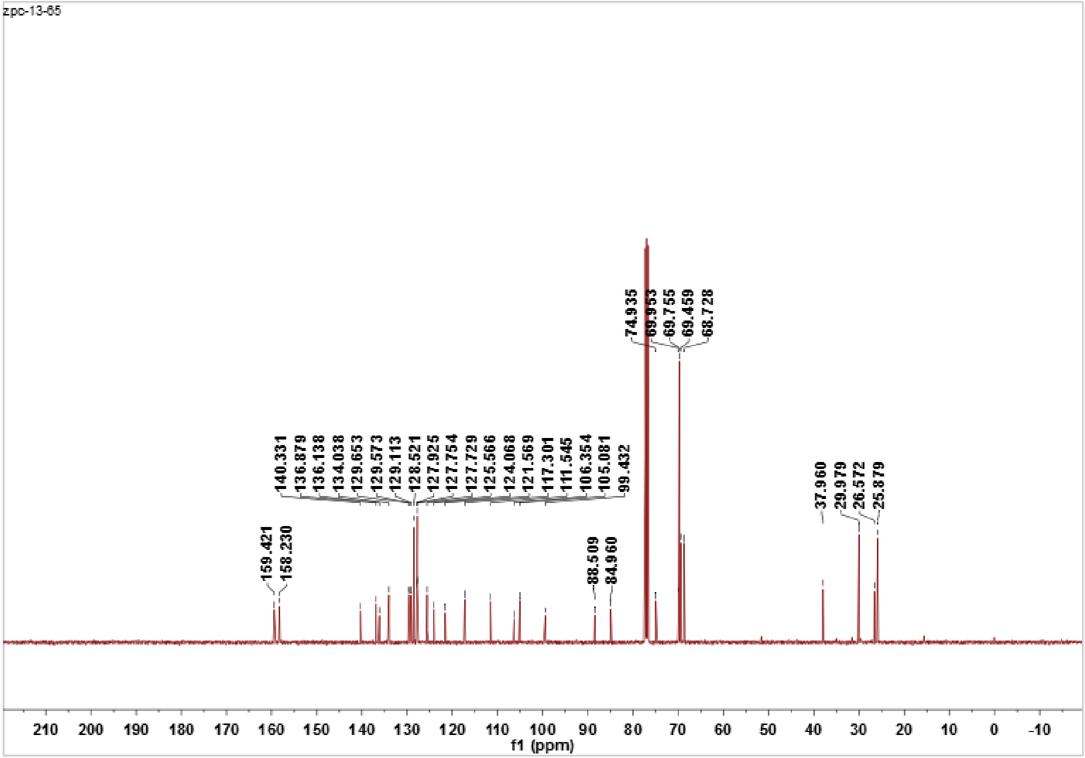

Supplementary Figure 90. ^{13}C NMR (400 MHz, $CDCl_3$) spectra for compound **1bj**

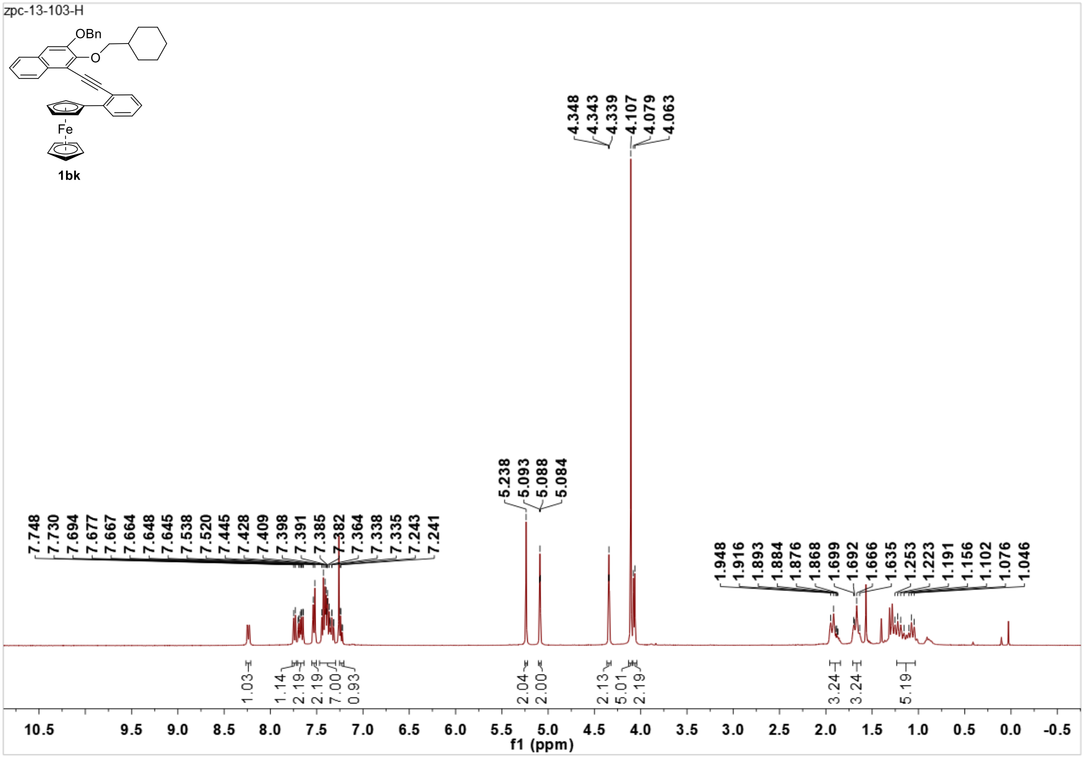

Supplementary Figure 91. ^{1}H NMR (400 MHz, $CDCl_3$) spectra for compound **1bk**

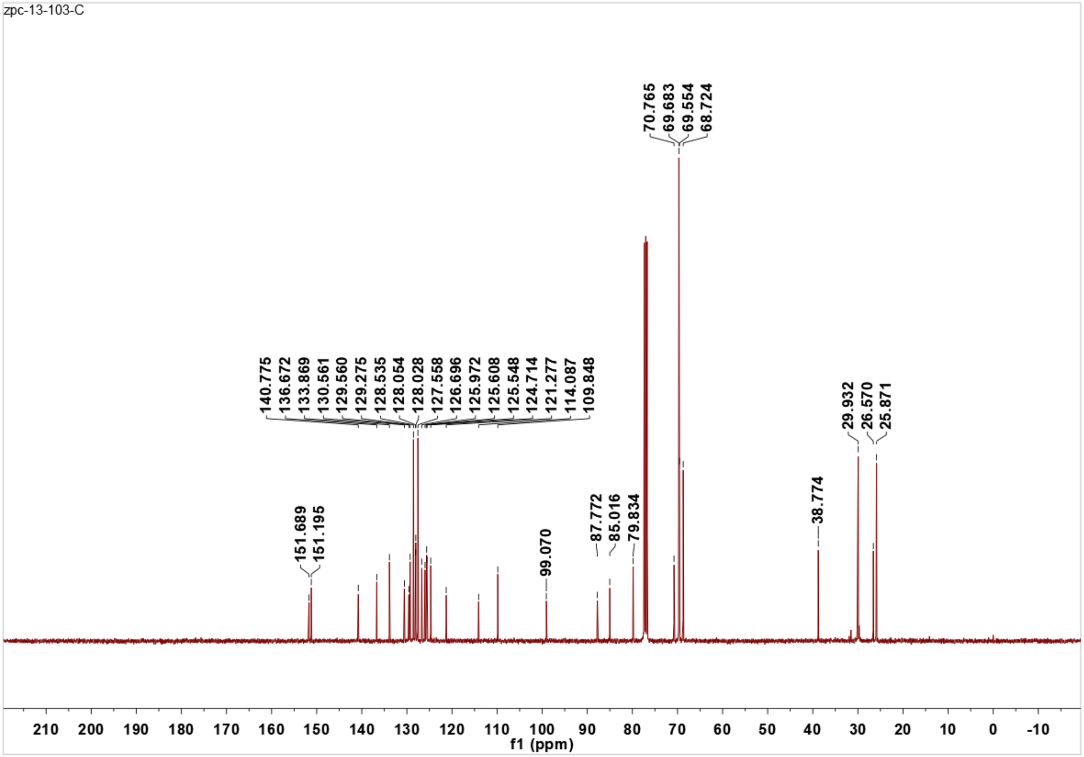

Supplementary Figure 92. ^{13}C NMR (400 MHz, $CDCl_3$) spectra for compound **1bk**

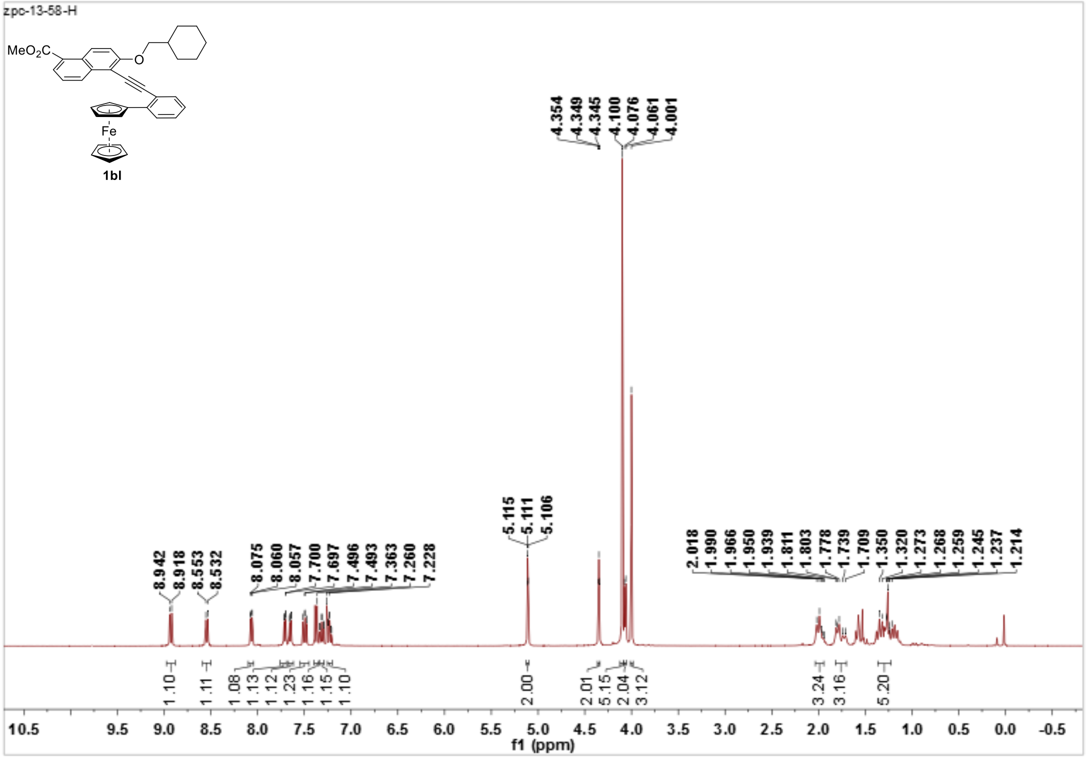

Supplementary Figure 93. ^{1}H NMR (400 MHz, $CDCl_3$) spectra for compound **1bl**

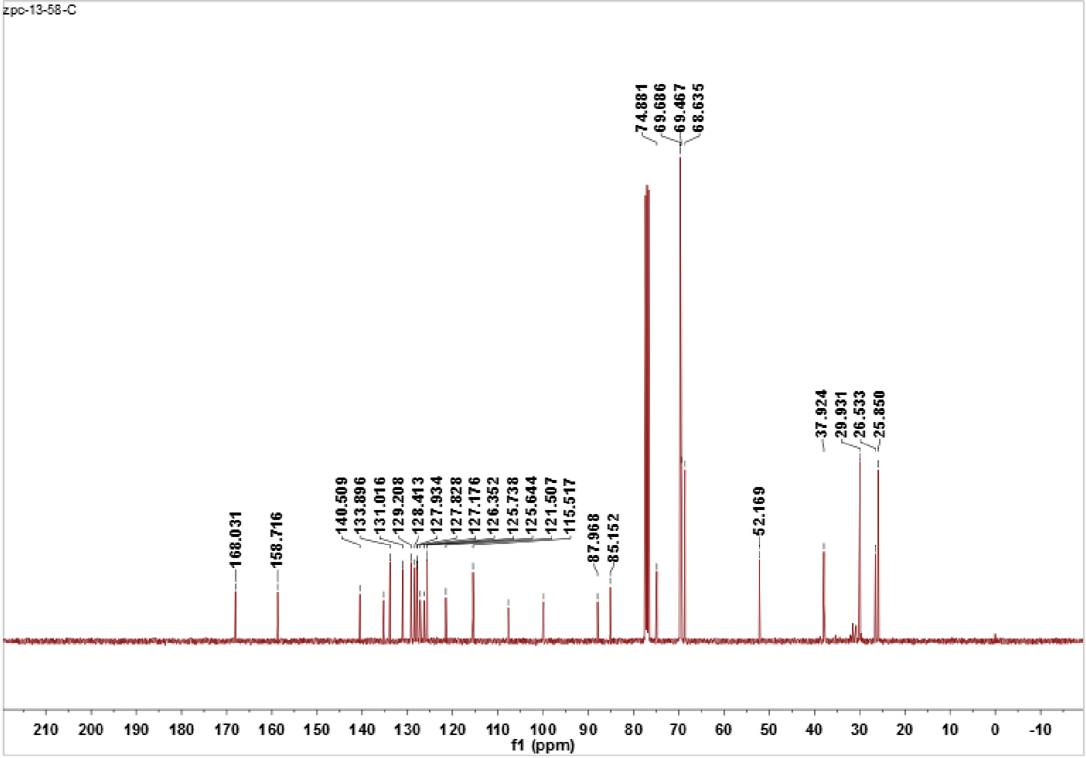

Supplementary Figure 94. ^{13}C NMR (400 MHz, $CDCl_3$) spectra for compound **1bl**

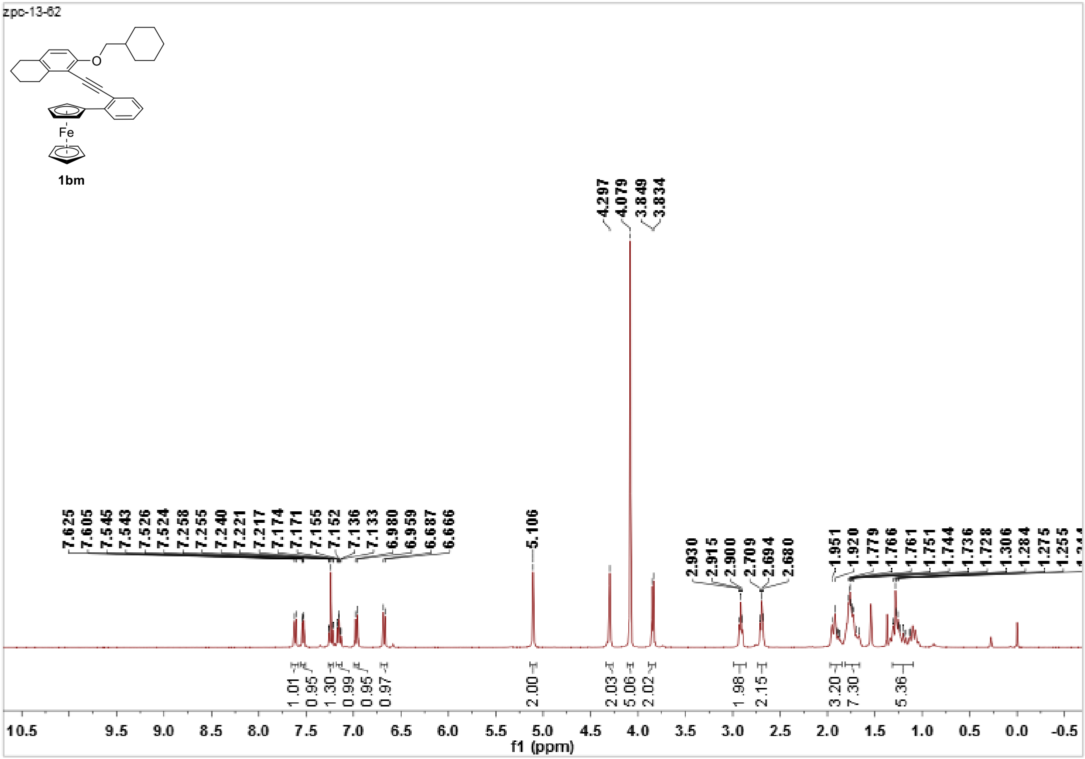

Supplementary Figure 95. ^{1}H NMR (400 MHz, $CDCl_3$) spectra for compound **1bm**

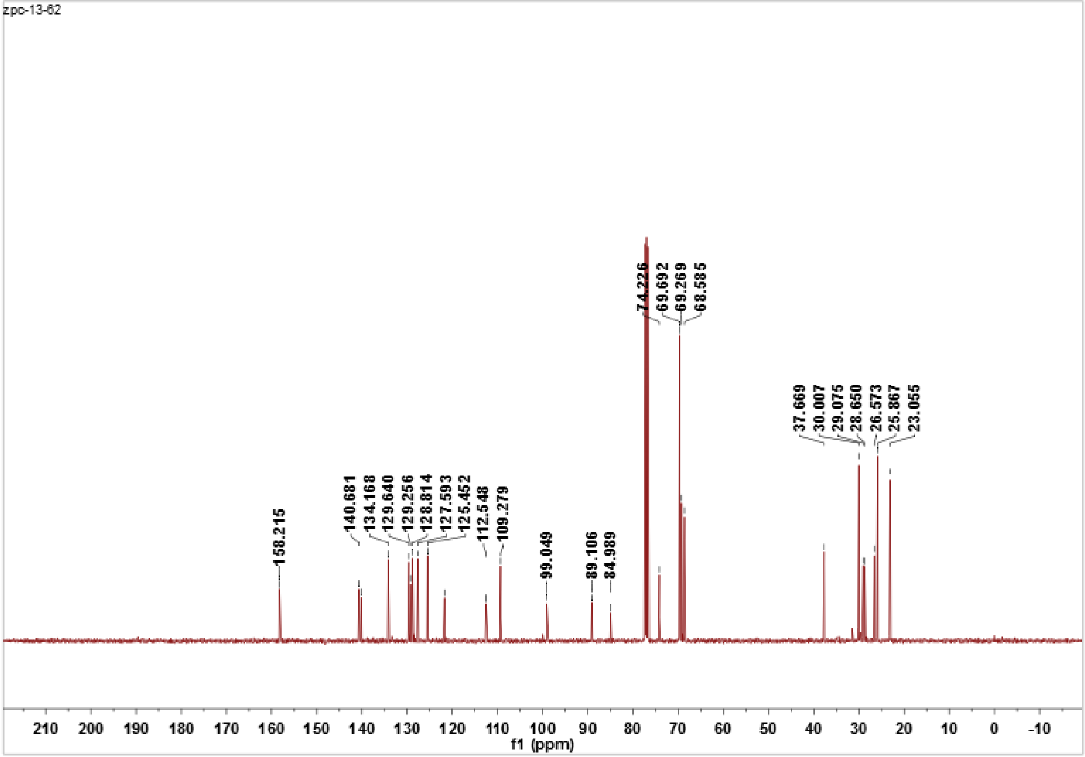

Supplementary Figure 96. ^{13}C NMR (400 MHz, $CDCl_3$) spectra for compound **1bm**

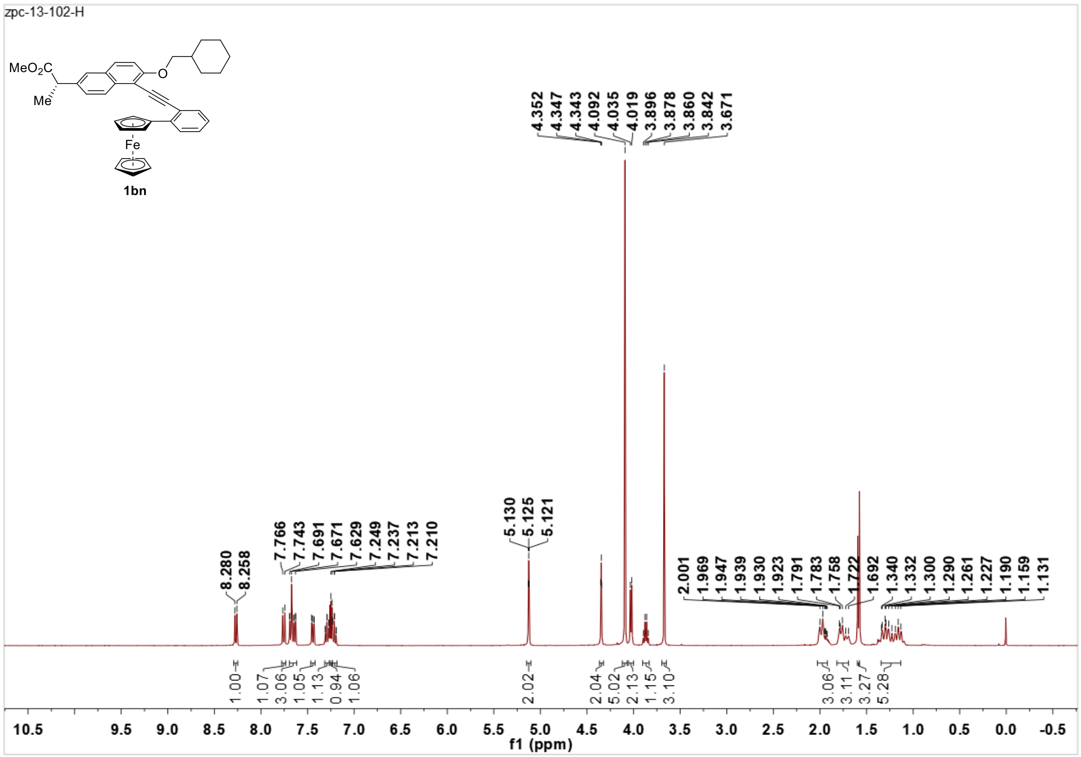

Supplementary Figure 97. ^{1}H NMR (400 MHz, $CDCl_3$) spectra for compound **1bn**

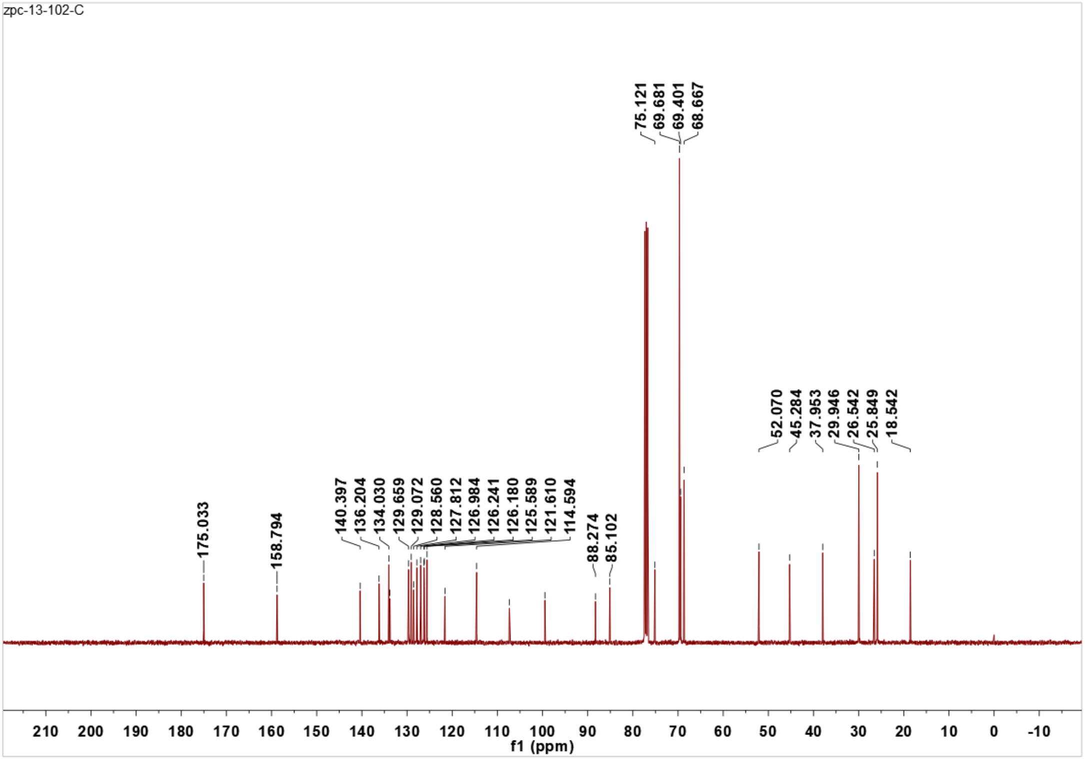

Supplementary Figure 98. ^{13}C NMR (400 MHz, $CDCl_3$) spectra for compound **1bn**

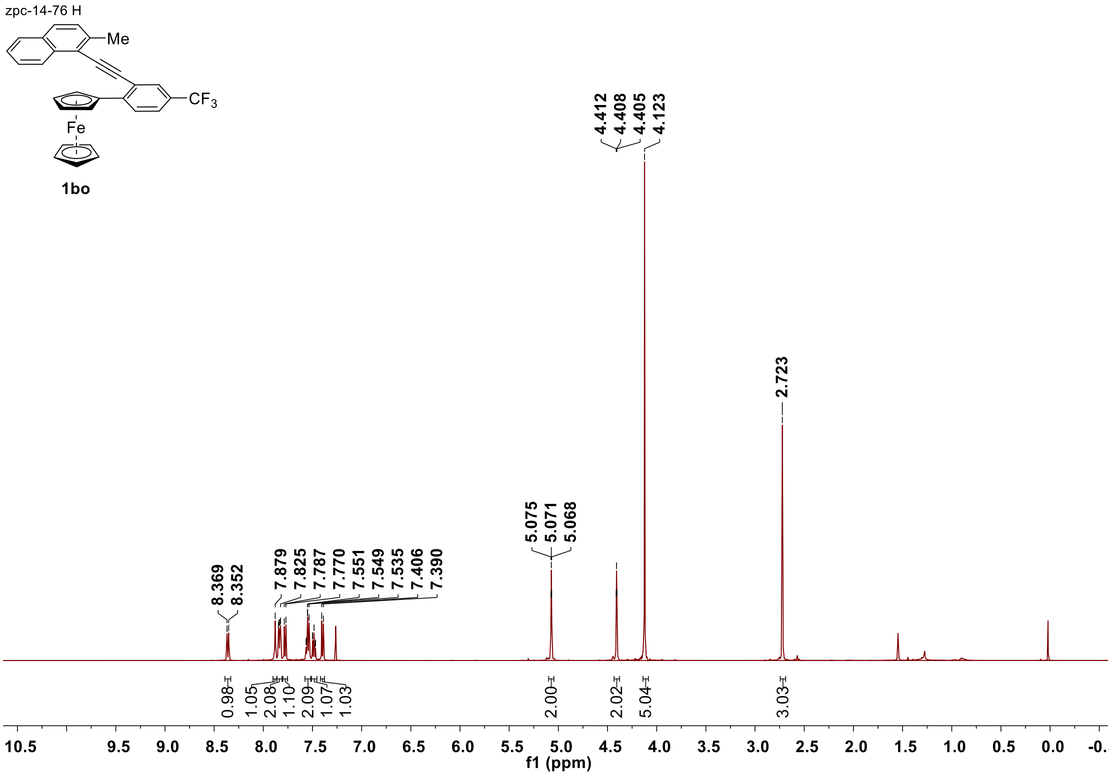

Supplementary Figure 99. ^{1}H NMR (400 MHz, $CDCl_3$) spectra for compound **1bo**

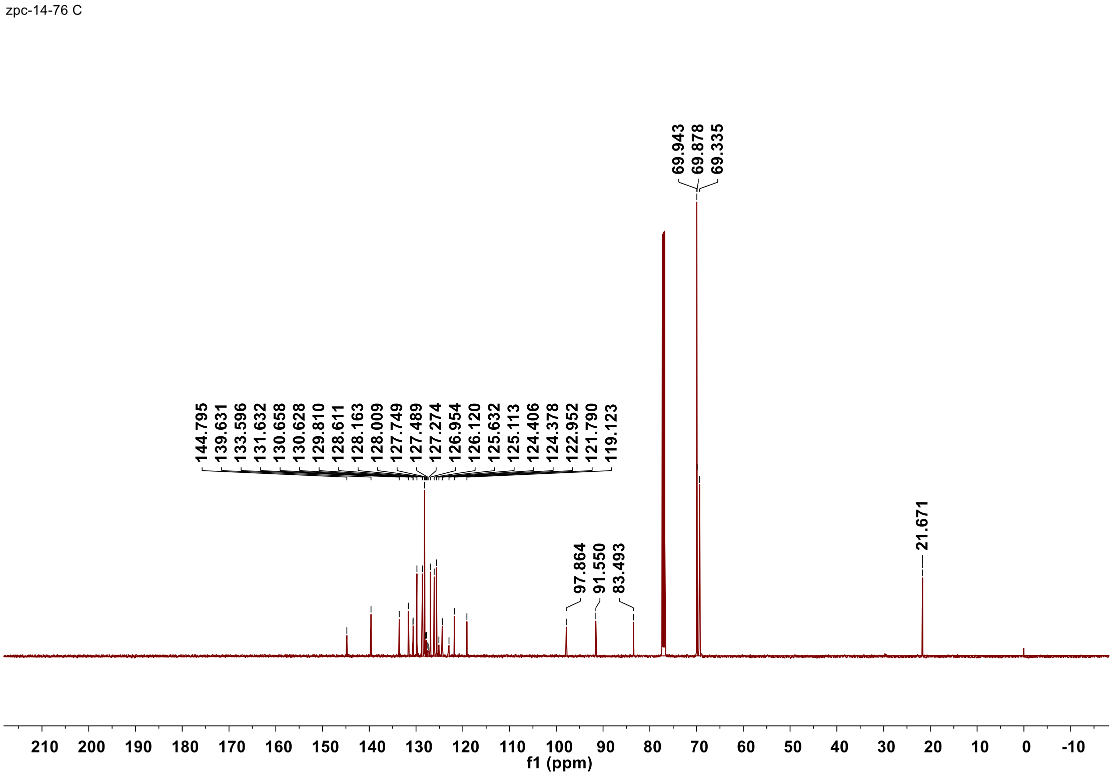

Supplementary Figure 100. ^{13}C NMR (400 MHz, $CDCl_3$) spectra for compound **1bo**

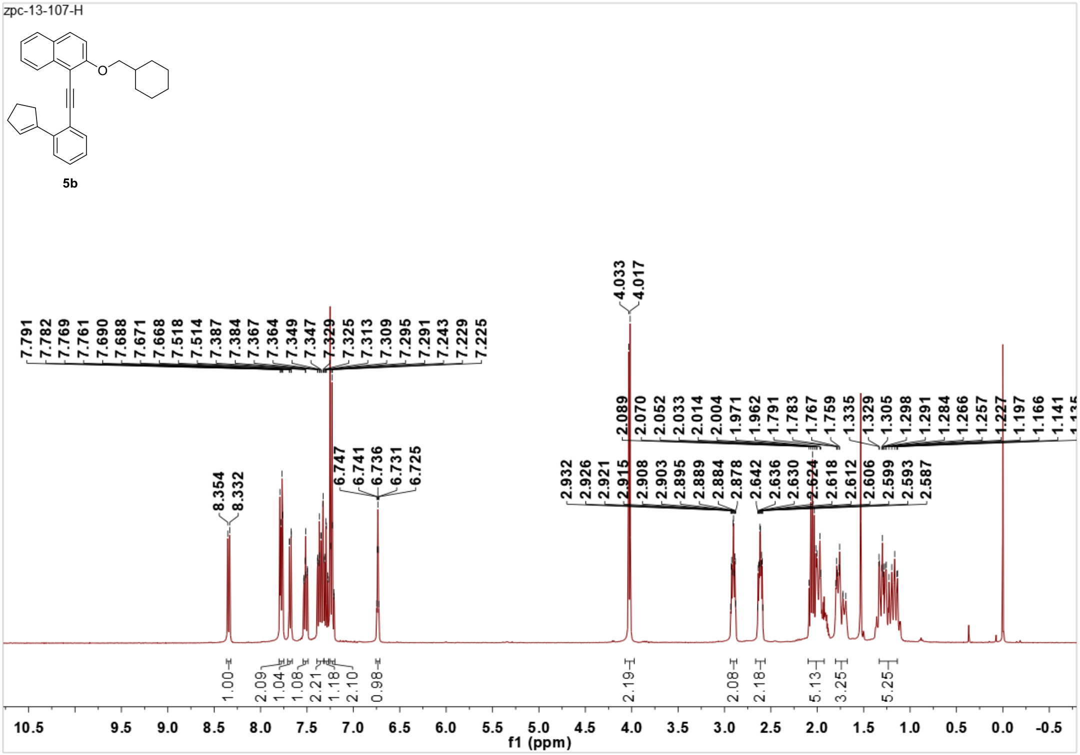

Supplementary Figure 101. ^{1}H NMR (400 MHz, $CDCl_3$) spectra for compound **5b**

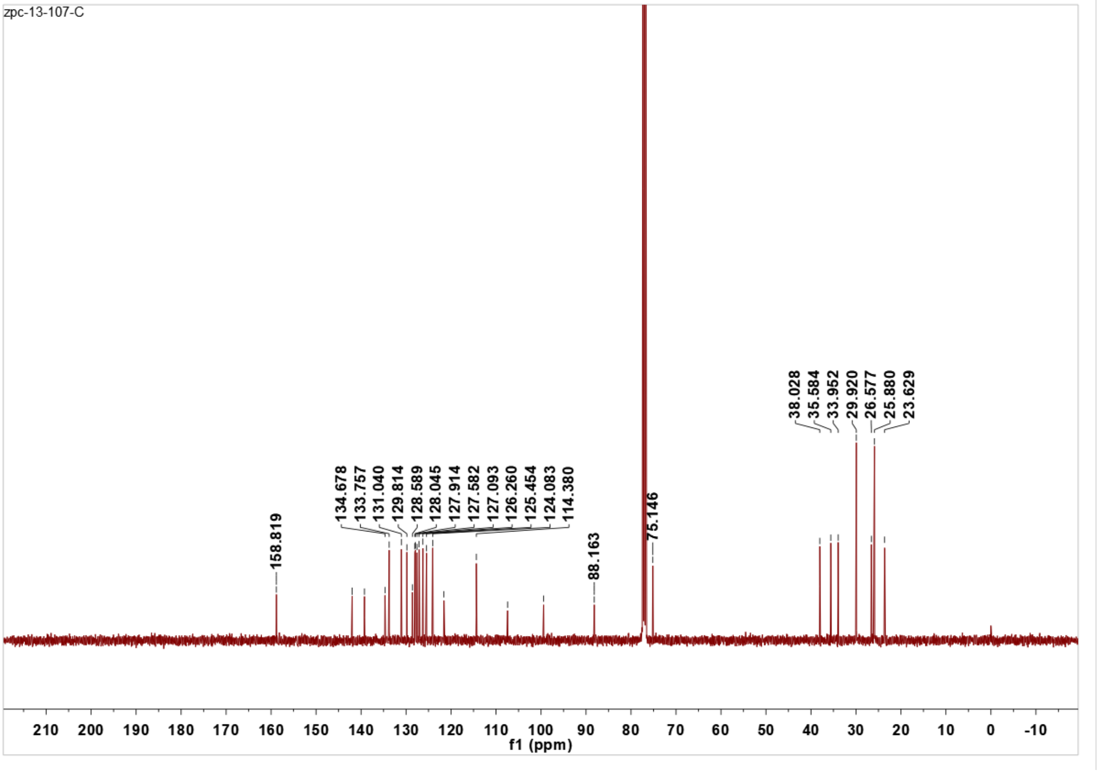

Supplementary Figure 102. ^{13}C NMR (400 MHz, $CDCl_3$) spectra for compound **5b**

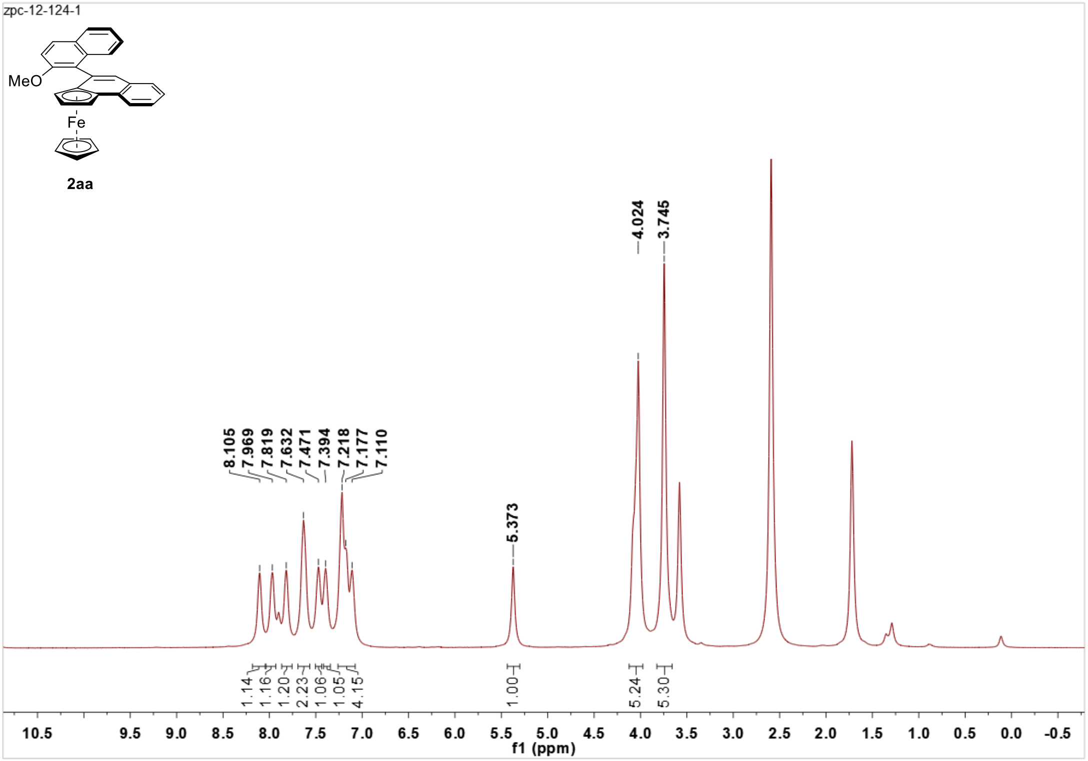

Supplementary Figure 103. ^{1}H NMR (500 MHz, THF-d_8) spectra for compound **2aa**

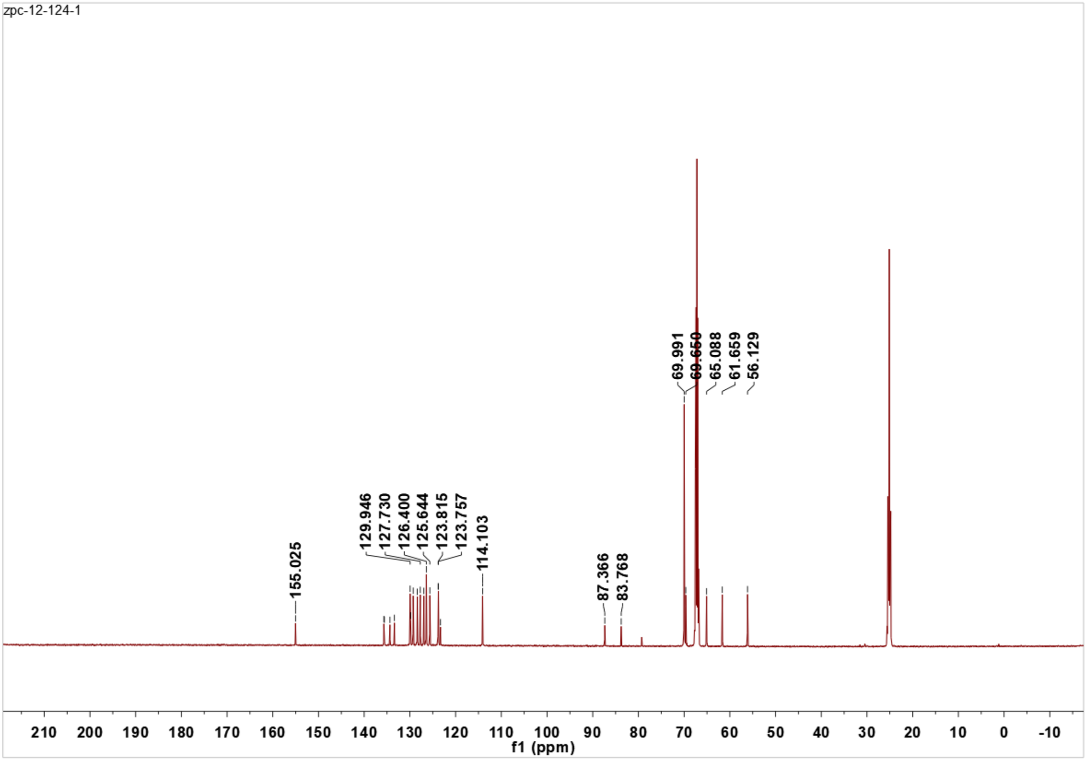

Supplementary Figure 104. ^{13}C NMR (500 MHz, THF-d_8) spectra for compound **2aa**

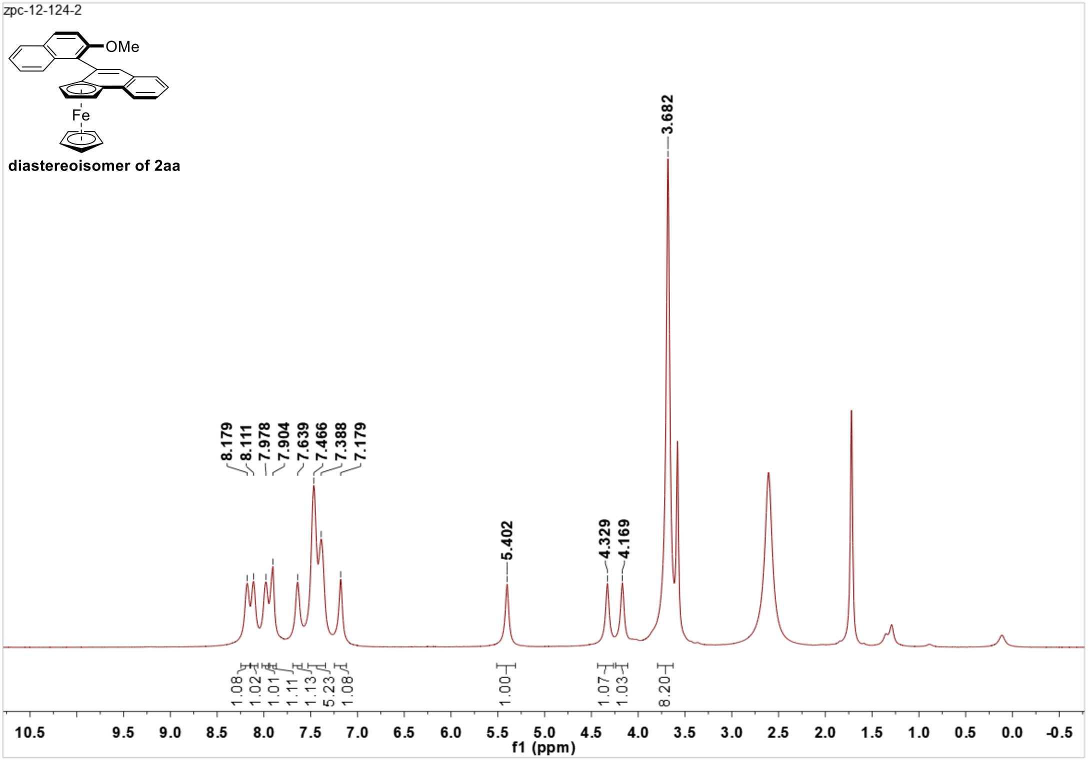

Supplementary Figure 105. ^{1}H NMR (500 MHz, THF-d_8) spectra for compound diastereoisomer **2aa**

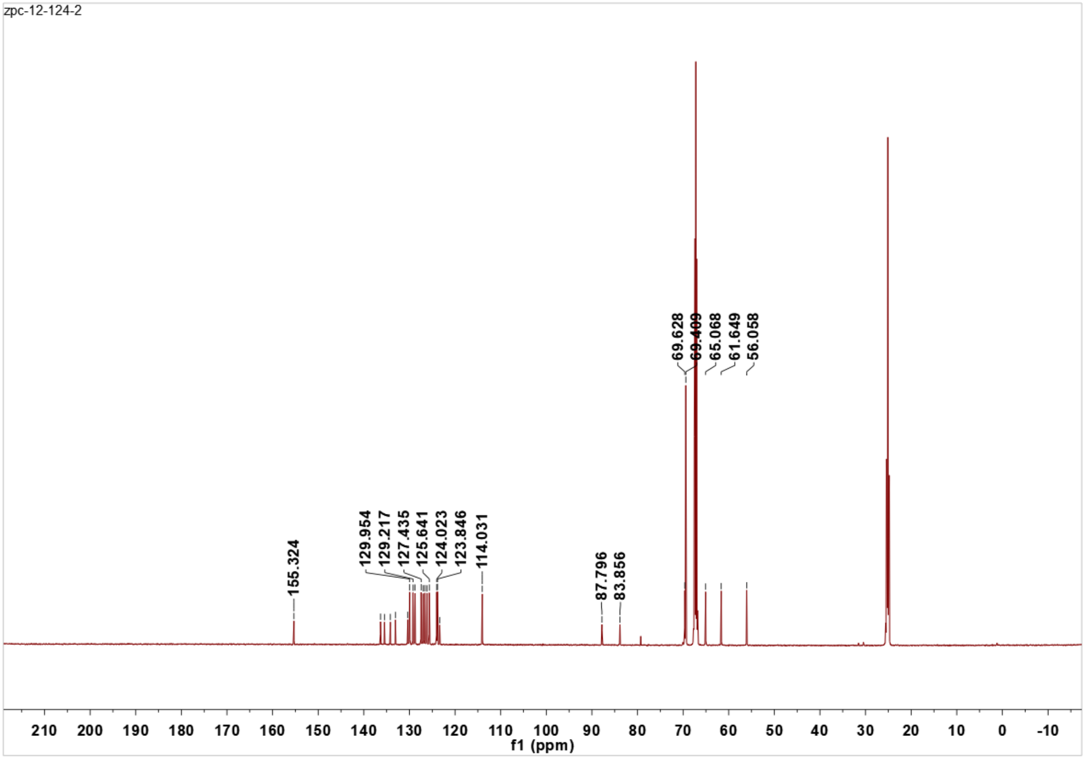

Supplementary Figure 106. ^{13}C NMR (500 MHz, THF-d_8) spectra for compound diastereoisomer **2aa**

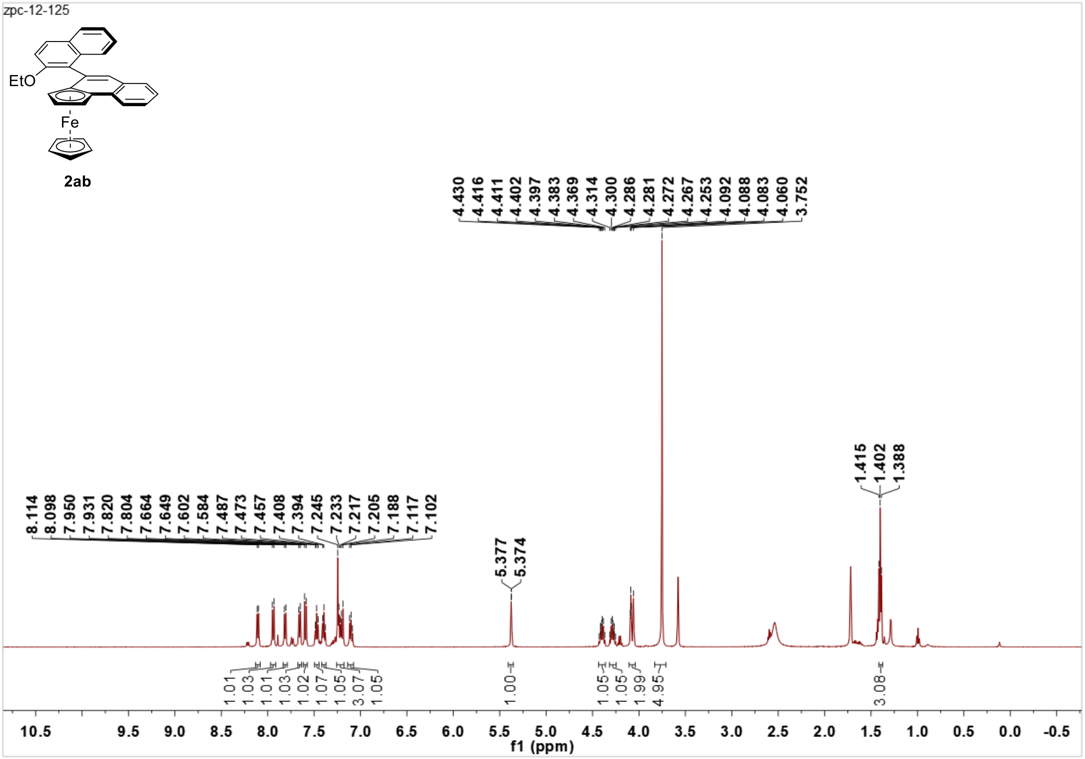

Supplementary Figure 107. ^{1}H NMR (500 MHz, THF-d_8) spectra for compound **2ab**

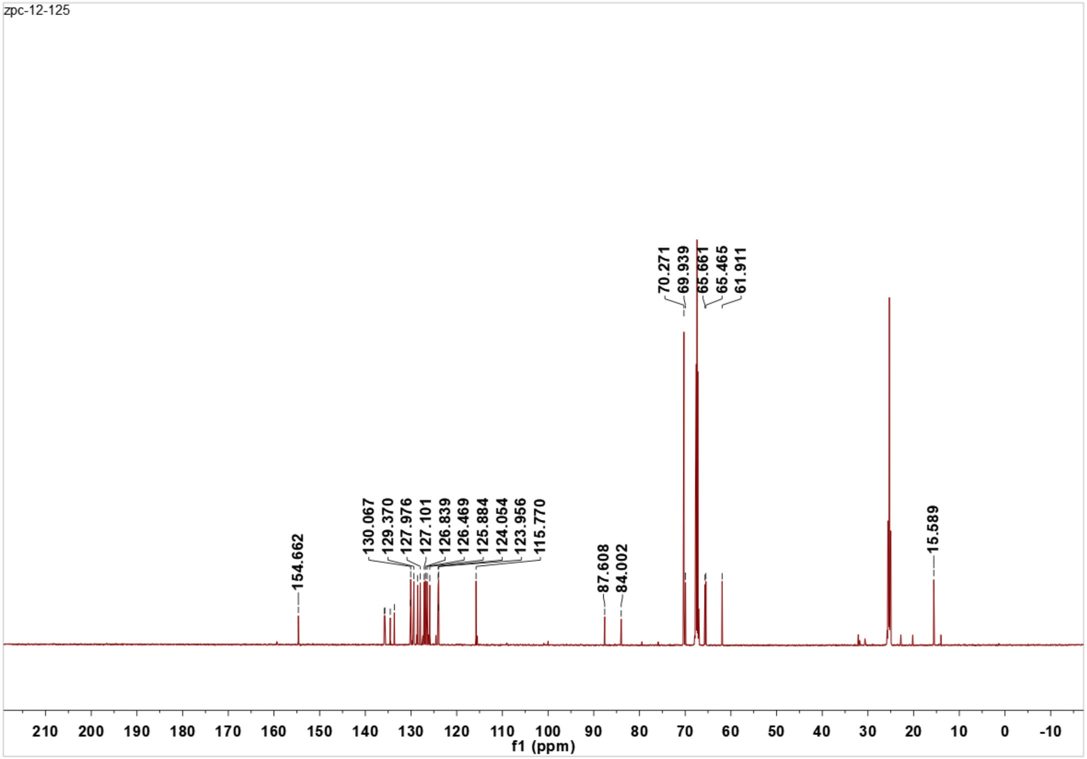

Supplementary Figure 108. ^{13}C NMR (500 MHz, THF-d_8) spectra for compound **2ab**

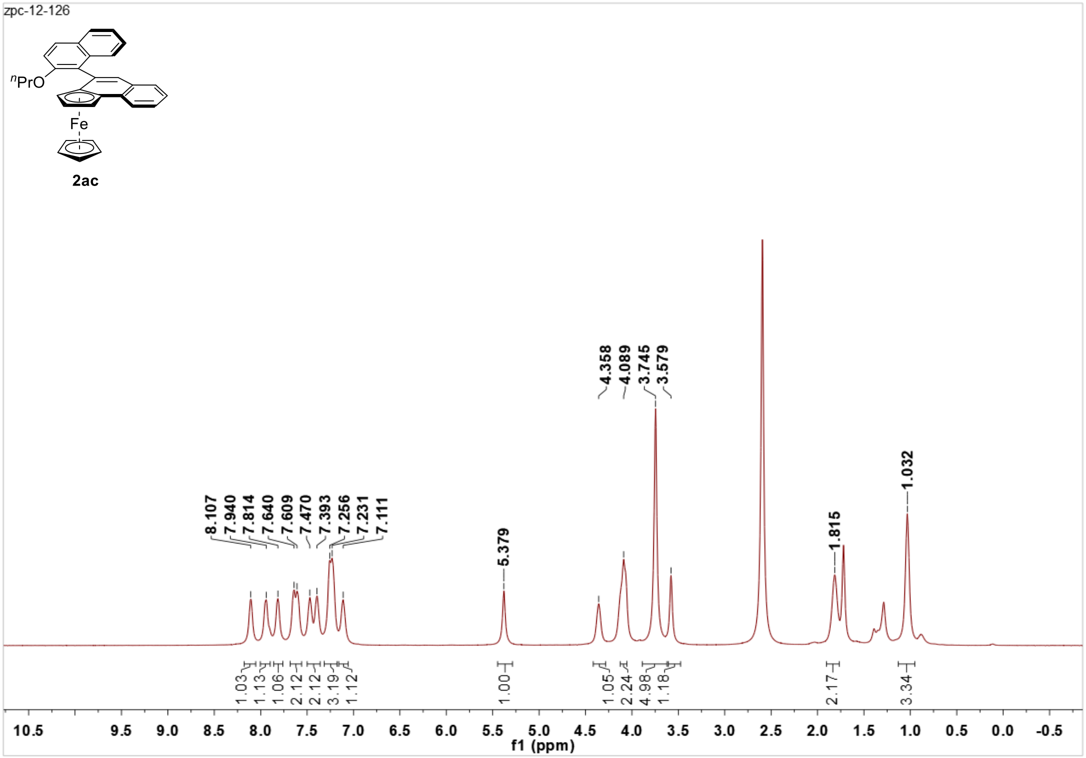

Supplementary Figure 109. ^{1}H NMR (500 MHz, THF-d_8) spectra for compound **2ac**

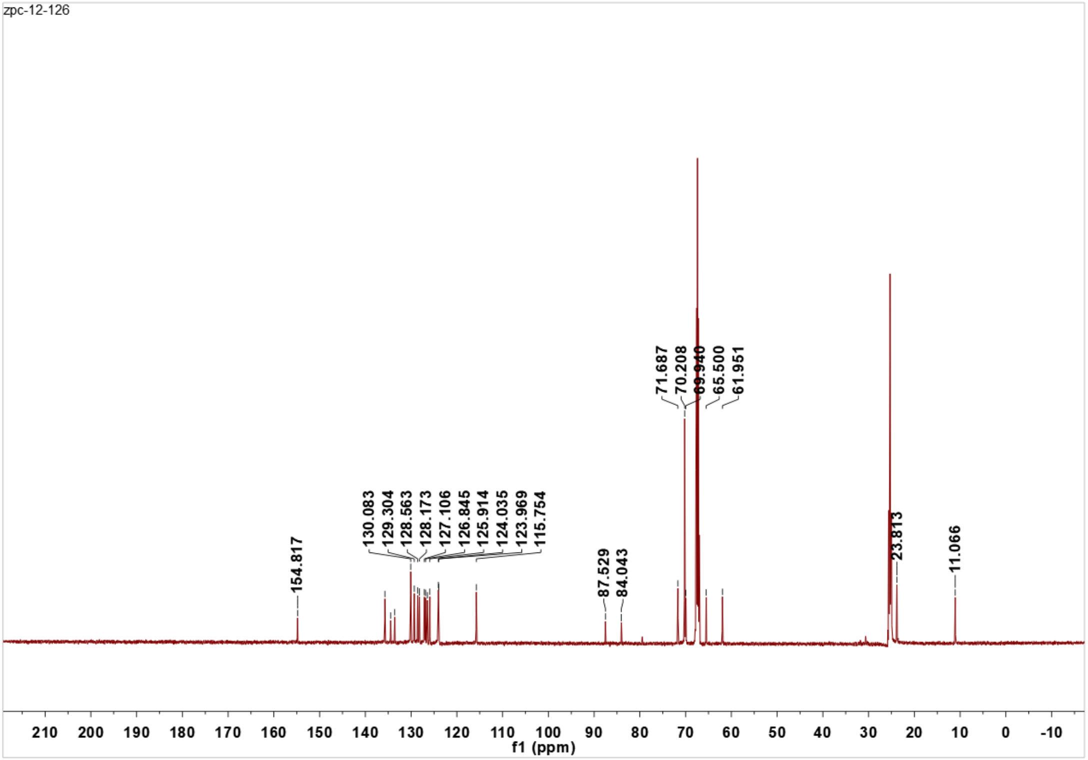

Supplementary Figure 110. ^{13}C NMR (500 MHz, THF-d_8) spectra for compound **2ac**

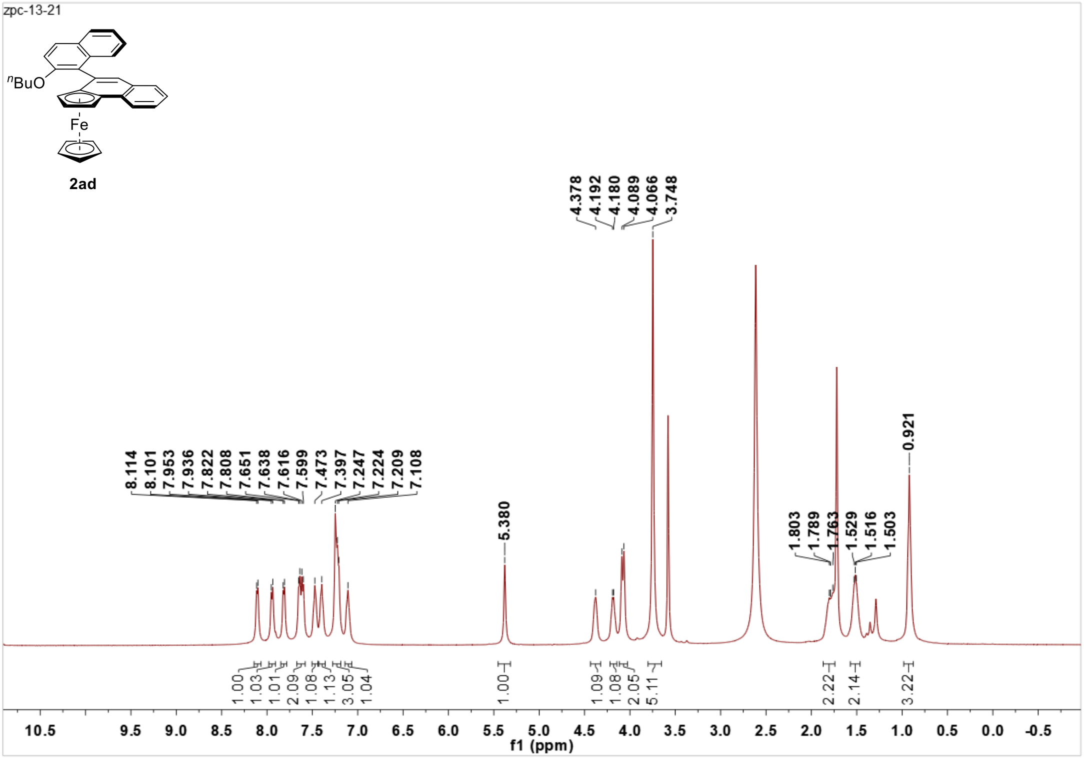

Supplementary Figure 111. ^{1}H NMR (500 MHz, THF-d_8) spectra for compound **2ad**

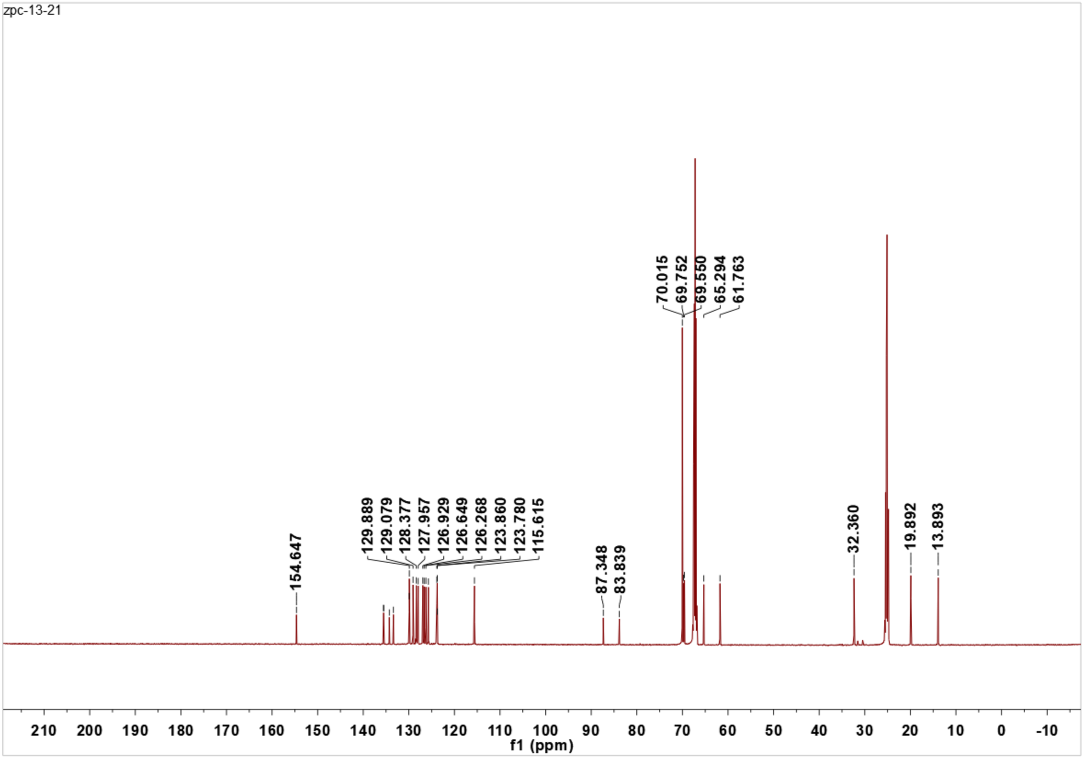

Supplementary Figure 112. ^{13}C NMR (500 MHz, THF-d_8) spectra for compound **2ad**

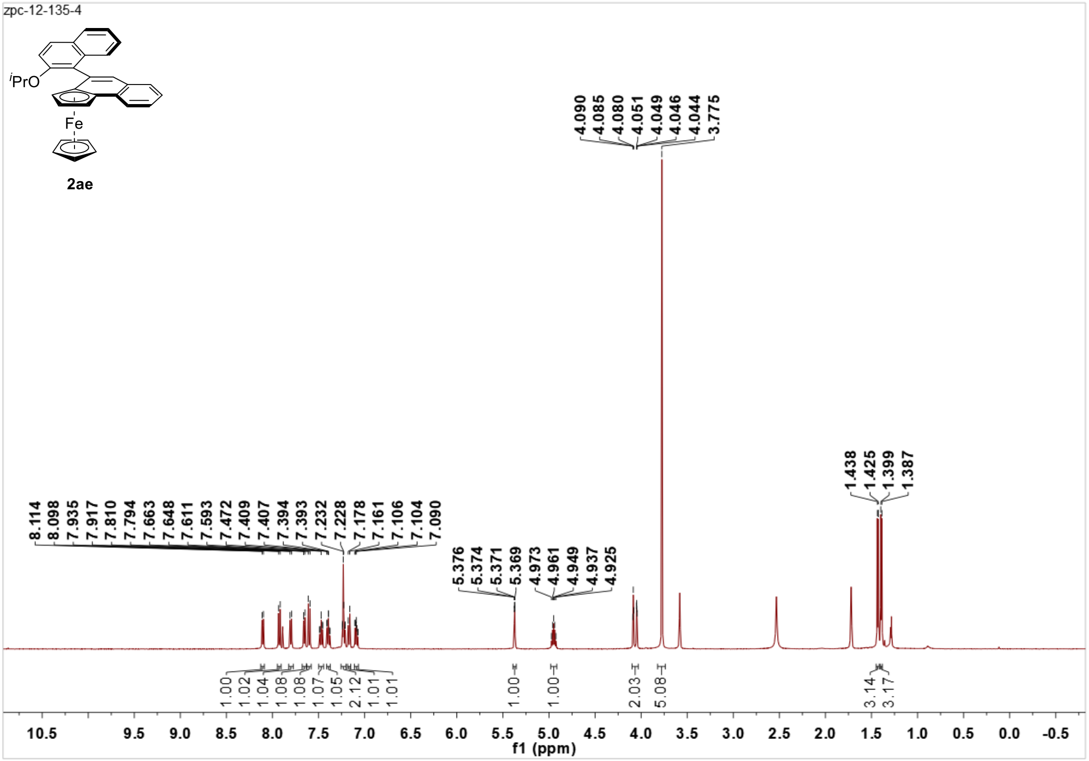

Supplementary Figure 113. ^{1}H NMR (500 MHz, THF-d_8) spectra for compound **2ae**

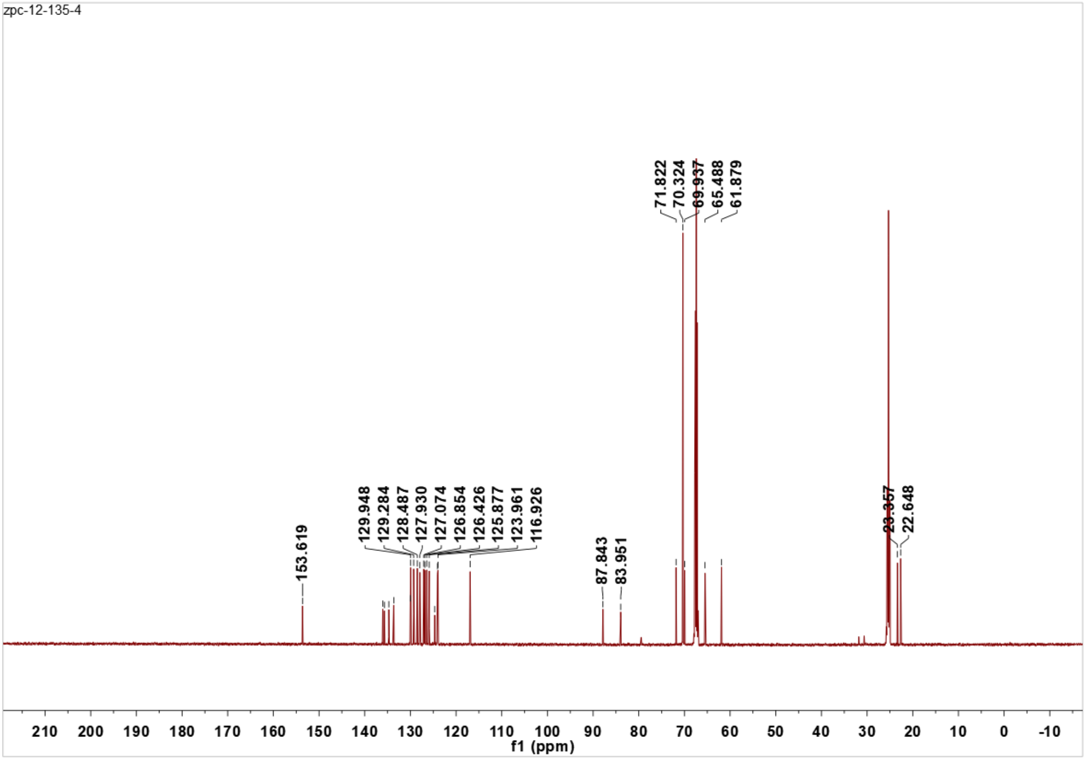

Supplementary Figure 114. ^{13}C NMR (500 MHz, THF-d_8) spectra for compound **2ae**

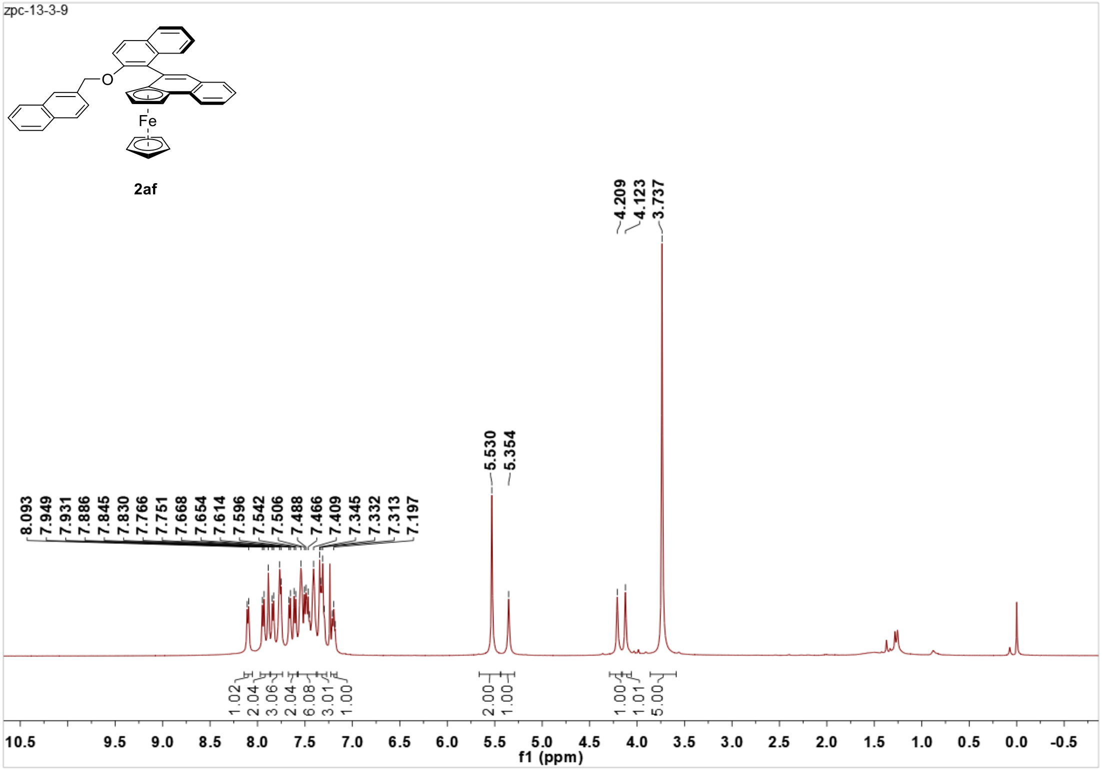

Supplementary Figure 115. ^{1}H NMR (500 MHz, $CDCl_3$) spectra for compound **2af**

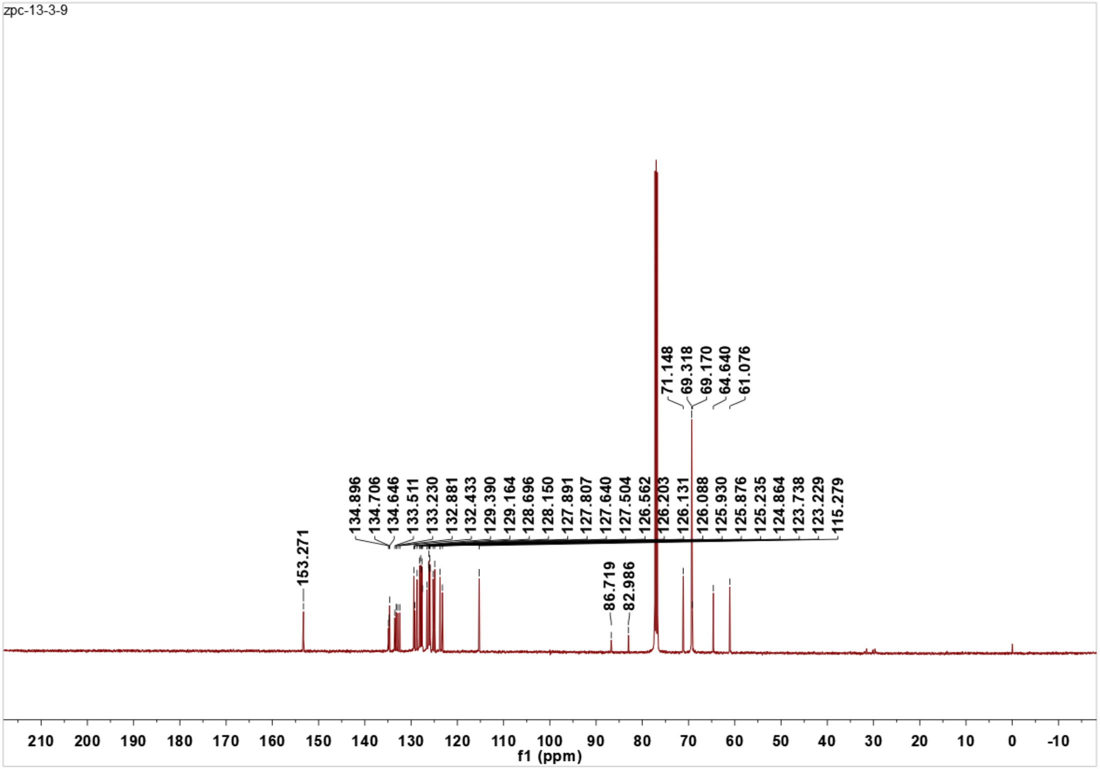

Supplementary Figure 116. ^{13}C NMR (500 MHz, $CDCl_3$) spectra for compound **2af**

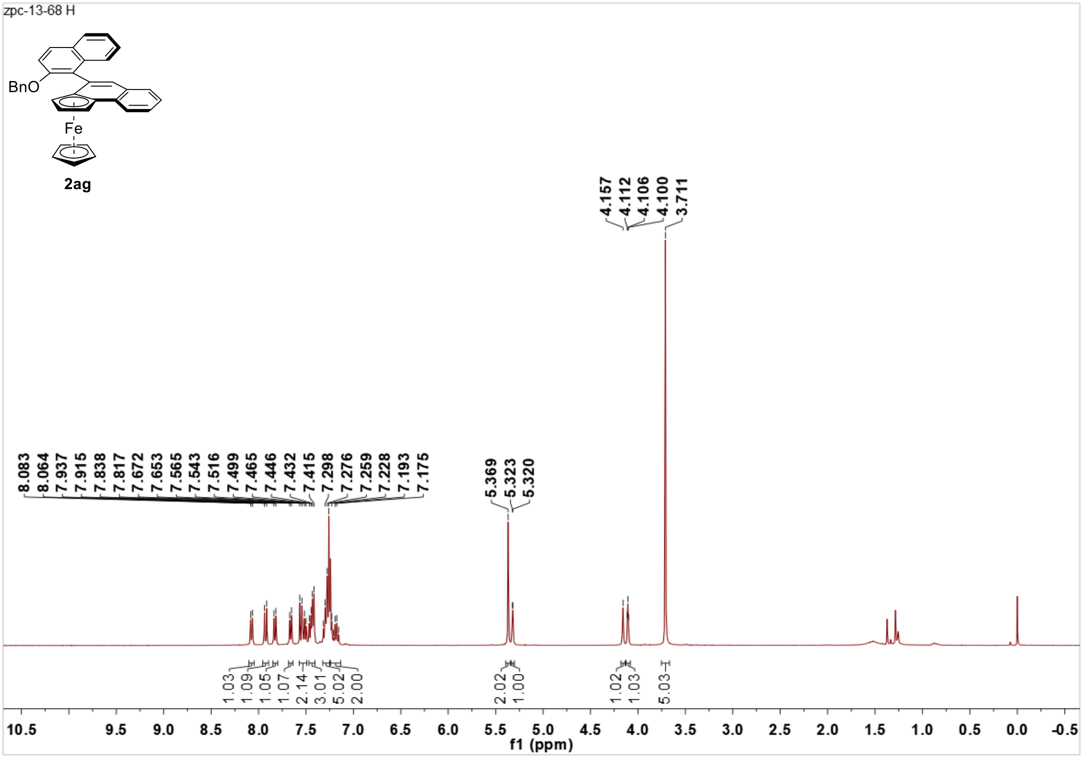

Supplementary Figure 117. ^{1}H NMR (400 MHz, $CDCl_3$) spectra for compound **2ag**

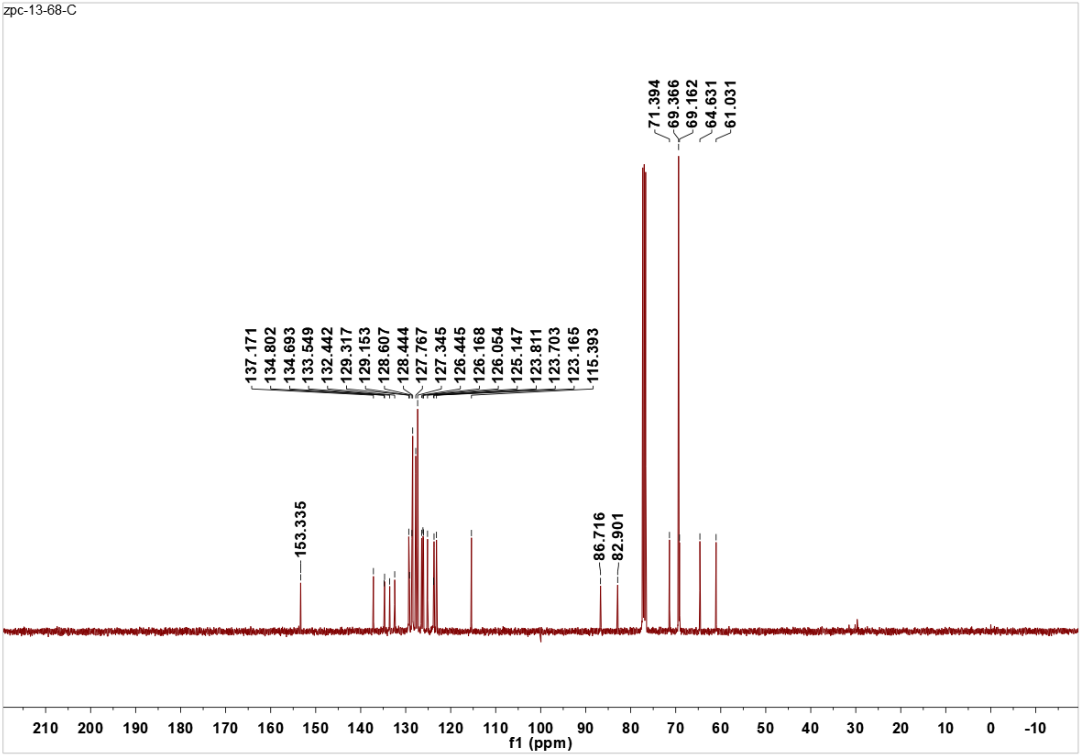

Supplementary Figure 118. ^{13}C NMR (400 MHz, $CDCl_3$) spectra for compound **2ag**

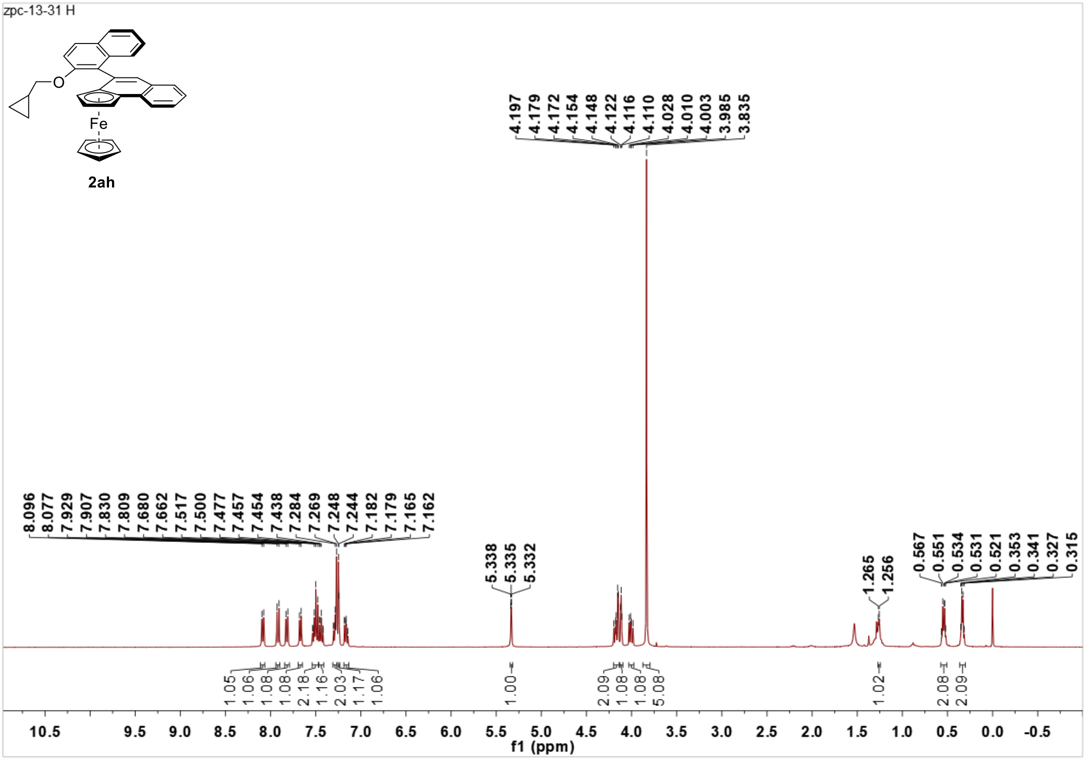

Supplementary Figure 119. ^{1}H NMR (400 MHz, $CDCl_3$) spectra for compound **2ah**

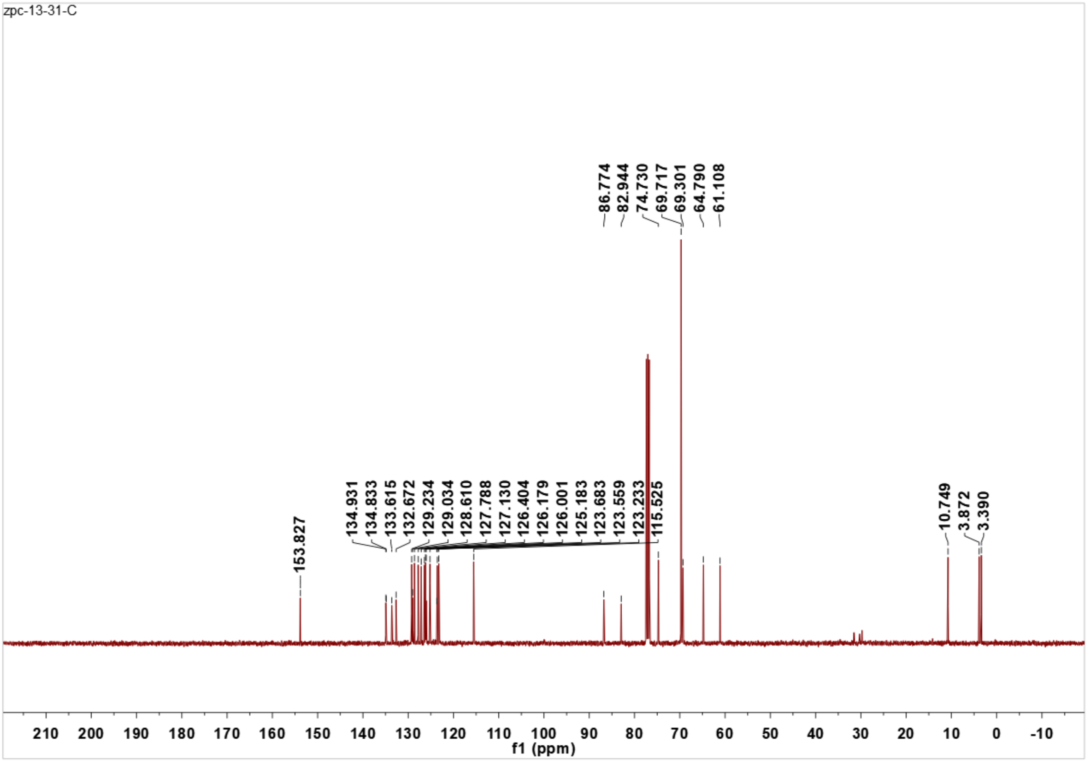

Supplementary Figure 120. ^{13}C NMR (400 MHz, $CDCl_3$) spectra for compound **2ah**

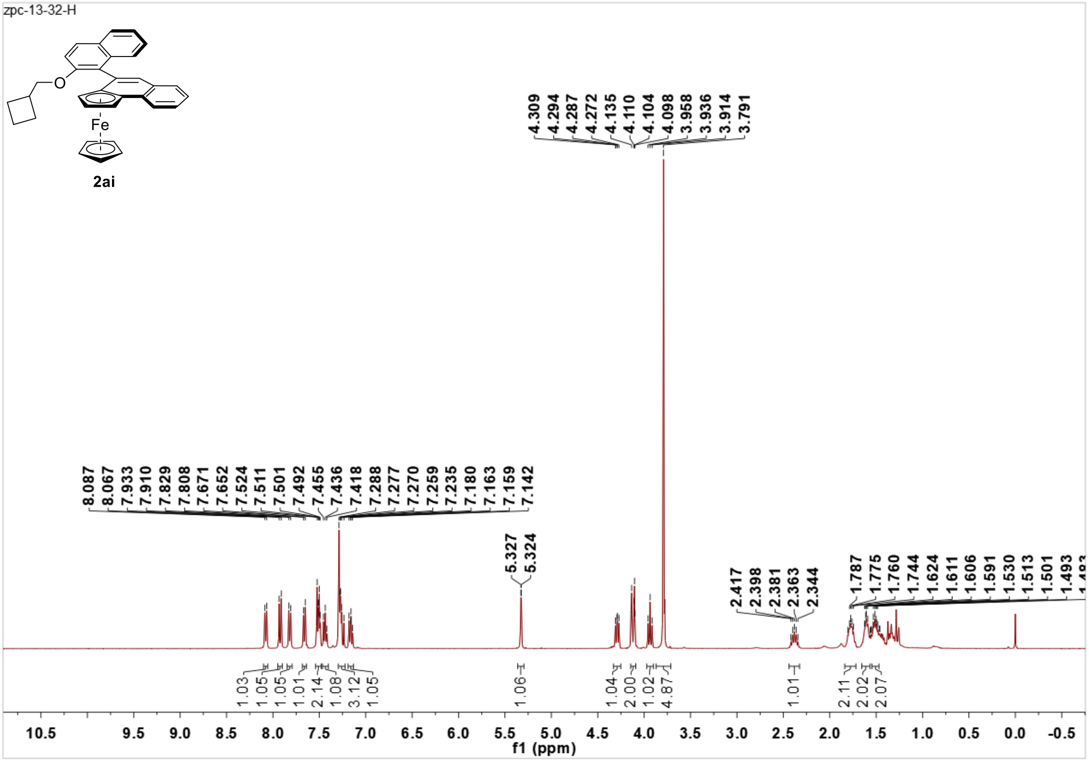

Supplementary Figure 121. ^{1}H NMR (400 MHz, $CDCl_3$) spectra for compound **2ai**

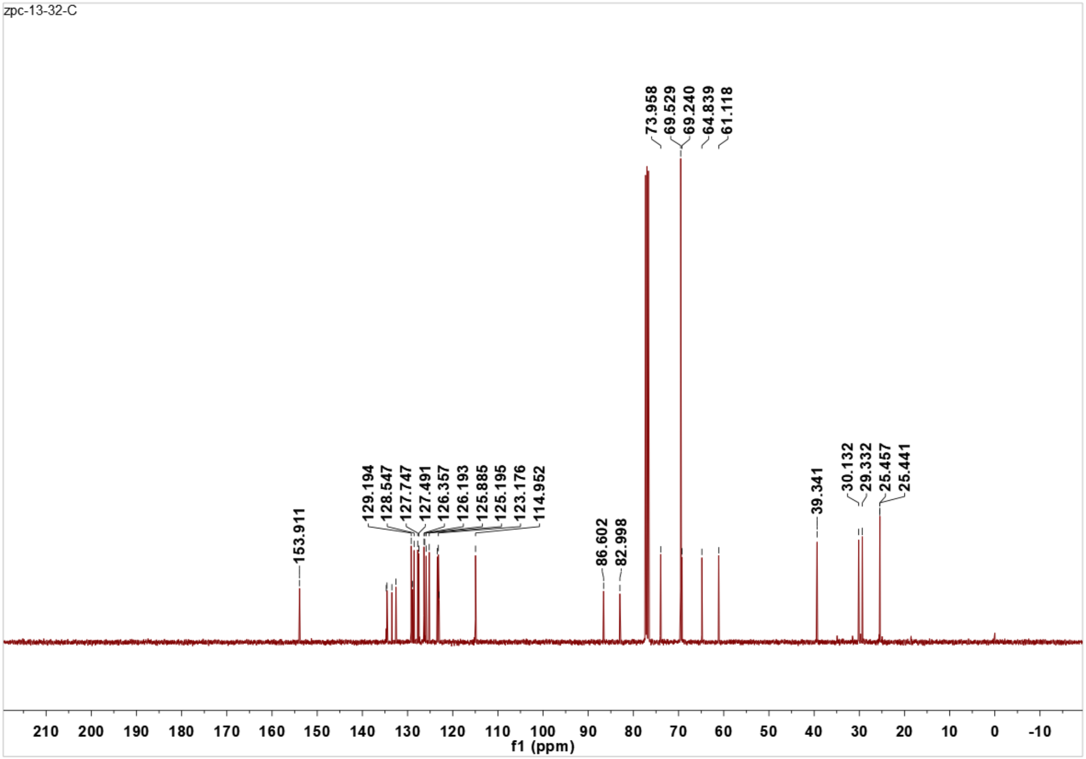

Supplementary Figure 122. ^{13}C NMR (400 MHz, $CDCl_3$) spectra for compound **2ai**

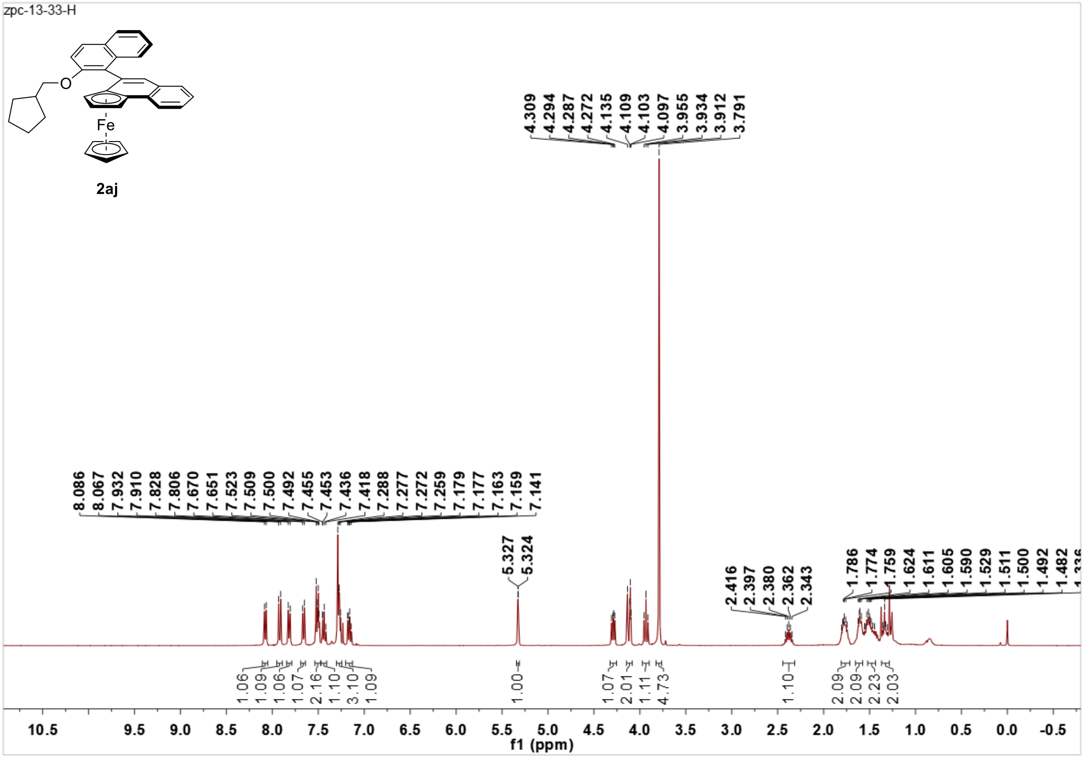

Supplementary Figure 123. ^{1}H NMR (400 MHz, $CDCl_3$) spectra for compound **2aj**

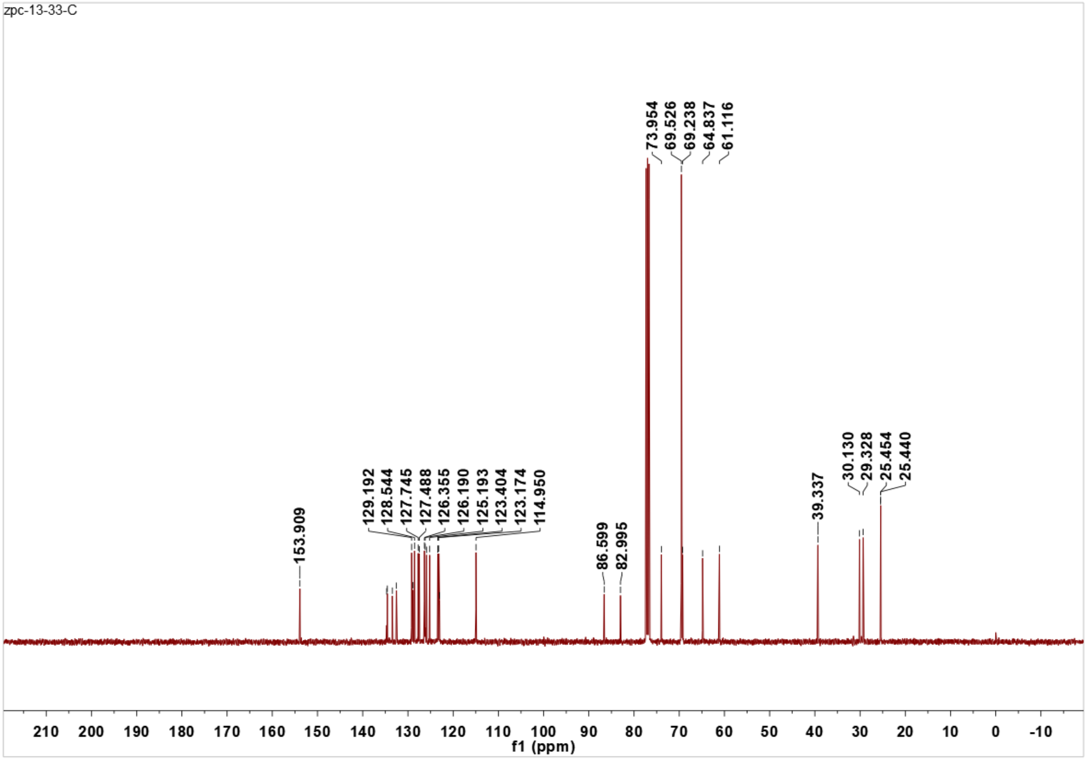

Supplementary Figure 124. ^{13}C NMR (400 MHz, $CDCl_3$) spectra for compound **2aj**

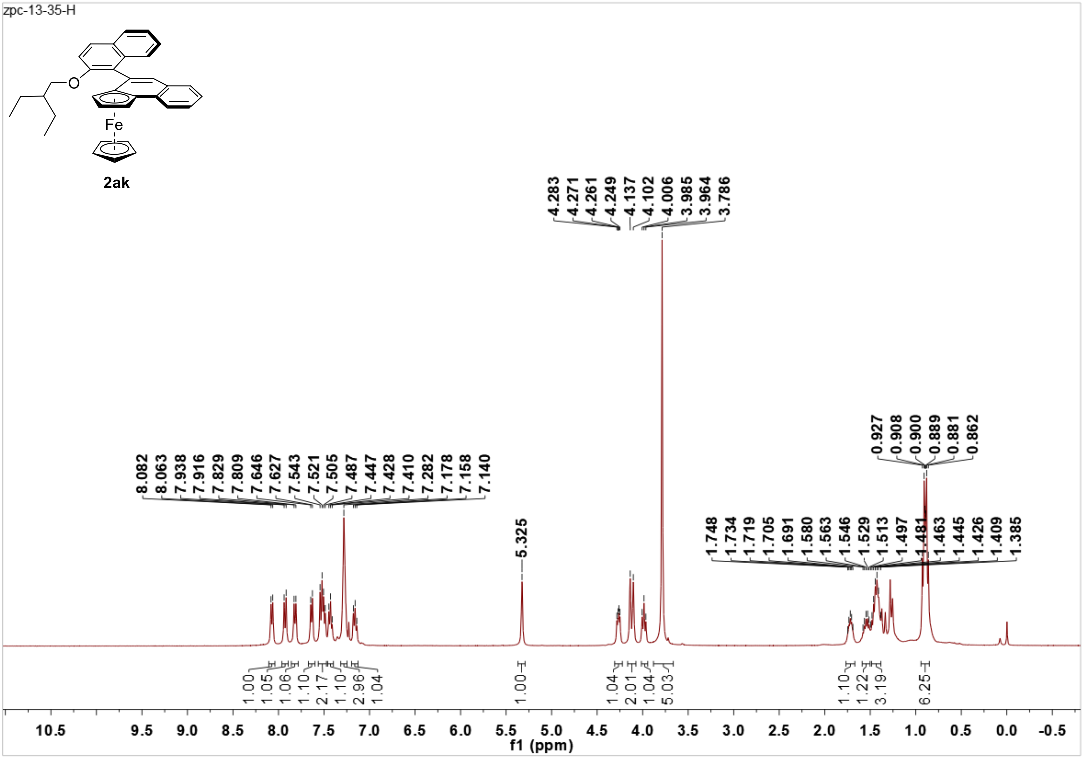

Supplementary Figure 125. ^{1}H NMR (400 MHz, $CDCl_3$) spectra for compound **2ak**

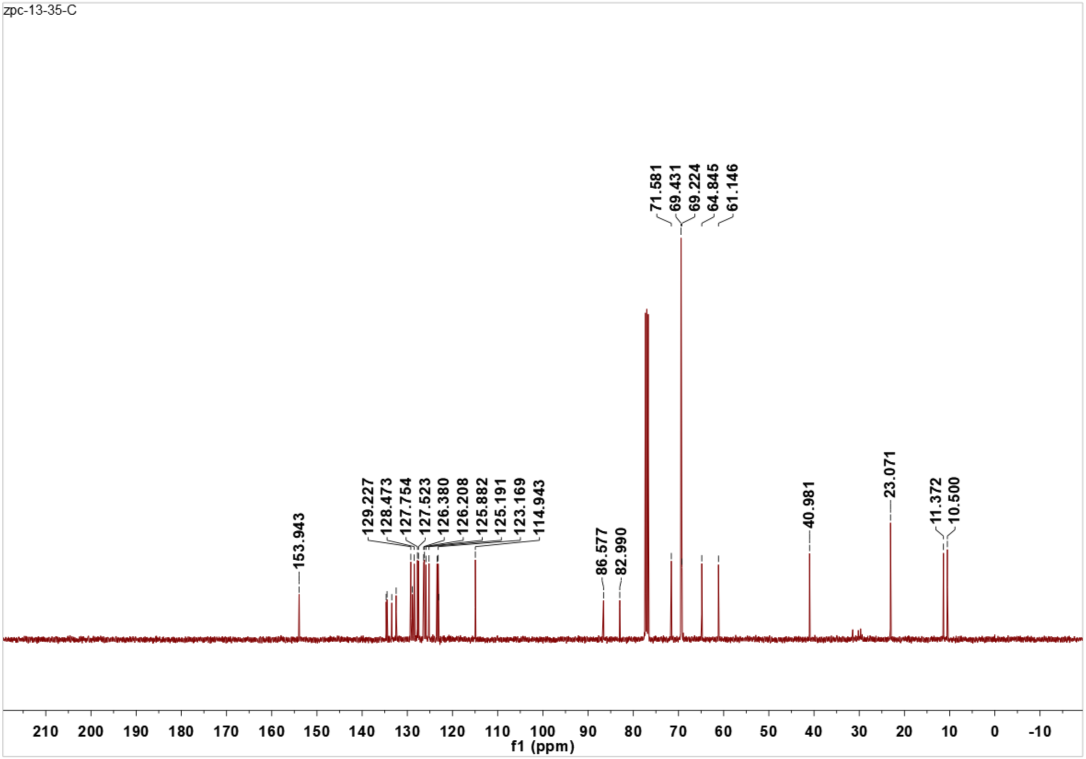

Supplementary Figure 126. ^{13}C NMR (400 MHz, $CDCl_3$) spectra for compound **2ak**

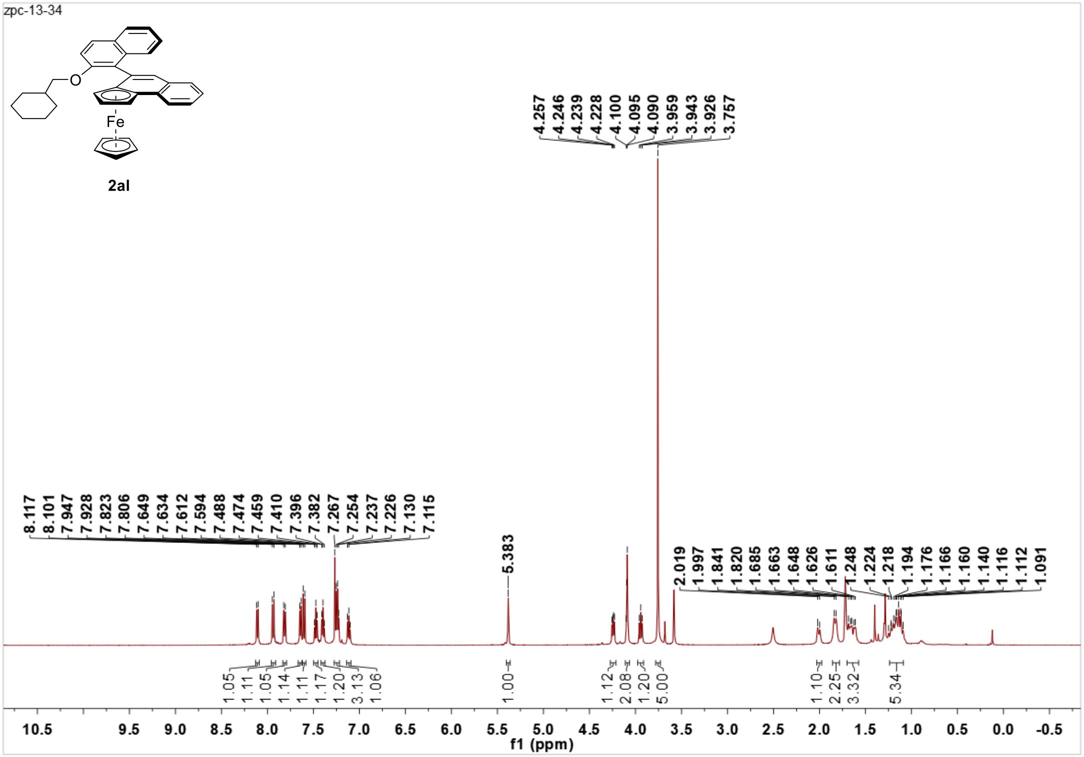

Supplementary Figure 127. ^{1}H NMR (500 MHz, THF-d_8) spectra for compound **2al**

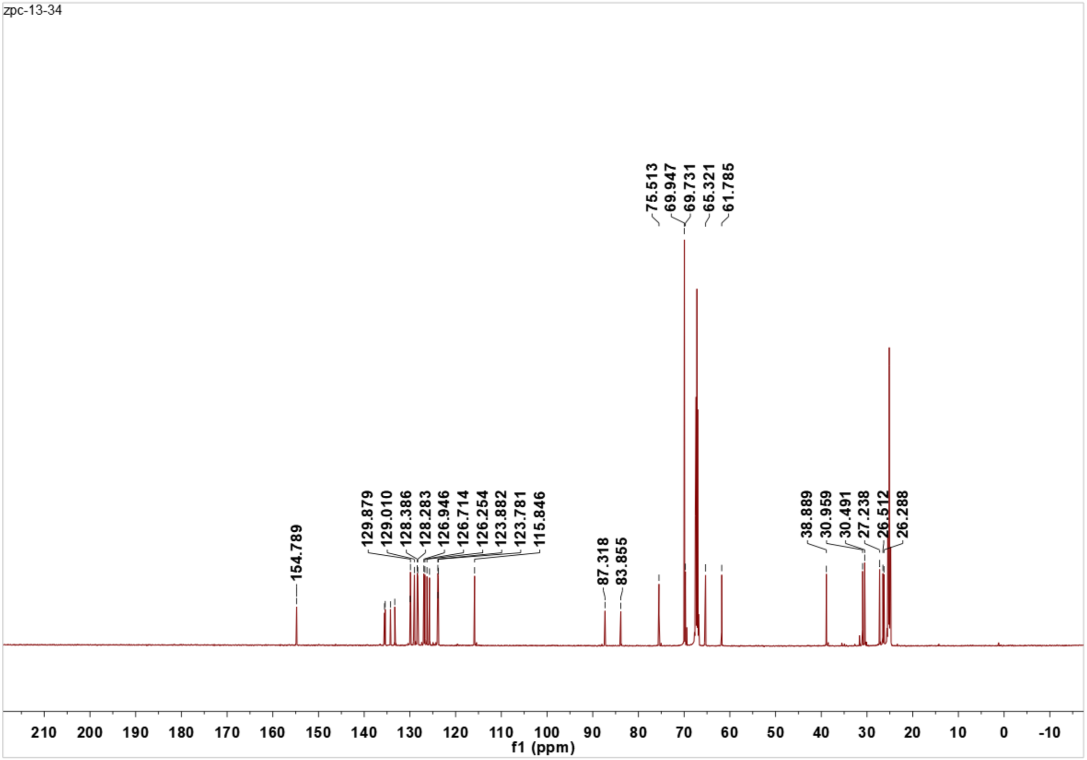

Supplementary Figure 128. ^{13}C NMR (500 MHz, THF-d_8) spectra for compound **2al**

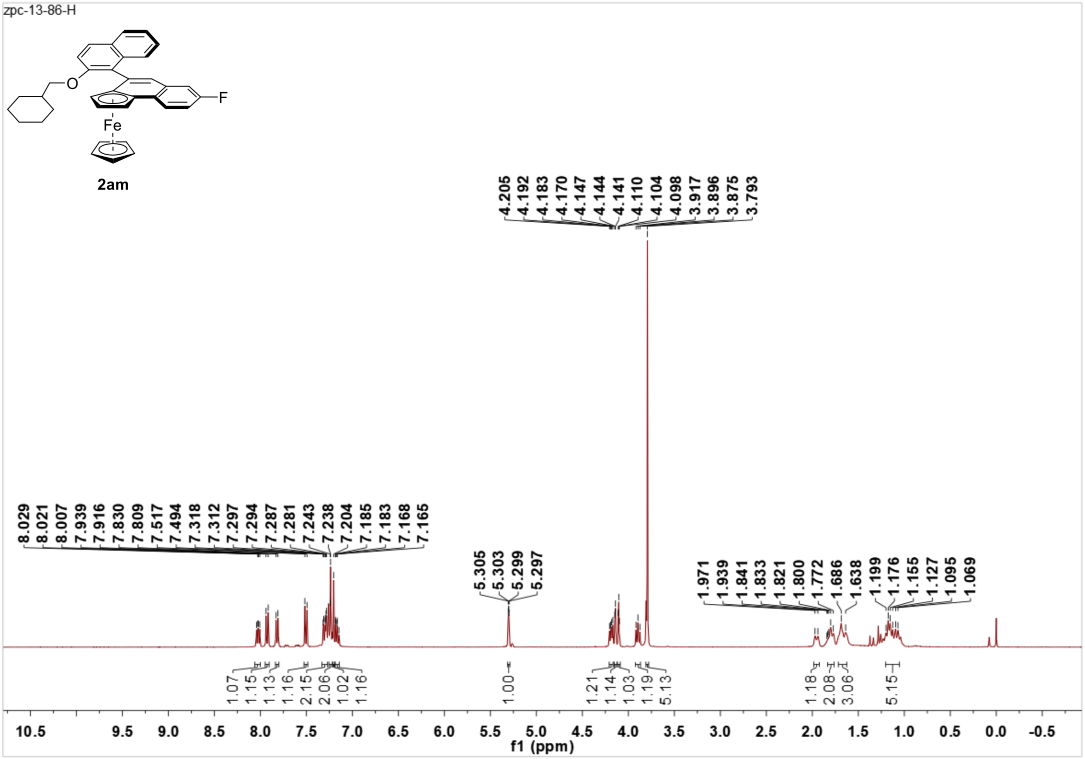

Supplementary Figure 129. ^{1}H NMR (400 MHz, $CDCl_3$) spectra for compound **2am**

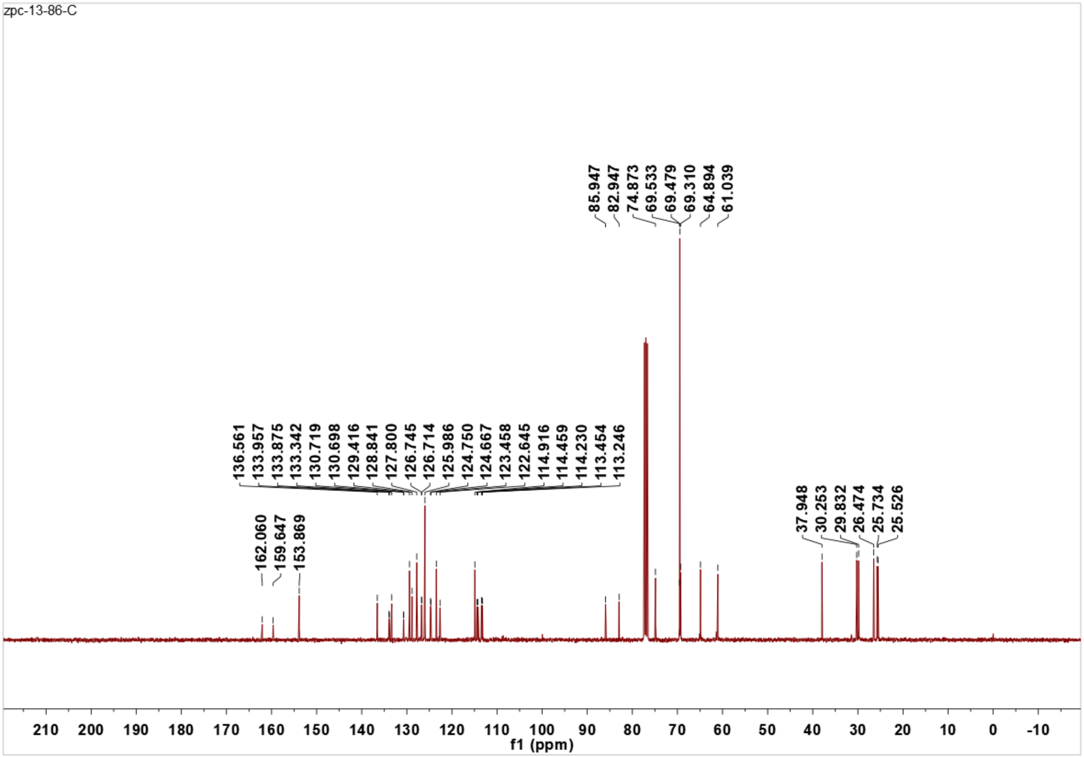

Supplementary Figure 130. ^{13}C NMR (400 MHz, $CDCl_3$) spectra for compound **2am**

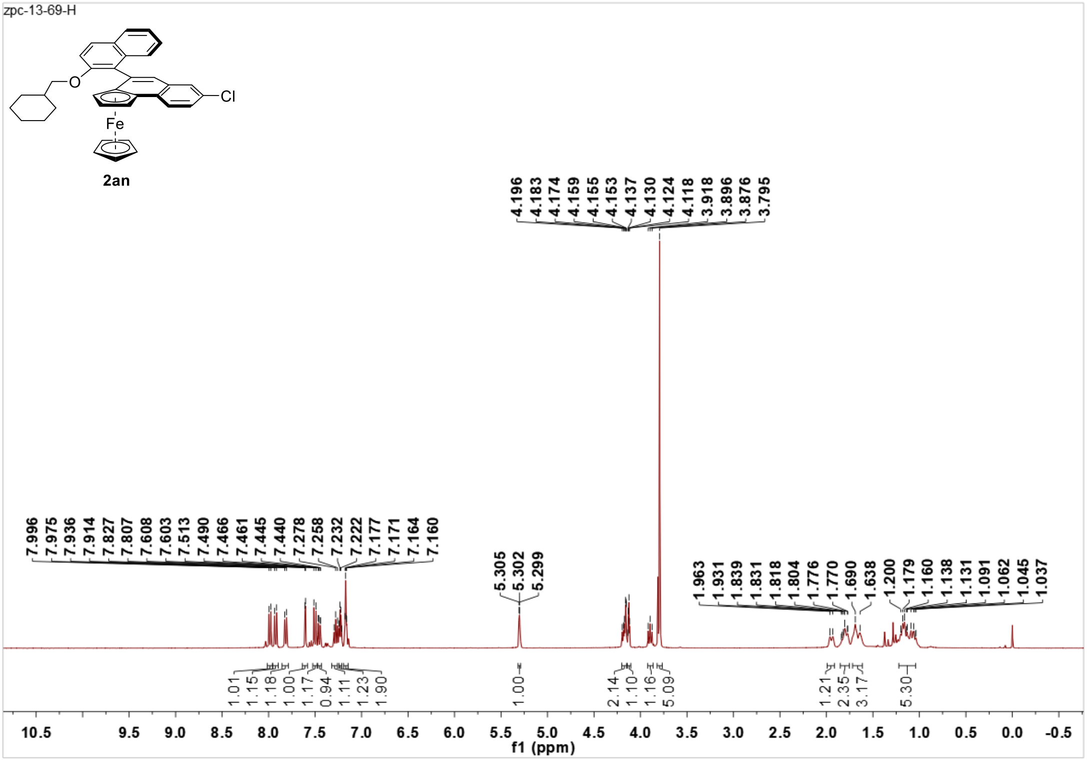

Supplementary Figure 131. ^{1}H NMR (400 MHz, $CDCl_3$) spectra for compound **2an**

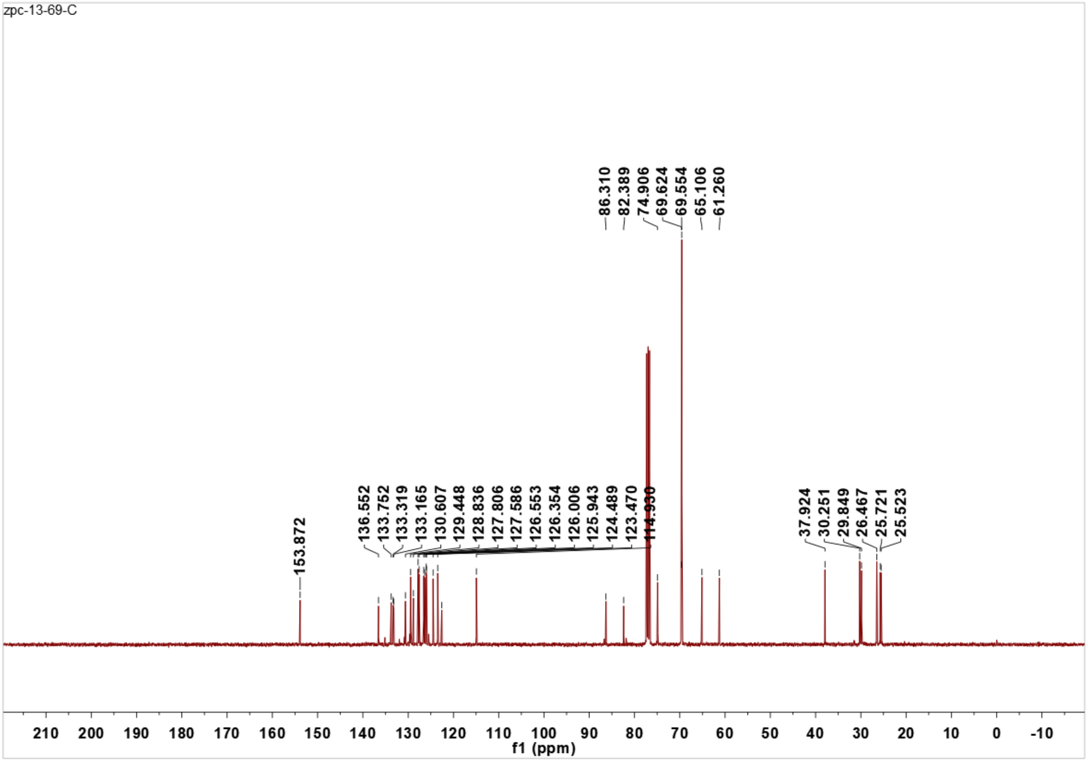

Supplementary Figure 132. ^{13}C NMR (400 MHz, $CDCl_3$) spectra for compound **2an**

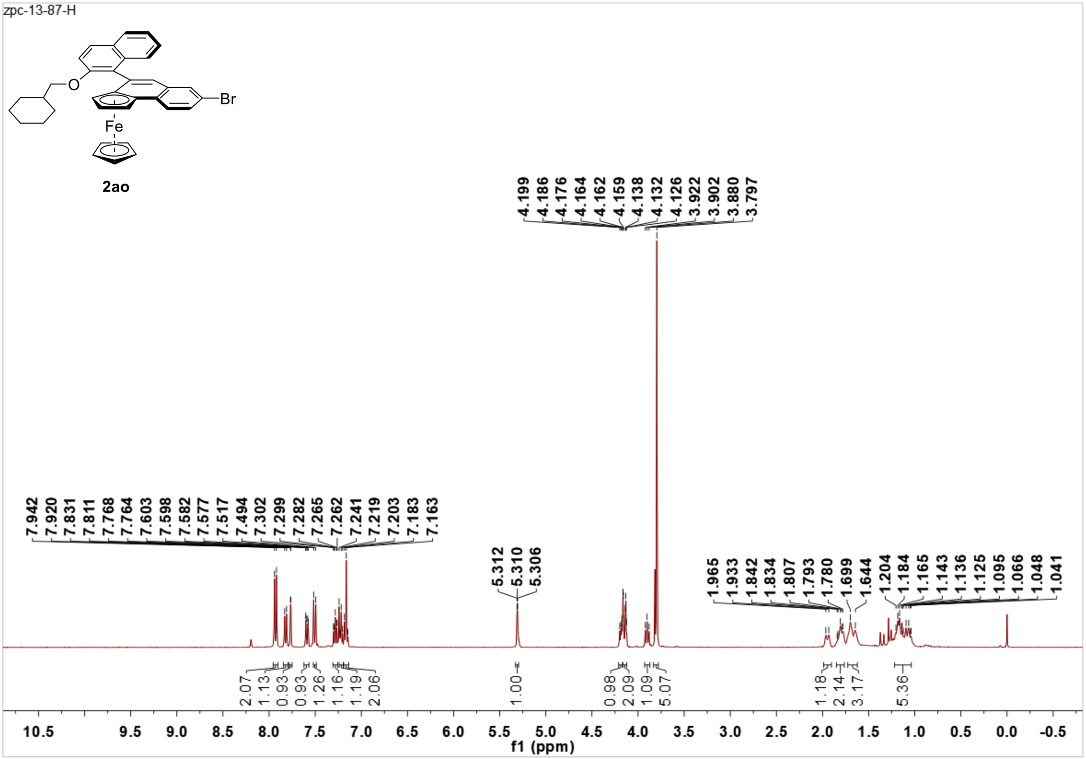

Supplementary Figure 133. ^{1}H NMR (400 MHz, $CDCl_3$) spectra for compound **2ao**

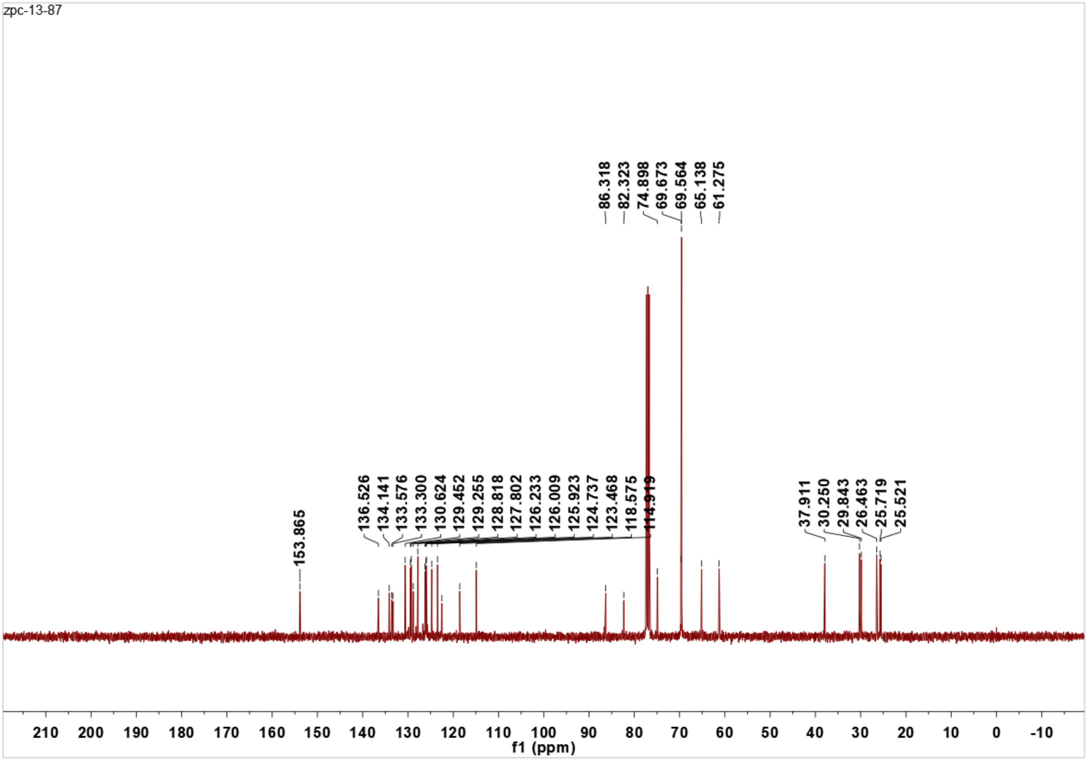

Supplementary Figure 134. ^{13}C NMR (400 MHz, $CDCl_3$) spectra for compound **2ao**

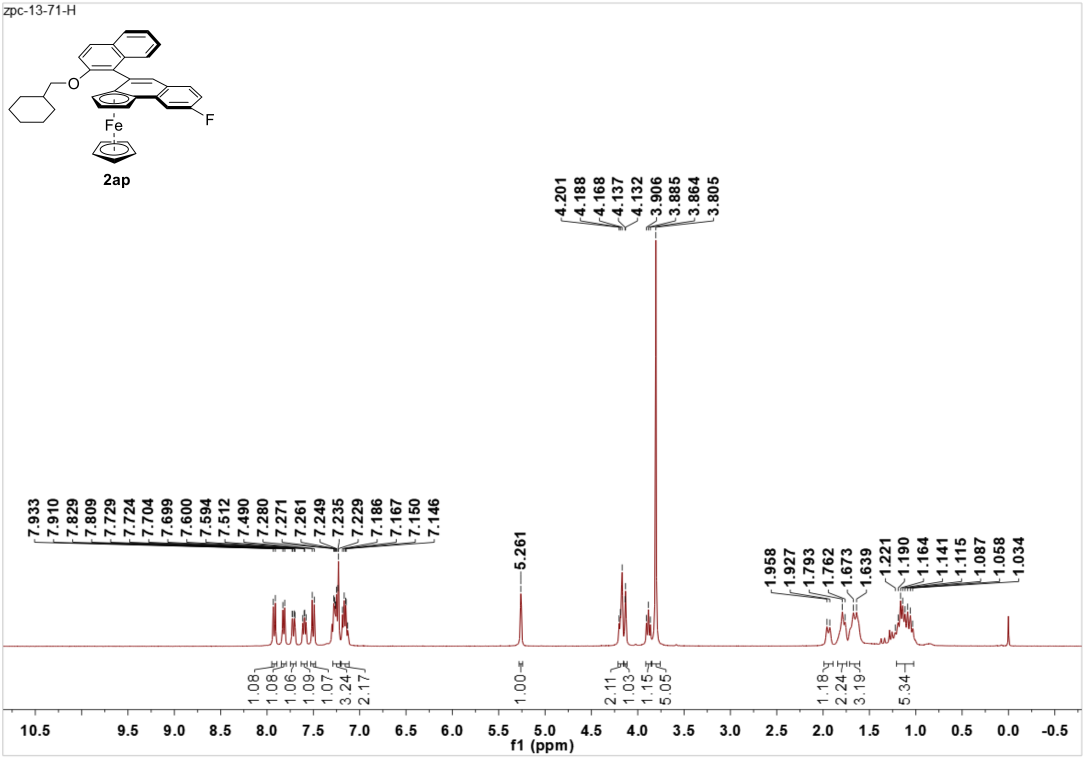

Supplementary Figure 135. ^{1}H NMR (400 MHz, $CDCl_3$) spectra for compound **2ap**

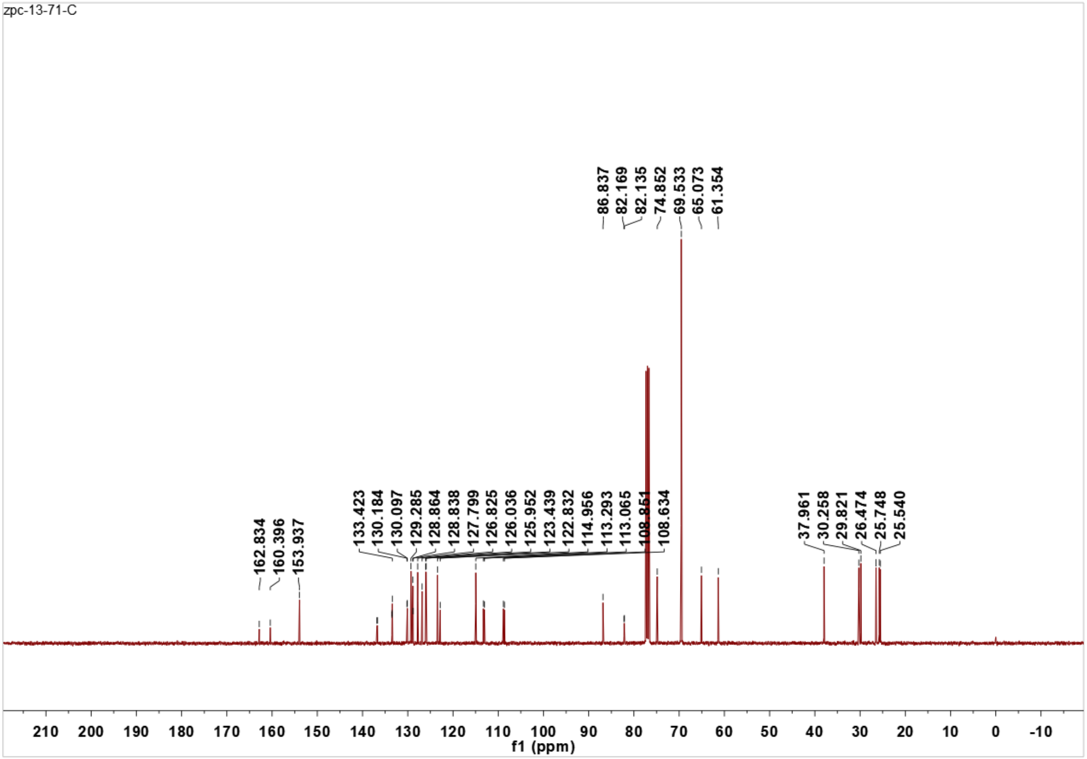

Supplementary Figure 136. ^{13}C NMR (400 MHz, $CDCl_3$) spectra for compound **2ap**

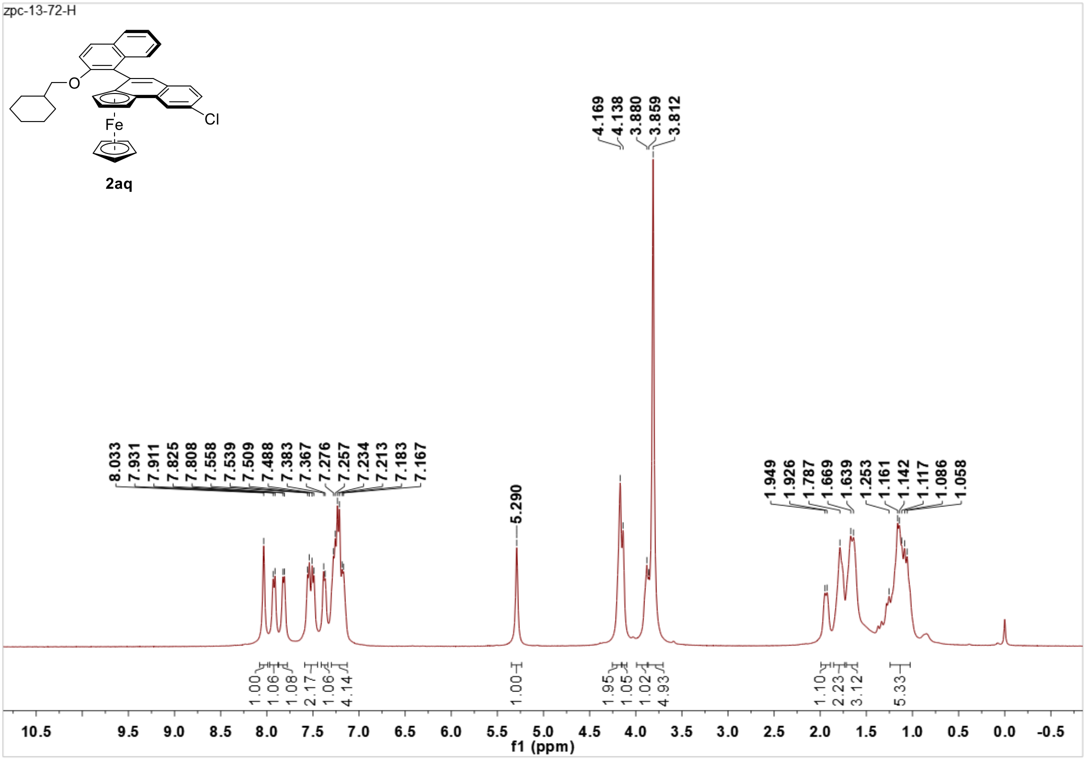

Supplementary Figure 137. ^{1}H NMR (400 MHz, $CDCl_3$) spectra for compound **2aq**

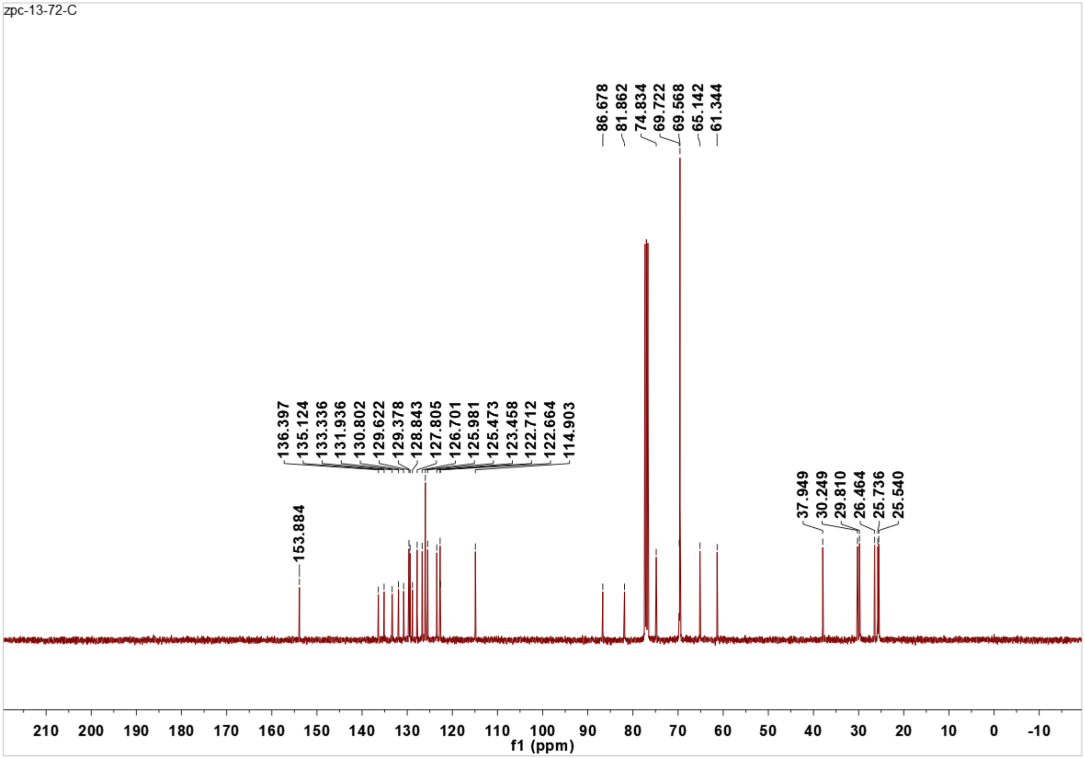

Supplementary Figure 138. ^{13}C NMR (400 MHz, $CDCl_3$) spectra for compound **2aq**

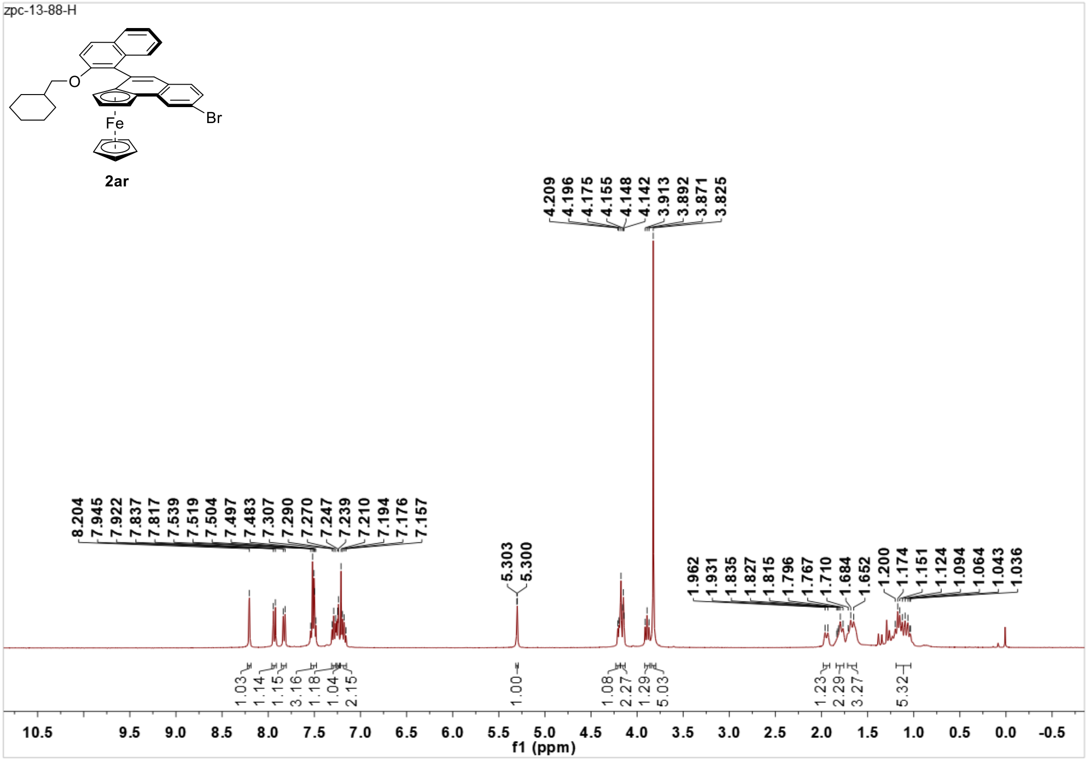

Supplementary Figure 139. ^{1}H NMR (400 MHz, $CDCl_3$) spectra for compound **2ar**

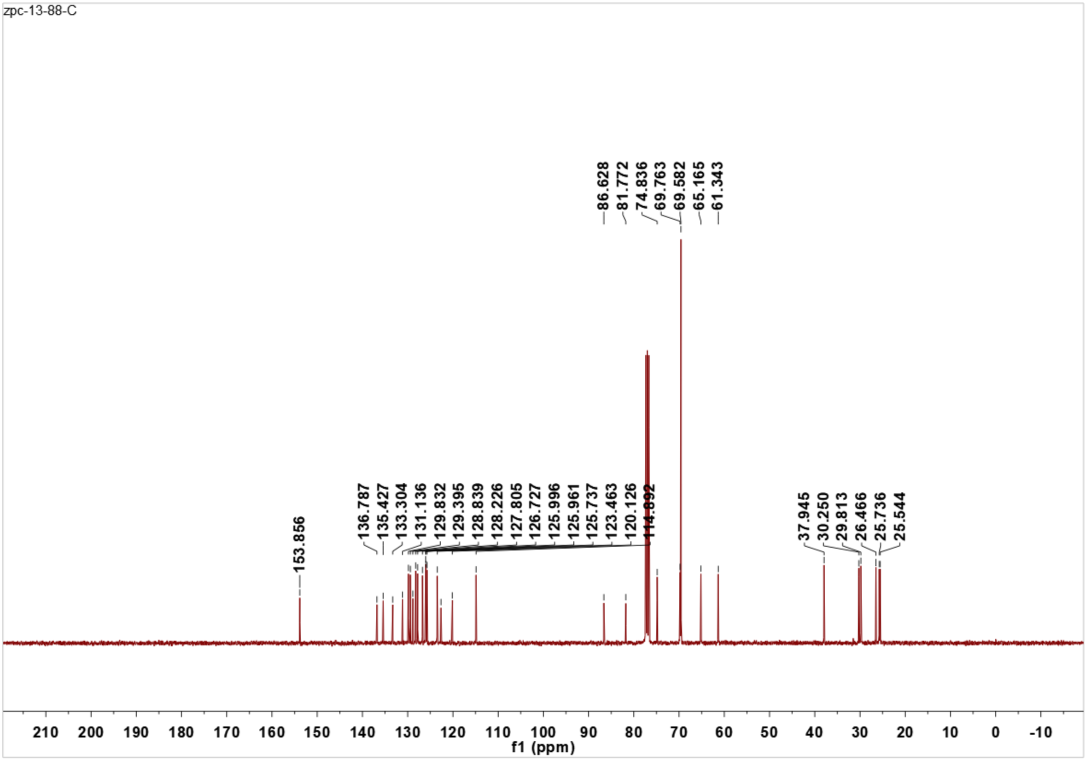

Supplementary Figure 140. ^{13}C NMR (400 MHz, $CDCl_3$) spectra for compound **2ar**

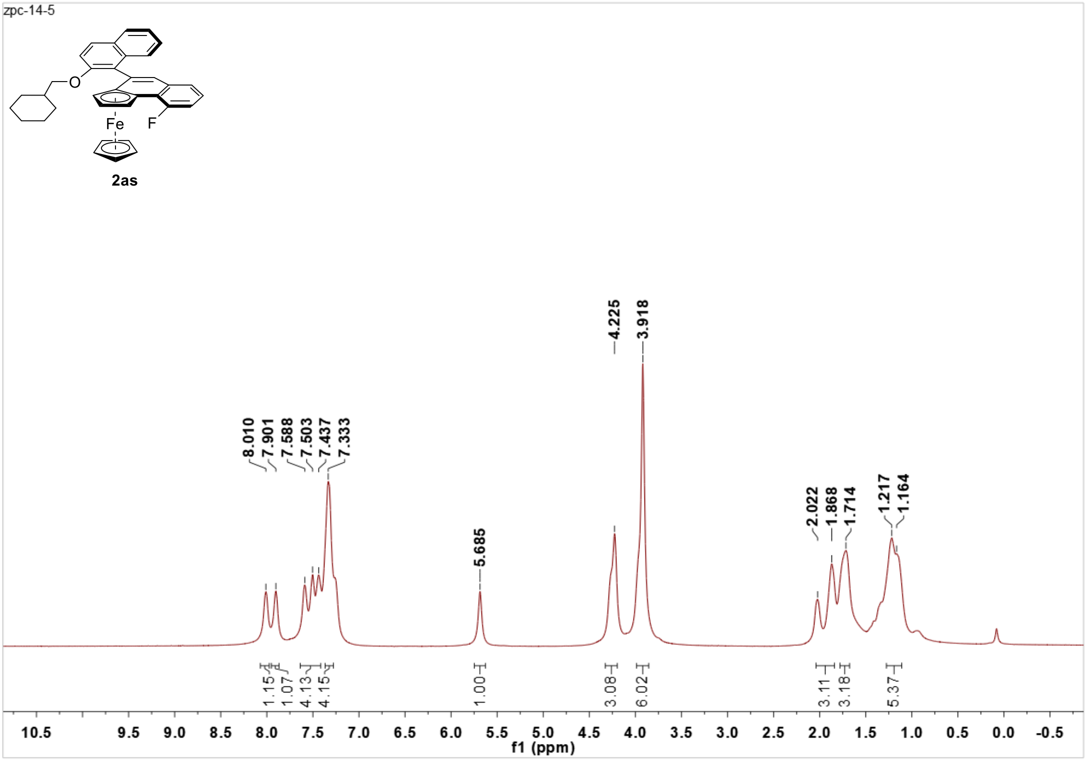

Supplementary Figure 141. ^{1}H NMR (500 MHz, $CDCl_3$) spectra for compound **2as**

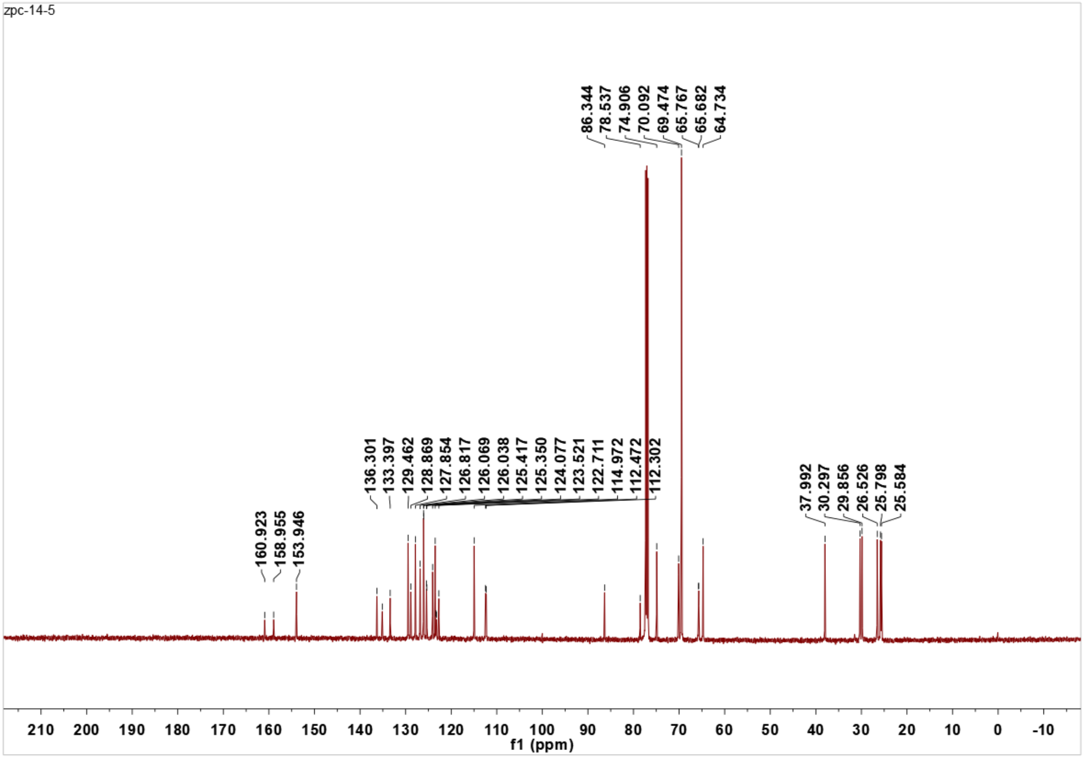

Supplementary Figure 142. ^{13}C NMR (500 MHz, $CDCl_3$) spectra for compound **2as**

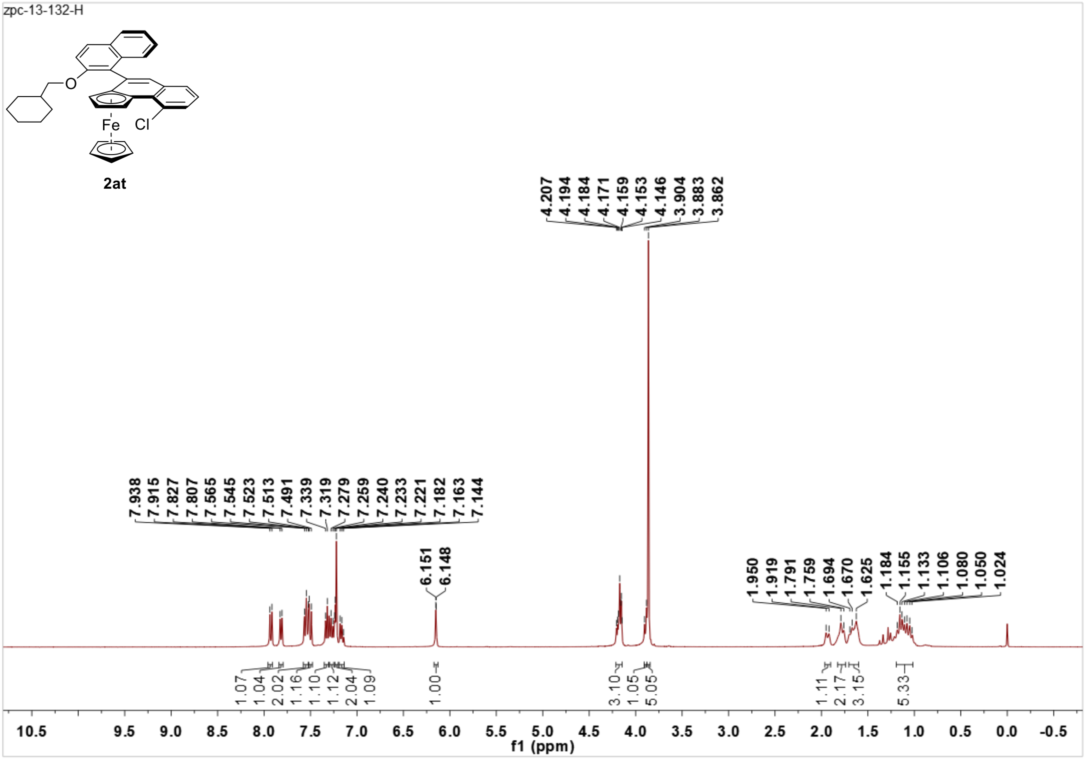

Supplementary Figure 143. ^{1}H NMR (400 MHz, $CDCl_3$) spectra for compound **2at**

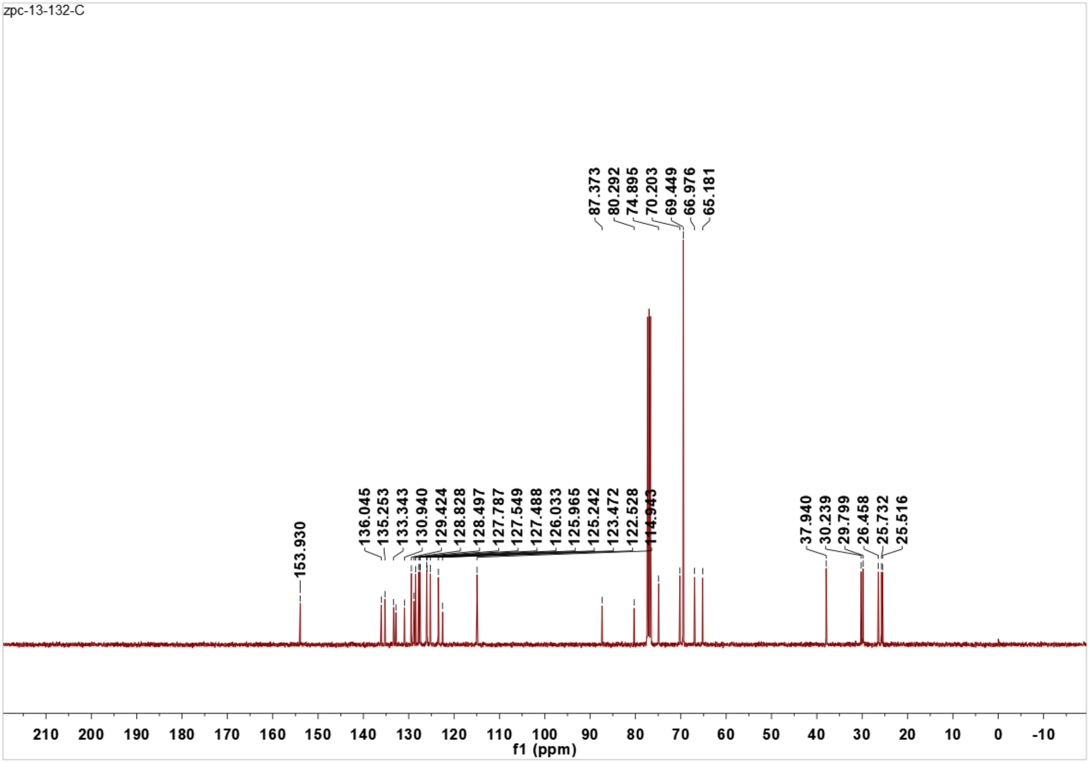

Supplementary Figure 144. ^{13}C NMR (400 MHz, $CDCl_3$) spectra for compound **2at**

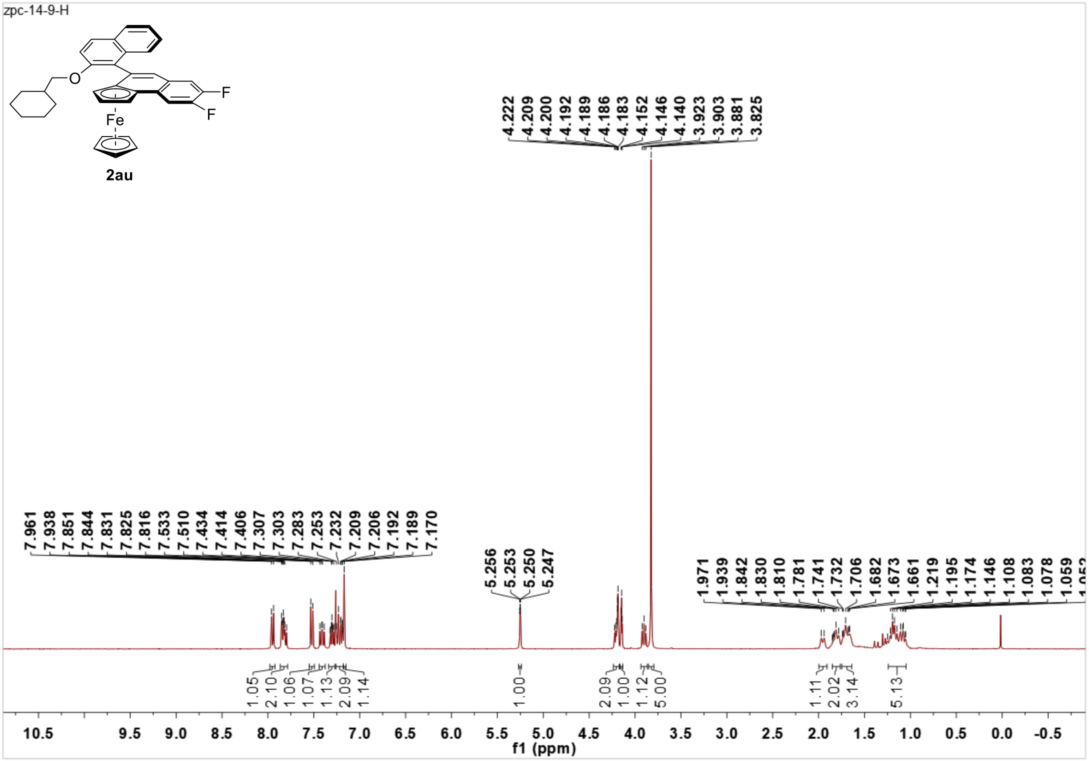

Supplementary Figure 145. ^{1}H NMR (400 MHz, $CDCl_3$) spectra for compound **2au**

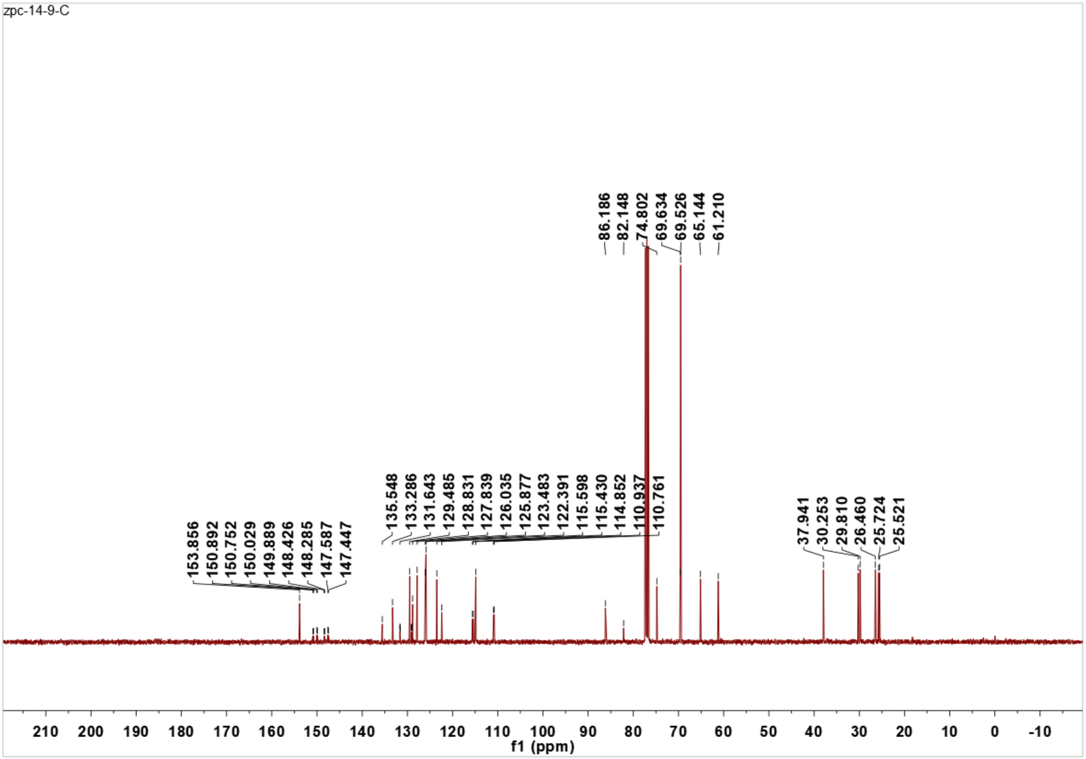

Supplementary Figure 146. ^{13}C NMR (400 MHz, $CDCl_3$) spectra for compound **2au**

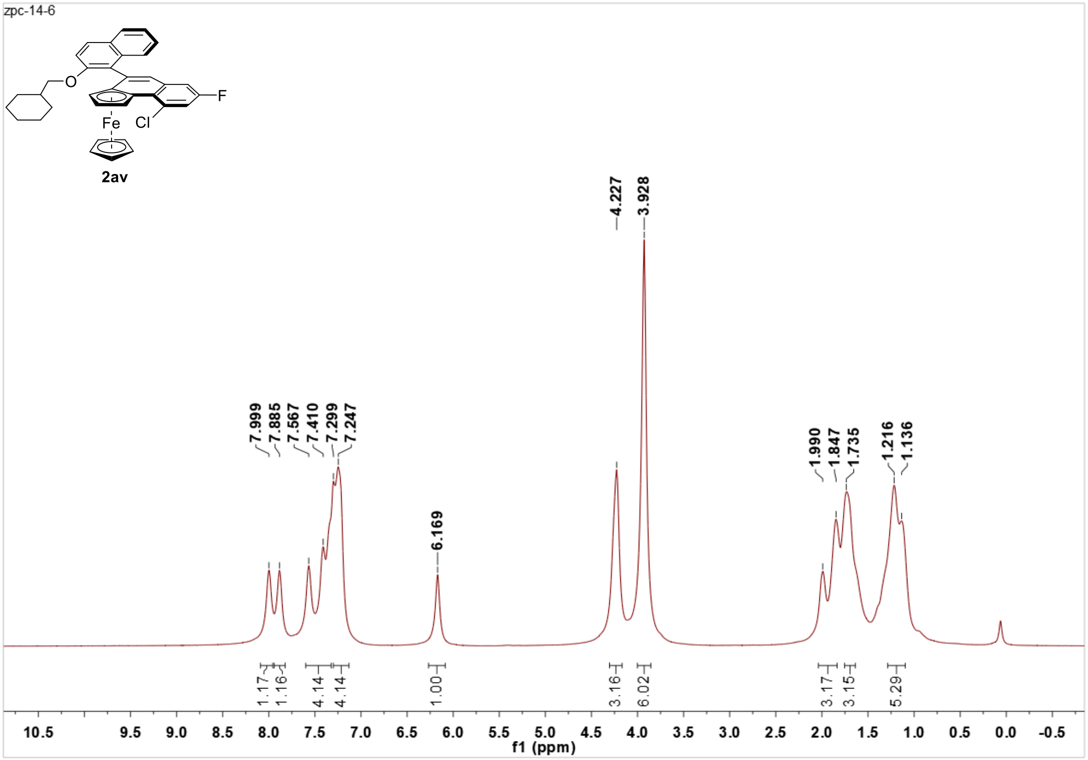

Supplementary Figure 147. ^{1}H NMR (500 MHz, $CDCl_3$) spectra for compound **2av**

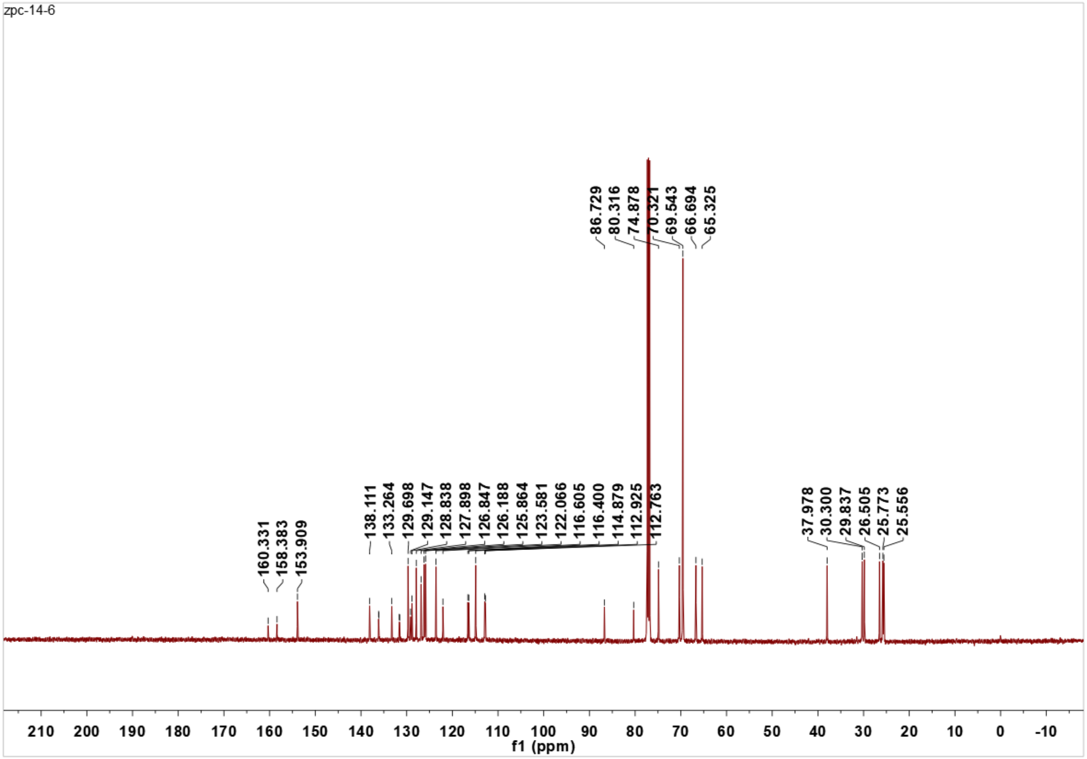

Supplementary Figure 148. ^{13}C NMR (500 MHz, $CDCl_3$) spectra for compound **2av**

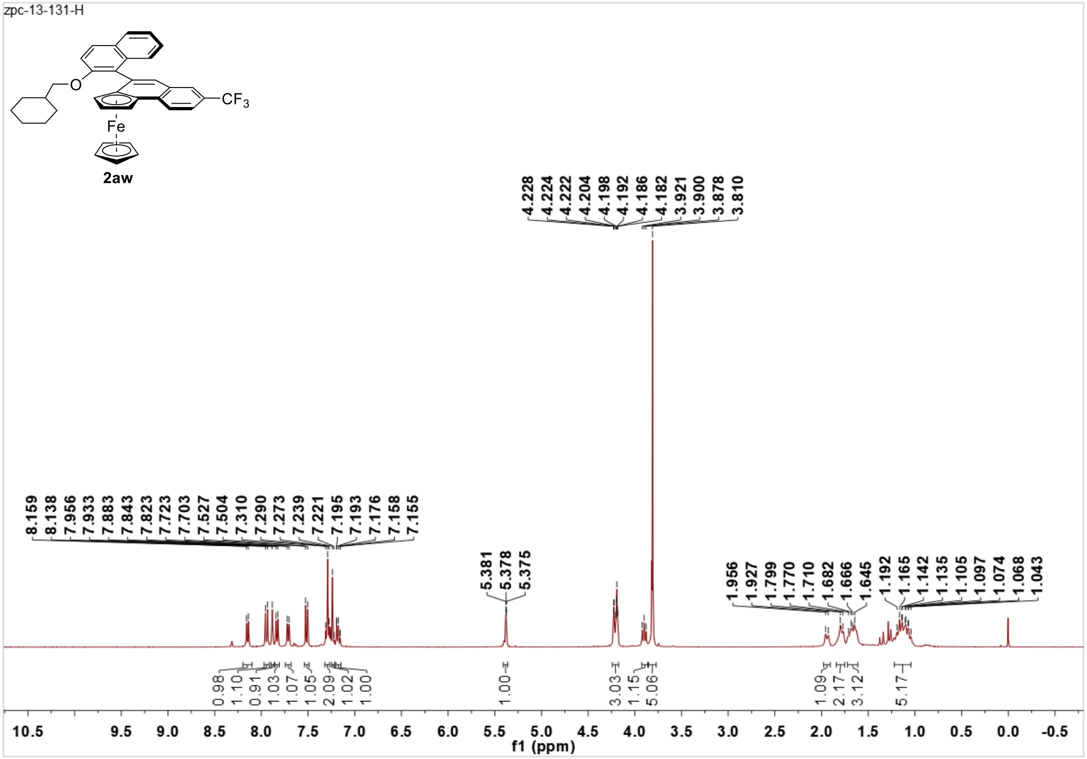

Supplementary Figure 149. ^{1}H NMR (400 MHz, $CDCl_3$) spectra for compound **2aw**

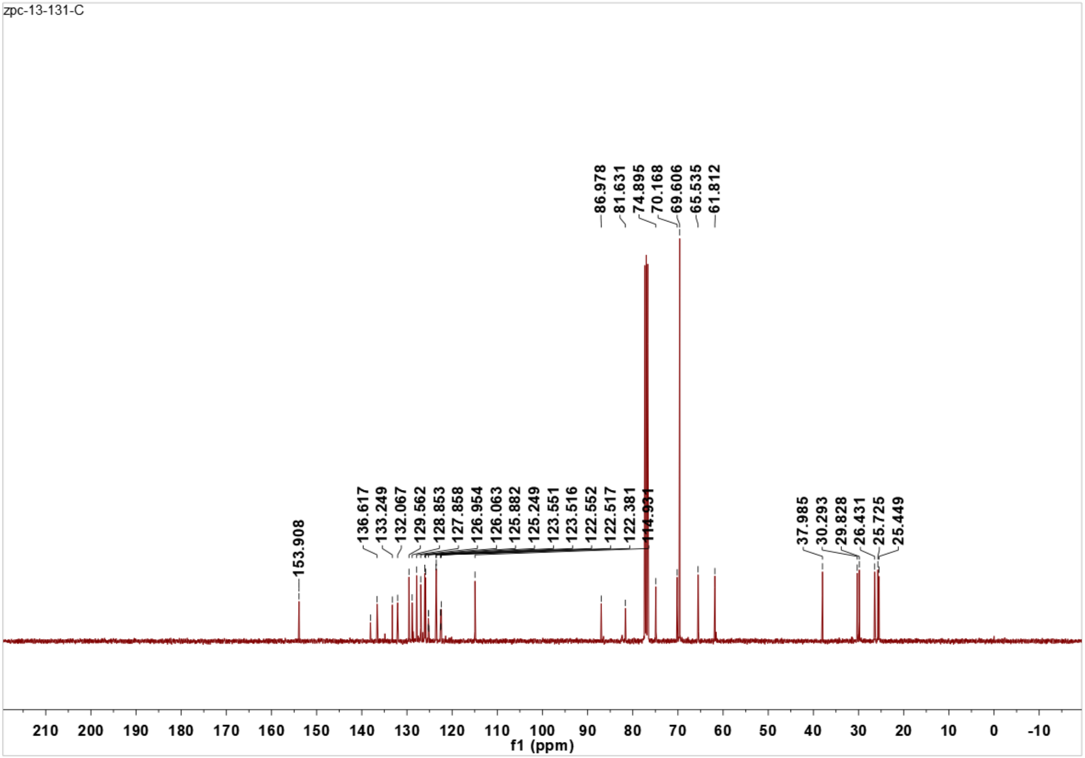

Supplementary Figure 150. ^{13}C NMR (400 MHz, $CDCl_3$) spectra for compound **2aw**

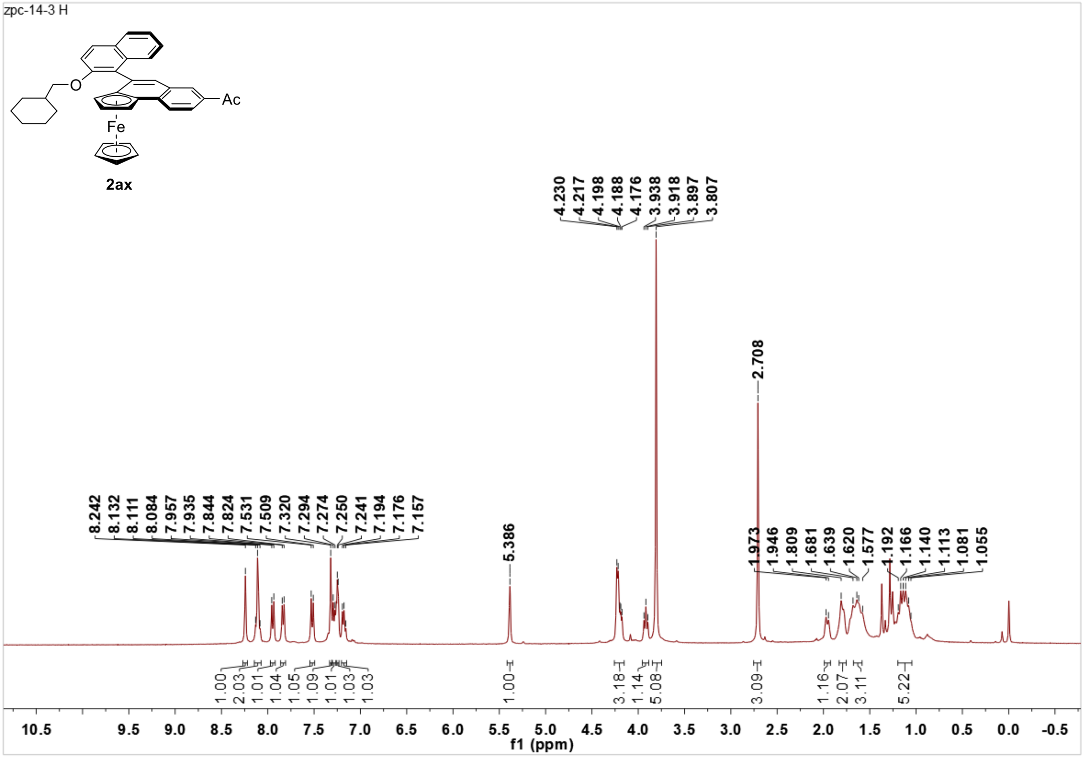

Supplementary Figure 151. ^{1}H NMR (400 MHz, $CDCl_3$) spectra for compound **2ax**

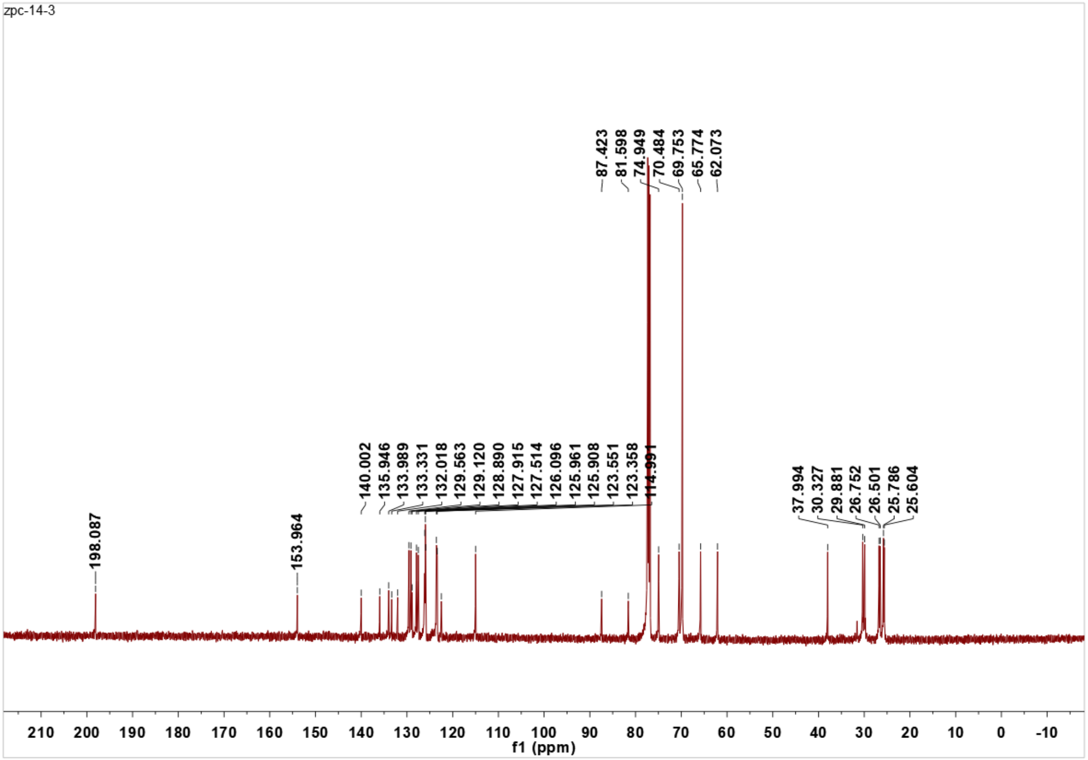

Supplementary Figure 152. ^{13}C NMR (400 MHz, $CDCl_3$) spectra for compound **2ax**

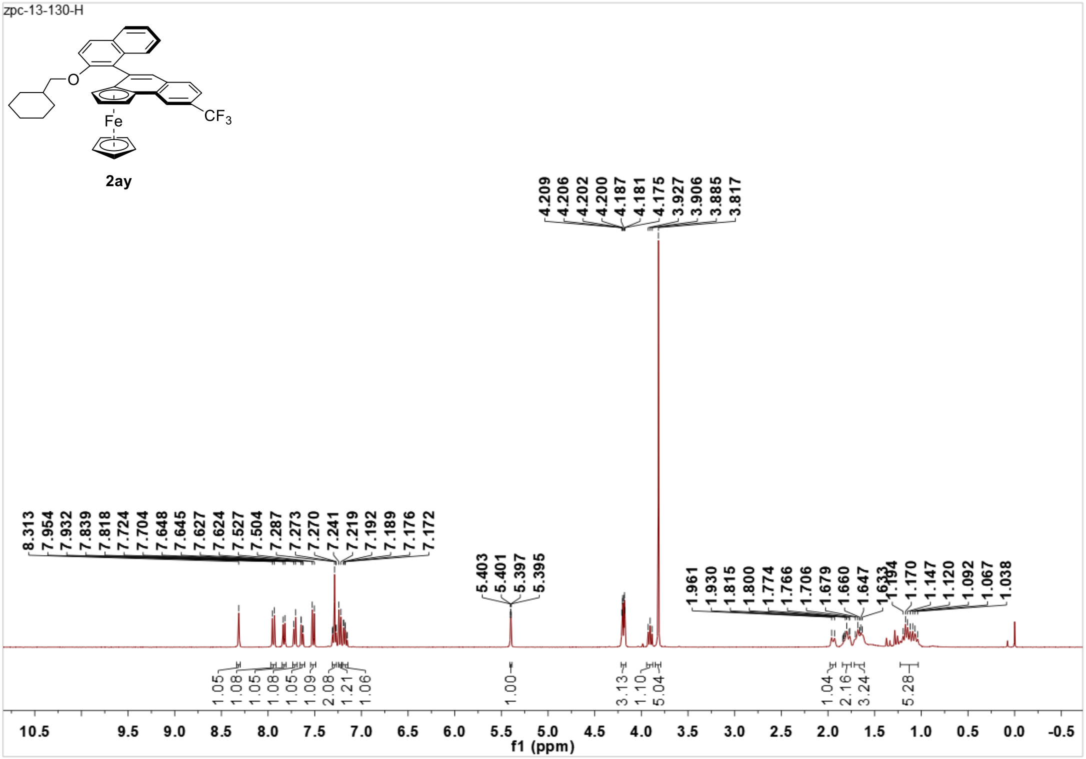

Supplementary Figure 153. ^{1}H NMR (400 MHz, $CDCl_3$) spectra for compound **2ay**

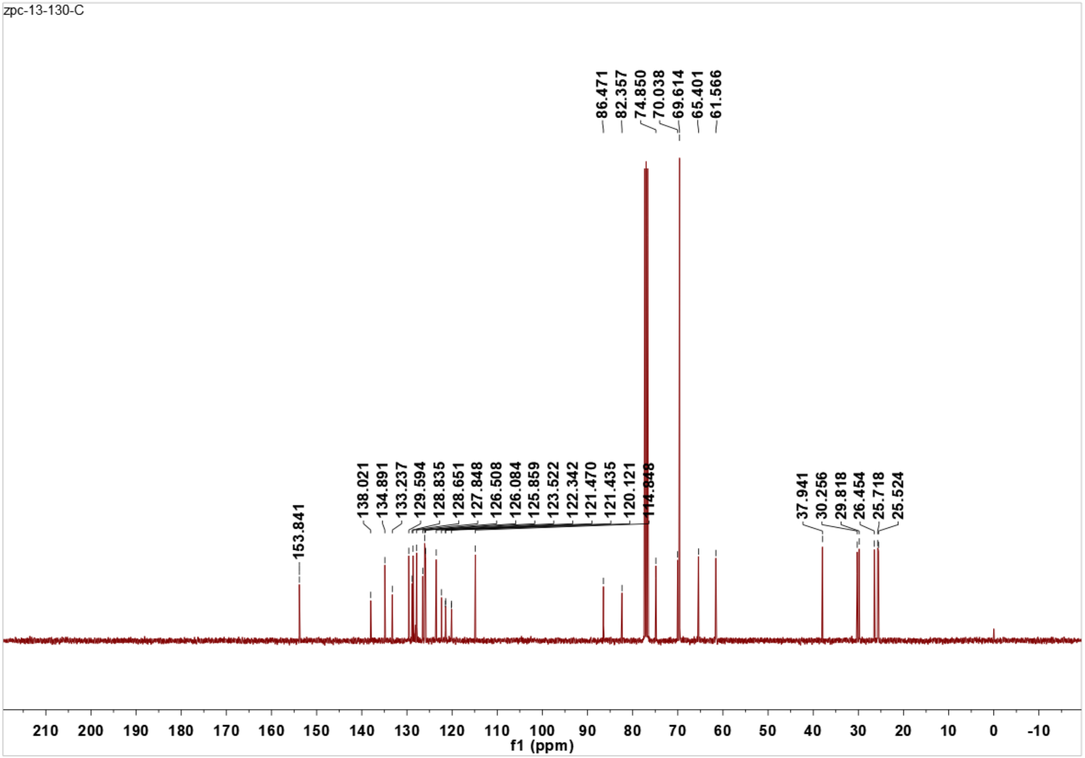

Supplementary Figure 154. ^{13}C NMR (400 MHz, $CDCl_3$) spectra for compound **2ay**

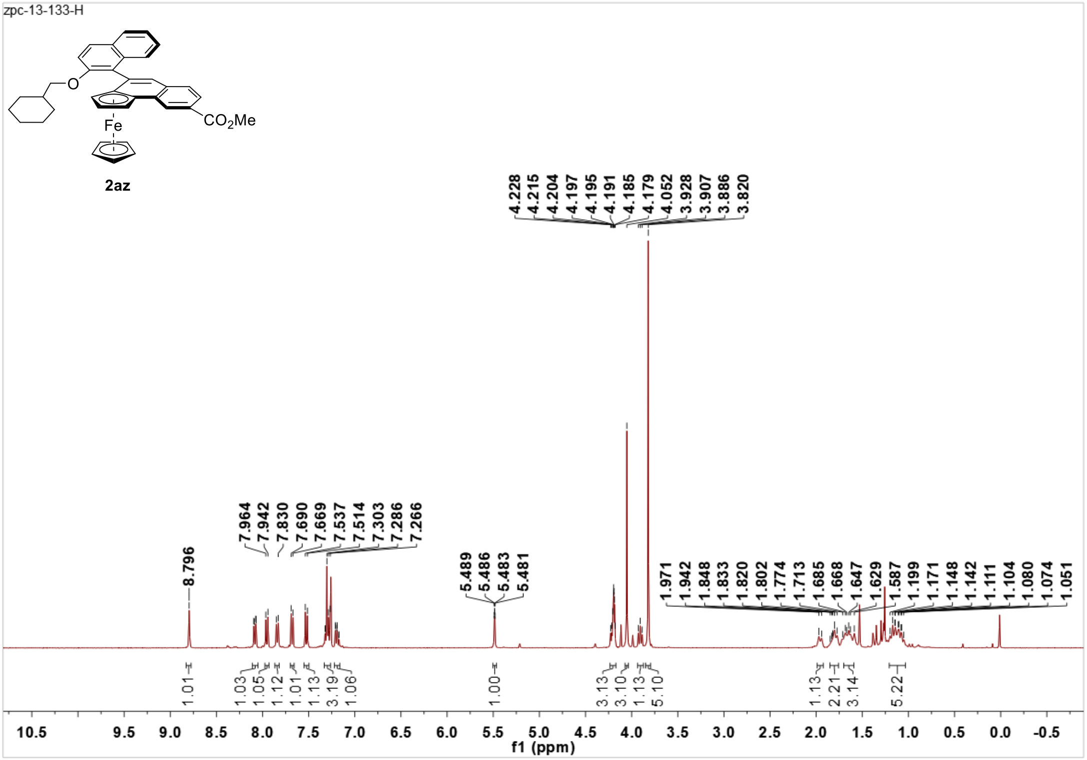

Supplementary Figure 155. ^{1}H NMR (400 MHz, $CDCl_3$) spectra for compound **2az**

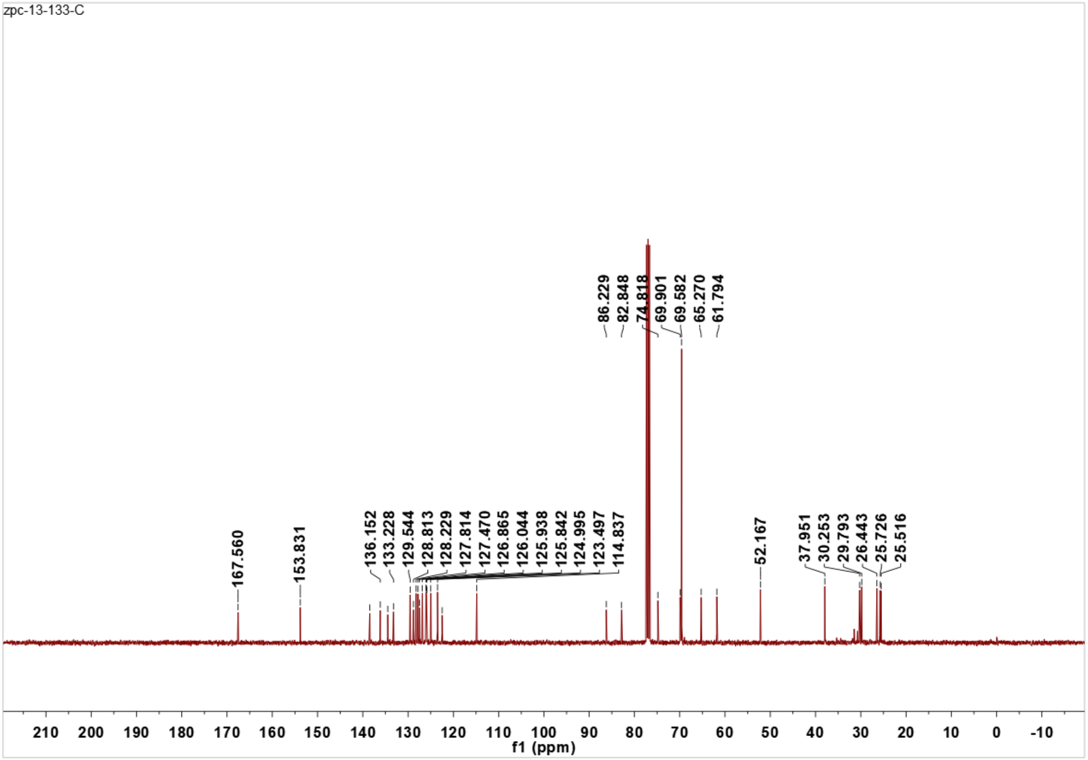

Supplementary Figure 156. ^{13}C NMR (400 MHz, $CDCl_3$) spectra for compound **2az**

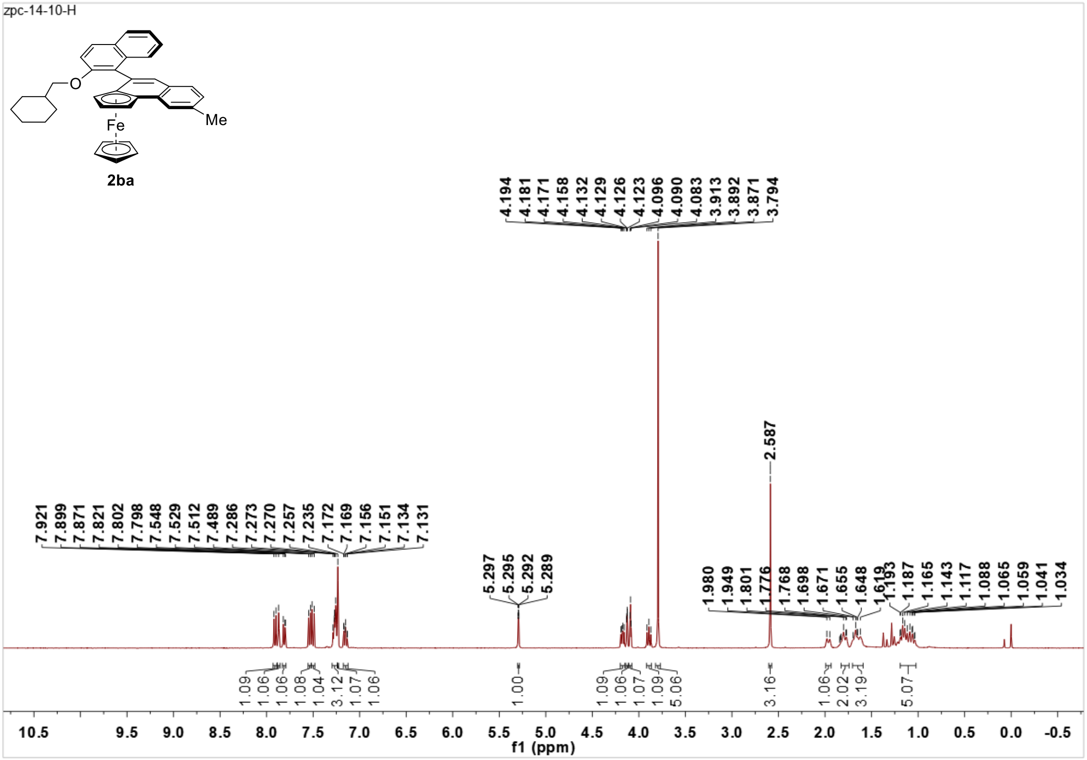

Supplementary Figure 157. ^{1}H NMR (400 MHz, $CDCl_3$) spectra for compound **2ba**

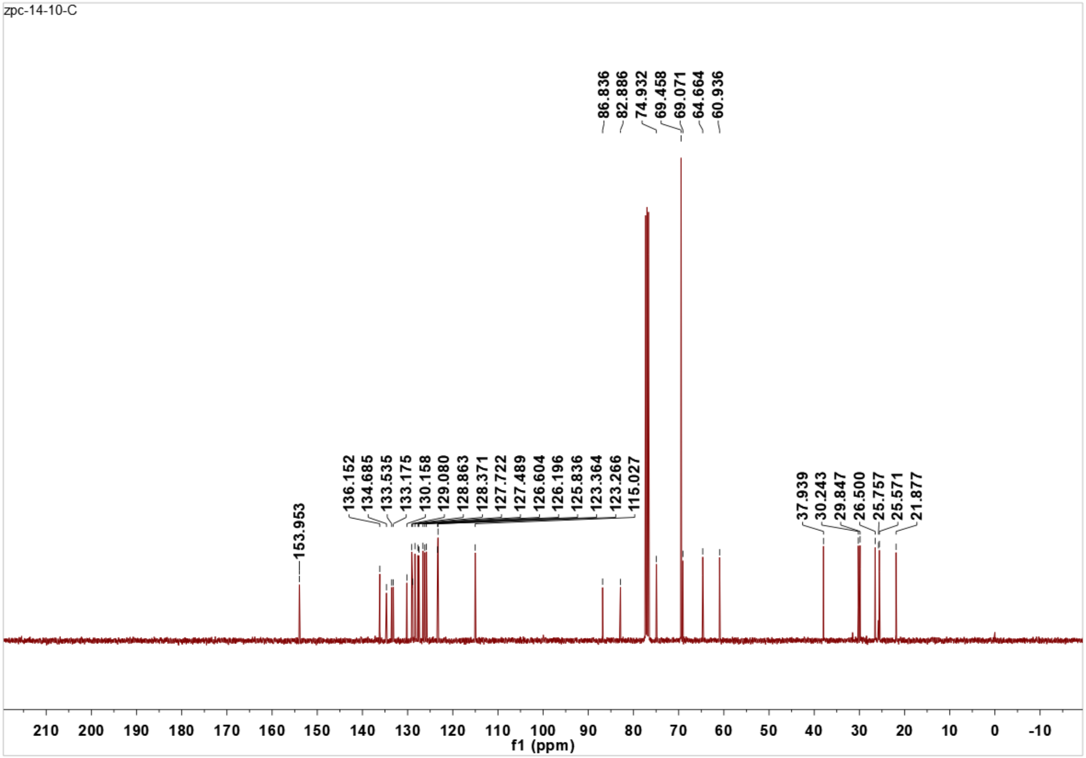

Supplementary Figure 158. ^{13}C NMR (400 MHz, $CDCl_3$) spectra for compound **2ba**

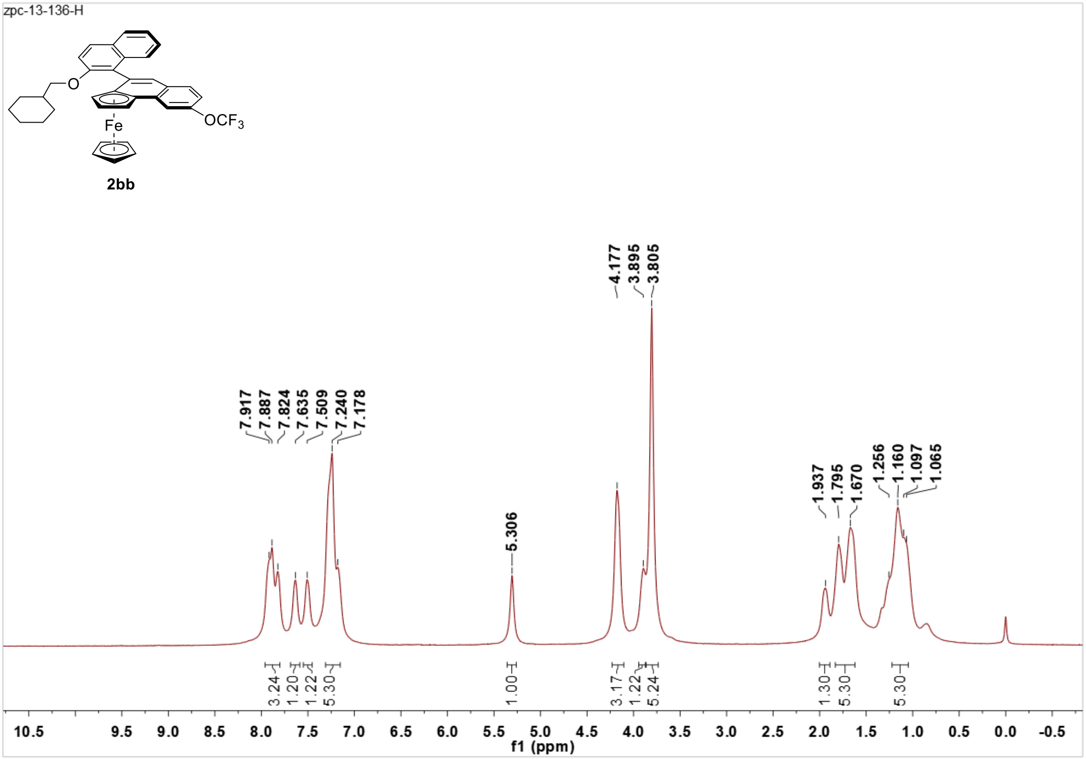

Supplementary Figure 159. ^{1}H NMR (400 MHz, $CDCl_3$) spectra for compound **2bb**

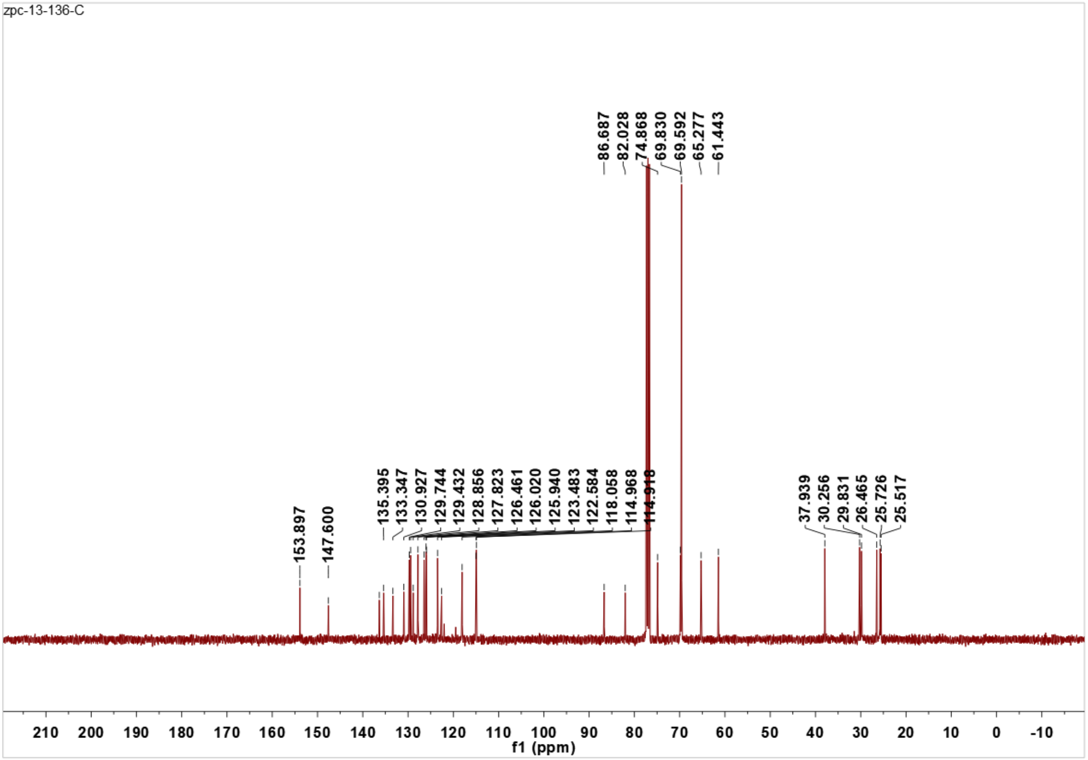

Supplementary Figure 160. ^{13}C NMR (400 MHz, $CDCl_3$) spectra for compound **2bb**

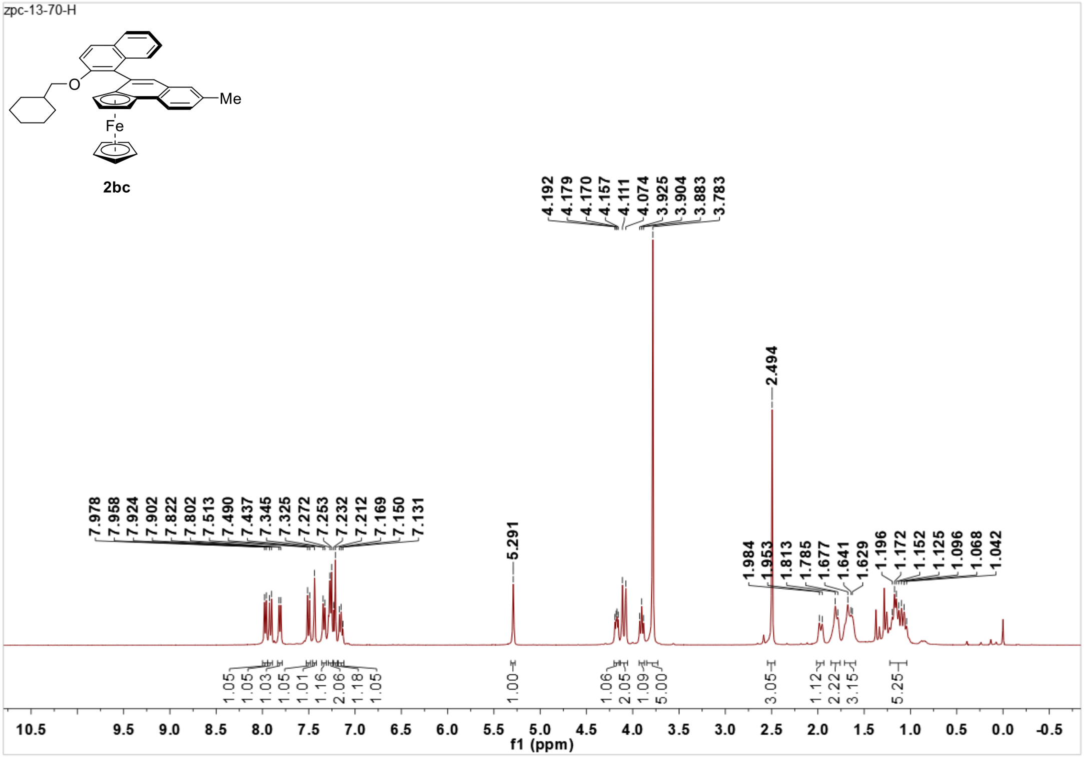

Supplementary Figure 161. ^{1}H NMR (400 MHz, $CDCl_3$) spectra for compound **2bc**

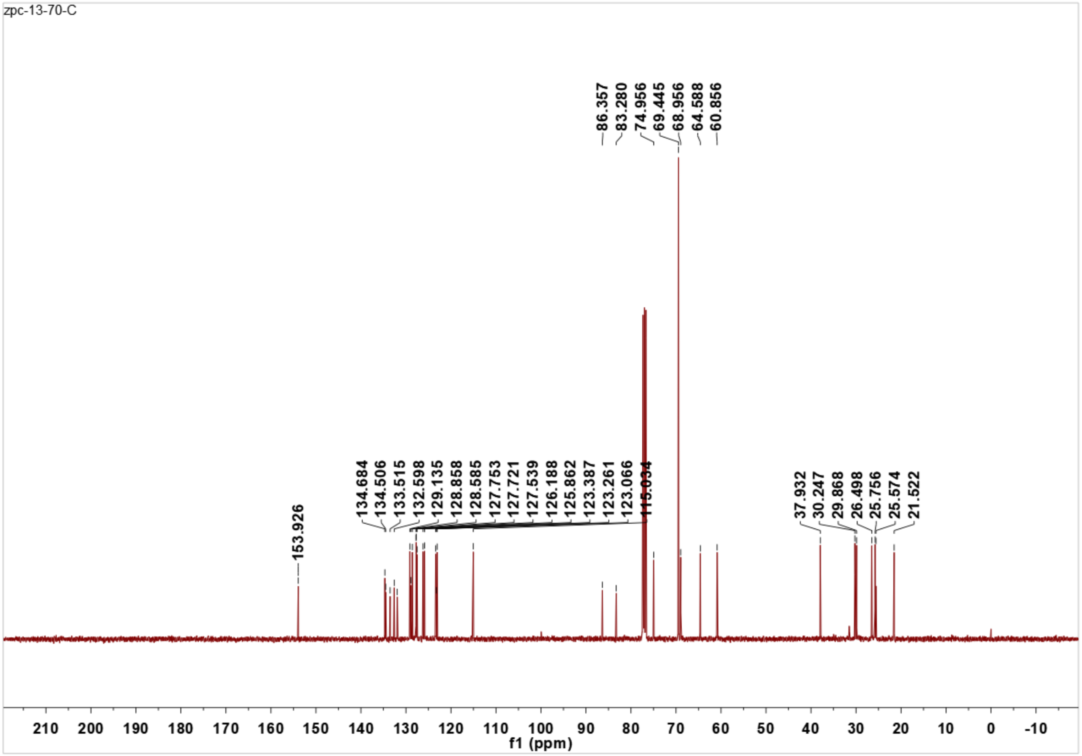

Supplementary Figure 162. ^{13}C NMR (400 MHz, $CDCl_3$) spectra for compound **2bc**

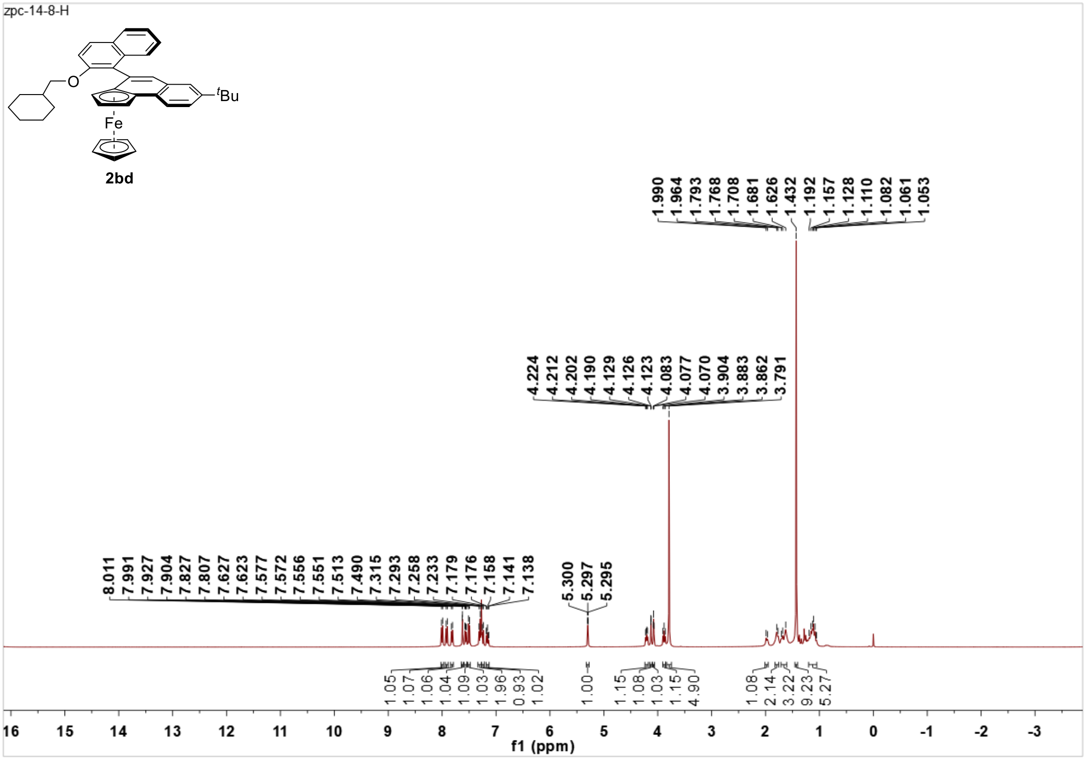

Supplementary Figure 163. ^{1}H NMR (400 MHz, $CDCl_3$) spectra for compound **2bd**

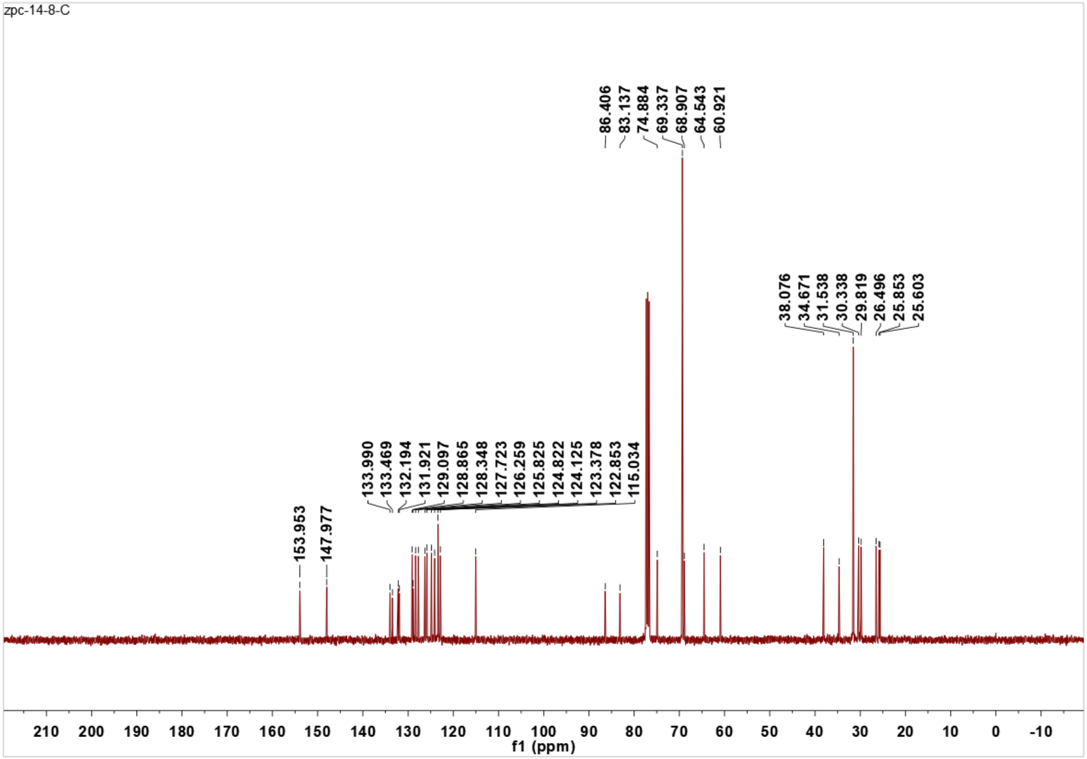

Supplementary Figure 164. ^{13}C NMR (400 MHz, $CDCl_3$) spectra for compound **2bd**

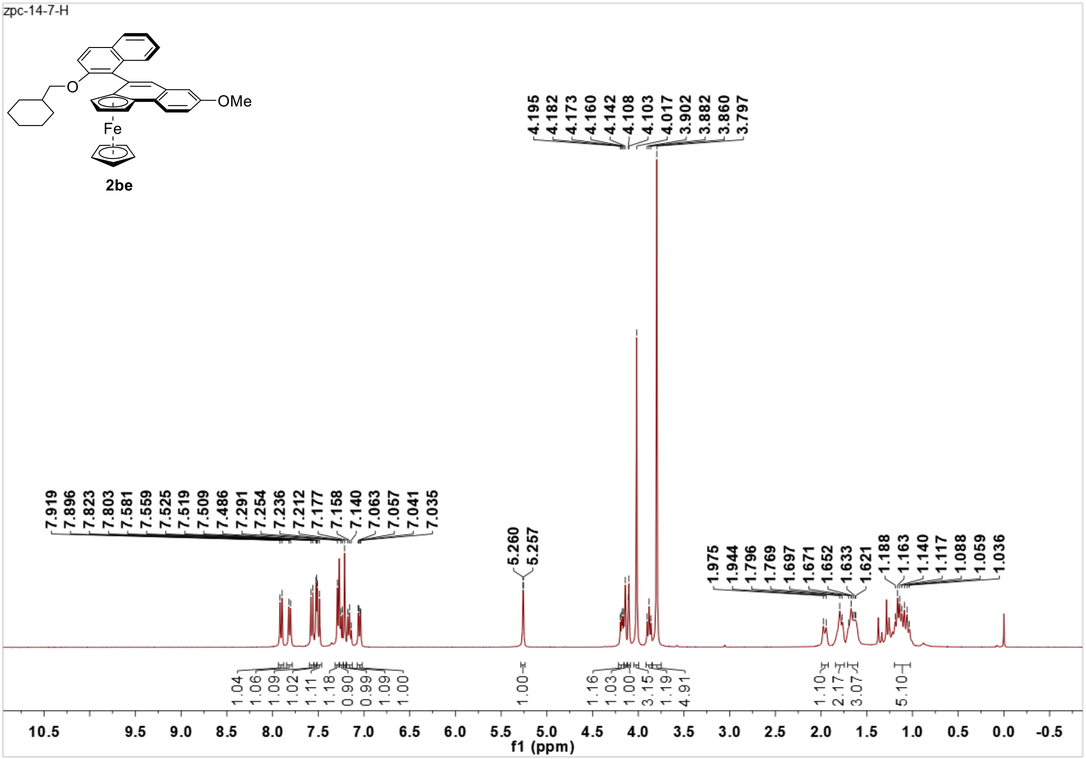

Supplementary Figure 165. ^{1}H NMR (400 MHz, $CDCl_3$) spectra for compound **2be**

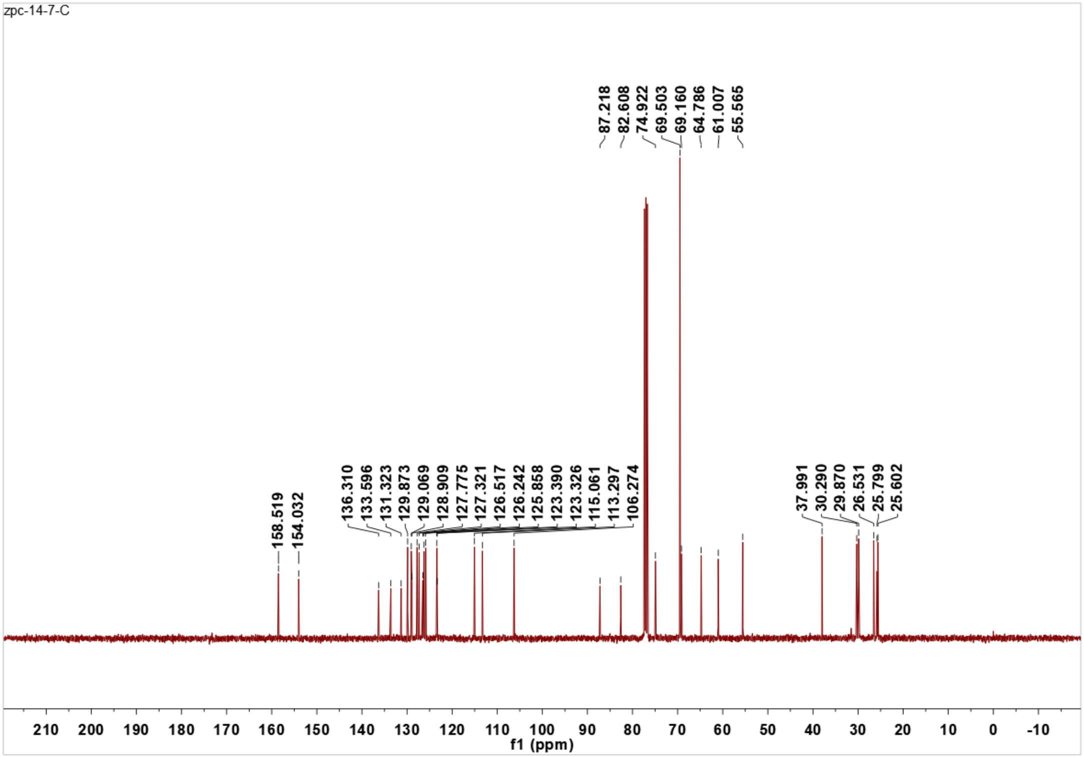

Supplementary Figure 166. ^{13}C NMR (400 MHz, $CDCl_3$) spectra for compound **2be**

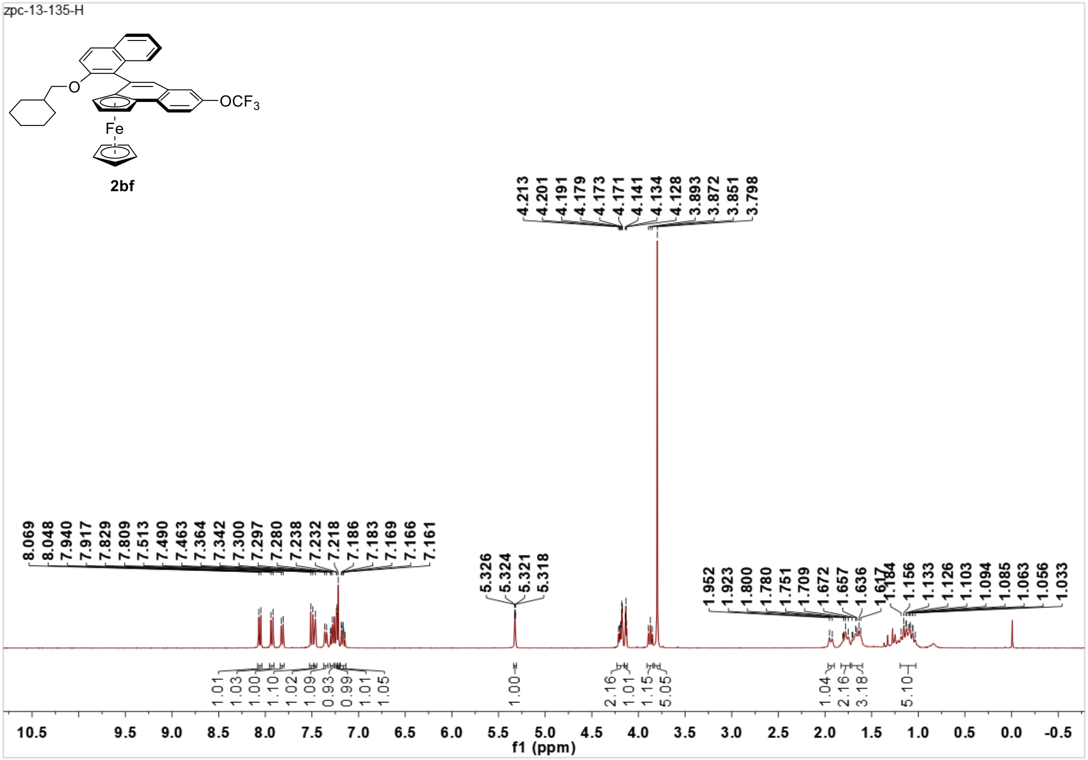

Supplementary Figure 167. ^{1}H NMR (400 MHz, $CDCl_3$) spectra for compound **2bf**

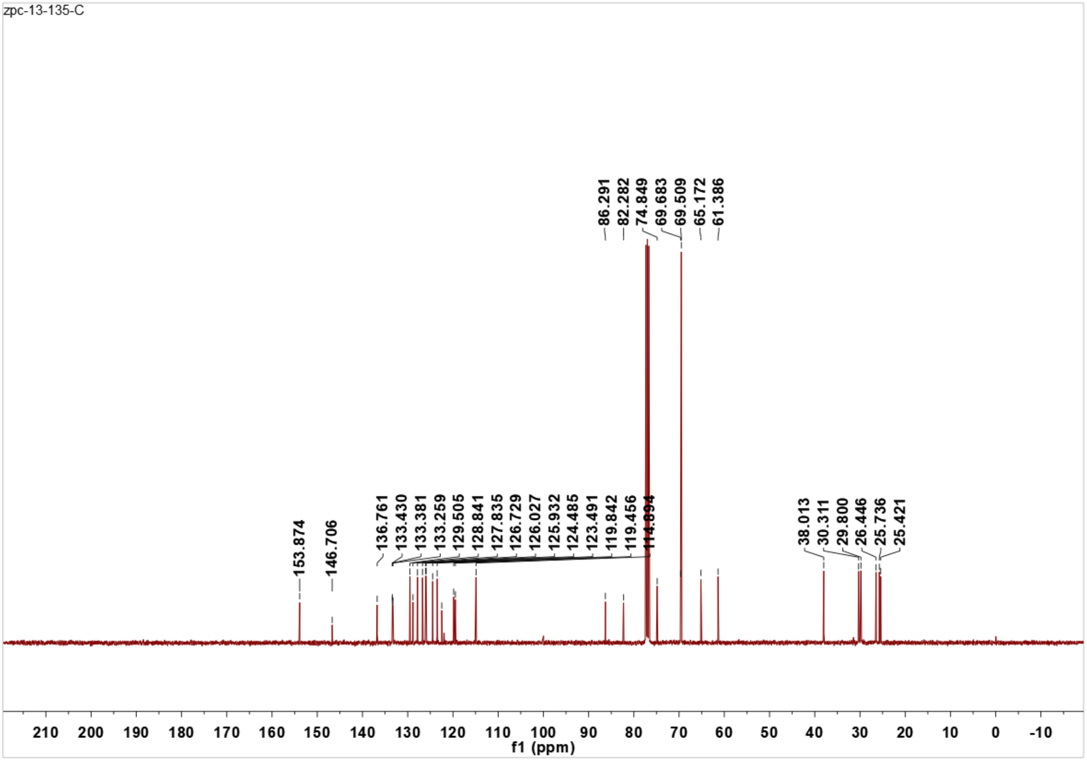

Supplementary Figure 168. ^{13}C NMR (400 MHz, $CDCl_3$) spectra for compound **2bf**

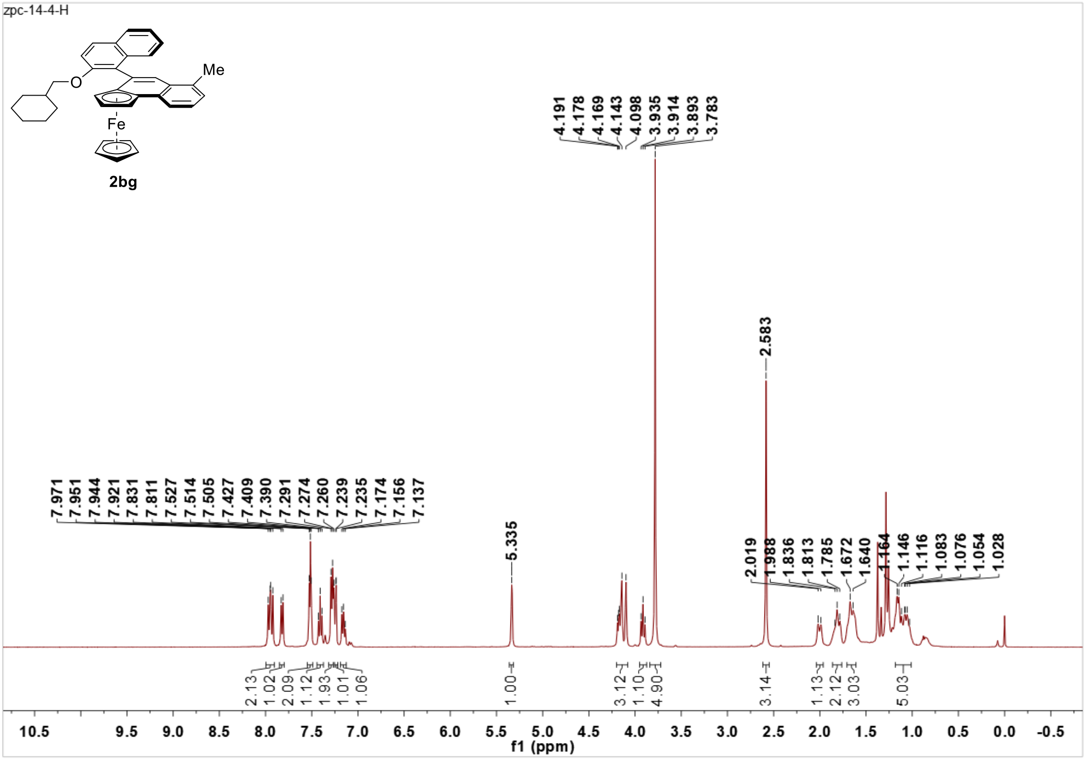

Supplementary Figure 169. ^{1}H NMR (400 MHz, $CDCl_3$) spectra for compound **2bg**

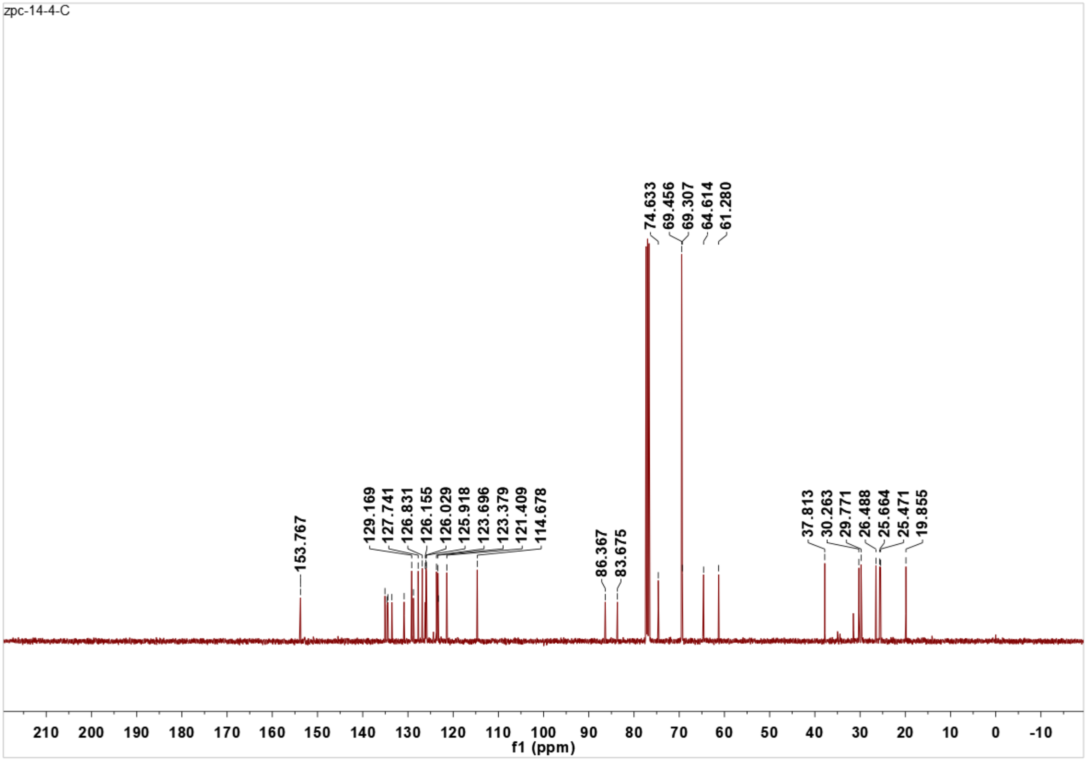

Supplementary Figure 170. ^{13}C NMR (400 MHz, $CDCl_3$) spectra for compound **2bg**

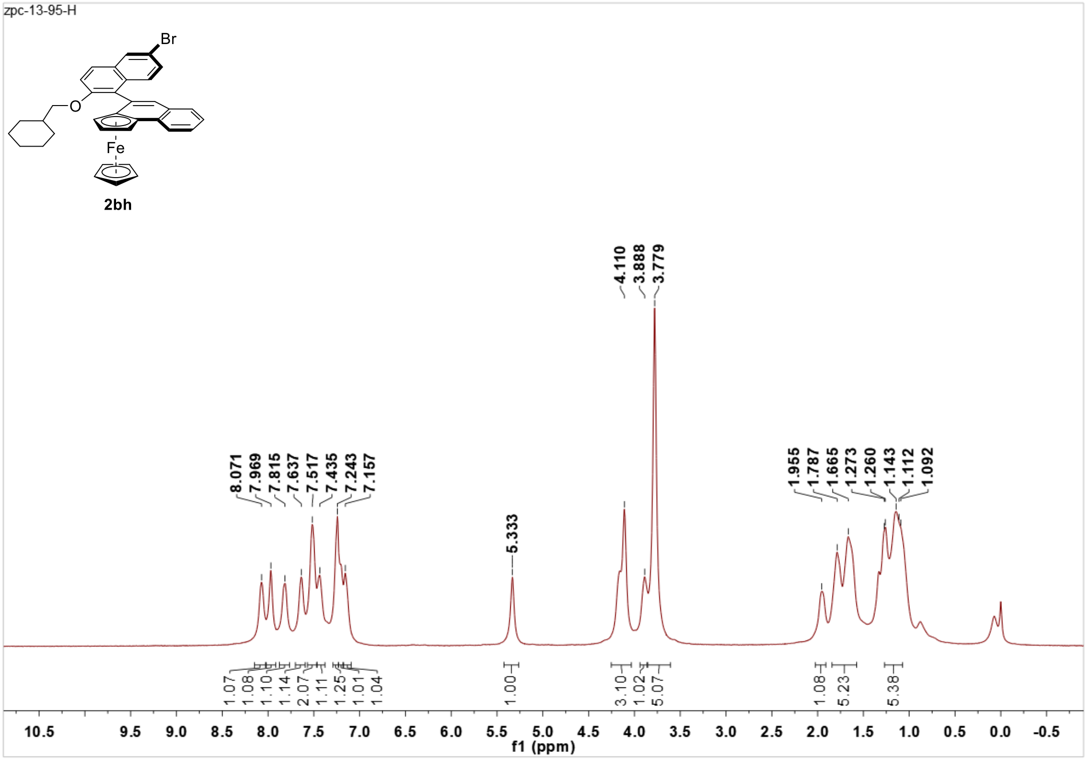

Supplementary Figure 171. ^{1}H NMR (400 MHz, $CDCl_3$) spectra for compound **2bh**

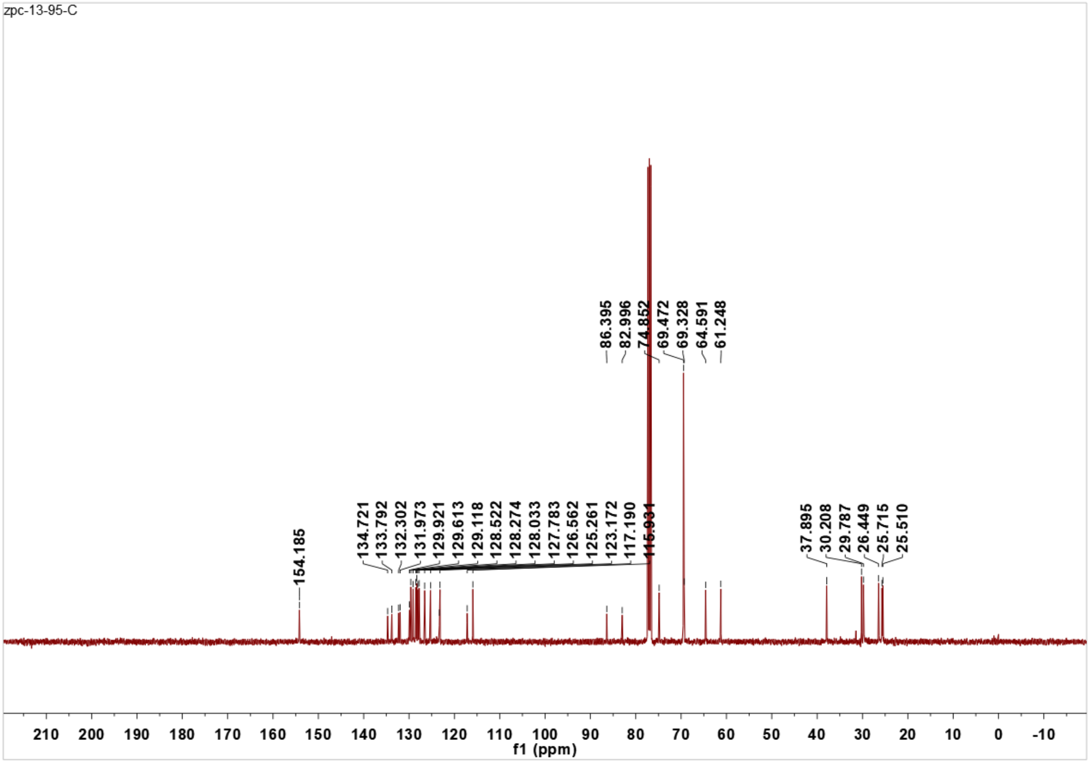

Supplementary Figure 172. ^{13}C NMR (400 MHz, $CDCl_3$) spectra for compound **2bh**

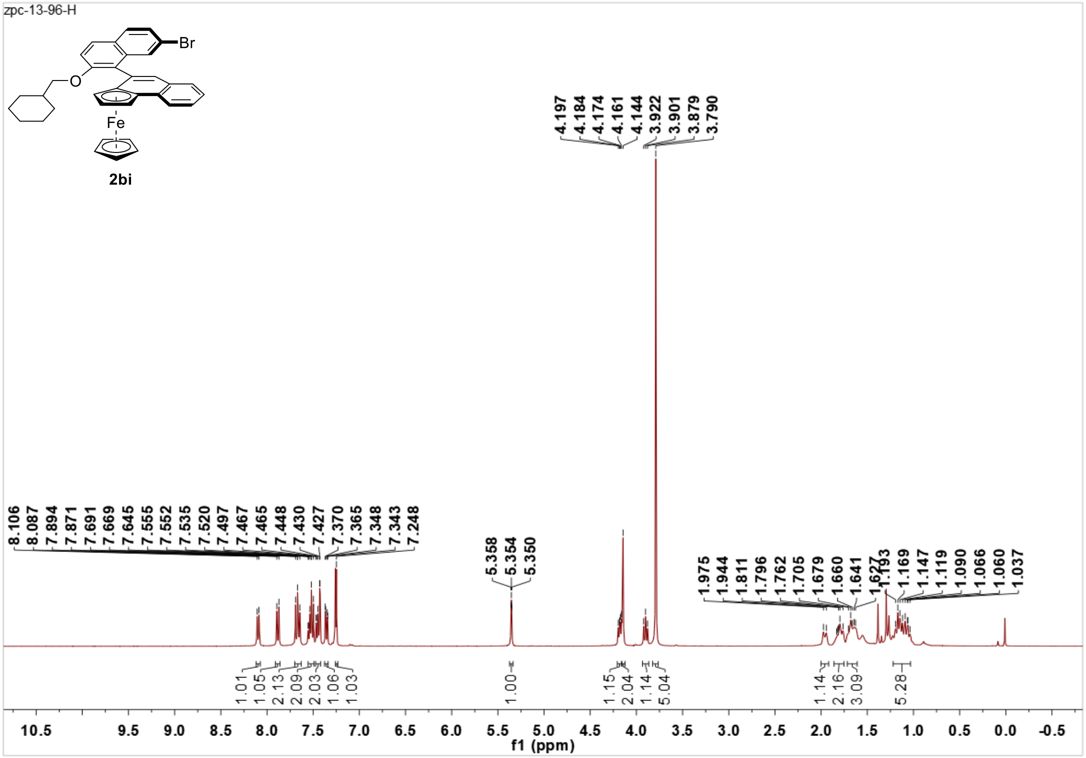

Supplementary Figure 173. ^{1}H NMR (400 MHz, $CDCl_3$) spectra for compound **2bi**

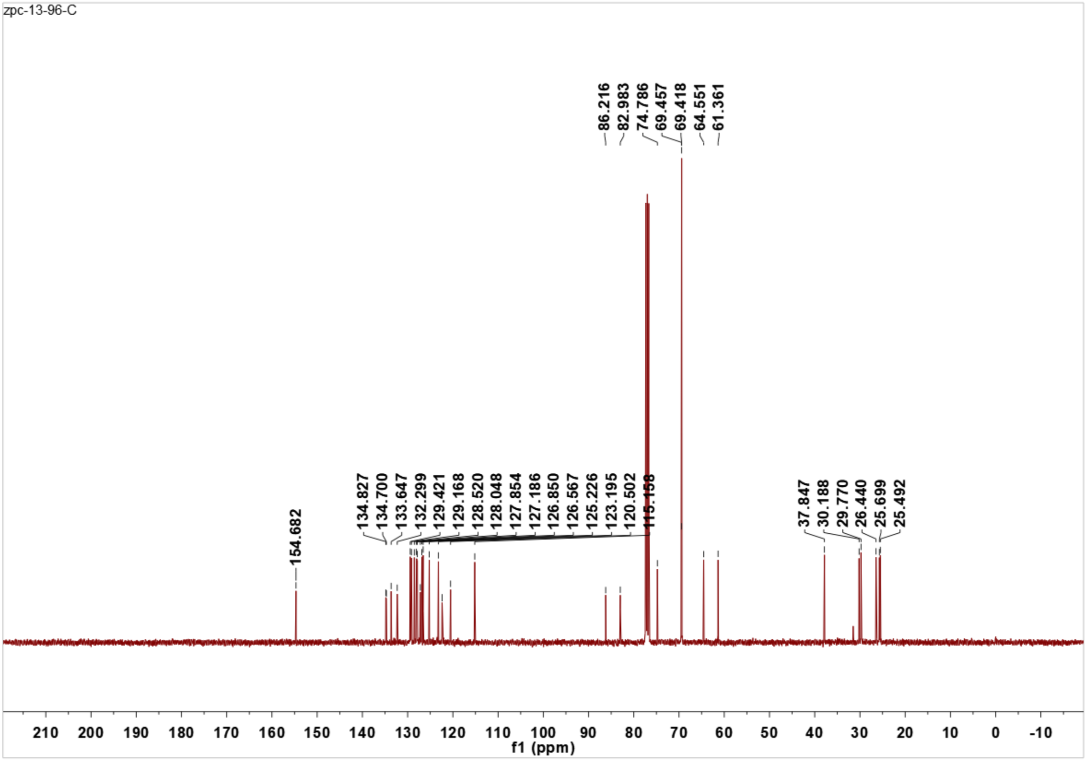

Supplementary Figure 174. ^{13}C NMR (400 MHz, $CDCl_3$) spectra for compound **2bi**

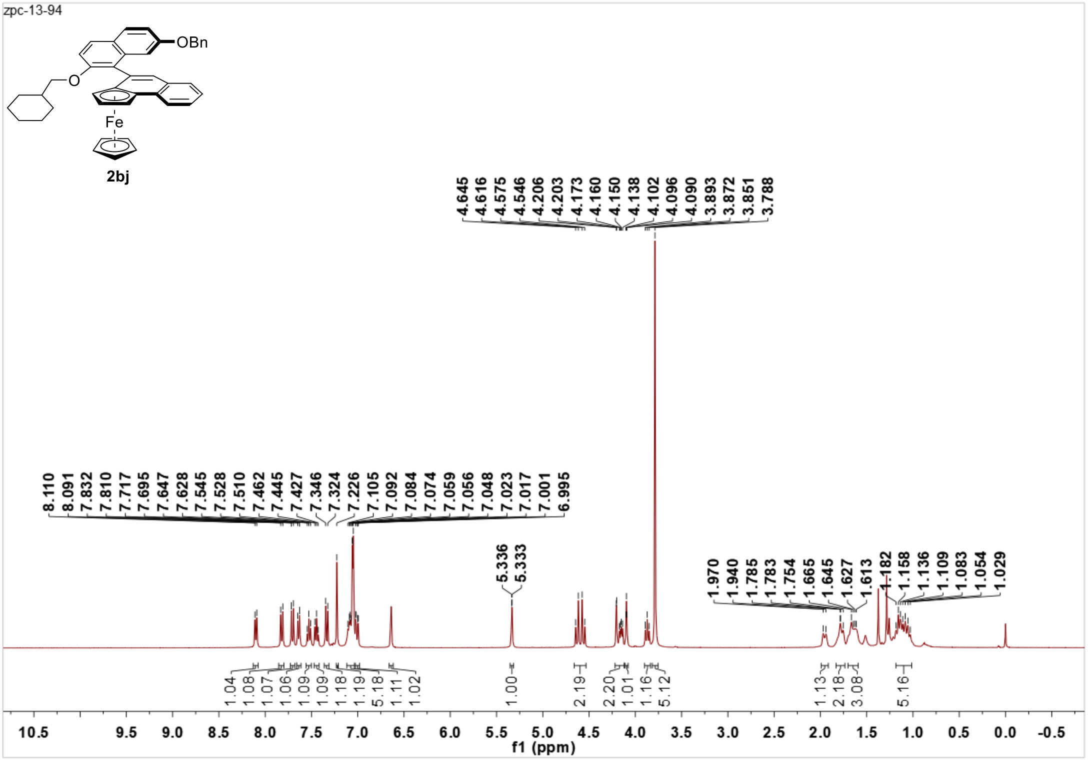

Supplementary Figure 175. ^{1}H NMR (400 MHz, $CDCl_3$) spectra for compound **2bj**

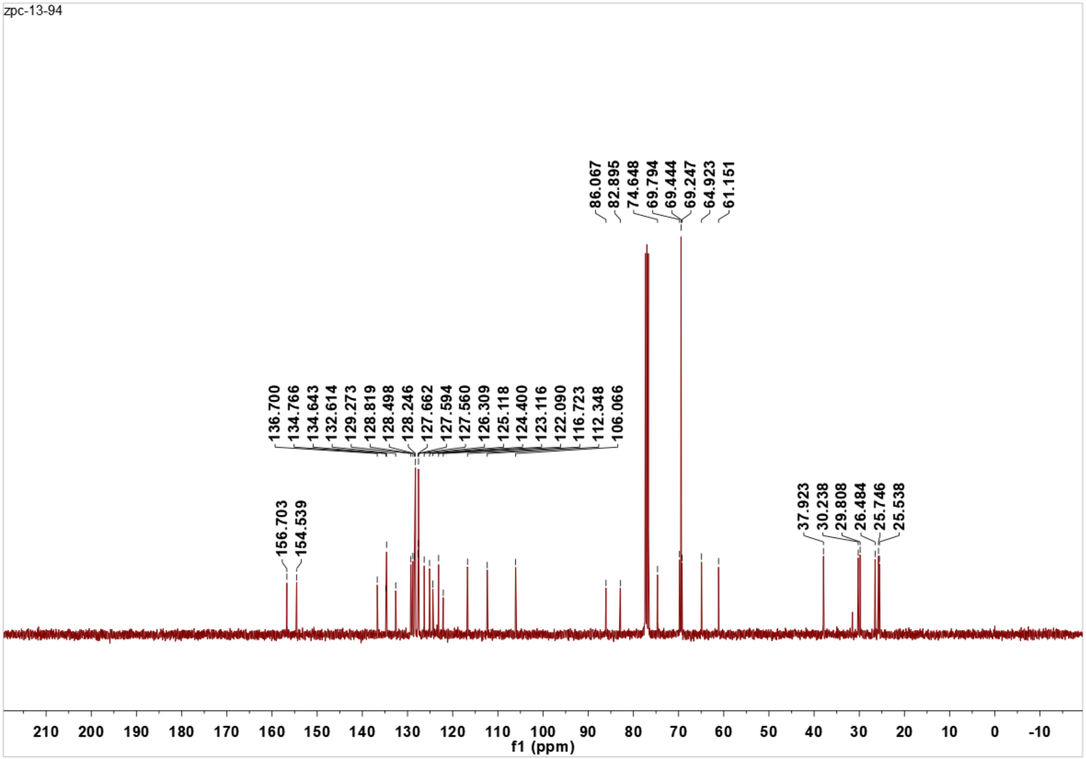

Supplementary Figure 176. ^{13}C NMR (400 MHz, $CDCl_3$) spectra for compound **2bj**

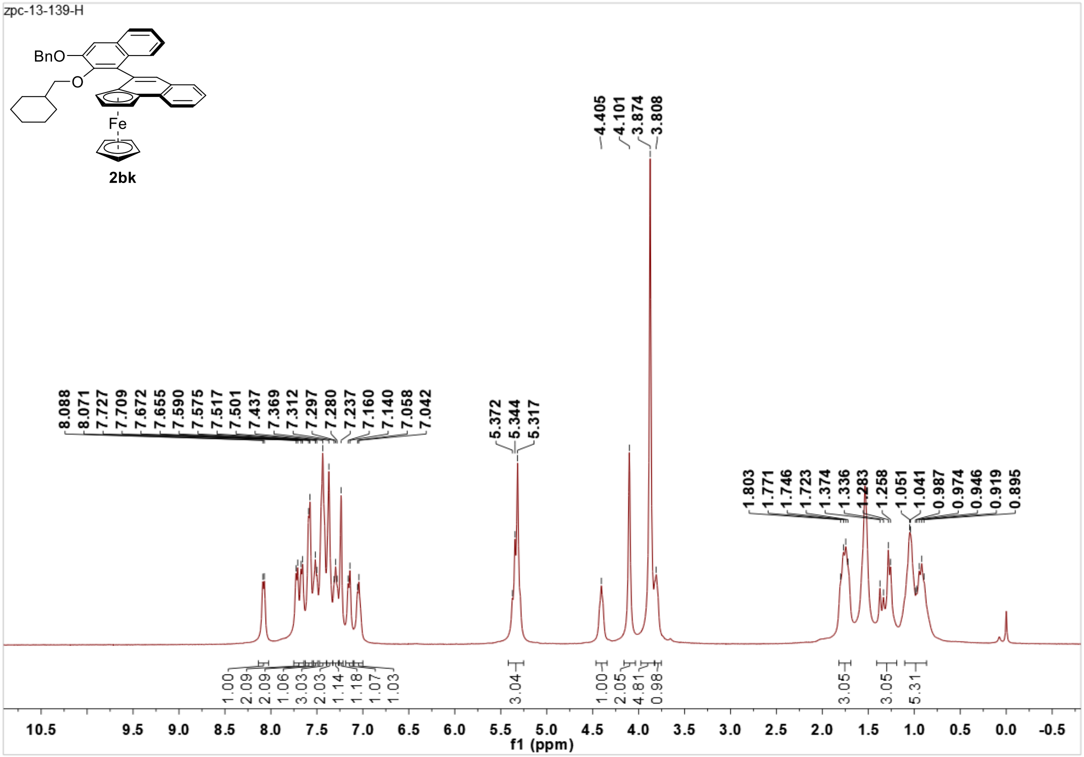

Supplementary Figure 177 ^{1}H NMR (400 MHz, $CDCl_3$) spectra for compound **2bk**

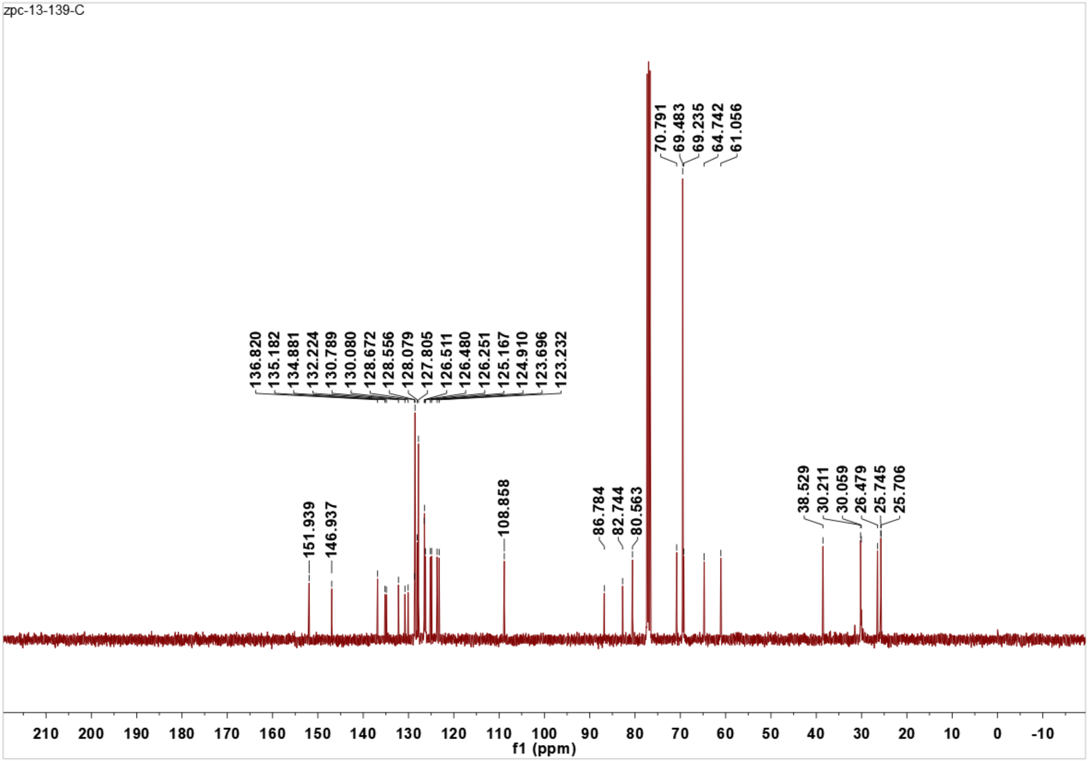

Supplementary Figure 178. ^{13}C NMR (400 MHz, $CDCl_3$) spectra for compound **2bk**

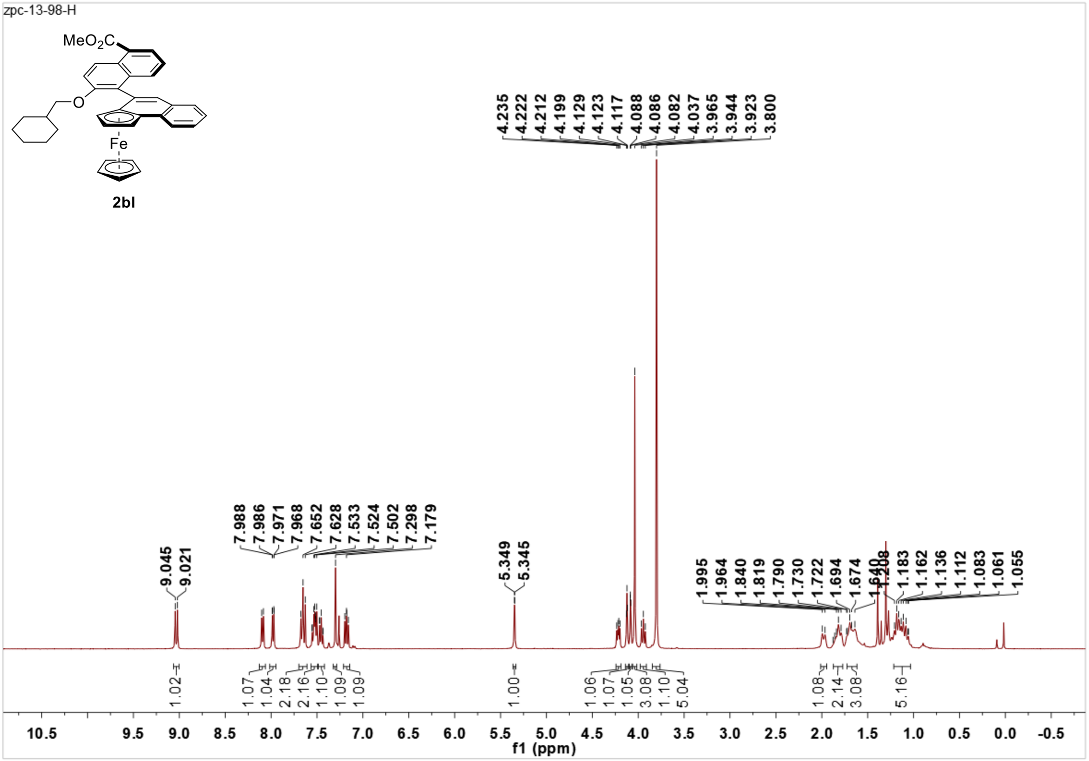

Supplementary Figure 179. ^{1}H NMR (400 MHz, $CDCl_3$) spectra for compound **2bl**

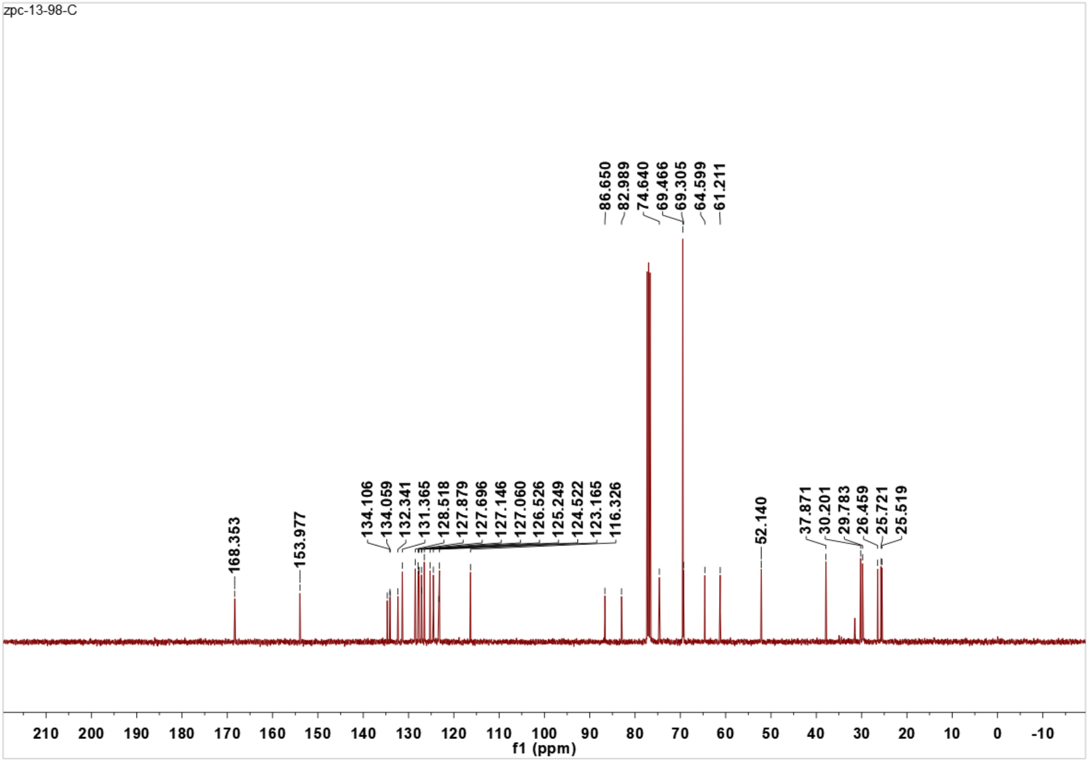

Supplementary Figure 180. ^{13}C NMR (400 MHz, $CDCl_3$) spectra for compound **2bl**

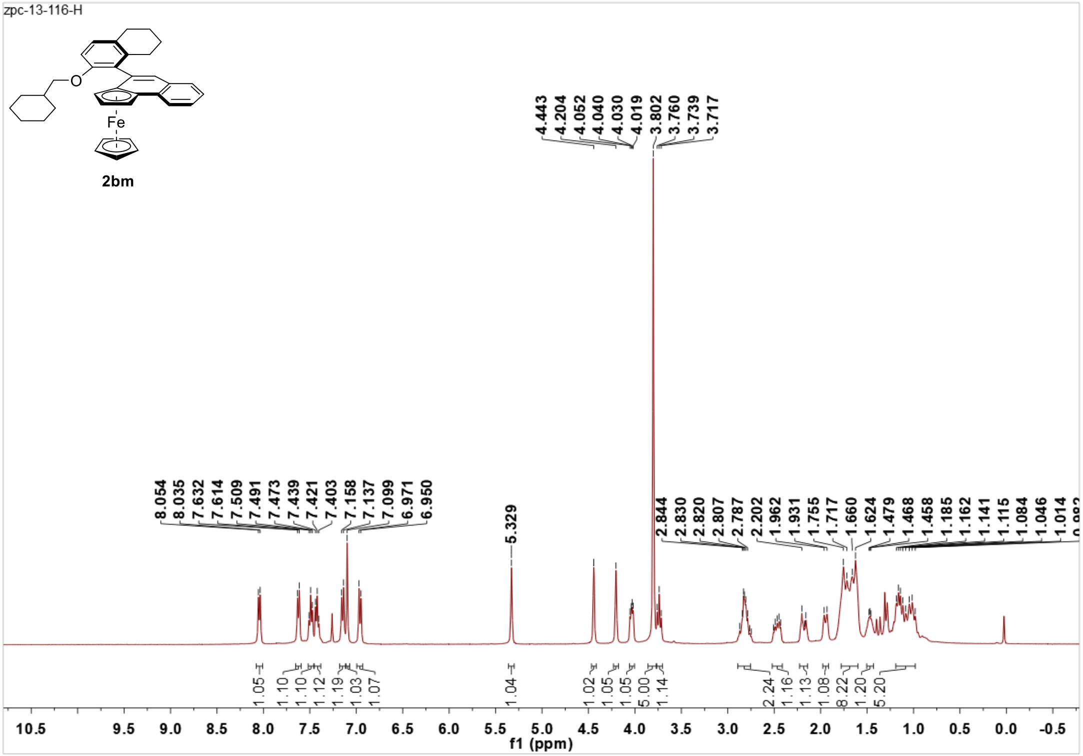

Supplementary Figure 181. ^{1}H NMR (400 MHz, $CDCl_3$) spectra for compound **2bm**

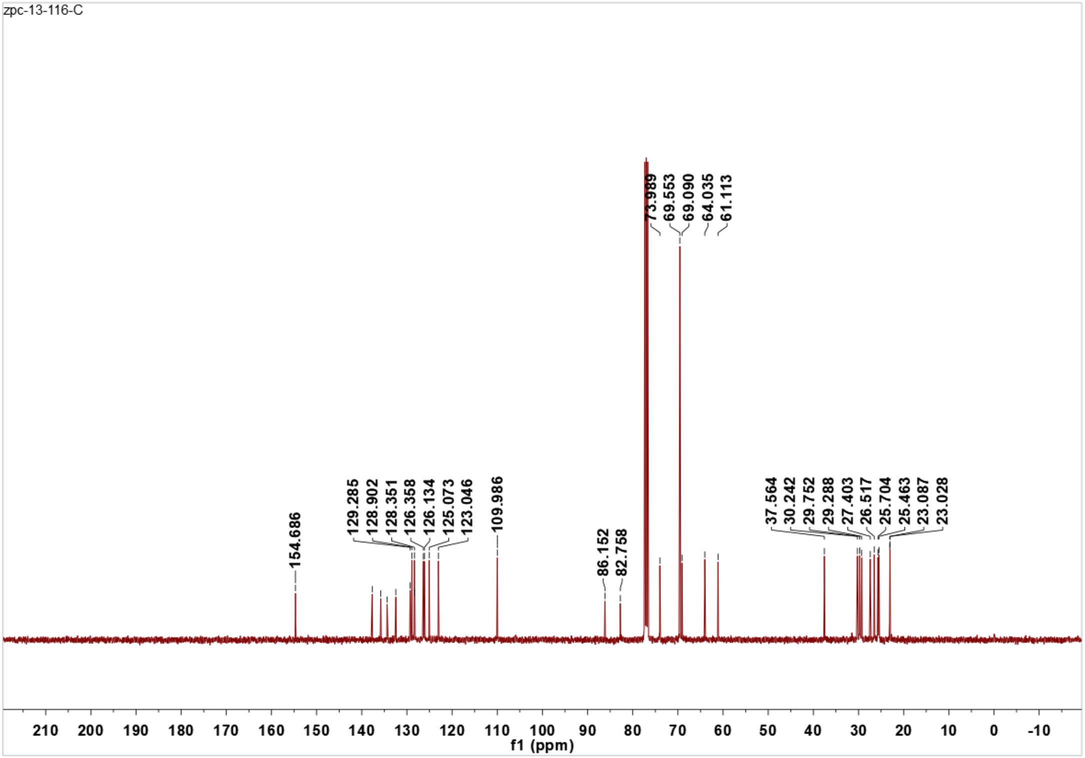

Supplementary Figure 182. ^{13}C NMR (400 MHz, $CDCl_3$) spectra for compound **2bm**

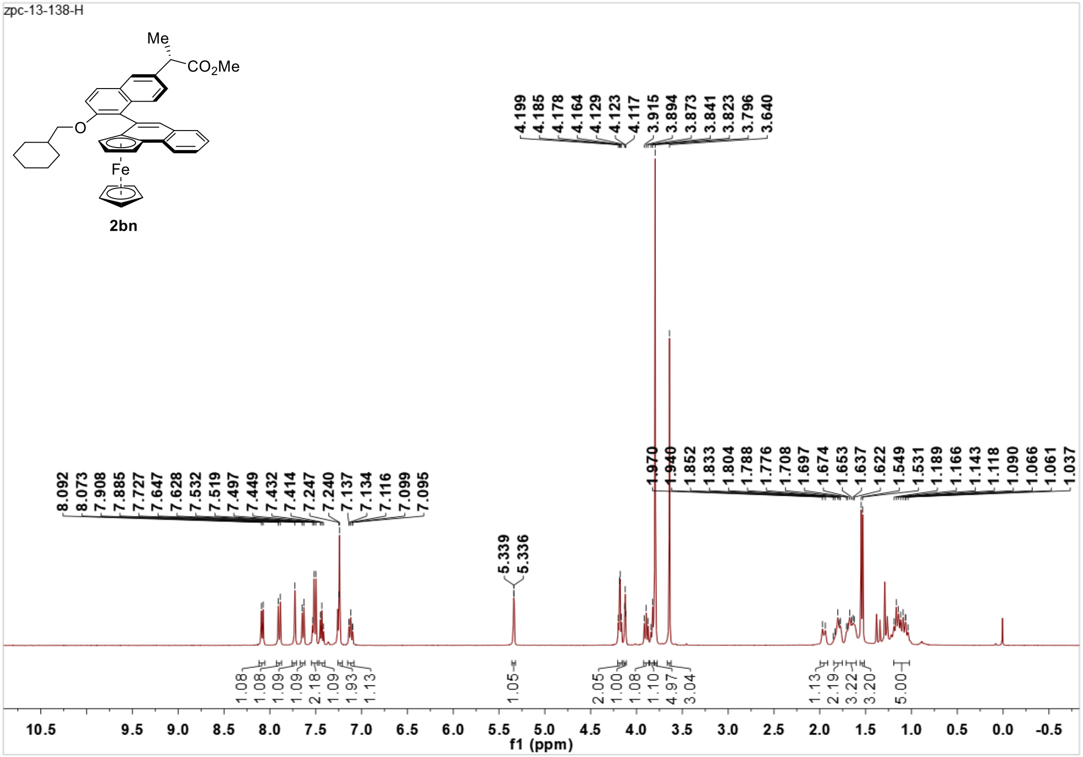

Supplementary Figure 183. ^{1}H NMR (400 MHz, $CDCl_3$) spectra for compound **2bn**

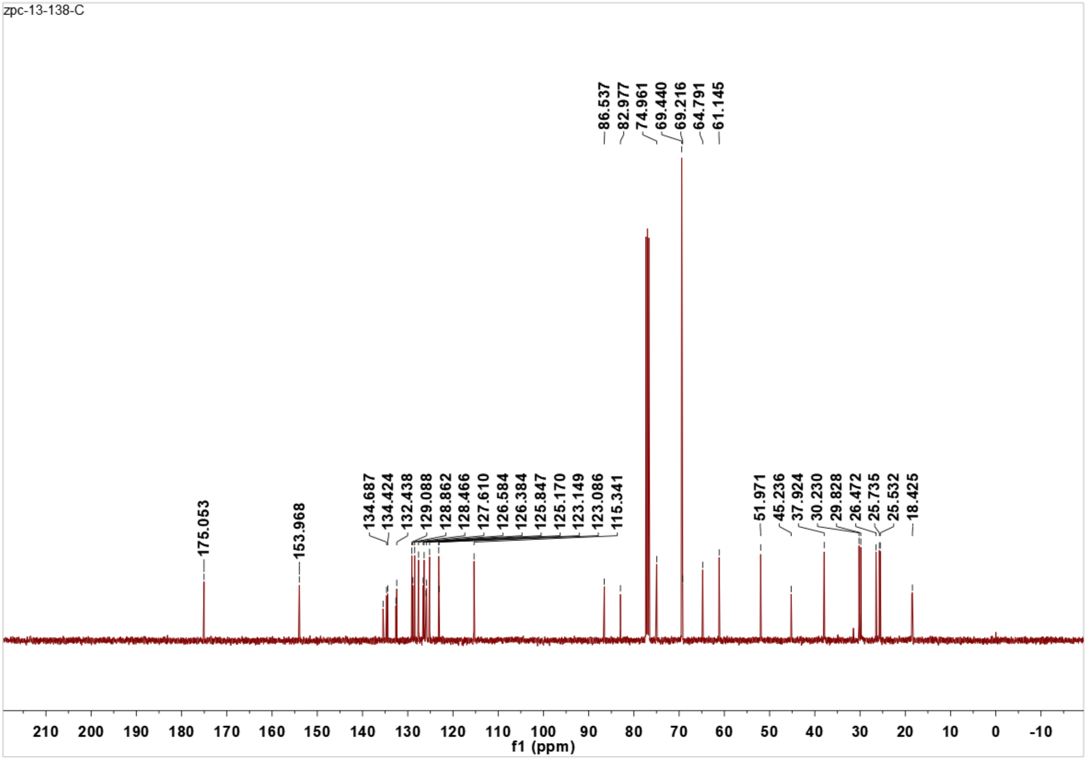

Supplementary Figure 184. ^{13}C NMR (400 MHz, $CDCl_3$) spectra for compound **2bn**

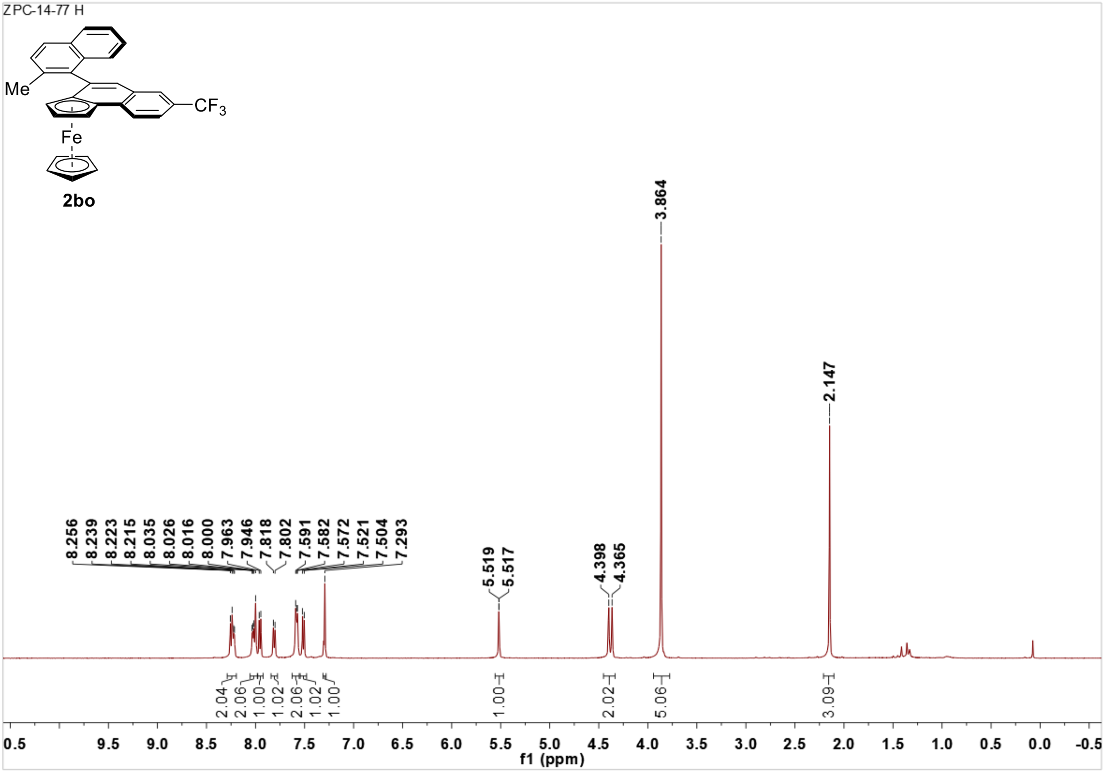

Supplementary Figure 185. ^{1}H NMR (500 MHz, $CDCl_3$) spectra for compound **2bo**

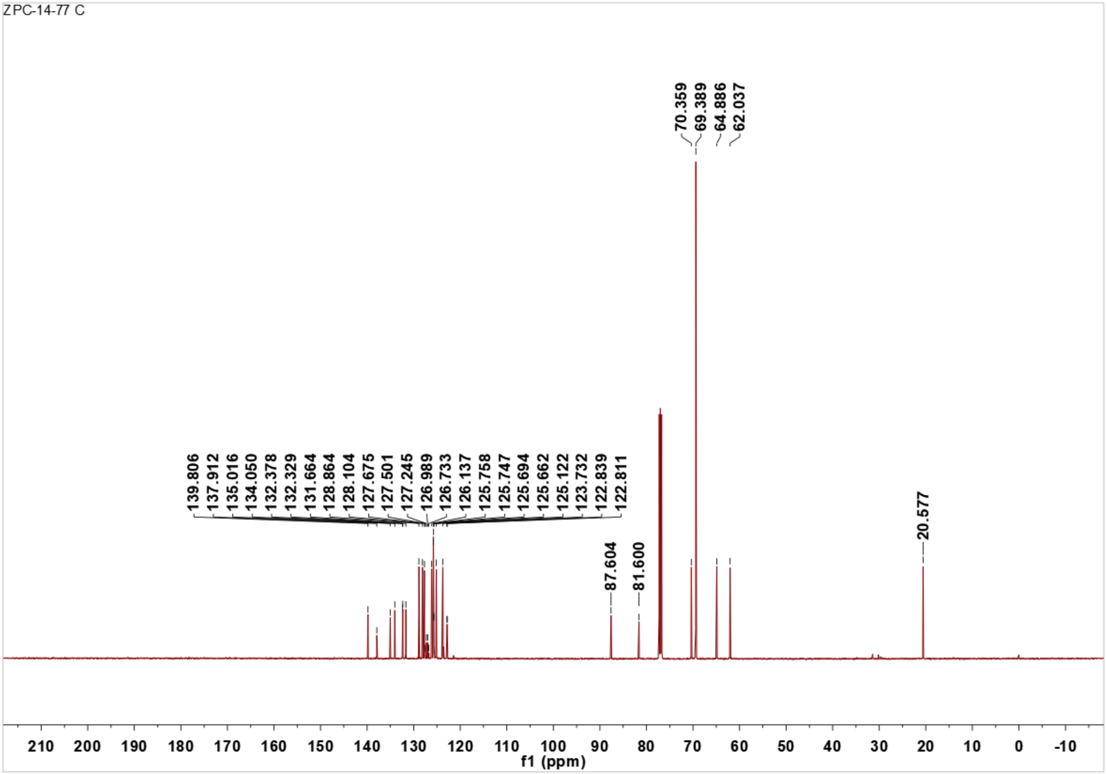

Supplementary Figure 186. ^{13}C NMR (500 MHz, $CDCl_3$) spectra for compound **2bo**

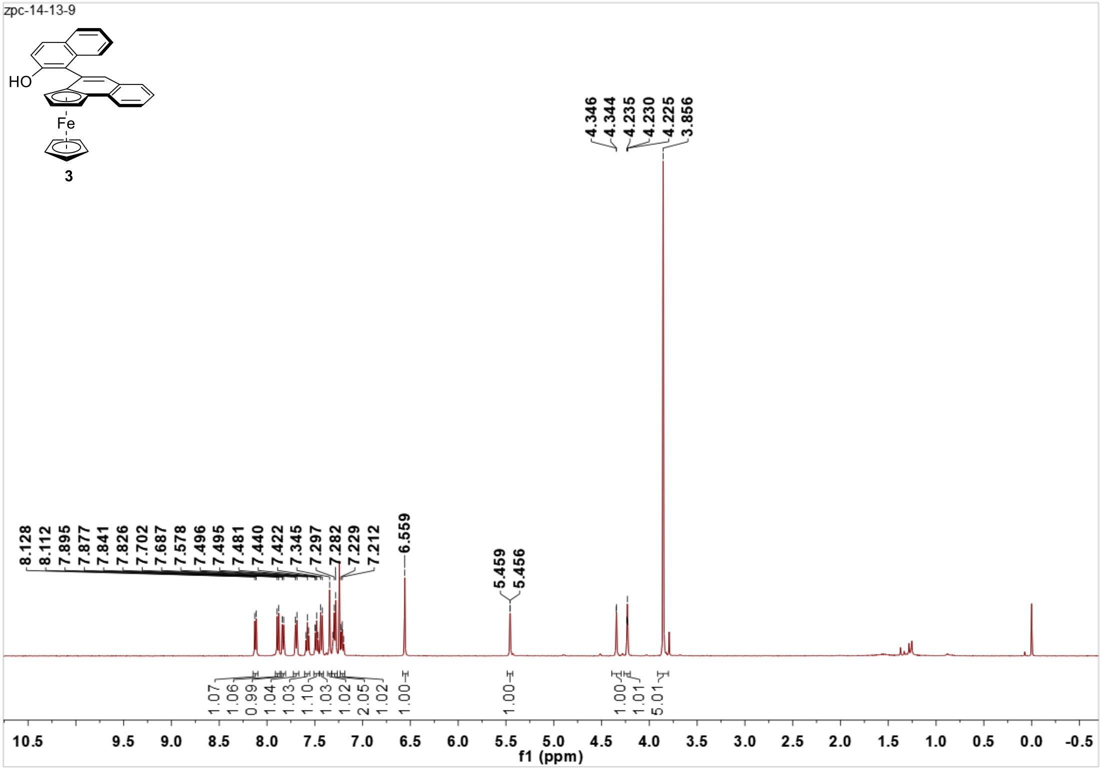

Supplementary Figure 187. ^{1}H NMR (500 MHz, $CDCl_3$) spectra for compound **3**

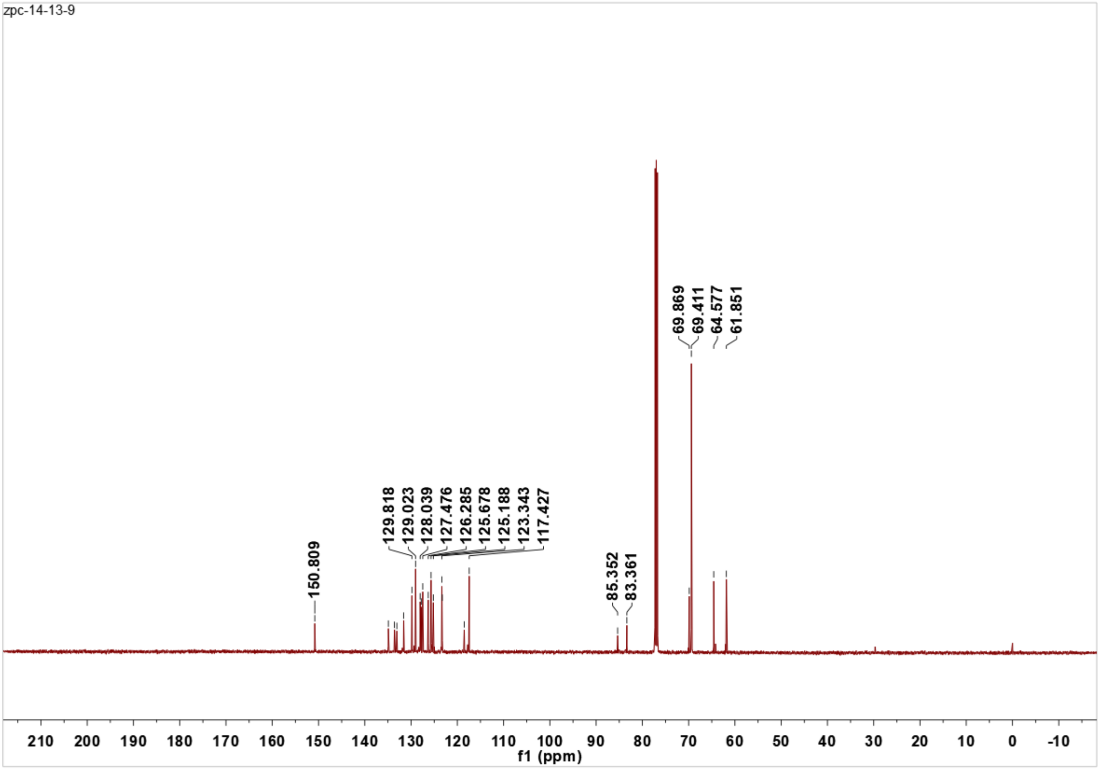

Supplementary Figure 188. ^{13}C NMR (500 MHz, $CDCl_3$) spectra for compound **3**

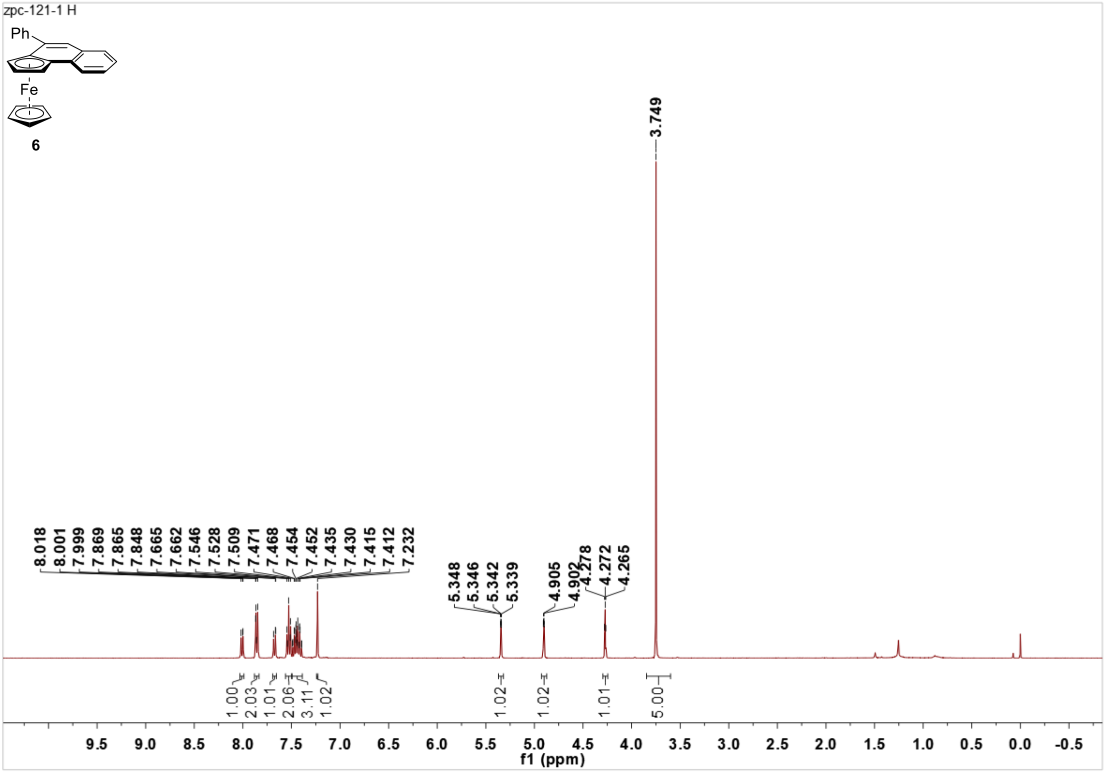

Supplementary Figure 189. ^{1}H NMR (400 MHz, $CDCl_3$) spectra for compound **6**

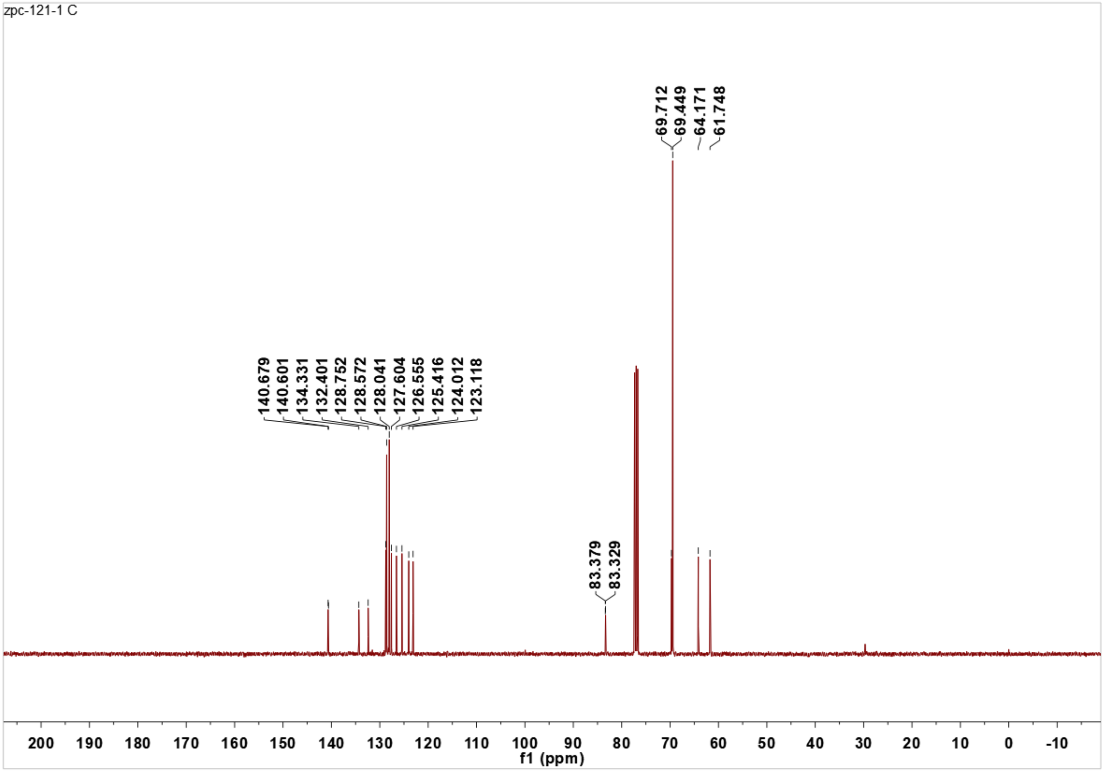

Supplementary Figure 190. ^{13}C NMR (400 MHz, $CDCl_3$) spectra for compound **6**

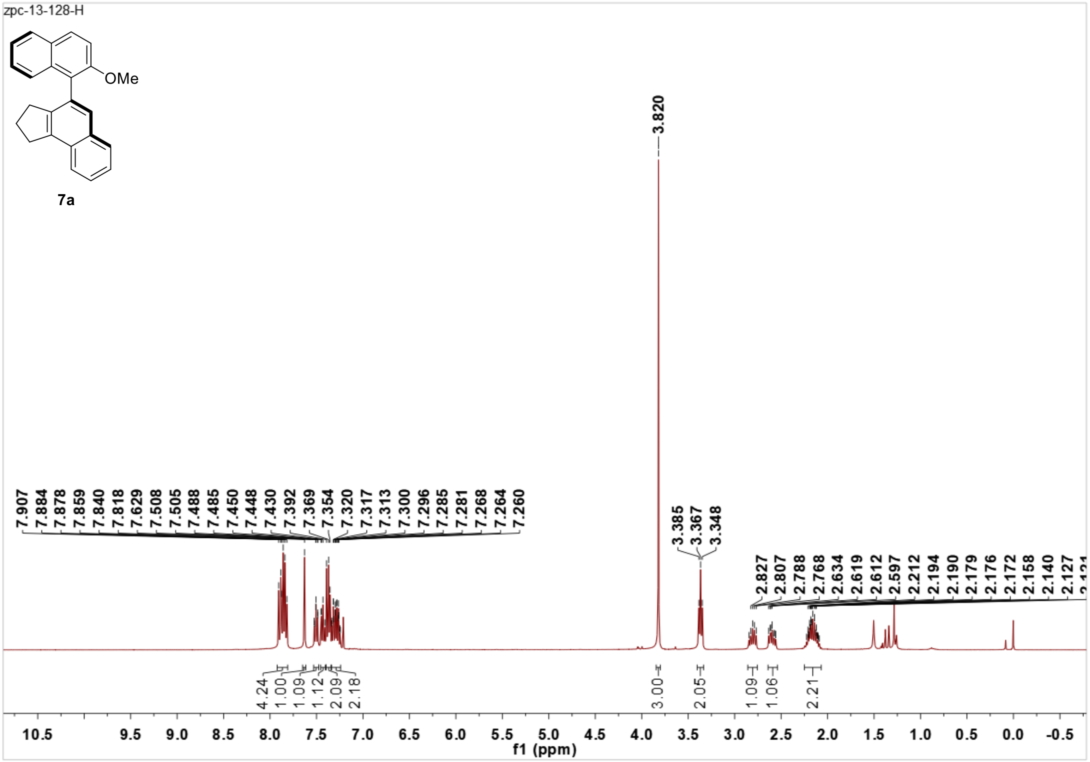

Supplementary Figure 191. ^{1}H NMR (400 MHz, $CDCl_3$) spectra for compound **7a**

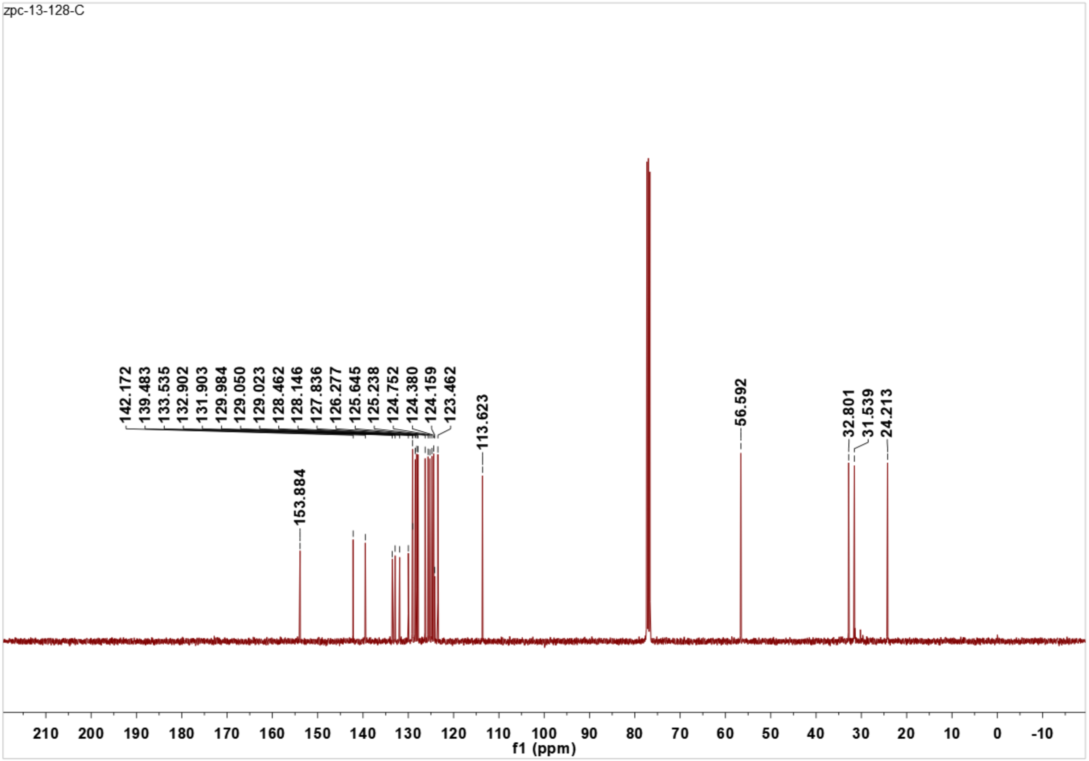

Supplementary Figure 192. ^{13}C NMR (400 MHz, $CDCl_3$) spectra for compound **7a**

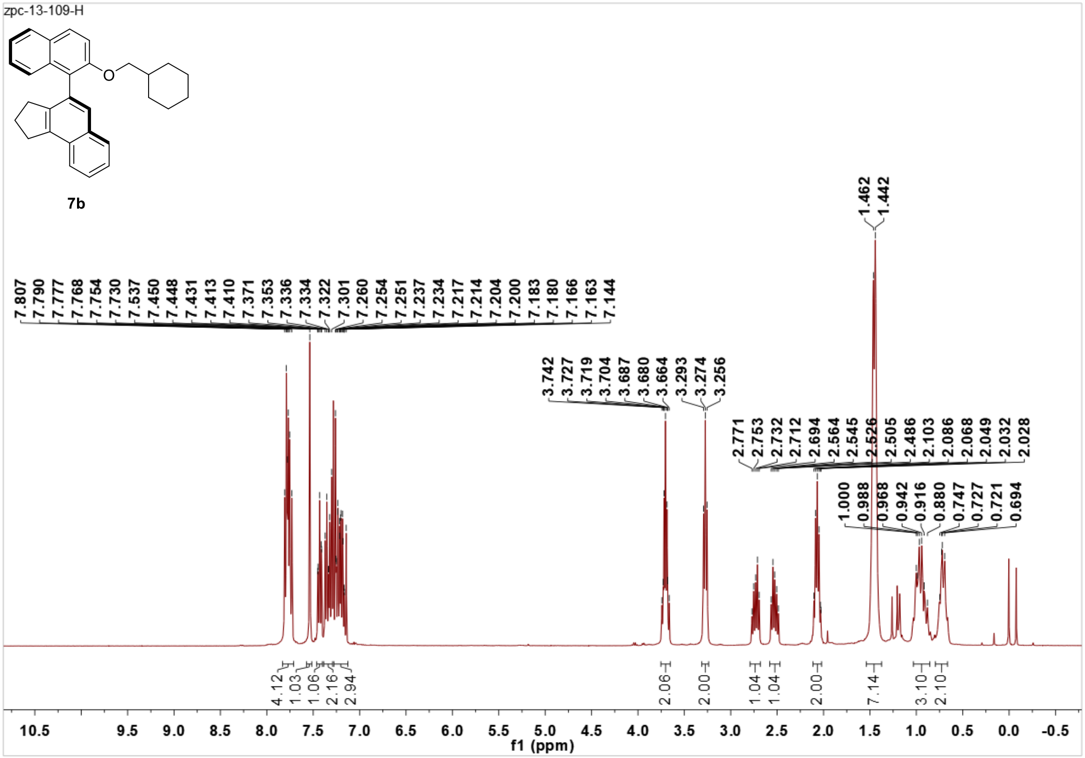

Supplementary Figure 193. ^{1}H NMR (400 MHz, $CDCl_3$) spectra for compound **7b**

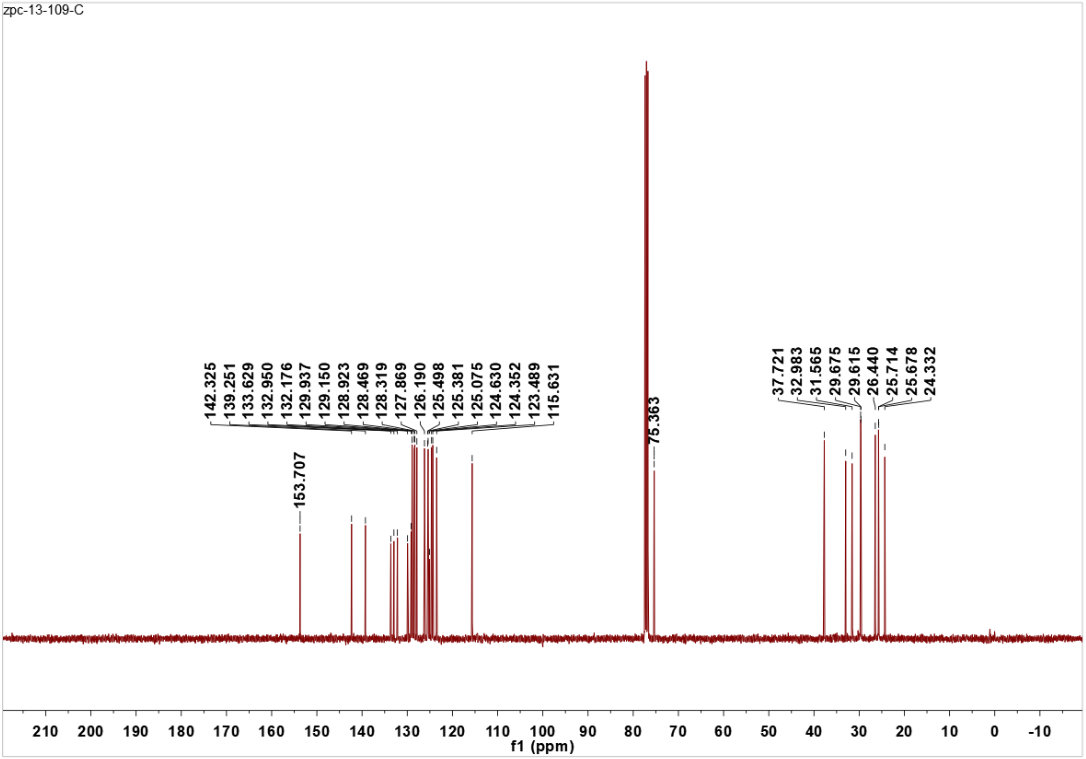

Supplementary Figure 194. ^{13}C NMR (400 MHz, $CDCl_3$) spectra for compound **7b**

12. Supplementary References.

1. Zhang, P.-C.; Wang, Y.; Qian, D.; Li, W.; Zhang, J. *Chin. J. Chem.* **2017**, *35*, 849-852.

2. Mu, D.; Yuan, W.; Chen, S.; Wang, N.; Yang, B.; You, L.; Zu, B.; Yu, P.; He, C. *J. Am. Chem. Soc.* **2020**, *142*, 13459-13468.

3. Kadoya, N.; Murai, M.; Ishiguro, M.; Uenishi, J.; Uemura, M. *Tetrahedron Lett*. **2013**, *54*, 512-514.

4. Zheng, S.-C.; Wang, Q.; Zhu, J. *Angew. Chem. Int. Ed.* **2019**, *58*, 1494-1498.
